# Clinical applicability of a new scoring system for population-based screening and risk factors of gastric cancer in the Wannan region

**DOI:** 10.1186/s12876-022-02384-w

**Published:** 2022-06-23

**Authors:** Lin Li, Jinjing Ni, Shenghong Sun, Xiaojuan Zha, Rong Li, Chiyi He

**Affiliations:** 1 Departments of Gastroenterology, Yijishan Hospital of Wannan Medical College, Wuhu, 241001 Anhui Province People’s Republic of China; 2 Departments of Health Management Center, Yijishan Hospital of Wannan Medical College, Wuhu, 241001 Anhui Province People’s Republic of China

**Keywords:** Gastric cancer, New scoring system, Gastroscopy

## Abstract

**Background:**

We aimed to evaluate the clinical applicability of a new scoring system that comprises the variables age, sex, pepsinogen ratio (PGR), gastrin-17 (G-17), and *Helicobacter pylori* (Hp) infection for gastric cancer (GC) screening in the Wannan region, China. We also explored the risk factors of GC in the Wannan region.

**Methods:**

We prospectively enrolled asymptomatic participants from January 1, 2019 to June 30, 2021 at the First Affiliated Hospital of Wannan Medical College. We used a receiver operating characteristic (ROC) curve to estimate the screening value of combined measurements of pepsinogen I, PGII, PGR, G-17, and Hp. Univariate analysis and multivariate analysis were used to explore the independent risk factors of GC.

**Results:**

A total of 25,194 asymptomatic patients were eventually screened. The area under the ROC curve (AUC) of combined measurements was 0.817 (95% confidence interval [CI] 0.721–0.913), the sensitivity was 81.5%, and the specificity was 77.8%. The detection rate of this new scoring system for GC screening in low-, medium-, and high-risk groups was 0%, 1.63%, and 9%, respectively (*P* < 0.001). Multivariate analysis showed that age (odds ratio [OR], 5.934; 95% CI 3.695–9.529; *P* < 0.001), sex (OR 5.721; 95% CI 2.579–12.695; *P* < 0.001), Hp infection (OR 1.992; 95% CI 1.255–3.163; *P* = 0.003), a history of smoking (OR 2.028; 95% CI 1.213–3.392; *P* = 0.007), consuming a high-salt diet (OR 2.877; 95% CI 1.807–4.580; *P* < 0.001), frequently eating pickled foods (OR 1.873; 95% CI 1.125–3.120; *P* = 0.016), and frequently eating fried foods (OR 2.459; 95% CI 1.384–4.369; *P* = 0.002) were independent risk factors for GC and precancerous lesions. However, frequent consumption of green vegetables (OR 0.388; 95% CI 0.242–0.620; *P* < 0.001) was an independent protective factor against GC and precancerous lesions.

**Conclusion:**

The new scoring system for GC screening was feasible in the Wannan region, especially in high-risk populations. Frequent consumption of green vegetables was an independent protective factor against GC and precancerous lesions.

**Supplementary Information:**

The online version contains supplementary material available at 10.1186/s12876-022-02384-w.

## Introduction

Gastric cancer (GC) remains the fifth most prevalent malignancy, posing a serious threat to human health. In early gastric cancer (EGC), patients who undergo endoscopic submucosal dissection (ESD) have a good prognosis, with a 5-year survival rate of over 90%. However, this rate in advanced gastric cancer (AGC) is less than 10% [1]. Thus, it is particularly important to develop a screening method to improve the detection of EGC. The gold standard for diagnosing GC is gastroscopy and histopathological examination [2]. However, gastroscopy is not well accepted by the general population because it is invasive and causes discomfort. Recently, serum pepsinogen (PG), gastrin-17 (G-17), and *Helicobacter pylori* (Hp) antibody indicators have been used in non-invasive detection methods for diagnosing atrophic gastritis [3, 4], and improving the detection of GC. PG is secreted by the gastric mucosa and includes PGI and PGII. PGI is secreted by the gastric fundic glands, and PGII is secreted by the gastric fundic glands, pylorus, and Brunner glands. However G-17 is only secreted by gastric antral G cells [5]. Serum levels of PG and G-17 reflect the morphology and functional status of the gastric mucosa [6, 7] and can be used as predictive indicators for atrophic gastritis and EGC. Hp, which is involved in the occurrence and development of GC through its pathogenic virulence factors and immune response after infection [8], has been listed as a class I carcinogen by the World Health Organization [9]. Moreover, Hp is associated with 90% of cases of non-cardiac GC [10].

Li et al. [11] developed and validated a new scoring system, which comprised the variables age, sex, the pepsinogen ratio (PGR), G-17, and anti-Hp IgG status, to provide accurate risk stratification for further GC screening in the Chinese population.

There are obvious regional differences in the distribution of GC in China [12]. The Wannan region, which is located in the southern part of Anhui Province in Eastern China, is one area with high morbidity and mortality of GC. According to the past investigation [13], the morbidity of GC in Anhui Province in 2015 was 43.85/100,000 population, and the mortality was 31.22/100,000, far higher than the national level. Currently, greater attention is being given to the lifestyle habits of patients with GC. Residents in Anhui Province are fond of eating high-salt and pickled foods. Zhao et al. [14] found that high-salt foods and pickled foods can produce high levels of carcinogens such as N-nitroso compounds, which might cause atrophy and structural destruction of the human gastric mucosa. Other studies [15–17] have pointed out that high salt intake aggravates damage to the gastric mucosa, hypergastrinemia, cell proliferation, and is associated with Hp infection.

The purpose of this study was to evaluate the clinical applicability of a new scoring system, which comprised the variables age, sex, PGR, G-17, and Hp infection for GC screening in the Wannan region. We also aimed to explore the risk factors of GC in the Wannan region.

## Patients and methods

### Study population

We prospectively collected and surveyed asymptomatic patients between January 1, 2019 and June 30, 2021 at Yijishan Hospital of Wannan Medical College, Anhui, China. All study participants volunteered to participate in this study and signed the informed consent form. Serum levels of PGI, PGII, and G17 in participants aged ≥ 40 years were measured. In our study, Hp infection status was determined using the ^13^C/^14^C-urea breath test (^13^C/^14^C-UBT). We excluded participants as follows: (1) patients with an upper abdominal mass or unplanned weight loss of more than 4.5 kg in the past 6 months or those with symptoms such as dysphagia, abdominal pain, melena, hematemesis, and vomiting; (2) patients taking proton pump inhibitors (PPIs) or H_2_ blockers in the past 2 weeks; (3) patients with a history of Hp eradication; (4) patients with previous gastric operations, chemotherapy or radiotherapy; (5) pregnant or lactating patients; and (6) patients with other serious systemic diseases or mental symptoms and who were unable to participate. This study was approved by the Ethics Committee of Yijishan Hospital of Wannan Medical College (SR-2020-05).

### Screening procedures

All included participants were asked to complete a questionnaire. Fasting blood samples were collected for serum detection of PGI, PGII, PGR and G-17. Additional ^13^C/^14^C-UBT tests were performed to determine the status of Hp infection. According to the new scoring system for GC screening [11], as shown in Table 1, scores 0 to 11, 12 to 16, and 17 to 23 were combined to form three categories, corresponding to groups with a low, medium, and high risk of GC, respectively. With increased score, the risk of GC was increased. Patients with a medium or high risk of GC were advised to undergo gastroscopy by telephone.

**Table 1:** The new scoring system for GC screening in Chinese population

Variable	Classification	Score
Age	40–49	0
	50–59	5
	60–69	6
	> 69	10
Sex	Female	0
	Male	4
Hp	Negative	0
	Positive	1
PGR	≥ 3.89	0
	< 3.89	3
G-17 (pmol/L)	< 1.5	0
	1.5–5.7	3
	≥ 5.7	5

*EGC* early gastric cancer, *Hp* *Helicobacter pylori*, *PGR* pepsinogen ratio, *G-17* gastrin-17

### *Serological detection and *^*13*^*C/*^*14*^*C-UBT test*

Fasting blood samples (5 mL) were collected and then centrifuged at 3000 rpm for 3 min, and the serum was collected. The serum was stored in − 80 ℃. PGI, PGII, PGR, and G-17 concentrations were detected using enzyme-linked immunosorbent assay (ELISA) (Biohit, Helsinki, Finland) according to the manufacturer’s instructions. The concentrations of each measure were tested in duplicate. The normal detection concentration range of PGI, PGII, PGR, and G-17 is 70–165 μg/L, 3–15 μ g/L, 7–20, and 1–15 pmol/L, respectively.

After an 8-h overnight fast, participants were requested to exhale into a collection bag and then swallow ^13^C/^14^C labelled urea dissolved in water (200 mL); after 30 min, participants were asked to exhale into a second collection bag. The normal upper limit of ^13^C and ^14^C is 4 dpm and 100 dpm, respectively.

### Endoscopic and histological examinations

Patients with a medium or high risk of GC were advised to undergo gastroscopy. Before gastroscopy, patients were required to sign an informed consent form for the procedure. White light endoscopy, narrowband imaging endoscopy, magnification endoscopy or indigo lipid staining was used, as appropriate. Gastroscopy was performed by expert endoscopists who had performed endoscopy examination or therapy in more than 2000 cases. At least three biopsies were collected from each participant and each specimen was reviewed independently by two independently.

According to the endoscopic and histological results, patients were divided into a non-atrophic gastritis group, atrophic gastritis group, gastric ulcer group, non-neoplastic polyp group, precancerous lesion group, EGC group, and AGC group. The non-atrophic gastritis group included chronic superficial gastritis and erosive gastritis. Precancerous lesions included intestinal-type gastritis and low-grade intraepithelial neoplasia (LGIN). High-grade intraepithelial neoplasia (HGIN) and intramucosal carcinoma were included in the EGC group [18–20].

### Questionnaire survey

The questionnaire included basic information for age, sex, body mass index (BMI), history of smoking, alcohol consumption, and family history of GC. We also collected information on patients’ dietary habits including questions to determine dietary intake, whether patients consumed a high-salt diet, and the frequency of consuming certain foods (fried, barbecued, pickled, smoked,and overnight leftover foods; green vegetables; and fresh fruits) consumption. We defined a history of smoking as smoking more than one cigarette every day for more than 1 year and a history of drinking was defined as drinking alcohol of any types more than once a week for more than 1 year. A high-salt diet was defined as eating more than six grams of salt per day. The frequency of consuming fried, barbecued, preserved, smoked, overnight leftover foods; green vegetables; and fresh fruits was recorded as once per week, twice per week, or more than three times per week. We defined frequent consumption of the above foods as more than three times per week.

### Database of the Wannan region

We established a hospital database (https://mgr.scyllatech.cn/researchmgr) for the management of the data in our study. Participants’ ID number, age, sex, BMI, history of smoking and alcohol consumption, dietary habits, serological test results, status of Hp infection, endoscopic and pathological results, and treatment and follow-up records were all entered in this database. All information related to study participants was kept confidential.

### Statistical analysis

We used IBM SPSS26.0 (IBM Corp., Armonk, NY, USA) to analyze the data. Measurement data are presented as mean ± standard deviation (SD), and counting data are presented as number (%). We used one-way analysis of variance (ANOVA), the chi-square test, or Fisher's exact test in the analysis, as appropriate. The combined detection of PGI, PGII, PGR, G-17, and Hp for diagnosing GC was determined using receiver operating characteristic (ROC) curves. Additionally, univariate and multivariate logistic regression analyses were carried out to estimate the risk factors of GC. *P* < 0.05 was considered statistically significant.

## Results

### Patient characteristics

A total of 25,194 asymptomatic participants were surveyed. According to the new scoring system for EGC screening [11], a total of 8009 patients were categorized as having a medium–high risk of GC, among which 7360 (91.90%) patients were classified as having a medium risk of GC and 649 (8.10%) as having a high risk of GC. The age of the medium–high GC risk group ranged from 41 to 89 years, and included 7186 men with average (± SD) age of 57.73 (± 7.38) years and 823 women with an average (± SD) age of 66.32 (± 7.31) years; the sex ratio was 8.73:1.

### Results of gastroscopy

Participants with a medium or high risk of GC were advised to undergo gastroscopy, and 1019 individuals finally completed gastroscopy; the response rate for gastroscopy was 12.72%. Of these, we identified 655 participants with non-atrophic gastritis (388 with chronic superficial gastritis, 267 with erosive gastritis), 52 patients with atrophic gastritis, 109 with gastric ulcer, 79 with non-neoplastic polyps, 94 with precancerous lesion (69 patients with intestinal-type gastritis, 25 with LGIN), 12 patients with early-stage cancer (9 with HGIN, 1 with gastric intramucosal carcinoma, 2 with early esophageal carcinoma), 15 patients with advanced cancer (14 with AGC, 1 with advanced esophageal carcinoma), and 3 patients with gastric stromal tumor. Additionally, 648 participants with a low risk of GC underwent gastroscopy and no GC cases were identified (Table 2).

**Table 2:** Characteristics of patients underwent gastroscopy in the medium–high risk of GC groups

Characteristics	Overall (%)
Overall	1019 (100)
Age	
40–49	9 (0.89)
50–59	631 (61.92)
60–69	271 (26.59)
> 69	108 (10.6)
Sex	
Female	166 (16.29)
Male	853 (83.71)
Hp	
Negative	522 (51.23)
Positive	497 (48.77)
PGR	
< 3.89	110 (10.79)
≥ 3.89	909 (89.21)
G-17	
≤ 1.50	7 (0.69)
1.50–5.70	494 (48.48)
> 5.70	518 (50.83)
Risk level	
Moderate risk group	919 (90.19)
High risk group	100 (9.91)
Gastroscopy result	
Chronic superficial gastritis	388 (38.08)
Erosive gastritis	267 (26.2)
Non-neoplastic polyps	79 (7.75)
Gastric ulcer	109 (10.7)
Atrophic gastritis	52 (5.1)
Intestinal-type gastritis	69 (6.77)
LGIN	25 (2.45)
Gastric stromal tumor	3 (0.29)
Early cancer	12 (1.18)
Advanced cancer	15 (1.47)

*GC* gastric cancer, *Hp Helicobacter pylori*, *PGR* pepsinogen ratio, *G-17* gastrin-17, *LGIN* low-grade intraepithelial neoplasia

Among the 14 patients with AGC, lesions located in the gastric antrum, cardia, gastric body, and gastric angle were found in 5, 3, 3, and 3 patients, respectively. Two patients had high differentiation, eight had medium differentiation, and four patients had low differentiation. Additionally, four patients had infiltration of the muscle layer and 10 had infiltration of the serous layer. Ten patients with EGC and three patients with gastric stromal tumor were completely resected via ESD. Of the 25 patients with LGIN, 11 requested treatment, and all lesions were successfully removed using ESD.

### Serum PG and G-17 and Hp status in each group

As shown in Table 3, compared with the group who had non-atrophic gastritis as the control group, the serum levels of PGI were significantly increased in the gastric ulcer group (*P* < 0.05); however, the atrophic gastritis group, precancerous lesion group, EGC group, and AGC group had lower PGI levels (*P* < 0.05). The gastric ulcer group, non-neoplastic polyp group, precancerous lesion group, EGC group, and AGC group had higher PGII and G-17 levels than the non-atrophic gastritis group. The expression levels of PGR in the atrophic gastritis group, non-neoplastic polyp group, precancerous lesion group, EGC group, and AGC group were significantly lower than those in the non-atrophic gastritis group (*P* < 0.05). The positive rate of Hp increased gradually in the non-atrophic gastritis group, non-neoplastic polyp group, atrophic gastritis group, EGC group, precancerous lesion group, gastric ulcer group, and AGC group (*P* < 0.05). Serum levels of PGI, PGII, PGR, G-17, and rates of HP infection were significantly different among groups (*P* < 0.05).

**Table 3:** Serum PG, G-17 and status of HP in each group

Group (n)	PGI (ng/ml)	PGII (ng/ml)	PGR	G-17 (mmol/l)	Hp (+) n (%)
Non atrophic gastritis group (655)	130.34 ± 68.33	13.95 ± 7.74	10.68 ± 5.39	5.53 (2.95, 10.56)	284 (43.36)
Atrophic gastritis group (52)	122.27 ± 52.77*	18.84 ± 11.13*	7.37 ± 2.78*	5.53 (4.24, 9.02)	26* (50.00)
Gastric ulcer group (109)	169.85 ± 84.51*	20.09 ± 11.50*	9.69 ± 4.85	5.96 (3.77, 11.25)*	71* (65.14)
Non-neoplastic polyp group (79)	132.37 ± 62.39	19.37 ± 11.58*	8.49 ± 5.62*	6.39 (4.53, 10.95)*	39 (49.37)
Precancerous lesion group (94)	109.70 ± 60.57*	18.08 ± 10.49*	6.95 ± 3.56*	6.65 (4.06, 13.02)*	58* (61.70)
Early cancer group (12)	98.61 ± 72.45*	17.26 ± 5.81*	6.13 ± 4.11*	5.89 (4.96, 13.24)*	7* (58.33)
Advanced cancer group (15)	66.30 ± 29.97*	19.70 ± 8.84*	4.54 ± 4.95*	10.6 (4.83, 16.26)*	10* (66.67)
*P*	< 0.001	< 0.001	< 0.001	0.003	< 0.001

*GC* gastric cancer, *Hp Helicobacter pylori*, *PGR* pepsinogen ratio, *G-17* gastrin-17

Compared with non-atrophic gastritis group, *P < 0.05

### ROC curves for diagnostic cutoffs of PG, G17, and Hp in GC

**Fig. 1:**
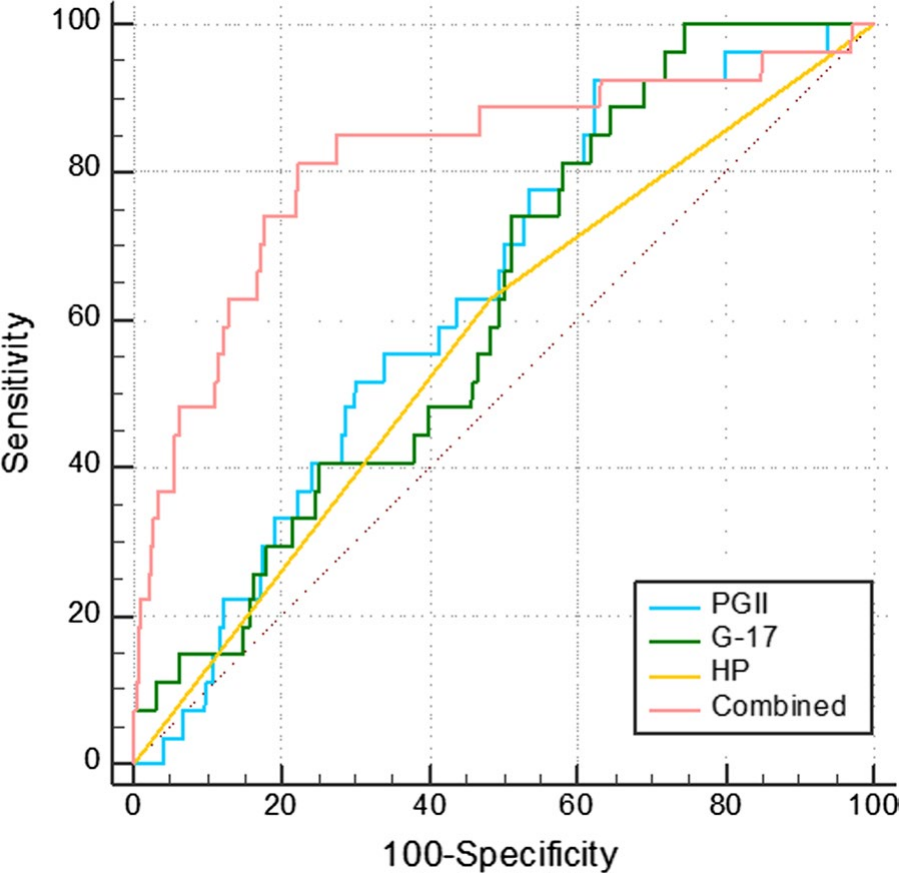
Receiver operating characteristic (ROC) curves for diagnosing gastric cancer (GC). ROC curve of combined is means the combination of PGI, PGII, PGR, G-17, and Hp for diagnosing GC

Patients with EGC and AGC were selected as the case group (n = 24) and those with non-atrophic gastritis, atrophic gastritis, gastric ulcer, non-neoplastic polyps, and precancerous lesions were selected as the contrast group (n = 995). As shown in Fig. 1 and Table 4, serum levels of PGI, PGII, PGR, G-17, and infection with Hp had diagnostic value for GC, among which the area under the ROC curve (AUC) of PGII, G-17, and Hp was higher than for the other two serum indicators (PGI and PGR). For PGII, the AUC was 0.636 (95% confidence interval [CI] 0.545–0.726), and the sensitivity and specificity of PGII in diagnosing GC were 92.6% and 37.5%, respectively. For G-17, the AUC was 0.621 (95% CI 0.533–0.710); the sensitivity and specificity of PGII in diagnosing GC were 100% and 25.4%, respectively. For Hp, the AUC was 0.573 (95% CI 0.466–0.681), and the sensitivity and specificity of Hp in diagnosing GC were 63% and 51.7%, respectively. The sensitivity, specificity, and AUC of the combination of PGI, PGII, PGR, G-17, and Hp in diagnosing GC were 81.5%, 77.8%, and 0.817 (95% CI 0.721–0.913), respectively.

**Table 4:** The accuracy of different serum gastric markers in diagnosing GC

Serum maker	AUC	95% CI	*P*
PGI	0.243	0.150–0.336	< 0.001
PGII	0.636	0.545–0.726	0.016
PGR	0.196	0.100–0.292	< 0.001
G-17	0.621	0.533–0.710	0.031
Hp	0.573	0.466–0.681	0.019
Combined	0.817	0.721–0.913	< 0.001

*GC* gastric cancer, *AUC* area under the receiver operating characteristic curve, *PG* pepsinogen, *PGR* pepsinogen ratio, *G-17* gastrin-17, *HP Helicobacter pylori*

#### Screening efficiency of the new scoring system for GC in the Wannan region

In our study, in patients with a medium–high risk of GC, the overall detection rate of GC was 2.36% (24/1019). There were 648, 919 and 100 patients in the low-, medium-, and high-risk of GC groups, respectively. The detection rate of GC in the low-, medium- and high-risk groups was 0% (0/648), 1.63% (15/919), and 9% (9/100), respectively (*P* < 0.001).

### Risk factors of GC in the Wannan region

We carried out univariate analysis to explore the risk factors for GC in the investigated population. The results suggested that there were no statistically significant differences between the two groups in terms of BMI; family history of GC; a history of drinking; and frequently consuming smoked, barbecued and overnight leftover foods (*P* > 0.05). GC and precancerous lesions were more frequently found in patients with age ≥ 60 years old, male sex, Hp infection, and a history of smoking (*P* < 0.05). Patients who ate a high-salt diet, and those who frequently consumed pickled and fried foods were more likely to have GC and precancerous lesions (*P* < 0.05); however, patients who frequently consumed green vegetables and fresh fruits were less likely to have GC and precancerous lesions (*P* < 0.05) (Table 5).

**Table 5:** Univariate analysis and multivariate analysis of risk factors for GC and precancerous lesions in the Wannan region

Risk factors	GC and precancerouslesions (n = 109)	Non-GC or precancerous lesions (n = 910)	OR	95% CI	*P*
Age					
< 60	40	654	4.440	2.904–6.666	< 0.001
≥ 60	69	256			
Sex					
Female	9	219	3.345	1.662–6.732	0.001
Male	100	691			
BMI					
< 18	27	84	0.474	0.673–1.202	0.900
18–25	31	506			
> 25	51	320			
Hp					
Negative	39	454	1.787	1.183–2.700	0.006
Positive	70	456			
Family history of GC					
No	87	793	1.714	1.033–2.844	0.037
Yes	22	117			
A history of smoking					
No	34	403	1.753	1.145–2.684	0.01
Yes	75	507			
A history of drinking					
No	80	687	1.117	0.711–1.753	0.631
Yes	29	223			
A high-salt diet					
No	47	581	2.330	1.588–3.484	< 0.001
Yes	62	329			
Frequently eating pickled foods					
No	69	753	2.780	1.816–4.256	< 0.001
Yes	40	157			
Frequently eating fried foods					
No	86	816	2.322	1.398–3.855	0.001
Yes	23	94			
Frequently eating smoked foods					
No	98	838	1.306	0.670–2.548	0.433
Yes	11	72			
Frequently eating barbecue foods					
No	102	847	1.065	0.496–2.286	0.872
Yes	7	63			
Frequently eating overnight foods					
No	76	564	0.708	0.460–1.088	0.115
Yes	33	346			
Frequent consumption of green vegetables					
No	46	209	0.408	0.271–0.615	< 0.001
Yes	63	701			
Frequent consumption of fresh fruits					
No	63	382	0.528	0.353–0.790	0.002
Yes	46	528			

Variable	*P* value	OR	95% CI
Multivariate analysis of risk factors for GC and precancerous lesions			
Age	< 0.001	5.934	3.695–9.529
Sex	< 0.001	5.721	2.579–12.695
Hp	0.003	1.992	1.255–3.163
A history of smoking	0.007	2.028	1.213–3.392
A high salt diet	< 0.001	2.877	1.807–4.580
Frequently eating pickled foods	0.016	1.873	1.125–3.120
Frequently eating fried foods	0.002	2.459	1.384–4.369
Frequent consumption of green vegetables	< 0.001	0.388	0.242–0.620

*GC* gastric cancer, *BMI* body mass index, *Hp Helicobacter pylori*, *OR* odds ratio, *CI* confidence interval

### Multivariate analysis of risk factors for GC in the Wannan region

We conducted a multivariate analysis of the risk factors for GC in the Wannan region. The results showed that age (odds ratio [OR], 5.934; 95% CI 3.695–9.529; *P* < 0.001), sex (OR 5.721; 95% CI 2.579–12.695; *P* < 0.001), Hp infection (OR 1.992; 95% CI 1.255–3.163; *P* = 0.003), a history of smoking (OR 2.028; 95% CI 1.213–3.392; *P* = 0.007), consuming a high-salt diet (OR 2.877; 95% CI 1.807–4.580; *P* < 0.001), frequent consumption of pickled foods (OR 1.873; 95% CI 1.125–3.120; *P* = 0.016) and frequent consumption of fried foods (OR 2.459; 95% CI 1.384–4.369; *P* = 0.002) were independent risk factors for GC and precancerous lesions (*P* < 0.05). However, frequent consumption of green vegetables (OR 0.388; 95% CI 0.242–0.620; *P* < 0.001) was an independent protective factor against GC and precancerous lesions (Table 5).

## Discussion

The aim of this study was to test the feasibility of a new scoring system for GC screening in the Wannan region. The results of our study showed that PGII, G-17, and Hp had relatively high accuracy in predicting the risk of GC. The AUC of the combination of PGI, PGII, PGR, G-17, and Hp in diagnosing GC was 0.817, which suggested that combined measurement of serum PGI, PGII, PGR, G-17, and Hp can be used as an effective non-invasive indicators in screening individuals for GC.

Our results showed that patients with GC had low serum PGI and PGR levels but high PGII and G17 levels; serum PGII and G17 levels showed good sensitivity but low specificity for diagnosing GC. The sensitivity of the combination of PGI, PGII, PGR, G-17, and Hp for diagnosing GC was 81.50%, which was lower than those using PGII alone and G-17 alone; however, the specificity (77.8%) was higher. Thus, the combination of serum indicators and status of Hp infection can raise the diagnostic accuracy of GC for screening in the Wannan region. In future clinical work, when combined measurements yield positive results, it is necessary to screen patients carefully using gastroscopy for early diagnosis and treatment. However, one study in Taizhou, China [21] suggested that the AUCs of PGI, PGR, and G-17 were 0.728, 0.726, and 0.556, respectively, which was not consistent with our results. This might be attributed to regional differences.

Our results also showed that the detection rates of GC in the low-, medium-, and high-GC risk groups were 0%, 1,63%, and 9%, respectively (*P* < 0.001), which suggested that this new scoring system for GC screening is more valuable and feasible in individuals with a high risk of GC.

In our research, a total of 25,194 asymptomatic participants were randomly screened, and 8009 patients with a medium–high risk of GC were identified, among which 1019 underwent gastroscopy; the response rate for gastroscopy in our patients (12.72%) was much lower than the average rate in China (26.07%) [22]. This might be attributed to the fact that endoscopy is an invasive procedure and the current screened population lacked awareness about the concept of cancer prevention and treatment. Socioeconomic and education levels in this population were also relatively lower than the national levels. Thus, greater attention is needed to raising awareness of GC prevention to improve the rate of gastroscopy in the population of Wannan region.

Our research revealed that age ≥ 60 years old, male sex, and Hp infection were independent risk factors for GC and precancerous lesions. The morbidity of GC increases with age, especially after 40 years old [23, 24]. Compared with women, men have a higher risk of GC. A retrospective analysis based on a study population of 250,000 in China showed [25] that the sex ratio of patients with GC was 3:1; however, the sex ratio in our study was 8.73:1, which was significantly higher than that past reported. This indicates that the morbidity of GC is more frequent among men than among women in the Wannan region. Chen et al. [26] reported that individuals with overweight (BMI 23.9–27.9 kg/m^2^) and obesity (BMI > 28 kg/m^2^) are at risk for cancer of the gastric cardia. However, in our study, we failed to find a relationship between BMI and GC. Further research is required to explore the association between BMI and GC.

Currently, greater attention is being paid to the lifestyle habits of patients with GC. In our study, we found that a history of smoking, high-salt diet, and frequent consumption of fried foods were independent risk factors for GC and precancerous lesions whereas frequently eating green vegetables was an independent protective factor for GC. Other studies [27, 28] have also reported that smoking is an independent risk factor for GC. This might be explained by the fact that smoking stimulates gastric acid secretion and vasoconstriction of gastric mucosa, which reduces the synthesis of protective barrier of the gastric mucosa, such as prostaglandin factor, leading to aggravation of gastric mucosa injury. Previous reports [29, 30] have confirmed that a high-salt diet increases the risk of GC. Cai et al. [11] found that frequently eating fried and pickled foods were risk factors for GC. In our study, we also found frequent fried food consumption was an independent risk factor for GC and precancerous lesions, but frequently eating pickled foods was not. Lunet et al. [31] showed that fruit and vegetable intake could reduce the risk of GC. However, Wang et al. [32] suggested that fruit intake was a protective factor against GC whereas vegetable intake was not a protective factor for GC. Another national multicenter study found that eating green vegetables and fresh fruits frequently were not independently associated with the risk of GC [11]. In our study, frequent consumption of green vegetables was an independently protective factor against GC and precancerous lesions, whereas frequently eating fresh fruits was not. The relationship between GC and frequent consumption of green vegetables and fresh fruits remains controversial. Large-scale clinical trials are still needed to further explore this correlation.

Some limitations of this analysis should be acknowledged. Firstly, the sample size of our study was small; large-scale, multicenter clinical trials are required. Secondly, serum levels of PG and G-17 were related to anatomical location, and the location of lesions was not further analyzed in this study. Thirdly, serum PG and G-17 levels might differ according to regions, ethnicity, diet, environment, and genetics. It is necessary to explore the optimal critical values of serum PG and G-17 for GC screening in the Wannan region. Fourthly, levels of PG and G-17 are affected by PPIs far more often than are underlying diseases. Although we excluded patients who had used antacid within the past 2 weeks, the potential effect of PPI use status before enrollment is unknown. Fifthly, the classification of the enrolled patients in this study was based on the main diagnosis, so there was some overlap between some groups. For example, 19 patients with gastric ulcer accompanied by non-atrophic gastritis were classified into gastric ulcer group in our study. Lastly, because the detection cost of tumor markers is relatively expensive in large-scale population screening, tumor markers were not included; the hazard ratio of tumor markers should be included in future studies.

## Conclusion

Our results suggested that a combination of PGI, PGII, PGR, G-17, and Hp can be used as valid markers in screening for GC prior to gastroscopy in the Wannan region. The new scoring system for GC screening was valuable and feasible in Wannan region, especially in the high-risk population. Use of this scoring system could lead to timely treatment and a consequent improvement in patients’ quality of life. Age ≥ 60, male sex, Hp infection, a history of smoking, consuming a high-salt diet and frequent consumption of fried foods were independent risk factors for GC and precancerous lesions whereas frequently eating green vegetables was an independent protective factor against GC and precancerous lesions. Therefore, habitual consumption of green vegetables may be an effective preventive measure against the development of GC.

## Supplementary Information

**Additional file 1.** Primary data of the enrolled patients.

## Data Availability

The datasets in the current study are available from the corresponding author. We also provide it as a Additional file 1.

## Abbreviations

GC: Gastric cancer
EGC: Early gastric cancer
AGC: Advanced gastric cancer
ESD: Endoscopic submucosal dissection
PGR: Pepsinogen ratio
G-17: Gastrin-17
Hp: *Helicobacter pylori*
ROC: Receiver operating characteristic
PG: Pepsinogen
CI: Confidence interval
WHO: World Health Organization
UBT: Urea breath test
ELISA: Enzyme-linked immunosorbent assay
WLE: White light endoscopy
NBI: Narrowband imaging endoscopy
ME: Magnification endoscopy
LGIN: Low-grade intraepithelial neoplasia
HGIN: High-grade intraepithelial neoplasia
BMI: Body mass index
ANOVA: One-way analysis of variance
SD: Standard deviation
