# Supplementary material for: Clinical applicability of a new scoring system for population-based screening and risk factors of gastric cancer in the Wannan region
Source: BMC Gastroenterol. 2022 Jun 23;22:306. doi: 10.1186/s12876-022-02384-w (PMC9219187; doi:10.1186/s12876-022-02384-w)
Supplement: Supplementary file 1 — Additional file 1. Primary data of the enrolled patients. [file 12876_2022_2384_MOESM1_ESM.docx]

Supplementary table 1: Primary data of enrolled 8009 cases with medium-high risk of GC in this study.

| ID | sex（male/female） | age | Hp(negative/positive ) | PGI | PGII | G-17 | PGI/PGII | gastroscopy | score | result of gastroscopy | histological examinations |
| --- | --- | --- | --- | --- | --- | --- | --- | --- | --- | --- | --- |
| 1 | Male | 81 | negative | 105.09 | 12.76 | 0.64 | 8.24 | negative | 14 |  |  |
| 2 | Female | 80 | positive | 181.12 | 22.73 | 11.24 | 7.97 | negative | 16 |  |  |
| 3 | Male | 79 | positive | 187.05 | 18.82 | 11.62 | 9.94 | negative | 20 |  |  |
| 4 | Female | 79 | positive | 75.14 | 15.72 | 4.36 | 4.78 | negative | 14 |  |  |
| 5 | female | 78 | negative | 68.95 | 4.78 | 2.32 | 14.42 | negative | 13 |  |  |
| 6 | Male | 78 | negative | 27.97 | 8.23 | 45.21 | 3.40 | negative | 22 |  |  |
| 7 | male | 78 | negative | 66.26 | 13.20 | 2.03 | 5.02 | negative | 17 |  |  |
| 8 | male | 78 | positive | 147.90 | 21.79 | 3.37 | 6.79 | negative | 18 |  |  |
| 9 | female | 76 | negative | 103.80 | 7.59 | 3.61 | 13.68 | negative | 13 |  |  |
| 10 | male | 82 | negative | 174.18 | 13.25 | 0.58 | 13.15 | negative | 14 |  |  |
| 11 | female | 78 | negative | 114.89 | 5.16 | 3.19 | 22.27 | negative | 13 |  |  |
| 12 | male | 79 | positive | 27.51 | 9.54 | 2.12 | 2.88 | negative | 21 |  |  |
| 13 | female | 82 | positive | 220.16 | 38.44 | 13.83 | 5.73 | negative | 16 |  |  |
| 14 | female | 77 | negative | 86.66 | 5.17 | 3.18 | 16.76 | negative | 13 |  |  |
| 15 | female | 76 | negative | 128.78 | 3.72 | 3.41 | 34.62 | negative | 13 |  |  |
| 16 | male | 79 | negative | 87.54 | 7.43 | 1.11 | 11.78 | negative | 14 |  |  |
| 17 | male | 79 | negative | 300.00 | 23.04 | 1.05 | 13.02 | negative | 14 |  |  |
| 18 | female | 76 | positive | 103.87 | 18.49 | 5.14 | 5.62 | negative | 14 |  |  |
| 19 | male | 77 | negative | 26.57 | 10.13 | 60.00 | 2.62 | negative | 22 |  |  |
| 20 | female | 76 | positive | 287.35 | 23.20 | 8.59 | 12.39 | negative | 16 |  |  |
| 21 | male | 78 | negative | 187.12 | 13.04 | 22.58 | 14.35 | negative | 19 |  |  |
| 22 | male | 79 | negative | 65.82 | 7.09 | 2.64 | 9.28 | negative | 17 |  |  |
| 23 | male | 78 | negative | 146.28 | 8.70 | 1.80 | 16.81 | negative | 17 |  |  |
| 24 | female | 79 | negative | 104.47 | 9.88 | 12.91 | 10.57 | negative | 15 |  |  |
| 25 | female | 79 | negative | 113.40 | 8.86 | 4.20 | 12.80 | negative | 13 |  |  |
| 26 | male | 81 | negative | 126.05 | 5.84 | 3.04 | 21.58 | negative | 17 |  |  |
| 27 | male | 80 | negative | 155.51 | 6.59 | 2.04 | 23.60 | negative | 17 |  |  |
| 28 | female | 77 | negative | 300.00 | 26.42 | 20.40 | 11.36 | negative | 15 |  |  |
| 29 | male | 77 | negative | 119.42 | 8.85 | 2.29 | 13.49 | negative | 17 |  |  |
| 30 | male | 79 | negative | 200.21 | 13.68 | 3.93 | 14.64 | negative | 17 |  |  |
| 31 | male | 78 | negative | 132.18 | 9.78 | 9.10 | 13.52 | negative | 19 |  |  |
| 32 | male | 77 | negative | 222.20 | 16.50 | 10.60 | 13.47 | negative | 19 |  |  |
| 33 | female | 79 | negative | 110.40 | 14.60 | 3.30 | 7.56 | negative | 13 |  |  |
| 34 | female | 77 | positive | 147.15 | 10.89 | 9.16 | 13.51 | negative | 16 |  |  |
| 35 | female | 78 | negative | 72.37 | 5.30 | 4.66 | 13.65 | negative | 13 |  |  |
| 36 | female | 77 | positive | 97.23 | 5.80 | 2.78 | 16.76 | negative | 14 |  |  |
| 37 | female | 76 | negative | 182.88 | 17.52 | 5.83 | 10.44 | negative | 15 |  |  |
| 38 | female | 78 | negative | 102.07 | 6.97 | 4.25 | 14.64 | negative | 13 |  |  |
| 39 | female | 76 | positive | 201.90 | 29.50 | 6.20 | 6.84 | negative | 16 |  |  |
| 40 | male | 77 | negative | 285.62 | 19.53 | 9.46 | 14.62 | negative | 19 |  |  |
| 41 | male | 77 | negative | 175.71 | 11.23 | 1.41 | 15.65 | negative | 14 |  |  |
| 42 | male | 80 | positive | 204.68 | 30.55 | 4.96 | 6.70 | negative | 18 |  |  |
| 43 | female | 79 | negative | 123.39 | 10.83 | 3.11 | 11.39 | negative | 13 |  |  |
| 44 | male | 78 | positive | 172.44 | 17.63 | 5.39 | 9.78 | negative | 18 |  |  |
| 45 | male | 81 | negative | 65.36 | 9.56 | 12.76 | 6.84 | negative | 19 |  |  |
| 46 | male | 89 | negative | 45.60 | 13.40 | 29.30 | 3.40 | negative | 22 |  |  |
| 47 | male | 82 | negative | 180.07 | 11.97 | 1.93 | 15.04 | negative | 17 |  |  |
| 48 | male | 77 | negative | 45.14 | 6.53 | 0.51 | 6.91 | negative | 14 |  |  |
| 49 | male | 77 | negative | 139.80 | 9.32 | 4.03 | 15.00 | negative | 17 |  |  |
| 50 | male | 89 | negative | 254.71 | 12.75 | 1.27 | 19.98 | negative | 14 |  |  |
| 51 | male | 77 | positive | 145.49 | 16.21 | 6.63 | 8.98 | negative | 20 |  |  |
| 52 | male | 77 | negative | 28.39 | 11.55 | 1.78 | 2.46 | negative | 20 |  |  |
| 53 | male | 76 | negative | 43.03 | 15.79 | 43.78 | 2.73 | negative | 22 |  |  |
| 54 | female | 76 | negative | 118.82 | 10.17 | 4.66 | 11.68 | negative | 13 |  |  |
| 55 | male | 82 | positive | 48.21 | 8.36 | 1.40 | 5.77 | negative | 15 |  |  |
| 56 | male | 79 | negative | 95.30 | 6.74 | 2.99 | 14.14 | negative | 17 |  |  |
| 57 | male | 79 | positive | 150.19 | 17.51 | 5.97 | 8.58 | negative | 20 |  |  |
| 58 | male | 78 | positive | 159.49 | 10.82 | 1.03 | 14.74 | negative | 15 |  |  |
| 59 | male | 79 | negative | 99.50 | 10.30 | 2.83 | 9.66 | negative | 17 |  |  |
| 60 | male | 81 | negative | 88.83 | 9.22 | 8.01 | 9.63 | negative | 19 |  |  |
| 61 | female | 79 | negative | 205.50 | 16.70 | 32.60 | 12.31 | negative | 15 |  |  |
| 62 | male | 79 | negative | 232.90 | 10.26 | 1.92 | 22.70 | negative | 17 |  |  |
| 63 | female | 76 | negative | 207.38 | 13.39 | 5.29 | 15.49 | negative | 13 |  |  |
| 64 | male | 85 | negative | 98.68 | 6.37 | 2.58 | 15.49 | negative | 17 |  |  |
| 65 | female | 77 | negative | 71.01 | 6.27 | 4.83 | 11.33 | negative | 13 |  |  |
| 66 | male | 80 | negative | 100.50 | 10.63 | 5.81 | 9.45 | negative | 19 |  |  |
| 67 | male | 77 | negative | 137.35 | 8.86 | 2.86 | 15.50 | negative | 17 |  |  |
| 68 | female | 76 | negative | 148.00 | 7.40 | 7.88 | 20.00 | negative | 15 |  |  |
| 69 | male | 77 | negative | 56.90 | 3.98 | 0.72 | 14.30 | negative | 14 |  |  |
| 70 | male | 77 | negative | 214.21 | 12.93 | 2.38 | 16.57 | negative | 17 |  |  |
| 71 | male | 76 | positive | 121.28 | 33.46 | 22.53 | 3.62 | negative | 23 |  |  |
| 72 | male | 79 | positive | 188.55 | 18.69 | 6.24 | 10.09 | negative | 20 |  |  |
| 73 | male | 77 | negative | 49.21 | 8.80 | 3.08 | 5.59 | negative | 17 |  |  |
| 74 | female | 80 | negative | 36.28 | 3.68 | 1.55 | 9.86 | negative | 13 |  |  |
| 75 | female | 79 | negative | 171.55 | 6.35 | 2.50 | 27.02 | negative | 13 |  |  |
| 76 | male | 79 | negative | 127.27 | 8.80 | 1.95 | 14.46 | negative | 17 |  |  |
| 77 | female | 82 | negative | 140.30 | 13.82 | 7.79 | 10.15 | negative | 15 |  |  |
| 78 | male | 78 | negative | 192.70 | 10.89 | 1.74 | 17.70 | negative | 17 |  |  |
| 79 | male | 77 | positive | 92.04 | 11.33 | 3.35 | 8.12 | negative | 18 |  |  |
| 80 | male | 77 | negative | 74.90 | 6.60 | 3.40 | 11.35 | negative | 17 |  |  |
| 81 | male | 77 | negative | 52.34 | 4.87 | 1.54 | 10.75 | negative | 17 |  |  |
| 82 | female | 77 | negative | 69.91 | 5.77 | 1.74 | 12.12 | negative | 13 |  |  |
| 83 | male | 83 | negative | 108.28 | 22.68 | 30.12 | 4.77 | negative | 19 |  |  |
| 84 | male | 77 | positive | 147.65 | 10.41 | 4.26 | 14.18 | negative | 18 |  |  |
| 85 | female | 77 | negative | 93.83 | 4.49 | 9.47 | 20.90 | negative | 15 |  |  |
| 86 | male | 82 | positive | 97.11 | 14.16 | 3.10 | 6.86 | negative | 18 |  |  |
| 87 | male | 78 | negative | 96.15 | 8.37 | 4.95 | 11.49 | negative | 17 |  |  |
| 88 | female | 84 | negative | 40.03 | 22.41 | 11.10 | 1.79 | negative | 18 |  |  |
| 89 | male | 76 | negative | 157.30 | 23.00 | 3.70 | 6.84 | negative | 17 |  |  |
| 90 | male | 78 | negative | 82.43 | 6.49 | 2.79 | 12.70 | negative | 17 |  |  |
| 91 | male | 77 | negative | 136.60 | 7.73 | 0.82 | 17.67 | negative | 14 |  |  |
| 92 | male | 85 | positive | 267.50 | 35.33 | 8.11 | 7.57 | negative | 20 |  |  |
| 93 | male | 76 | positive | 91.34 | 11.84 | 4.04 | 7.71 | negative | 18 |  |  |
| 94 | male | 78 | negative | 192.94 | 21.52 | 2.12 | 8.97 | negative | 17 |  |  |
| 95 | male | 78 | negative | 97.34 | 12.69 | 2.56 | 7.67 | negative | 17 |  |  |
| 96 | male | 80 | negative | 144.55 | 7.52 | 1.92 | 19.22 | negative | 17 |  |  |
| 97 | female | 78 | positive | 129.14 | 19.09 | 11.28 | 6.76 | negative | 16 |  |  |
| 98 | male | 80 | negative | 144.29 | 6.95 | 2.47 | 20.76 | negative | 17 |  |  |
| 99 | male | 77 | negative | 282.65 | 22.81 | 5.69 | 12.39 | negative | 17 |  |  |
| 100 | male | 76 | negative | 101.97 | 8.99 | 5.12 | 11.34 | negative | 17 |  |  |
| 101 | female | 80 | negative | 162.56 | 17.41 | 3.31 | 9.34 | negative | 13 |  |  |
| 102 | male | 81 | negative | 90.22 | 8.91 | 5.19 | 10.13 | negative | 17 |  |  |
| 103 | male | 80 | negative | 80.77 | 4.28 | 1.98 | 18.87 | negative | 17 |  |  |
| 104 | male | 78 | negative | 132.74 | 11.86 | 2.27 | 11.19 | negative | 17 |  |  |
| 105 | male | 77 | positive | 151.21 | 18.15 | 11.10 | 8.33 | negative | 20 |  |  |
| 106 | male | 81 | negative | 69.50 | 17.10 | 3.90 | 4.06 | negative | 17 |  |  |
| 107 | male | 77 | negative | 76.88 | 6.92 | 1.46 | 11.11 | negative | 14 |  |  |
| 108 | male | 77 | positive | 68.02 | 14.94 | 4.56 | 4.55 | negative | 18 |  |  |
| 109 | male | 77 | negative | 93.46 | 7.31 | 2.14 | 12.79 | negative | 17 |  |  |
| 110 | male | 84 | negative | 163.83 | 9.72 | 1.93 | 16.85 | negative | 17 |  |  |
| 111 | female | 80 | negative | 89.86 | 4.50 | 2.26 | 19.97 | negative | 13 |  |  |
| 112 | female | 84 | negative | 162.52 | 13.70 | 2.39 | 11.86 | negative | 13 |  |  |
| 113 | male | 76 | positive | 55.23 | 19.21 | 60.00 | 2.88 | negative | 23 |  |  |
| 114 | male | 76 | negative | 61.78 | 10.42 | 3.76 | 5.93 | negative | 17 |  |  |
| 115 | male | 79 | negative | 173.94 | 25.71 | 13.17 | 6.77 | negative | 19 |  |  |
| 116 | female | 81 | positive | 300.00 | 37.99 | 5.00 | 7.90 | negative | 14 |  |  |
| 117 | male | 78 | negative | 82.40 | 9.00 | 6.80 | 9.16 | negative | 19 |  |  |
| 118 | female | 76 | negative | 92.60 | 4.79 | 3.91 | 19.33 | negative | 13 |  |  |
| 119 | female | 76 | negative | 255.53 | 46.56 | 41.72 | 5.49 | negative | 15 |  |  |
| 120 | male | 81 | positive | 145.82 | 23.99 | 3.09 | 6.08 | negative | 18 |  |  |
| 121 | female | 87 | negative | 106.75 | 5.60 | 2.19 | 19.06 | negative | 13 |  |  |
| 122 | male | 78 | negative | 95.93 | 9.03 | 2.49 | 10.62 | negative | 17 |  |  |
| 123 | male | 80 | negative | 111.81 | 8.37 | 2.34 | 13.36 | negative | 17 |  |  |
| 124 | male | 80 | positive | 75.10 | 14.70 | 3.90 | 5.11 | negative | 18 |  |  |
| 125 | male | 81 | negative | 19.53 | 3.96 | 59.87 | 4.93 | negative | 19 |  |  |
| 126 | female | 78 | negative | 172.32 | 10.87 | 2.98 | 15.85 | negative | 13 |  |  |
| 127 | female | 75 | positive | 151.86 | 21.27 | 2.72 | 7.14 | negative | 14 |  |  |
| 128 | female | 73 | negative | 147.78 | 9.26 | 5.40 | 15.96 | negative | 13 |  |  |
| 129 | female | 74 | negative | 84.80 | 8.60 | 2.34 | 9.86 | negative | 13 |  |  |
| 130 | male | 75 | negative | 134.42 | 15.19 | 1.97 | 8.85 | negative | 17 |  |  |
| 131 | female | 75 | negative | 54.50 | 2.85 | 18.26 | 19.12 | negative | 15 |  |  |
| 132 | male | 76 | negative | 52.00 | 3.17 | 2.20 | 16.40 | negative | 17 |  |  |
| 133 | female | 74 | negative | 39.46 | 8.13 | 23.79 | 4.85 | negative | 15 |  |  |
| 134 | male | 76 | negative | 60.33 | 7.21 | 2.69 | 8.37 | negative | 17 |  |  |
| 135 | female | 74 | positive | 86.70 | 22.90 | 20.70 | 3.79 | negative | 19 |  |  |
| 136 | female | 75 | negative | 107.88 | 10.85 | 5.48 | 9.94 | negative | 13 |  |  |
| 137 | female | 74 | negative | 77.55 | 12.34 | 4.38 | 6.28 | negative | 13 |  |  |
| 138 | female | 74 | negative | 68.57 | 7.33 | 2.14 | 9.35 | negative | 13 |  |  |
| 139 | male | 76 | negative | 90.79 | 5.79 | 2.14 | 15.68 | negative | 17 |  |  |
| 140 | female | 75 | positive | 257.49 | 36.58 | 20.77 | 7.04 | negative | 16 |  |  |
| 141 | male | 75 | negative | 125.97 | 12.08 | 6.94 | 10.43 | negative | 19 |  |  |
| 142 | male | 75 | negative | 172.25 | 8.94 | 1.86 | 19.27 | negative | 17 |  |  |
| 143 | male | 74 | positive | 128.44 | 19.64 | 3.45 | 6.54 | negative | 18 |  |  |
| 144 | male | 74 | positive | 127.04 | 26.60 | 41.65 | 4.78 | negative | 20 |  |  |
| 145 | female | 74 | negative | 72.26 | 4.33 | 4.81 | 16.69 | negative | 13 |  |  |
| 146 | male | 74 | negative | 89.75 | 5.17 | 1.87 | 17.36 | negative | 17 |  |  |
| 147 | male | 75 | negative | 90.75 | 25.69 | 4.35 | 3.53 | negative | 20 |  |  |
| 148 | male | 74 | negative | 102.63 | 9.81 | 2.82 | 10.46 | negative | 17 |  |  |
| 149 | male | 73 | negative | 178.18 | 14.16 | 7.66 | 12.58 | negative | 19 |  |  |
| 150 | female | 75 | positive | 249.50 | 27.70 | 10.70 | 9.01 | negative | 16 |  |  |
| 151 | female | 74 | negative | 163.65 | 6.74 | 2.53 | 24.28 | negative | 13 |  |  |
| 152 | male | 74 | positive | 279.55 | 29.59 | 3.77 | 9.45 | negative | 18 |  |  |
| 153 | female | 73 | positive | 203.50 | 32.80 | 7.80 | 6.20 | negative | 16 |  |  |
| 154 | female | 73 | negative | 72.39 | 7.02 | 6.19 | 10.31 | negative | 15 |  |  |
| 155 | female | 74 | positive | 86.18 | 19.19 | 5.88 | 4.49 | negative | 16 |  |  |
| 156 | male | 75 | negative | 107.21 | 6.01 | 3.33 | 17.84 | negative | 17 |  |  |
| 157 | male | 74 | negative | 300.00 | 26.48 | 9.52 | 11.33 | negative | 19 |  |  |
| 158 | female | 74 | negative | 93.18 | 7.82 | 1.85 | 11.92 | negative | 13 |  |  |
| 159 | female | 75 | negative | 51.06 | 5.01 | 14.76 | 10.19 | negative | 15 |  |  |
| 160 | female | 74 | negative | 120.24 | 7.06 | 3.39 | 17.03 | negative | 13 |  |  |
| 161 | male | 74 | negative | 55.11 | 5.40 | 1.84 | 10.21 | negative | 17 |  |  |
| 162 | male | 75 | positive | 124.72 | 15.11 | 5.68 | 8.25 | negative | 18 |  |  |
| 163 | male | 74 | negative | 146.12 | 11.45 | 1.50 | 12.76 | negative | 17 |  |  |
| 164 | male | 74 | negative | 53.84 | 7.23 | 2.01 | 7.45 | negative | 17 |  |  |
| 165 | female | 74 | negative | 53.40 | 5.79 | 9.20 | 9.22 | negative | 15 |  |  |
| 166 | male | 74 | positive | 83.69 | 15.88 | 17.82 | 5.27 | negative | 20 |  |  |
| 167 | male | 76 | negative | 72.80 | 5.30 | 2.10 | 13.74 | negative | 17 |  |  |
| 168 | male | 74 | negative | 199.80 | 18.00 | 1.50 | 11.10 | negative | 17 |  |  |
| 169 | female | 73 | positive | 85.48 | 15.37 | 75.14 | 5.56 | negative | 16 |  |  |
| 170 | female | 74 | negative | 300.00 | 31.75 | 16.01 | 9.45 | negative | 15 |  |  |
| 171 | female | 75 | negative | 92.04 | 8.33 | 2.61 | 11.05 | negative | 13 |  |  |
| 172 | female | 74 | positive | 177.04 | 26.96 | 5.42 | 6.57 | negative | 14 |  |  |
| 173 | male | 75 | negative | 100.56 | 14.73 | 4.43 | 6.83 | negative | 17 |  |  |
| 174 | female | 74 | negative | 295.35 | 18.24 | 9.60 | 16.19 | negative | 15 |  |  |
| 175 | male | 74 | negative | 205.46 | 16.21 | 4.31 | 12.67 | negative | 17 |  |  |
| 176 | male | 75 | negative | 93.44 | 5.47 | 2.23 | 17.08 | negative | 17 |  |  |
| 177 | female | 75 | positive | 124.73 | 19.84 | 3.69 | 6.29 | negative | 14 |  |  |
| 178 | female | 74 | negative | 92.80 | 14.27 | 3.40 | 6.50 | negative | 13 |  |  |
| 179 | female | 76 | positive | 115.09 | 12.01 | 11.96 | 9.58 | negative | 16 |  |  |
| 180 | male | 74 | negative | 78.30 | 10.57 | 17.79 | 7.41 | negative | 19 |  |  |
| 181 | female | 74 | negative | 203.45 | 23.14 | 3.64 | 8.79 | negative | 13 |  |  |
| 182 | male | 75 | negative | 119.03 | 6.91 | 0.68 | 17.23 | negative | 14 |  |  |
| 183 | female | 75 | negative | 110.61 | 5.72 | 1.78 | 19.34 | negative | 13 |  |  |
| 184 | female | 75 | negative | 133.27 | 8.43 | 11.92 | 15.81 | negative | 15 |  |  |
| 185 | male | 74 | negative | 101.28 | 6.01 | 2.53 | 16.85 | negative | 17 |  |  |
| 186 | male | 76 | negative | 176.92 | 11.63 | 0.64 | 15.21 | negative | 14 |  |  |
| 187 | female | 75 | positive | 107.35 | 13.02 | 6.91 | 8.25 | negative | 16 |  |  |
| 188 | male | 75 | negative | 216.89 | 7.32 | 1.85 | 29.63 | negative | 17 |  |  |
| 189 | male | 73 | positive | 80.00 | 18.50 | 5.80 | 4.32 | negative | 20 |  |  |
| 190 | male | 75 | positive | 67.01 | 10.72 | 3.10 | 6.25 | negative | 18 |  |  |
| 191 | female | 75 | negative | 149.94 | 14.68 | 3.03 | 10.21 | negative | 13 |  |  |
| 192 | male | 75 | negative | 65.80 | 4.47 | 3.06 | 14.72 | negative | 17 |  |  |
| 193 | male | 74 | positive | 95.96 | 23.46 | 6.44 | 4.09 | negative | 20 |  |  |
| 194 | male | 73 | negative | 148.53 | 18.04 | 5.30 | 8.23 | negative | 17 |  |  |
| 195 | female | 74 | negative | 239.75 | 18.23 | 2.72 | 13.15 | negative | 13 |  |  |
| 196 | male | 75 | negative | 268.92 | 15.45 | 1.22 | 17.41 | negative | 14 |  |  |
| 197 | female | 74 | negative | 80.05 | 7.44 | 3.40 | 10.76 | negative | 13 |  |  |
| 198 | male | 75 | negative | 138.52 | 10.02 | 4.53 | 13.82 | negative | 17 |  |  |
| 199 | male | 73 | negative | 181.28 | 14.54 | 5.12 | 12.47 | negative | 17 |  |  |
| 200 | female | 75 | negative | 40.85 | 5.82 | 20.57 | 7.02 | negative | 15 |  |  |
| 201 | male | 75 | negative | 126.12 | 9.90 | 2.32 | 12.74 | negative | 17 |  |  |
| 202 | male | 75 | negative | 86.90 | 10.74 | 6.82 | 8.09 | negative | 19 |  |  |
| 203 | male | 74 | positive | 79.61 | 12.72 | 10.45 | 6.26 | negative | 20 |  |  |
| 204 | female | 75 | negative | 75.74 | 6.72 | 3.91 | 11.27 | negative | 13 |  |  |
| 205 | male | 75 | negative | 118.33 | 13.81 | 5.00 | 8.57 | negative | 17 |  |  |
| 206 | female | 75 | negative | 72.13 | 3.81 | 5.02 | 18.93 | negative | 13 |  |  |
| 207 | male | 76 | negative | 108.60 | 8.52 | 1.00 | 12.75 | negative | 14 |  |  |
| 208 | male | 74 | positive | 144.06 | 10.21 | 2.25 | 14.11 | negative | 18 |  |  |
| 209 | female | 74 | negative | 89.21 | 5.26 | 3.31 | 16.96 | negative | 13 |  |  |
| 210 | male | 75 | positive | 101.97 | 17.02 | 7.05 | 5.99 | negative | 20 |  |  |
| 211 | male | 74 | negative | 32.19 | 17.02 | 31.72 | 1.89 | negative | 22 |  |  |
| 212 | female | 74 | positive | 90.18 | 6.47 | 3.03 | 13.94 | negative | 14 |  |  |
| 213 | male | 75 | negative | 162.88 | 11.66 | 2.34 | 13.97 | negative | 17 |  |  |
| 214 | male | 73 | positive | 165.03 | 19.21 | 8.38 | 8.59 | negative | 20 |  |  |
| 215 | male | 75 | negative | 141.25 | 10.60 | 1.05 | 13.33 | negative | 14 |  |  |
| 216 | female | 75 | negative | 79.37 | 6.78 | 3.00 | 11.71 | negative | 13 |  |  |
| 217 | male | 74 | positive | 75.66 | 13.12 | 1.36 | 5.77 | negative | 15 |  |  |
| 218 | male | 74 | negative | 100.65 | 8.31 | 4.02 | 12.11 | negative | 17 |  |  |
| 219 | male | 74 | positive | 160.27 | 10.94 | 6.16 | 14.65 | negative | 20 |  |  |
| 220 | male | 74 | negative | 60.02 | 4.49 | 2.17 | 13.37 | negative | 17 |  |  |
| 221 | male | 76 | positive | 90.10 | 12.21 | 10.50 | 7.38 | negative | 20 |  |  |
| 222 | male | 75 | negative | 91.87 | 5.35 | 0.64 | 17.17 | negative | 14 |  |  |
| 223 | female | 74 | negative | 86.63 | 6.14 | 1.70 | 14.11 | negative | 13 |  |  |
| 224 | male | 75 | negative | 124.47 | 8.08 | 2.66 | 15.40 | negative | 17 |  |  |
| 225 | female | 74 | negative | 277.50 | 21.91 | 11.29 | 12.67 | negative | 15 |  |  |
| 226 | male | 73 | negative | 117.99 | 19.40 | 5.76 | 6.08 | negative | 19 |  |  |
| 227 | male | 75 | negative | 300.00 | 15.83 | 33.83 | 18.95 | negative | 19 |  |  |
| 228 | male | 74 | negative | 198.67 | 14.13 | 8.29 | 14.06 | negative | 19 |  |  |
| 229 | male | 74 | negative | 300.00 | 27.05 | 2.15 | 11.09 | negative | 17 |  |  |
| 230 | female | 75 | positive | 109.76 | 16.69 | 7.39 | 6.58 | negative | 16 |  |  |
| 231 | male | 75 | negative | 56.50 | 7.50 | 1.60 | 7.53 | negative | 17 |  |  |
| 232 | male | 74 | positive | 148.39 | 15.63 | 3.75 | 9.49 | negative | 18 |  |  |
| 233 | male | 75 | positive | 230.46 | 25.48 | 12.55 | 9.04 | negative | 20 |  |  |
| 234 | female | 74 | positive | 173.42 | 31.76 | 8.91 | 5.46 | negative | 16 |  |  |
| 235 | female | 74 | negative | 98.50 | 8.35 | 3.33 | 11.80 | negative | 13 |  |  |
| 236 | female | 74 | negative | 67.42 | 4.47 | 2.67 | 15.08 | negative | 13 |  |  |
| 237 | male | 74 | negative | 42.33 | 9.69 | 2.39 | 4.37 | negative | 17 |  |  |
| 238 | male | 75 | negative | 106.37 | 8.75 | 3.35 | 12.16 | negative | 17 |  |  |
| 239 | female | 74 | positive | 120.12 | 21.21 | 4.87 | 5.66 | negative | 14 |  |  |
| 240 | female | 74 | negative | 101.72 | 12.15 | 8.93 | 8.37 | negative | 15 |  |  |
| 241 | male | 74 | negative | 171.81 | 12.90 | 3.00 | 13.32 | negative | 17 |  |  |
| 242 | female | 74 | positive | 182.80 | 25.24 | 13.12 | 7.24 | negative | 16 |  |  |
| 243 | male | 74 | negative | 107.57 | 9.96 | 5.80 | 10.80 | negative | 19 |  |  |
| 244 | male | 74 | negative | 102.72 | 9.67 | 0.67 | 10.62 | negative | 14 |  |  |
| 245 | male | 75 | negative | 277.38 | 19.22 | 5.82 | 14.43 | negative | 19 |  |  |
| 246 | male | 73 | negative | 69.10 | 11.67 | 6.90 | 5.92 | negative | 19 |  |  |
| 247 | male | 76 | negative | 124.43 | 11.97 | 0.84 | 10.40 | negative | 14 |  |  |
| 248 | male | 74 | positive | 108.95 | 17.28 | 4.14 | 6.30 | negative | 18 |  |  |
| 249 | female | 73 | negative | 89.65 | 6.87 | 7.77 | 13.05 | negative | 15 |  |  |
| 250 | male | 75 | negative | 124.07 | 14.14 | 1.51 | 8.77 | negative | 17 |  |  |
| 251 | female | 75 | negative | 63.38 | 5.11 | 1.65 | 12.40 | negative | 13 |  |  |
| 252 | male | 73 | positive | 182.33 | 12.79 | 8.64 | 14.26 | negative | 20 |  |  |
| 253 | female | 74 | positive | 82.99 | 11.95 | 4.30 | 6.94 | negative | 14 |  |  |
| 254 | male | 74 | positive | 112.90 | 18.80 | 3.00 | 6.01 | negative | 18 |  |  |
| 255 | male | 75 | positive | 63.84 | 16.46 | 5.93 | 3.88 | negative | 23 |  |  |
| 256 | male | 74 | negative | 300.00 | 34.65 | 5.09 | 8.66 | negative | 17 |  |  |
| 257 | male | 74 | negative | 60.62 | 12.41 | 6.13 | 4.88 | negative | 19 |  |  |
| 258 | male | 75 | negative | 212.52 | 9.29 | 0.10 | 22.88 | negative | 14 |  |  |
| 259 | male | 73 | negative | 93.31 | 5.88 | 7.76 | 15.87 | negative | 19 |  |  |
| 260 | female | 76 | negative | 191.07 | 12.58 | 11.98 | 15.19 | negative | 15 |  |  |
| 261 | male | 75 | positive | 73.33 | 10.15 | 0.50 | 7.22 | negative | 15 |  |  |
| 262 | male | 76 | negative | 59.53 | 5.67 | 1.31 | 10.50 | negative | 14 |  |  |
| 263 | male | 74 | positive | 136.97 | 14.35 | 9.75 | 9.54 | negative | 20 |  |  |
| 264 | female | 74 | negative | 91.84 | 12.22 | 9.06 | 7.52 | negative | 15 |  |  |
| 265 | female | 74 | negative | 50.13 | 7.31 | 16.60 | 6.86 | negative | 15 |  |  |
| 266 | male | 74 | negative | 191.00 | 13.56 | 4.89 | 14.09 | negative | 17 |  |  |
| 267 | female | 73 | negative | 40.77 | 2.90 | 7.49 | 14.06 | negative | 15 |  |  |
| 268 | male | 75 | negative | 54.60 | 10.80 | 2.80 | 5.06 | negative | 17 |  |  |
| 269 | male | 74 | negative | 139.90 | 14.51 | 2.38 | 9.64 | negative | 17 |  |  |
| 270 | male | 74 | negative | 98.10 | 15.60 | 4.30 | 6.29 | negative | 17 |  |  |
| 271 | female | 75 | negative | 89.94 | 6.61 | 4.24 | 13.61 | negative | 13 |  |  |
| 272 | female | 74 | positive | 41.23 | 8.05 | 3.84 | 5.12 | negative | 14 |  |  |
| 273 | female | 72 | negative | 198.88 | 12.02 | 23.69 | 16.55 | negative | 15 |  |  |
| 274 | female | 73 | negative | 77.19 | 6.71 | 1.78 | 11.50 | negative | 13 |  |  |
| 275 | male | 72 | positive | 91.34 | 12.66 | 9.35 | 7.21 | negative | 20 |  |  |
| 276 | male | 72 | negative | 169.53 | 22.33 | 11.50 | 7.59 | negative | 19 |  |  |
| 277 | male | 72 | positive | 98.50 | 13.82 | 6.50 | 7.13 | negative | 20 |  |  |
| 278 | female | 73 | positive | 217.15 | 19.38 | 4.10 | 11.20 | negative | 14 |  |  |
| 279 | female | 72 | negative | 64.55 | 2.55 | 2.14 | 25.31 | negative | 13 |  |  |
| 280 | male | 73 | negative | 99.70 | 6.50 | 2.10 | 15.34 | negative | 17 |  |  |
| 281 | female | 73 | negative | 34.07 | 16.42 | 36.71 | 2.07 | negative | 18 |  |  |
| 282 | female | 72 | negative | 300.00 | 15.69 | 38.37 | 19.12 | negative | 15 |  |  |
| 283 | male | 73 | negative | 172.63 | 9.46 | 2.16 | 18.25 | negative | 17 |  |  |
| 284 | male | 73 | negative | 47.75 | 4.77 | 11.21 | 10.01 | negative | 19 |  |  |
| 285 | male | 73 | negative | 91.20 | 7.17 | 2.75 | 12.72 | negative | 17 |  |  |
| 286 | female | 73 | negative | 83.21 | 6.83 | 1.65 | 12.18 | negative | 13 |  |  |
| 287 | male | 72 | negative | 68.67 | 3.49 | 1.06 | 19.68 | negative | 14 |  |  |
| 288 | female | 72 | negative | 164.81 | 14.72 | 5.20 | 11.20 | negative | 13 |  |  |
| 289 | male | 72 | negative | 72.41 | 5.24 | 2.71 | 13.82 | negative | 17 |  |  |
| 290 | male | 72 | negative | 141.83 | 13.53 | 7.10 | 10.48 | negative | 19 |  |  |
| 291 | female | 72 | negative | 66.00 | 11.50 | 2.70 | 5.74 | negative | 13 |  |  |
| 292 | male | 72 | negative | 94.97 | 5.15 | 3.31 | 18.44 | negative | 17 |  |  |
| 293 | male | 72 | negative | 160.04 | 13.55 | 7.15 | 11.81 | negative | 19 |  |  |
| 294 | male | 73 | negative | 126.28 | 9.09 | 4.83 | 13.89 | negative | 17 |  |  |
| 295 | male | 72 | negative | 101.63 | 10.18 | 6.46 | 9.98 | negative | 19 |  |  |
| 296 | female | 72 | positive | 145.38 | 18.01 | 2.14 | 8.07 | negative | 14 |  |  |
| 297 | female | 72 | negative | 106.19 | 7.73 | 5.08 | 13.74 | negative | 13 |  |  |
| 298 | female | 73 | negative | 71.05 | 4.84 | 2.30 | 14.68 | negative | 13 |  |  |
| 299 | male | 72 | positive | 194.00 | 16.38 | 4.43 | 11.84 | negative | 18 |  |  |
| 300 | male | 73 | negative | 178.79 | 15.78 | 13.53 | 11.33 | negative | 19 |  |  |
| 301 | female | 72 | positive | 126.66 | 23.35 | 19.48 | 5.42 | negative | 16 |  |  |
| 302 | female | 73 | negative | 99.79 | 5.09 | 2.20 | 19.61 | negative | 13 |  |  |
| 303 | male | 73 | negative | 277.52 | 13.66 | 3.70 | 20.32 | negative | 17 |  |  |
| 304 | female | 72 | negative | 91.09 | 6.27 | 2.52 | 14.53 | negative | 13 |  |  |
| 305 | female | 73 | positive | 131.70 | 23.10 | 3.20 | 5.70 | negative | 14 |  |  |
| 306 | male | 72 | negative | 139.52 | 5.78 | 7.38 | 24.14 | negative | 19 |  |  |
| 307 | male | 72 | negative | 94.69 | 10.64 | 1.11 | 8.90 | negative | 14 |  |  |
| 308 | male | 73 | negative | 150.03 | 9.97 | 4.08 | 15.05 | negative | 17 |  |  |
| 309 | male | 72 | negative | 156.56 | 17.47 | 1.70 | 8.96 | negative | 17 |  |  |
| 310 | female | 73 | positive | 93.28 | 13.11 | 2.37 | 7.12 | negative | 14 |  |  |
| 311 | male | 72 | positive | 119.87 | 25.06 | 4.35 | 4.78 | negative | 18 |  |  |
| 312 | female | 72 | negative | 56.31 | 10.09 | 1.79 | 5.58 | negative | 13 |  |  |
| 313 | male | 72 | negative | 122.87 | 13.17 | 8.61 | 9.33 | negative | 19 |  |  |
| 314 | male | 72 | negative | 148.30 | 9.52 | 1.73 | 15.58 | negative | 17 |  |  |
| 315 | male | 73 | negative | 73.63 | 8.92 | 1.60 | 8.25 | negative | 17 |  |  |
| 316 | male | 72 | positive | 288.36 | 40.68 | 13.65 | 7.09 | negative | 20 |  |  |
| 317 | male | 73 | negative | 11.55 | 5.15 | 11.77 | 2.24 | negative | 22 |  |  |
| 318 | male | 72 | negative | 128.42 | 8.87 | 4.31 | 14.48 | negative | 17 |  |  |
| 319 | male | 73 | negative | 77.56 | 4.64 | 2.45 | 16.72 | negative | 17 |  |  |
| 320 | male | 72 | positive | 95.86 | 14.93 | 5.35 | 6.42 | negative | 18 |  |  |
| 321 | female | 73 | negative | 244.12 | 21.48 | 31.11 | 11.36 | negative | 15 |  |  |
| 322 | male | 72 | negative | 86.52 | 10.16 | 7.82 | 8.52 | negative | 19 |  |  |
| 323 | male | 71 | negative | 96.90 | 7.80 | 9.90 | 12.42 | negative | 19 |  |  |
| 324 | female | 73 | positive | 88.97 | 11.65 | 4.70 | 7.64 | negative | 14 |  |  |
| 325 | female | 73 | negative | 104.24 | 9.33 | 2.42 | 11.17 | negative | 13 |  |  |
| 326 | female | 72 | negative | 103.49 | 13.07 | 5.34 | 7.92 | negative | 13 |  |  |
| 327 | male | 73 | negative | 140.28 | 6.34 | 1.11 | 22.13 | negative | 14 |  |  |
| 328 | female | 73 | negative | 77.36 | 4.29 | 2.41 | 18.03 | negative | 13 |  |  |
| 329 | male | 72 | negative | 300.00 | 24.42 | 10.84 | 12.29 | negative | 19 |  |  |
| 330 | female | 72 | negative | 20.99 | 4.01 | 60.00 | 5.23 | negative | 15 |  |  |
| 331 | male | 72 | negative | 159.96 | 11.51 | 1.78 | 13.90 | negative | 17 |  |  |
| 332 | male | 72 | negative | 139.54 | 8.24 | 1.77 | 16.93 | negative | 17 |  |  |
| 333 | male | 72 | positive | 45.97 | 13.40 | 2.91 | 3.43 | negative | 21 |  |  |
| 334 | female | 72 | negative | 72.04 | 7.80 | 3.36 | 9.24 | negative | 13 |  |  |
| 335 | female | 72 | negative | 157.33 | 10.31 | 1.76 | 15.26 | negative | 13 |  |  |
| 336 | female | 73 | negative | 186.18 | 14.41 | 1.90 | 12.92 | negative | 13 |  |  |
| 337 | male | 73 | negative | 157.30 | 13.70 | 4.10 | 11.48 | negative | 17 |  |  |
| 338 | female | 72 | positive | 171.96 | 22.22 | 5.80 | 7.74 | negative | 16 |  |  |
| 339 | male | 72 | negative | 173.84 | 6.34 | 3.29 | 27.42 | negative | 17 |  |  |
| 340 | female | 72 | negative | 107.35 | 19.28 | 13.72 | 5.57 | negative | 15 |  |  |
| 341 | male | 72 | negative | 97.70 | 8.72 | 1.29 | 11.20 | negative | 14 |  |  |
| 342 | male | 73 | negative | 96.42 | 6.58 | 3.54 | 14.65 | negative | 17 |  |  |
| 343 | male | 72 | negative | 279.67 | 13.69 | 3.89 | 20.43 | negative | 17 |  |  |
| 344 | male | 73 | negative | 83.36 | 5.41 | 0.94 | 15.41 | negative | 14 |  |  |
| 345 | female | 72 | negative | 271.80 | 18.72 | 6.47 | 14.52 | negative | 15 |  |  |
| 346 | male | 73 | negative | 54.20 | 8.10 | 36.60 | 6.69 | negative | 19 |  |  |
| 347 | male | 72 | negative | 247.53 | 13.03 | 5.41 | 19.00 | negative | 17 |  |  |
| 348 | male | 72 | negative | 143.64 | 22.71 | 10.82 | 6.32 | negative | 19 |  |  |
| 349 | female | 72 | negative | 83.78 | 5.45 | 3.27 | 15.37 | negative | 13 |  |  |
| 350 | male | 72 | negative | 151.63 | 7.52 | 6.57 | 20.16 | negative | 19 |  |  |
| 351 | male | 73 | negative | 159.22 | 17.68 | 3.40 | 9.01 | negative | 17 |  |  |
| 352 | male | 72 | positive | 72.40 | 15.32 | 5.18 | 4.73 | negative | 18 |  |  |
| 353 | female | 72 | negative | 67.28 | 4.23 | 5.09 | 15.91 | negative | 13 |  |  |
| 354 | male | 72 | negative | 103.80 | 6.89 | 6.27 | 15.07 | negative | 19 |  |  |
| 355 | male | 73 | negative | 117.40 | 8.25 | 1.51 | 14.23 | negative | 17 |  |  |
| 356 | female | 72 | negative | 97.59 | 7.74 | 2.00 | 12.61 | negative | 13 |  |  |
| 357 | female | 72 | negative | 93.72 | 8.74 | 5.49 | 10.72 | negative | 13 |  |  |
| 358 | male | 72 | positive | 160.45 | 21.29 | 5.40 | 7.54 | negative | 18 |  |  |
| 359 | male | 72 | negative | 98.79 | 19.54 | 58.97 | 5.06 | negative | 19 |  |  |
| 360 | female | 73 | negative | 65.15 | 4.95 | 2.99 | 13.16 | negative | 13 |  |  |
| 361 | male | 72 | negative | 105.40 | 9.65 | 3.11 | 10.92 | negative | 17 |  |  |
| 362 | female | 72 | positive | 153.07 | 26.00 | 9.89 | 5.89 | negative | 16 |  |  |
| 363 | male | 72 | positive | 63.44 | 11.79 | 4.35 | 5.38 | negative | 18 |  |  |
| 364 | female | 73 | negative | 105.76 | 8.99 | 2.61 | 11.76 | negative | 13 |  |  |
| 365 | male | 72 | negative | 107.96 | 5.77 | 2.67 | 18.71 | negative | 17 |  |  |
| 366 | female | 72 | negative | 129.39 | 11.92 | 3.67 | 10.85 | negative | 13 |  |  |
| 367 | male | 72 | negative | 59.55 | 6.21 | 8.82 | 9.59 | negative | 19 |  |  |
| 368 | male | 72 | negative | 141.01 | 7.65 | 4.01 | 18.43 | negative | 17 |  |  |
| 369 | male | 73 | positive | 135.34 | 18.86 | 3.39 | 7.18 | negative | 18 |  |  |
| 370 | female | 73 | positive | 279.87 | 28.26 | 2.64 | 9.90 | negative | 14 |  |  |
| 371 | male | 72 | negative | 86.71 | 4.69 | 1.86 | 18.49 | negative | 17 |  |  |
| 372 | male | 72 | negative | 144.58 | 6.80 | 4.14 | 21.26 | negative | 17 |  |  |
| 373 | female | 73 | negative | 128.94 | 8.39 | 4.40 | 15.37 | negative | 13 |  |  |
| 374 | female | 73 | negative | 72.52 | 7.87 | 1.54 | 9.21 | negative | 13 |  |  |
| 375 | female | 72 | positive | 225.62 | 47.11 | 50.15 | 4.79 | negative | 16 |  |  |
| 376 | female | 72 | negative | 72.01 | 4.67 | 2.45 | 15.42 | negative | 13 |  |  |
| 377 | female | 73 | negative | 139.98 | 11.04 | 2.39 | 12.68 | negative | 13 |  |  |
| 378 | female | 73 | negative | 40.66 | 12.39 | 27.85 | 3.28 | negative | 18 |  |  |
| 379 | male | 72 | positive | 297.68 | 21.11 | 8.93 | 14.10 | negative | 20 |  |  |
| 380 | male | 73 | negative | 207.61 | 14.62 | 1.31 | 14.20 | negative | 14 |  |  |
| 381 | male | 73 | negative | 93.60 | 7.15 | 3.79 | 13.09 | negative | 17 |  |  |
| 382 | male | 72 | negative | 142.30 | 12.40 | 0.88 | 11.48 | negative | 14 |  |  |
| 383 | male | 73 | negative | 127.80 | 17.50 | 11.60 | 7.30 | negative | 19 |  |  |
| 384 | female | 73 | negative | 64.12 | 6.64 | 4.58 | 9.66 | negative | 13 |  |  |
| 385 | male | 73 | negative | 91.32 | 5.68 | 1.64 | 16.08 | negative | 17 |  |  |
| 386 | female | 73 | positive | 72.30 | 5.10 | 2.80 | 14.18 | negative | 14 |  |  |
| 387 | male | 73 | negative | 73.07 | 3.28 | 1.67 | 22.28 | negative | 17 |  |  |
| 388 | male | 73 | positive | 128.20 | 17.50 | 2.60 | 7.33 | negative | 18 |  |  |
| 389 | female | 72 | negative | 100.94 | 9.69 | 3.44 | 10.42 | negative | 13 |  |  |
| 390 | female | 72 | positive | 90.24 | 19.99 | 4.87 | 4.51 | negative | 14 |  |  |
| 391 | female | 72 | negative | 237.01 | 16.37 | 4.55 | 14.48 | negative | 13 |  |  |
| 392 | male | 72 | negative | 117.13 | 8.35 | 0.76 | 14.03 | negative | 14 |  |  |
| 393 | male | 72 | positive | 133.22 | 24.53 | 3.86 | 5.43 | negative | 18 |  |  |
| 394 | male | 73 | negative | 112.98 | 7.17 | 1.03 | 15.76 | negative | 14 |  |  |
| 395 | male | 72 | positive | 105.30 | 14.20 | 3.70 | 7.42 | negative | 18 |  |  |
| 396 | male | 72 | negative | 74.44 | 11.00 | 5.66 | 6.77 | negative | 17 |  |  |
| 397 | male | 73 | negative | 93.23 | 6.51 | 2.16 | 14.32 | negative | 17 |  |  |
| 398 | female | 73 | positive | 300.00 | 25.02 | 3.03 | 11.99 | negative | 14 |  |  |
| 399 | male | 73 | negative | 39.18 | 4.56 | 4.80 | 8.59 | negative | 17 |  |  |
| 400 | female | 70 | positive | 84.20 | 13.40 | 6.40 | 6.28 | negative | 16 |  |  |
| 401 | male | 71 | negative | 159.73 | 9.54 | 1.42 | 16.74 | negative | 14 |  |  |
| 402 | male | 70 | negative | 26.53 | 10.56 | 53.62 | 2.51 | negative | 22 |  |  |
| 403 | male | 71 | negative | 156.49 | 16.57 | 2.21 | 9.44 | negative | 17 |  |  |
| 404 | female | 71 | negative | 275.60 | 13.95 | 6.00 | 19.76 | negative | 15 |  |  |
| 405 | male | 71 | negative | 112.70 | 9.90 | 2.20 | 11.38 | negative | 17 |  |  |
| 406 | male | 71 | negative | 271.89 | 16.33 | 4.86 | 16.65 | negative | 17 |  |  |
| 407 | male | 71 | negative | 123.48 | 9.95 | 2.96 | 12.41 | negative | 17 |  |  |
| 408 | male | 71 | negative | 148.79 | 10.05 | 1.56 | 14.80 | negative | 17 |  |  |
| 409 | female | 71 | positive | 63.19 | 11.78 | 4.91 | 5.36 | negative | 14 |  |  |
| 410 | male | 71 | positive | 166.84 | 13.75 | 9.80 | 12.13 | negative | 20 |  |  |
| 411 | male | 71 | negative | 158.81 | 8.49 | 2.42 | 18.71 | negative | 17 |  |  |
| 412 | female | 71 | negative | 81.53 | 7.68 | 2.62 | 10.62 | negative | 13 |  |  |
| 413 | female | 70 | negative | 81.60 | 8.60 | 6.40 | 9.49 | negative | 15 |  |  |
| 414 | female | 70 | negative | 147.94 | 14.97 | 6.90 | 9.88 | negative | 15 |  |  |
| 415 | male | 71 | negative | 24.59 | 4.57 | 5.17 | 5.38 | negative | 17 |  |  |
| 416 | male | 71 | negative | 300.00 | 16.64 | 1.45 | 18.03 | negative | 14 |  |  |
| 417 | male | 71 | negative | 98.31 | 4.63 | 2.81 | 21.23 | negative | 17 |  |  |
| 418 | male | 71 | positive | 140.90 | 21.98 | 5.46 | 6.41 | negative | 18 |  |  |
| 419 | male | 71 | negative | 113.14 | 8.73 | 1.28 | 12.96 | negative | 14 |  |  |
| 420 | male | 71 | negative | 184.95 | 15.43 | 2.72 | 11.99 | negative | 17 |  |  |
| 421 | female | 71 | negative | 60.62 | 3.74 | 3.64 | 16.21 | negative | 13 |  |  |
| 422 | male | 70 | negative | 188.56 | 15.01 | 6.77 | 12.56 | negative | 19 |  |  |
| 423 | male | 70 | positive | 135.11 | 21.67 | 9.77 | 6.23 | negative | 20 |  |  |
| 424 | male | 71 | negative | 55.33 | 4.90 | 2.02 | 11.29 | negative | 17 |  |  |
| 425 | female | 71 | negative | 93.27 | 9.36 | 10.94 | 9.96 | negative | 15 |  |  |
| 426 | male | 71 | positive | 300.00 | 31.17 | 6.40 | 9.62 | negative | 20 |  |  |
| 427 | male | 71 | negative | 144.90 | 20.90 | 3.70 | 6.93 | negative | 17 |  |  |
| 428 | male | 71 | positive | 45.22 | 18.36 | 18.96 | 2.46 | negative | 23 |  |  |
| 429 | female | 71 | negative | 66.57 | 12.58 | 2.57 | 5.29 | negative | 13 |  |  |
| 430 | male | 71 | negative | 33.88 | 2.76 | 0.48 | 12.28 | negative | 14 |  |  |
| 431 | male | 71 | negative | 106.83 | 12.44 | 0.98 | 8.59 | negative | 14 |  |  |
| 432 | female | 71 | negative | 123.89 | 12.36 | 2.81 | 10.02 | negative | 13 |  |  |
| 433 | male | 71 | negative | 32.15 | 5.74 | 0.55 | 5.60 | negative | 14 |  |  |
| 434 | female | 71 | negative | 164.83 | 13.33 | 6.68 | 12.37 | negative | 15 |  |  |
| 435 | male | 70 | positive | 161.01 | 20.66 | 8.22 | 7.79 | negative | 20 |  |  |
| 436 | male | 71 | positive | 181.49 | 18.34 | 11.96 | 9.90 | negative | 20 |  |  |
| 437 | female | 70 | negative | 23.58 | 6.17 | 51.20 | 3.82 | negative | 18 |  |  |
| 438 | male | 71 | positive | 136.42 | 15.38 | 7.43 | 8.87 | negative | 20 |  |  |
| 439 | male | 70 | negative | 144.05 | 10.97 | 4.35 | 13.13 | negative | 17 |  |  |
| 440 | male | 71 | negative | 91.73 | 8.93 | 4.60 | 10.27 | negative | 17 |  |  |
| 441 | female | 71 | negative | 90.79 | 8.99 | 2.81 | 10.10 | negative | 13 |  |  |
| 442 | male | 71 | negative | 118.68 | 10.05 | 6.31 | 11.81 | negative | 19 |  |  |
| 443 | female | 71 | negative | 65.21 | 28.35 | 35.73 | 2.30 | negative | 18 |  |  |
| 444 | female | 71 | negative | 131.21 | 8.75 | 1.85 | 15.00 | negative | 13 |  |  |
| 445 | female | 71 | negative | 136.48 | 12.07 | 4.02 | 11.31 | negative | 13 |  |  |
| 446 | female | 70 | positive | 100.83 | 10.84 | 6.28 | 9.30 | negative | 16 |  |  |
| 447 | male | 71 | negative | 195.32 | 8.98 | 4.04 | 21.75 | negative | 17 |  |  |
| 448 | male | 71 | negative | 87.62 | 3.78 | 1.20 | 23.18 | negative | 14 |  |  |
| 449 | male | 70 | positive | 294.67 | 41.88 | 5.07 | 7.04 | negative | 18 |  |  |
| 450 | female | 70 | negative | 89.98 | 8.57 | 4.45 | 10.50 | negative | 13 |  |  |
| 451 | female | 71 | negative | 90.88 | 8.71 | 7.80 | 10.43 | negative | 15 |  |  |
| 452 | female | 70 | negative | 53.40 | 9.99 | 6.51 | 5.35 | negative | 15 |  |  |
| 453 | male | 70 | negative | 109.54 | 15.12 | 54.68 | 7.24 | negative | 19 |  |  |
| 454 | female | 71 | positive | 84.93 | 14.32 | 3.72 | 5.93 | negative | 14 |  |  |
| 455 | male | 71 | negative | 203.89 | 15.88 | 1.92 | 12.84 | negative | 17 |  |  |
| 456 | male | 71 | negative | 97.98 | 10.68 | 5.68 | 9.17 | negative | 17 |  |  |
| 457 | male | 70 | positive | 52.40 | 8.58 | 4.50 | 6.11 | negative | 18 |  |  |
| 458 | male | 71 | negative | 74.35 | 4.23 | 2.69 | 17.58 | negative | 17 |  |  |
| 459 | female | 70 | negative | 86.11 | 6.98 | 6.07 | 12.34 | negative | 15 |  |  |
| 460 | male | 71 | positive | 63.84 | 20.42 | 12.69 | 3.13 | negative | 23 |  |  |
| 461 | male | 71 | negative | 98.09 | 10.72 | 3.55 | 9.15 | negative | 17 |  |  |
| 462 | female | 71 | negative | 235.37 | 19.40 | 3.77 | 12.13 | negative | 13 |  |  |
| 463 | female | 70 | negative | 129.91 | 8.01 | 4.45 | 16.22 | negative | 13 |  |  |
| 464 | female | 70 | negative | 65.37 | 4.03 | 4.73 | 16.22 | negative | 13 |  |  |
| 465 | female | 71 | positive | 283.75 | 25.77 | 4.28 | 11.01 | negative | 14 |  |  |
| 466 | female | 70 | positive | 206.71 | 27.06 | 9.79 | 7.64 | negative | 16 |  |  |
| 467 | male | 70 | positive | 104.88 | 10.48 | 7.06 | 10.01 | negative | 20 |  |  |
| 468 | male | 71 | negative | 131.40 | 10.70 | 1.70 | 12.28 | negative | 17 |  |  |
| 469 | male | 71 | negative | 163.20 | 8.69 | 1.33 | 18.78 | negative | 14 |  |  |
| 470 | male | 70 | positive | 183.56 | 23.80 | 6.60 | 7.71 | negative | 20 |  |  |
| 471 | male | 71 | negative | 108.66 | 11.33 | 7.42 | 9.59 | negative | 19 |  |  |
| 472 | male | 70 | positive | 117.50 | 11.95 | 5.91 | 9.83 | negative | 20 |  |  |
| 473 | female | 71 | negative | 89.28 | 13.60 | 5.39 | 6.56 | negative | 13 |  |  |
| 474 | female | 70 | positive | 83.64 | 9.57 | 8.95 | 8.74 | negative | 16 |  |  |
| 475 | male | 71 | negative | 139.07 | 16.09 | 3.02 | 8.64 | negative | 17 |  |  |
| 476 | male | 71 | positive | 159.05 | 19.46 | 2.43 | 8.17 | negative | 18 |  |  |
| 477 | female | 70 | positive | 111.86 | 25.95 | 9.00 | 4.31 | negative | 16 |  |  |
| 478 | male | 71 | negative | 80.03 | 7.38 | 2.43 | 10.84 | negative | 17 |  |  |
| 479 | female | 71 | positive | 281.63 | 39.54 | 6.34 | 7.12 | negative | 16 |  |  |
| 480 | female | 71 | negative | 81.13 | 12.31 | 3.37 | 6.59 | negative | 13 |  |  |
| 481 | female | 71 | negative | 129.38 | 11.16 | 3.34 | 11.59 | negative | 13 |  |  |
| 482 | male | 71 | positive | 159.65 | 13.05 | 8.53 | 12.23 | negative | 20 |  |  |
| 483 | female | 71 | negative | 134.77 | 24.05 | 37.13 | 5.60 | negative | 15 |  |  |
| 484 | male | 71 | positive | 300.00 | 29.88 | 9.64 | 10.04 | negative | 20 |  |  |
| 485 | male | 71 | positive | 185.71 | 16.18 | 7.18 | 11.48 | negative | 20 |  |  |
| 486 | male | 70 | positive | 68.19 | 12.94 | 9.07 | 5.27 | negative | 20 |  |  |
| 487 | male | 71 | positive | 124.26 | 17.28 | 8.89 | 7.19 | negative | 20 |  |  |
| 488 | male | 71 | positive | 154.47 | 21.13 | 21.11 | 7.31 | negative | 20 |  |  |
| 489 | male | 71 | positive | 32.73 | 16.23 | 13.70 | 2.02 | negative | 23 |  |  |
| 490 | male | 71 | negative | 122.69 | 8.28 | 4.37 | 14.82 | negative | 17 |  |  |
| 491 | male | 71 | negative | 23.14 | 13.34 | 60.00 | 1.73 | negative | 22 |  |  |
| 492 | female | 71 | positive | 101.56 | 19.79 | 8.90 | 5.13 | negative | 16 |  |  |
| 493 | male | 71 | negative | 114.68 | 15.08 | 6.08 | 7.60 | negative | 19 |  |  |
| 494 | female | 71 | positive | 233.23 | 24.87 | 3.48 | 9.38 | negative | 14 |  |  |
| 495 | male | 70 | negative | 116.34 | 6.84 | 6.12 | 17.01 | negative | 19 |  |  |
| 496 | female | 71 | negative | 122.77 | 10.81 | 4.30 | 11.36 | negative | 13 |  |  |
| 497 | male | 71 | negative | 52.33 | 6.58 | 1.69 | 7.95 | negative | 17 |  |  |
| 498 | male | 71 | positive | 37.69 | 10.19 | 1.51 | 3.70 | negative | 21 |  |  |
| 499 | male | 71 | negative | 124.46 | 10.25 | 2.02 | 12.14 | negative | 17 |  |  |
| 500 | male | 70 | negative | 124.73 | 5.28 | 6.73 | 23.62 | negative | 19 |  |  |
| 501 | male | 71 | negative | 123.44 | 6.48 | 0.50 | 19.05 | negative | 14 |  |  |
| 502 | male | 71 | negative | 92.74 | 7.46 | 2.90 | 12.43 | negative | 17 |  |  |
| 503 | female | 71 | positive | 205.78 | 29.56 | 6.21 | 6.96 | negative | 16 |  |  |
| 504 | male | 71 | negative | 84.91 | 2.57 | 1.93 | 33.04 | negative | 17 |  |  |
| 505 | female | 71 | negative | 54.89 | 3.54 | 2.21 | 15.51 | negative | 13 |  |  |
| 506 | male | 71 | negative | 109.33 | 5.25 | 2.41 | 20.82 | negative | 17 |  |  |
| 507 | female | 70 | positive | 207.41 | 28.77 | 8.17 | 7.21 | negative | 16 |  |  |
| 508 | male | 71 | positive | 101.53 | 11.79 | 1.91 | 8.61 | negative | 18 |  |  |
| 509 | male | 70 | negative | 104.07 | 13.59 | 5.58 | 7.66 | negative | 17 |  |  |
| 510 | female | 70 | negative | 149.51 | 14.52 | 9.23 | 10.30 | negative | 15 |  |  |
| 511 | female | 70 | positive | 69.50 | 9.11 | 8.66 | 7.63 | negative | 16 |  |  |
| 512 | female | 70 | negative | 81.06 | 6.16 | 5.18 | 13.16 | negative | 13 |  |  |
| 513 | male | 71 | positive | 223.93 | 21.43 | 15.61 | 10.45 | negative | 20 |  |  |
| 514 | male | 70 | negative | 102.82 | 5.19 | 4.69 | 19.81 | negative | 17 |  |  |
| 515 | male | 71 | positive | 133.28 | 17.49 | 7.19 | 7.62 | negative | 20 |  |  |
| 516 | male | 71 | negative | 300.00 | 20.55 | 4.03 | 14.60 | negative | 17 |  |  |
| 517 | male | 71 | positive | 73.66 | 8.62 | 3.72 | 8.55 | negative | 18 |  |  |
| 518 | male | 71 | negative | 159.10 | 5.48 | 2.25 | 29.03 | negative | 17 |  |  |
| 519 | male | 71 | positive | 232.08 | 34.41 | 7.56 | 6.74 | negative | 20 |  |  |
| 520 | male | 71 | negative | 110.06 | 4.15 | 5.91 | 26.52 | negative | 19 |  |  |
| 521 | male | 71 | negative | 117.12 | 12.77 | 2.24 | 9.17 | negative | 17 |  |  |
| 522 | male | 71 | negative | 291.35 | 20.81 | 11.15 | 14.00 | negative | 19 |  |  |
| 523 | male | 71 | negative | 122.93 | 15.19 | 3.67 | 8.09 | negative | 17 |  |  |
| 524 | female | 71 | positive | 176.44 | 29.48 | 4.41 | 5.99 | negative | 14 |  |  |
| 525 | female | 71 | positive | 50.61 | 11.50 | 12.41 | 4.40 | negative | 16 |  |  |
| 526 | male | 71 | negative | 118.47 | 4.75 | 3.72 | 24.94 | negative | 17 |  |  |
| 527 | female | 71 | negative | 118.88 | 7.53 | 2.16 | 15.79 | negative | 13 |  |  |
| 528 | female | 71 | positive | 141.36 | 18.57 | 12.48 | 7.61 | negative | 16 |  |  |
| 529 | male | 71 | negative | 156.53 | 10.48 | 2.89 | 14.94 | negative | 17 |  |  |
| 530 | male | 71 | negative | 168.50 | 8.05 | 3.36 | 20.93 | negative | 17 |  |  |
| 531 | male | 71 | positive | 144.50 | 12.94 | 9.49 | 11.17 | negative | 20 |  |  |
| 532 | male | 71 | positive | 219.08 | 21.00 | 4.22 | 10.43 | negative | 18 |  |  |
| 533 | female | 70 | negative | 51.40 | 5.80 | 4.50 | 8.86 | negative | 13 |  |  |
| 534 | female | 71 | negative | 288.60 | 15.39 | 35.28 | 18.75 | negative | 15 |  |  |
| 535 | male | 71 | negative | 172.41 | 13.58 | 1.26 | 12.70 | negative | 14 |  |  |
| 536 | female | 71 | positive | 182.85 | 21.25 | 11.20 | 8.60 | negative | 16 |  |  |
| 537 | female | 70 | positive | 123.01 | 17.25 | 9.76 | 7.13 | negative | 16 |  |  |
| 538 | female | 71 | negative | 32.63 | 10.69 | 58.64 | 3.05 | negative | 18 |  |  |
| 539 | male | 69 | positive | 146.84 | 17.18 | 7.13 | 8.55 | negative | 16 |  |  |
| 540 | male | 69 | negative | 152.10 | 10.55 | 3.44 | 14.42 | negative | 13 |  |  |
| 541 | male | 69 | negative | 114.20 | 13.70 | 2.20 | 8.34 | negative | 13 |  |  |
| 542 | male | 69 | negative | 112.30 | 14.00 | 3.30 | 8.02 | negative | 13 |  |  |
| 543 | female | 69 | positive | 300.00 | 19.44 | 15.13 | 15.43 | negative | 12 |  |  |
| 544 | female | 70 | negative | 103.69 | 7.62 | 2.97 | 13.61 | negative | 13 |  |  |
| 545 | male | 69 | negative | 201.04 | 15.47 | 3.55 | 13.00 | negative | 13 |  |  |
| 546 | female | 70 | negative | 63.72 | 4.74 | 1.74 | 13.44 | negative | 13 |  |  |
| 547 | male | 69 | negative | 233.08 | 12.36 | 2.11 | 18.86 | negative | 13 |  |  |
| 548 | male | 70 | negative | 69.07 | 8.72 | 0.65 | 7.92 | negative | 14 |  |  |
| 549 | male | 70 | negative | 126.32 | 12.98 | 10.19 | 9.73 | negative | 19 |  |  |
| 550 | male | 70 | negative | 300.00 | 24.03 | 2.19 | 12.48 | negative | 17 |  |  |
| 551 | male | 69 | negative | 58.93 | 7.48 | 2.63 | 7.88 | negative | 13 |  |  |
| 552 | female | 70 | negative | 34.09 | 4.85 | 3.48 | 7.03 | negative | 13 |  |  |
| 553 | female | 70 | negative | 124.92 | 5.27 | 3.43 | 23.70 | negative | 13 |  |  |
| 554 | male | 69 | negative | 256.50 | 16.79 | 32.65 | 15.28 | negative | 15 |  |  |
| 555 | male | 70 | positive | 128.29 | 12.21 | 2.76 | 10.51 | negative | 18 |  |  |
| 556 | female | 69 | positive | 115.50 | 19.44 | 48.07 | 5.94 | negative | 12 |  |  |
| 557 | male | 70 | negative | 130.79 | 14.04 | 3.63 | 9.32 | negative | 17 |  |  |
| 558 | female | 70 | negative | 84.45 | 5.59 | 1.93 | 15.11 | negative | 13 |  |  |
| 559 | male | 70 | positive | 81.32 | 8.05 | 2.80 | 10.10 | negative | 18 |  |  |
| 560 | male | 69 | negative | 131.30 | 11.48 | 3.35 | 11.44 | negative | 13 |  |  |
| 561 | male | 70 | negative | 152.71 | 10.92 | 2.94 | 13.98 | negative | 17 |  |  |
| 562 | male | 70 | negative | 114.45 | 11.01 | 2.64 | 10.40 | negative | 17 |  |  |
| 563 | male | 70 | negative | 176.65 | 16.74 | 2.33 | 10.55 | negative | 17 |  |  |
| 564 | female | 69 | negative | 17.35 | 5.17 | 60.00 | 3.36 | negative | 14 |  |  |
| 565 | male | 69 | negative | 58.64 | 3.07 | 3.87 | 19.10 | negative | 13 |  |  |
| 566 | male | 69 | negative | 117.72 | 13.24 | 4.05 | 8.89 | negative | 13 |  |  |
| 567 | male | 70 | negative | 295.66 | 35.17 | 13.93 | 8.41 | negative | 19 |  |  |
| 568 | male | 70 | negative | 55.84 | 4.90 | 3.36 | 11.40 | negative | 17 |  |  |
| 569 | male | 70 | negative | 150.90 | 45.70 | 26.80 | 3.30 | negative | 22 |  |  |
| 570 | male | 69 | positive | 97.49 | 22.33 | 5.59 | 4.37 | negative | 14 |  |  |
| 571 | male | 69 | negative | 144.46 | 12.24 | 12.05 | 11.80 | negative | 15 |  |  |
| 572 | female | 69 | positive | 147.68 | 29.73 | 6.76 | 4.97 | negative | 12 |  |  |
| 573 | male | 69 | positive | 67.70 | 16.14 | 4.25 | 4.19 | negative | 14 |  |  |
| 574 | male | 69 | negative | 251.10 | 13.24 | 2.06 | 18.97 | negative | 13 |  |  |
| 575 | male | 70 | negative | 88.95 | 10.33 | 2.86 | 8.61 | negative | 17 |  |  |
| 576 | male | 69 | positive | 170.83 | 17.29 | 8.44 | 9.88 | negative | 16 |  |  |
| 577 | male | 69 | negative | 94.22 | 7.64 | 3.06 | 12.33 | negative | 13 |  |  |
| 578 | male | 69 | negative | 96.83 | 7.73 | 2.75 | 12.53 | negative | 13 |  |  |
| 579 | male | 69 | negative | 106.11 | 9.79 | 3.49 | 10.84 | negative | 13 |  |  |
| 580 | male | 69 | negative | 148.52 | 4.57 | 2.15 | 32.50 | negative | 13 |  |  |
| 581 | male | 70 | negative | 93.82 | 6.39 | 1.55 | 14.68 | negative | 17 |  |  |
| 582 | male | 69 | positive | 187.46 | 14.57 | 9.34 | 12.87 | negative | 16 |  |  |
| 583 | male | 69 | negative | 21.58 | 8.87 | 57.87 | 2.43 | negative | 18 |  |  |
| 584 | male | 70 | negative | 162.89 | 21.80 | 2.33 | 7.47 | negative | 17 |  |  |
| 585 | female | 69 | negative | 9.09 | 3.68 | 60.00 | 2.47 | negative | 14 |  |  |
| 586 | female | 70 | negative | 107.92 | 6.39 | 2.87 | 16.89 | negative | 13 |  |  |
| 587 | male | 70 | positive | 115.98 | 14.53 | 20.76 | 7.98 | negative | 20 |  |  |
| 588 | male | 70 | negative | 104.59 | 9.51 | 0.69 | 11.00 | negative | 14 |  |  |
| 589 | male | 69 | positive | 356.14 | 25.88 | 8.60 | 13.76 | negative | 16 |  |  |
| 590 | male | 70 | positive | 120.08 | 13.44 | 25.49 | 8.93 | negative | 20 |  |  |
| 591 | female | 70 | negative | 138.92 | 6.90 | 2.08 | 20.13 | negative | 13 |  |  |
| 592 | male | 69 | positive | 110.10 | 8.54 | 6.73 | 12.89 | negative | 16 |  |  |
| 593 | male | 70 | negative | 161.90 | 8.55 | 1.62 | 18.94 | negative | 17 |  |  |
| 594 | female | 69 | positive | 170.05 | 14.77 | 5.82 | 11.51 | negative | 12 |  |  |
| 595 | male | 70 | negative | 104.77 | 6.47 | 1.39 | 16.19 | negative | 14 |  |  |
| 596 | male | 69 | negative | 183.44 | 11.39 | 5.96 | 16.11 | negative | 15 |  |  |
| 597 | male | 69 | positive | 167.44 | 11.86 | 4.75 | 14.12 | negative | 14 |  |  |
| 598 | male | 69 | negative | 98.78 | 9.70 | 6.37 | 10.18 | negative | 15 |  |  |
| 599 | male | 69 | negative | 118.48 | 7.74 | 2.01 | 15.31 | negative | 13 |  |  |
| 600 | male | 69 | positive | 66.19 | 15.69 | 40.54 | 4.22 | negative | 16 |  |  |
| 601 | male | 69 | negative | 168.43 | 9.07 | 3.15 | 18.57 | negative | 13 |  |  |
| 602 | male | 69 | negative | 152.05 | 11.61 | 3.50 | 13.10 | negative | 13 |  |  |
| 603 | male | 69 | positive | 120.90 | 12.80 | 2.70 | 9.45 | negative | 14 |  |  |
| 604 | male | 69 | negative | 113.48 | 10.29 | 3.05 | 11.03 | negative | 13 |  |  |
| 605 | male | 69 | positive | 285.35 | 12.34 | 2.31 | 23.12 | negative | 14 |  |  |
| 606 | male | 69 | positive | 123.40 | 17.27 | 4.80 | 7.15 | negative | 14 |  |  |
| 607 | male | 69 | negative | 96.32 | 16.43 | 5.02 | 5.86 | negative | 13 |  |  |
| 608 | male | 69 | negative | 53.88 | 4.38 | 9.93 | 12.30 | negative | 15 |  |  |
| 609 | female | 70 | positive | 94.15 | 14.31 | 2.42 | 6.58 | negative | 14 |  |  |
| 610 | male | 70 | negative | 171.01 | 13.30 | 2.50 | 12.86 | negative | 17 |  |  |
| 611 | female | 70 | negative | 124.71 | 6.73 | 1.98 | 18.53 | negative | 13 |  |  |
| 612 | male | 70 | positive | 122.95 | 20.05 | 1.14 | 6.13 | negative | 15 |  |  |
| 613 | female | 69 | positive | 103.07 | 10.47 | 13.61 | 9.84 | negative | 12 |  |  |
| 614 | male | 69 | negative | 149.56 | 10.62 | 4.03 | 14.08 | negative | 13 |  |  |
| 615 | male | 69 | negative | 113.02 | 8.53 | 2.24 | 13.25 | negative | 13 |  |  |
| 616 | male | 69 | negative | 64.45 | 3.23 | 5.90 | 19.95 | negative | 15 |  |  |
| 617 | male | 70 | negative | 156.23 | 8.88 | 2.43 | 17.59 | negative | 17 |  |  |
| 618 | female | 70 | negative | 162.52 | 17.17 | 3.34 | 9.47 | negative | 13 |  |  |
| 619 | male | 70 | negative | 104.29 | 15.83 | 12.70 | 6.59 | negative | 19 |  |  |
| 620 | female | 70 | negative | 63.52 | 5.02 | 2.75 | 12.65 | negative | 13 |  |  |
| 621 | male | 69 | negative | 110.31 | 6.16 | 8.91 | 17.91 | negative | 15 |  |  |
| 622 | male | 70 | negative | 114.00 | 5.85 | 2.78 | 19.49 | negative | 17 |  |  |
| 623 | male | 69 | positive | 94.01 | 23.39 | 4.88 | 4.02 | negative | 14 |  |  |
| 624 | male | 69 | negative | 111.96 | 10.68 | 2.54 | 10.48 | negative | 13 |  |  |
| 625 | male | 70 | positive | 282.56 | 45.11 | 10.93 | 6.26 | negative | 20 |  |  |
| 626 | male | 69 | negative | 86.50 | 7.43 | 4.06 | 11.64 | negative | 13 |  |  |
| 627 | female | 69 | positive | 116.23 | 18.55 | 12.38 | 6.27 | negative | 12 |  |  |
| 628 | male | 69 | negative | 72.74 | 5.80 | 2.10 | 12.54 | negative | 13 |  |  |
| 629 | male | 70 | positive | 173.14 | 16.70 | 4.28 | 10.37 | negative | 18 |  |  |
| 630 | female | 70 | positive | 144.04 | 14.09 | 2.71 | 10.22 | negative | 14 |  |  |
| 631 | female | 70 | negative | 130.38 | 11.68 | 1.91 | 11.16 | negative | 13 |  |  |
| 632 | male | 69 | negative | 181.55 | 10.62 | 2.88 | 17.10 | negative | 13 |  |  |
| 633 | male | 70 | negative | 158.64 | 19.88 | 1.05 | 7.98 | negative | 14 |  |  |
| 634 | male | 69 | positive | 118.56 | 23.73 | 7.51 | 5.00 | negative | 16 |  |  |
| 635 | female | 70 | negative | 133.41 | 15.62 | 3.79 | 8.54 | negative | 13 |  |  |
| 636 | male | 70 | negative | 99.80 | 11.00 | 2.60 | 9.07 | negative | 17 |  |  |
| 637 | male | 70 | negative | 113.23 | 8.46 | 3.47 | 13.38 | negative | 17 |  |  |
| 638 | female | 70 | negative | 82.90 | 14.60 | 3.00 | 5.68 | negative | 13 |  |  |
| 639 | male | 70 | negative | 138.06 | 13.35 | 4.18 | 10.34 | negative | 17 |  |  |
| 640 | female | 70 | negative | 104.23 | 4.67 | 1.80 | 22.32 | negative | 13 |  |  |
| 641 | male | 69 | positive | 20.48 | 9.42 | 37.83 | 2.17 | negative | 19 |  |  |
| 642 | female | 70 | positive | 160.72 | 17.08 | 3.00 | 9.41 | negative | 14 |  |  |
| 643 | male | 69 | positive | 123.31 | 29.86 | 9.58 | 4.13 | negative | 16 |  |  |
| 644 | male | 70 | negative | 76.54 | 4.34 | 12.00 | 17.64 | negative | 19 |  |  |
| 645 | male | 69 | negative | 113.51 | 14.40 | 6.74 | 7.88 | negative | 15 |  |  |
| 646 | female | 69 | positive | 121.01 | 17.22 | 5.94 | 7.03 | negative | 12 |  |  |
| 647 | male | 70 | negative | 92.10 | 8.90 | 1.10 | 10.35 | negative | 14 |  |  |
| 648 | male | 69 | positive | 138.57 | 22.98 | 9.80 | 6.03 | negative | 16 |  |  |
| 649 | male | 70 | negative | 117.31 | 10.42 | 2.82 | 11.26 | negative | 17 |  |  |
| 650 | male | 69 | positive | 158.10 | 21.53 | 7.67 | 7.34 | negative | 16 |  |  |
| 651 | male | 70 | negative | 157.99 | 15.21 | 4.21 | 10.39 | negative | 17 |  |  |
| 652 | female | 70 | negative | 55.96 | 17.49 | 26.43 | 3.20 | negative | 18 |  |  |
| 653 | male | 69 | negative | 150.89 | 15.63 | 2.71 | 9.65 | negative | 13 |  |  |
| 654 | male | 70 | negative | 71.87 | 12.63 | 1.51 | 5.69 | negative | 17 |  |  |
| 655 | male | 70 | positive | 43.80 | 19.03 | 1.17 | 2.30 | negative | 18 |  |  |
| 656 | female | 69 | positive | 142.70 | 28.80 | 20.30 | 4.95 | negative | 12 |  |  |
| 657 | female | 70 | negative | 92.87 | 7.78 | 3.06 | 11.94 | negative | 13 |  |  |
| 658 | male | 70 | positive | 215.01 | 17.39 | 18.20 | 12.36 | negative | 20 |  |  |
| 659 | male | 69 | negative | 115.67 | 9.33 | 7.89 | 12.40 | negative | 15 |  |  |
| 660 | male | 69 | negative | 73.90 | 10.80 | 15.10 | 6.84 | negative | 15 |  |  |
| 661 | male | 70 | negative | 56.92 | 8.31 | 4.21 | 6.85 | negative | 17 |  |  |
| 662 | female | 70 | negative | 121.46 | 6.06 | 2.45 | 20.04 | negative | 13 |  |  |
| 663 | female | 70 | negative | 94.73 | 6.45 | 3.59 | 14.69 | negative | 13 |  |  |
| 664 | female | 70 | negative | 89.63 | 4.98 | 2.05 | 18.00 | negative | 13 |  |  |
| 665 | male | 70 | negative | 122.16 | 10.43 | 13.84 | 11.71 | negative | 19 |  |  |
| 666 | female | 70 | negative | 209.07 | 19.31 | 10.76 | 10.83 | negative | 15 |  |  |
| 667 | male | 69 | positive | 157.78 | 26.03 | 11.73 | 6.06 | negative | 16 |  |  |
| 668 | male | 67 | negative | 61.02 | 9.63 | 2.36 | 6.34 | negative | 13 |  |  |
| 669 | female | 68 | positive | 181.61 | 28.88 | 13.71 | 6.29 | negative | 12 |  |  |
| 670 | male | 67 | negative | 143.77 | 27.67 | 5.25 | 5.20 | negative | 13 |  |  |
| 671 | male | 67 | positive | 147.28 | 14.36 | 3.70 | 10.26 | negative | 14 |  |  |
| 672 | male | 67 | negative | 108.28 | 6.78 | 3.27 | 15.97 | negative | 13 |  |  |
| 673 | male | 67 | positive | 128.23 | 13.03 | 8.22 | 9.84 | negative | 16 |  |  |
| 674 | male | 68 | negative | 174.06 | 11.95 | 3.62 | 14.57 | negative | 13 |  |  |
| 675 | male | 68 | negative | 81.70 | 10.00 | 2.60 | 8.17 | negative | 13 |  |  |
| 676 | male | 67 | negative | 100.12 | 18.74 | 3.59 | 5.34 | negative | 13 |  |  |
| 677 | female | 68 | positive | 153.99 | 20.88 | 6.19 | 7.38 | negative | 12 |  |  |
| 678 | male | 68 | negative | 150.83 | 15.82 | 4.46 | 9.53 | negative | 13 |  |  |
| 679 | female | 69 | positive | 127.20 | 31.59 | 11.23 | 4.03 | negative | 12 |  |  |
| 680 | female | 69 | positive | 119.43 | 20.48 | 11.03 | 5.83 | negative | 12 |  |  |
| 681 | male | 68 | positive | 170.95 | 17.94 | 5.21 | 9.53 | negative | 14 |  |  |
| 682 | female | 67 | positive | 128.66 | 20.63 | 9.73 | 6.24 | negative | 12 |  |  |
| 683 | male | 67 | positive | 122.70 | 13.10 | 7.30 | 9.37 | negative | 16 |  |  |
| 684 | male | 68 | positive | 167.18 | 19.58 | 7.27 | 8.54 | negative | 16 |  |  |
| 685 | male | 68 | negative | 169.73 | 10.87 | 1.66 | 15.61 | negative | 13 |  |  |
| 686 | male | 67 | positive | 70.80 | 13.10 | 7.90 | 5.40 | negative | 16 |  |  |
| 687 | male | 68 | negative | 103.34 | 8.39 | 4.96 | 12.32 | negative | 13 |  |  |
| 688 | male | 67 | negative | 93.50 | 13.50 | 3.40 | 6.93 | negative | 13 |  |  |
| 689 | male | 67 | positive | 141.30 | 27.41 | 3.95 | 5.16 | negative | 14 |  |  |
| 690 | male | 68 | negative | 151.05 | 13.22 | 4.02 | 11.43 | negative | 13 |  |  |
| 691 | male | 67 | negative | 75.13 | 5.85 | 3.05 | 12.84 | negative | 13 |  |  |
| 692 | female | 68 | positive | 150.19 | 17.79 | 10.74 | 8.44 | negative | 12 |  |  |
| 693 | male | 68 | positive | 189.52 | 13.20 | 3.41 | 14.36 | negative | 14 |  |  |
| 694 | male | 68 | negative | 57.68 | 4.97 | 1.88 | 11.61 | negative | 13 |  |  |
| 695 | male | 68 | positive | 242.51 | 19.58 | 4.64 | 12.39 | negative | 14 |  |  |
| 696 | female | 67 | positive | 55.67 | 7.40 | 25.60 | 7.52 | negative | 12 |  |  |
| 697 | male | 67 | negative | 120.39 | 9.16 | 4.81 | 13.14 | negative | 13 |  |  |
| 698 | male | 68 | negative | 137.50 | 7.45 | 3.61 | 18.46 | negative | 13 |  |  |
| 699 | male | 68 | positive | 92.04 | 19.05 | 14.65 | 4.83 | negative | 16 |  |  |
| 700 | male | 67 | negative | 356.40 | 57.60 | 4.70 | 6.19 | negative | 13 |  |  |
| 701 | male | 67 | positive | 101.16 | 11.71 | 3.16 | 8.64 | negative | 14 |  |  |
| 702 | male | 68 | positive | 242.09 | 16.84 | 6.23 | 14.38 | negative | 16 |  |  |
| 703 | male | 67 | negative | 151.68 | 8.07 | 2.34 | 18.80 | negative | 13 |  |  |
| 704 | male | 67 | negative | 249.98 | 17.11 | 6.92 | 14.61 | negative | 15 |  |  |
| 705 | male | 67 | positive | 151.71 | 20.79 | 4.28 | 7.30 | negative | 14 |  |  |
| 706 | male | 68 | positive | 112.71 | 13.69 | 7.73 | 8.23 | negative | 16 |  |  |
| 707 | female | 67 | positive | 80.59 | 12.03 | 9.89 | 6.70 | negative | 12 |  |  |
| 708 | female | 68 | positive | 192.41 | 16.87 | 8.90 | 11.41 | negative | 12 |  |  |
| 709 | male | 68 | negative | 115.94 | 7.92 | 6.11 | 14.64 | negative | 15 |  |  |
| 710 | male | 67 | positive | 76.04 | 9.67 | 7.72 | 7.86 | negative | 16 |  |  |
| 711 | male | 67 | negative | 149.89 | 12.02 | 3.01 | 12.47 | negative | 13 |  |  |
| 712 | male | 68 | negative | 151.06 | 7.93 | 1.92 | 19.05 | negative | 13 |  |  |
| 713 | female | 67 | positive | 170.15 | 17.80 | 9.42 | 9.56 | negative | 12 |  |  |
| 714 | male | 67 | negative | 118.97 | 6.87 | 3.61 | 17.32 | negative | 13 |  |  |
| 715 | female | 67 | positive | 106.20 | 18.40 | 8.60 | 5.77 | negative | 12 |  |  |
| 716 | female | 67 | positive | 66.80 | 27.68 | 7.53 | 2.41 | negative | 15 |  |  |
| 717 | male | 68 | positive | 111.45 | 12.16 | 4.65 | 9.17 | negative | 14 |  |  |
| 718 | male | 69 | negative | 119.01 | 10.62 | 1.86 | 11.21 | negative | 13 |  |  |
| 719 | male | 67 | positive | 134.95 | 15.08 | 6.65 | 8.95 | negative | 16 |  |  |
| 720 | male | 67 | positive | 134.47 | 10.31 | 3.70 | 13.04 | negative | 14 |  |  |
| 721 | male | 68 | negative | 103.22 | 10.36 | 2.74 | 9.96 | negative | 13 |  |  |
| 722 | male | 68 | positive | 124.77 | 16.34 | 4.98 | 7.64 | negative | 14 |  |  |
| 723 | male | 68 | negative | 96.92 | 9.65 | 2.46 | 10.04 | negative | 13 |  |  |
| 724 | male | 68 | negative | 136.57 | 12.66 | 2.35 | 10.79 | negative | 13 |  |  |
| 725 | male | 68 | negative | 114.95 | 9.27 | 2.20 | 12.40 | negative | 13 |  |  |
| 726 | male | 68 | negative | 289.08 | 17.44 | 8.54 | 16.58 | negative | 15 |  |  |
| 727 | male | 68 | negative | 202.34 | 8.00 | 2.36 | 25.29 | negative | 13 |  |  |
| 728 | male | 68 | negative | 129.77 | 16.12 | 2.76 | 8.05 | negative | 13 |  |  |
| 729 | male | 68 | negative | 83.80 | 8.60 | 9.00 | 9.74 | negative | 15 |  |  |
| 730 | male | 68 | negative | 123.42 | 8.76 | 1.82 | 14.09 | negative | 13 |  |  |
| 731 | male | 69 | negative | 82.27 | 7.01 | 1.59 | 11.74 | negative | 13 |  |  |
| 732 | female | 68 | positive | 115.10 | 12.90 | 9.80 | 8.92 | negative | 12 |  |  |
| 733 | female | 67 | positive | 46.14 | 9.94 | 51.68 | 4.64 | negative | 12 |  |  |
| 734 | female | 68 | positive | 251.41 | 33.70 | 16.35 | 7.46 | negative | 12 |  |  |
| 735 | male | 68 | negative | 121.50 | 16.06 | 2.94 | 7.57 | negative | 13 |  |  |
| 736 | male | 68 | negative | 101.71 | 9.58 | 2.79 | 10.62 | negative | 13 |  |  |
| 737 | male | 67 | negative | 110.31 | 15.19 | 3.00 | 7.26 | negative | 13 |  |  |
| 738 | male | 67 | negative | 153.51 | 10.54 | 4.44 | 14.56 | negative | 13 |  |  |
| 739 | male | 69 | negative | 141.26 | 13.65 | 1.63 | 10.35 | negative | 13 |  |  |
| 740 | male | 67 | positive | 168.63 | 15.39 | 4.35 | 10.96 | negative | 14 |  |  |
| 741 | male | 67 | positive | 300.00 | 51.93 | 5.54 | 5.78 | negative | 14 |  |  |
| 742 | male | 68 | positive | 100.49 | 24.57 | 3.64 | 4.09 | negative | 14 |  |  |
| 743 | male | 68 | negative | 84.40 | 4.50 | 2.61 | 18.76 | negative | 13 |  |  |
| 744 | male | 68 | negative | 62.42 | 5.68 | 3.93 | 10.99 | negative | 13 |  |  |
| 745 | male | 68 | negative | 101.85 | 15.74 | 3.98 | 6.47 | negative | 13 |  |  |
| 746 | male | 68 | positive | 146.91 | 14.09 | 6.54 | 10.43 | negative | 16 |  |  |
| 747 | male | 67 | negative | 135.61 | 12.93 | 3.93 | 10.49 | negative | 13 |  |  |
| 748 | male | 68 | positive | 206.72 | 20.99 | 3.46 | 9.85 | negative | 14 |  |  |
| 749 | male | 68 | positive | 197.33 | 29.66 | 21.41 | 6.65 | negative | 16 |  |  |
| 750 | male | 67 | negative | 81.86 | 18.39 | 6.07 | 4.45 | negative | 15 |  |  |
| 751 | female | 68 | positive | 150.43 | 19.19 | 47.46 | 7.84 | negative | 12 |  |  |
| 752 | male | 68 | positive | 146.38 | 19.04 | 8.09 | 7.69 | negative | 16 |  |  |
| 753 | male | 67 | positive | 278.55 | 19.32 | 4.15 | 14.42 | negative | 14 |  |  |
| 754 | male | 68 | positive | 278.35 | 31.03 | 16.38 | 8.97 | negative | 16 |  |  |
| 755 | male | 67 | negative | 202.42 | 22.07 | 4.79 | 9.17 | negative | 13 |  |  |
| 756 | male | 67 | positive | 294.38 | 27.42 | 7.79 | 10.74 | negative | 16 |  |  |
| 757 | male | 67 | negative | 144.10 | 14.70 | 2.40 | 9.80 | negative | 13 |  |  |
| 758 | male | 68 | positive | 285.78 | 16.48 | 4.00 | 17.34 | negative | 14 |  |  |
| 759 | male | 68 | positive | 136.15 | 17.93 | 7.30 | 7.59 | negative | 16 |  |  |
| 760 | male | 68 | negative | 119.40 | 10.38 | 6.14 | 11.50 | negative | 15 |  |  |
| 761 | male | 68 | positive | 125.54 | 19.17 | 44.76 | 6.55 | negative | 16 |  |  |
| 762 | female | 67 | positive | 179.70 | 11.92 | 6.78 | 15.08 | negative | 12 |  |  |
| 763 | male | 68 | negative | 138.54 | 13.34 | 9.66 | 10.39 | negative | 15 |  |  |
| 764 | male | 68 | negative | 46.20 | 15.10 | 8.00 | 3.06 | negative | 18 |  |  |
| 765 | female | 67 | positive | 111.94 | 8.19 | 6.19 | 13.67 | negative | 12 |  |  |
| 766 | male | 68 | negative | 106.16 | 6.00 | 3.70 | 17.69 | negative | 13 |  |  |
| 767 | male | 67 | negative | 87.06 | 10.95 | 2.28 | 7.95 | negative | 13 |  |  |
| 768 | female | 67 | positive | 134.25 | 20.53 | 5.79 | 6.54 | negative | 12 |  |  |
| 769 | male | 68 | negative | 103.93 | 10.72 | 2.61 | 9.69 | negative | 13 |  |  |
| 770 | male | 68 | negative | 99.67 | 7.63 | 2.42 | 13.06 | negative | 13 |  |  |
| 771 | male | 67 | negative | 87.86 | 4.80 | 2.50 | 18.30 | negative | 13 |  |  |
| 772 | male | 67 | negative | 102.40 | 11.10 | 3.30 | 9.23 | negative | 13 |  |  |
| 773 | female | 67 | positive | 195.64 | 20.53 | 8.62 | 9.53 | negative | 12 |  |  |
| 774 | male | 68 | positive | 71.80 | 16.60 | 3.10 | 4.33 | negative | 14 |  |  |
| 775 | male | 67 | negative | 125.41 | 10.97 | 2.33 | 11.43 | negative | 13 |  |  |
| 776 | male | 68 | negative | 76.60 | 3.01 | 2.88 | 25.45 | negative | 13 |  |  |
| 777 | male | 69 | negative | 300.00 | 23.02 | 1.58 | 13.03 | negative | 13 |  |  |
| 778 | male | 68 | negative | 184.47 | 21.50 | 4.46 | 8.58 | negative | 13 |  |  |
| 779 | male | 68 | positive | 95.94 | 18.02 | 12.37 | 5.32 | negative | 16 |  |  |
| 780 | female | 68 | positive | 112.02 | 20.72 | 11.86 | 5.41 | negative | 12 |  |  |
| 781 | male | 67 | negative | 100.53 | 5.12 | 2.44 | 19.63 | negative | 13 |  |  |
| 782 | male | 68 | negative | 183.51 | 11.76 | 2.87 | 15.60 | negative | 13 |  |  |
| 783 | male | 67 | negative | 91.82 | 10.23 | 3.16 | 8.98 | negative | 13 |  |  |
| 784 | male | 68 | positive | 132.84 | 25.12 | 2.91 | 5.29 | negative | 14 |  |  |
| 785 | male | 67 | negative | 98.20 | 13.00 | 3.20 | 7.55 | negative | 13 |  |  |
| 786 | male | 67 | positive | 45.60 | 7.10 | 3.79 | 6.42 | negative | 14 |  |  |
| 787 | male | 67 | negative | 102.90 | 8.78 | 4.20 | 11.72 | negative | 13 |  |  |
| 788 | male | 68 | positive | 227.77 | 24.50 | 3.54 | 9.30 | negative | 14 |  |  |
| 789 | male | 67 | positive | 211.67 | 17.75 | 46.60 | 11.93 | negative | 16 |  |  |
| 790 | male | 68 | positive | 91.81 | 8.70 | 4.47 | 10.55 | negative | 14 |  |  |
| 791 | female | 69 | positive | 231.70 | 25.79 | 10.18 | 8.98 | negative | 12 |  |  |
| 792 | male | 67 | positive | 94.29 | 19.76 | 4.70 | 4.77 | negative | 14 |  |  |
| 793 | female | 67 | positive | 102.70 | 14.28 | 20.74 | 7.19 | negative | 12 |  |  |
| 794 | female | 68 | positive | 114.67 | 11.81 | 9.71 | 9.71 | negative | 12 |  |  |
| 795 | male | 67 | positive | 120.96 | 19.23 | 4.19 | 6.29 | negative | 14 |  |  |
| 796 | male | 68 | negative | 292.32 | 21.83 | 6.12 | 13.39 | negative | 15 |  |  |
| 797 | male | 67 | negative | 78.80 | 5.90 | 3.30 | 13.36 | negative | 13 |  |  |
| 798 | female | 68 | negative | 20.08 | 8.18 | 60.00 | 2.45 | negative | 14 |  |  |
| 799 | male | 67 | negative | 229.86 | 21.31 | 7.87 | 10.79 | negative | 15 |  |  |
| 800 | male | 68 | positive | 63.91 | 10.34 | 5.80 | 6.18 | negative | 16 |  |  |
| 801 | male | 68 | negative | 216.94 | 8.78 | 3.41 | 24.71 | negative | 13 |  |  |
| 802 | female | 67 | negative | 58.78 | 19.72 | 60.00 | 2.98 | negative | 14 |  |  |
| 803 | male | 69 | negative | 72.20 | 7.02 | 1.60 | 10.28 | negative | 13 |  |  |
| 804 | male | 68 | negative | 130.04 | 13.73 | 9.44 | 9.47 | negative | 15 |  |  |
| 805 | male | 67 | negative | 102.30 | 20.60 | 4.50 | 4.97 | negative | 13 |  |  |
| 806 | male | 67 | negative | 174.41 | 9.46 | 3.95 | 18.44 | negative | 13 |  |  |
| 807 | male | 68 | negative | 93.25 | 8.33 | 2.88 | 11.19 | negative | 13 |  |  |
| 808 | male | 67 | negative | 233.86 | 20.40 | 8.34 | 11.46 | negative | 15 |  |  |
| 809 | male | 68 | negative | 57.27 | 10.09 | 1.96 | 5.68 | negative | 13 |  |  |
| 810 | male | 69 | positive | 216.05 | 21.42 | 11.17 | 10.09 | negative | 16 |  |  |
| 811 | female | 68 | positive | 287.96 | 36.62 | 9.09 | 7.86 | negative | 12 |  |  |
| 812 | male | 68 | positive | 105.31 | 15.83 | 17.83 | 6.65 | negative | 16 |  |  |
| 813 | male | 68 | negative | 97.61 | 10.33 | 8.72 | 9.45 | negative | 15 |  |  |
| 814 | female | 68 | positive | 90.12 | 10.94 | 11.97 | 8.24 | negative | 12 |  |  |
| 815 | male | 68 | positive | 177.90 | 17.05 | 3.58 | 10.43 | negative | 14 |  |  |
| 816 | male | 67 | negative | 85.99 | 7.24 | 4.50 | 11.88 | negative | 13 |  |  |
| 817 | male | 68 | negative | 214.08 | 15.42 | 7.35 | 13.88 | negative | 15 |  |  |
| 818 | male | 68 | negative | 130.40 | 12.15 | 4.90 | 10.73 | negative | 13 |  |  |
| 819 | male | 68 | negative | 284.12 | 9.55 | 24.26 | 29.75 | negative | 15 |  |  |
| 820 | male | 68 | positive | 132.82 | 14.05 | 5.35 | 9.45 | negative | 14 |  |  |
| 821 | male | 68 | negative | 105.53 | 10.37 | 3.74 | 10.18 | negative | 13 |  |  |
| 822 | female | 68 | negative | 10.32 | 7.68 | 60.00 | 1.34 | negative | 14 |  |  |
| 823 | male | 68 | positive | 77.27 | 7.97 | 7.19 | 9.70 | negative | 16 |  |  |
| 824 | male | 67 | negative | 21.90 | 6.85 | 60.00 | 3.20 | negative | 18 |  |  |
| 825 | male | 67 | negative | 209.83 | 21.21 | 5.79 | 9.89 | negative | 15 |  |  |
| 826 | male | 68 | negative | 287.63 | 22.52 | 16.86 | 12.77 | negative | 15 |  |  |
| 827 | male | 67 | negative | 140.98 | 11.46 | 4.00 | 12.30 | negative | 13 |  |  |
| 828 | male | 67 | positive | 101.00 | 19.20 | 7.00 | 5.26 | negative | 16 |  |  |
| 829 | male | 67 | negative | 96.91 | 5.11 | 4.82 | 18.96 | negative | 13 |  |  |
| 830 | male | 67 | positive | 110.87 | 7.97 | 2.72 | 13.91 | negative | 14 |  |  |
| 831 | female | 66 | negative | 12.37 | 5.72 | 13.36 | 2.16 | negative | 14 |  |  |
| 832 | male | 67 | negative | 70.11 | 8.98 | 2.21 | 7.81 | negative | 13 |  |  |
| 833 | male | 66 | positive | 98.80 | 23.80 | 3.00 | 4.15 | negative | 14 |  |  |
| 834 | male | 66 | negative | 115.32 | 13.58 | 5.80 | 8.49 | negative | 15 |  |  |
| 835 | female | 66 | positive | 128.85 | 14.18 | 9.76 | 9.09 | negative | 12 |  |  |
| 836 | male | 67 | negative | 170.38 | 27.09 | 16.45 | 6.29 | negative | 15 |  |  |
| 837 | male | 66 | negative | 135.52 | 10.73 | 2.26 | 12.63 | negative | 13 |  |  |
| 838 | male | 67 | positive | 80.96 | 10.96 | 14.07 | 7.39 | negative | 16 |  |  |
| 839 | male | 66 | positive | 159.70 | 19.37 | 5.26 | 8.24 | negative | 14 |  |  |
| 840 | male | 67 | negative | 62.04 | 12.74 | 1.73 | 4.87 | negative | 13 |  |  |
| 841 | male | 66 | positive | 198.51 | 14.10 | 15.47 | 14.08 | negative | 16 |  |  |
| 842 | male | 66 | negative | 118.94 | 11.61 | 6.34 | 10.24 | negative | 15 |  |  |
| 843 | female | 67 | positive | 65.45 | 12.36 | 12.32 | 5.30 | negative | 12 |  |  |
| 844 | male | 67 | negative | 92.98 | 5.28 | 1.78 | 17.61 | negative | 13 |  |  |
| 845 | male | 66 | negative | 300.00 | 17.62 | 18.31 | 17.03 | negative | 15 |  |  |
| 846 | male | 66 | negative | 121.46 | 11.13 | 2.44 | 10.91 | negative | 13 |  |  |
| 847 | male | 66 | negative | 277.94 | 21.82 | 12.66 | 12.74 | negative | 15 |  |  |
| 848 | male | 67 | negative | 68.19 | 5.01 | 1.52 | 13.61 | negative | 13 |  |  |
| 849 | male | 66 | negative | 90.95 | 8.26 | 2.42 | 11.01 | negative | 13 |  |  |
| 850 | male | 66 | negative | 67.04 | 8.27 | 3.81 | 8.11 | negative | 13 |  |  |
| 851 | male | 66 | positive | 165.95 | 20.42 | 5.80 | 8.13 | negative | 16 |  |  |
| 852 | male | 66 | positive | 164.67 | 17.76 | 8.89 | 9.27 | negative | 16 |  |  |
| 853 | male | 66 | negative | 94.96 | 8.48 | 2.00 | 11.20 | negative | 13 |  |  |
| 854 | female | 66 | positive | 106.49 | 14.52 | 14.30 | 7.33 | negative | 12 |  |  |
| 855 | male | 66 | positive | 100.57 | 28.44 | 52.35 | 3.54 | negative | 19 |  |  |
| 856 | male | 66 | negative | 45.00 | 10.80 | 2.81 | 4.17 | negative | 13 |  |  |
| 857 | male | 66 | positive | 116.82 | 13.43 | 7.44 | 8.70 | negative | 16 |  |  |
| 858 | male | 66 | negative | 203.54 | 12.16 | 3.97 | 16.74 | negative | 13 |  |  |
| 859 | male | 67 | positive | 244.42 | 23.32 | 10.29 | 10.48 | negative | 16 |  |  |
| 860 | female | 66 | positive | 90.05 | 15.33 | 8.40 | 5.87 | negative | 12 |  |  |
| 861 | male | 66 | positive | 117.09 | 10.87 | 4.69 | 10.77 | negative | 14 |  |  |
| 862 | male | 67 | negative | 107.78 | 7.22 | 2.12 | 14.93 | negative | 13 |  |  |
| 863 | male | 66 | positive | 138.44 | 21.48 | 3.74 | 6.45 | negative | 14 |  |  |
| 864 | male | 66 | negative | 117.58 | 9.03 | 5.32 | 13.02 | negative | 13 |  |  |
| 865 | female | 66 | positive | 99.85 | 28.05 | 3.04 | 3.56 | negative | 13 |  |  |
| 866 | male | 67 | negative | 124.55 | 5.67 | 1.76 | 21.97 | negative | 13 |  |  |
| 867 | female | 66 | negative | 72.42 | 27.86 | 50.37 | 2.60 | negative | 14 |  |  |
| 868 | male | 66 | negative | 285.32 | 32.28 | 11.56 | 8.84 | negative | 15 |  |  |
| 869 | male | 66 | negative | 120.08 | 6.06 | 2.43 | 19.82 | negative | 13 |  |  |
| 870 | male | 66 | negative | 142.56 | 13.98 | 2.32 | 10.20 | negative | 13 |  |  |
| 871 | male | 66 | negative | 61.80 | 3.48 | 3.63 | 17.76 | negative | 13 |  |  |
| 872 | male | 66 | positive | 238.38 | 12.48 | 13.03 | 19.10 | negative | 16 |  |  |
| 873 | male | 67 | negative | 277.38 | 31.34 | 14.47 | 8.85 | negative | 15 |  |  |
| 874 | male | 66 | negative | 235.96 | 13.06 | 4.30 | 18.07 | negative | 13 |  |  |
| 875 | male | 66 | negative | 288.78 | 24.75 | 4.17 | 11.67 | negative | 13 |  |  |
| 876 | male | 67 | negative | 199.09 | 21.68 | 2.03 | 9.18 | negative | 13 |  |  |
| 877 | male | 66 | positive | 156.26 | 14.91 | 6.00 | 10.48 | negative | 16 |  |  |
| 878 | male | 66 | positive | 260.02 | 15.02 | 6.75 | 17.31 | negative | 16 |  |  |
| 879 | male | 66 | negative | 117.93 | 14.49 | 2.92 | 8.14 | negative | 13 |  |  |
| 880 | male | 66 | negative | 173.88 | 9.00 | 3.60 | 19.32 | negative | 13 |  |  |
| 881 | male | 67 | positive | 130.48 | 12.61 | 12.70 | 10.35 | negative | 16 |  |  |
| 882 | male | 66 | positive | 99.50 | 18.65 | 11.78 | 5.34 | negative | 16 |  |  |
| 883 | male | 66 | negative | 159.49 | 14.08 | 3.58 | 11.33 | negative | 13 |  |  |
| 884 | male | 67 | negative | 129.60 | 8.61 | 2.14 | 15.05 | negative | 13 |  |  |
| 885 | male | 66 | negative | 90.99 | 8.98 | 5.10 | 10.13 | negative | 13 |  |  |
| 886 | male | 66 | negative | 126.65 | 11.19 | 7.73 | 11.32 | negative | 15 |  |  |
| 887 | male | 66 | negative | 233.83 | 20.15 | 20.75 | 11.60 | negative | 15 |  |  |
| 888 | male | 66 | negative | 40.62 | 2.64 | 24.62 | 15.39 | negative | 15 |  |  |
| 889 | male | 66 | positive | 89.05 | 14.13 | 7.83 | 6.30 | negative | 16 |  |  |
| 890 | male | 67 | negative | 136.88 | 12.68 | 2.03 | 10.79 | negative | 13 |  |  |
| 891 | male | 66 | positive | 229.80 | 19.47 | 4.53 | 11.80 | negative | 14 |  |  |
| 892 | male | 67 | negative | 76.62 | 7.12 | 2.25 | 10.76 | negative | 13 |  |  |
| 893 | male | 66 | positive | 183.49 | 31.84 | 14.58 | 5.76 | negative | 16 |  |  |
| 894 | male | 66 | negative | 93.38 | 9.35 | 4.53 | 9.99 | negative | 13 |  |  |
| 895 | female | 66 | positive | 234.83 | 16.41 | 14.55 | 14.31 | negative | 12 |  |  |
| 896 | male | 67 | positive | 103.00 | 17.40 | 11.30 | 5.92 | negative | 16 |  |  |
| 897 | male | 67 | negative | 26.28 | 5.26 | 1.76 | 5.00 | negative | 13 |  |  |
| 898 | male | 67 | negative | 138.18 | 25.17 | 13.57 | 5.49 | negative | 15 |  |  |
| 899 | male | 66 | positive | 178.08 | 11.87 | 7.86 | 15.00 | negative | 16 |  |  |
| 900 | male | 66 | positive | 138.60 | 28.50 | 9.80 | 4.86 | negative | 16 |  |  |
| 901 | male | 66 | positive | 56.12 | 14.44 | 17.68 | 3.89 | negative | 16 |  |  |
| 902 | male | 66 | negative | 159.83 | 12.36 | 7.80 | 12.93 | negative | 15 |  |  |
| 903 | male | 66 | negative | 111.70 | 13.78 | 4.82 | 8.11 | negative | 13 |  |  |
| 904 | male | 66 | negative | 115.41 | 7.40 | 7.55 | 15.60 | negative | 15 |  |  |
| 905 | male | 66 | positive | 139.30 | 19.90 | 6.40 | 7.00 | negative | 16 |  |  |
| 906 | female | 66 | positive | 74.77 | 14.31 | 9.44 | 5.23 | negative | 12 |  |  |
| 907 | male | 67 | positive | 70.07 | 11.62 | 1.97 | 6.03 | negative | 14 |  |  |
| 908 | male | 67 | positive | 121.93 | 17.08 | 12.74 | 7.14 | negative | 16 |  |  |
| 909 | male | 66 | negative | 101.86 | 11.72 | 3.87 | 8.69 | negative | 13 |  |  |
| 910 | male | 66 | positive | 239.88 | 16.69 | 13.40 | 14.37 | negative | 16 |  |  |
| 911 | female | 66 | positive | 62.17 | 18.41 | 20.73 | 3.38 | negative | 15 |  |  |
| 912 | male | 66 | negative | 174.49 | 9.51 | 3.78 | 18.35 | negative | 13 |  |  |
| 913 | male | 67 | negative | 145.30 | 10.78 | 2.25 | 13.48 | negative | 13 |  |  |
| 914 | female | 66 | positive | 67.59 | 9.06 | 20.70 | 7.46 | negative | 12 |  |  |
| 915 | male | 66 | negative | 129.13 | 8.02 | 3.32 | 16.10 | negative | 13 |  |  |
| 916 | male | 67 | negative | 96.51 | 7.50 | 1.90 | 12.87 | negative | 13 |  |  |
| 917 | male | 66 | negative | 61.94 | 5.49 | 4.64 | 11.28 | negative | 13 |  |  |
| 918 | male | 67 | negative | 56.16 | 10.37 | 17.48 | 5.42 | negative | 15 |  |  |
| 919 | male | 67 | negative | 157.02 | 17.75 | 1.72 | 8.85 | negative | 13 |  |  |
| 920 | female | 66 | positive | 222.29 | 19.52 | 6.66 | 11.39 | negative | 12 |  |  |
| 921 | male | 66 | positive | 152.19 | 21.46 | 6.51 | 7.09 | negative | 16 |  |  |
| 922 | female | 66 | negative | 69.26 | 29.42 | 24.45 | 2.35 | negative | 14 |  |  |
| 923 | female | 67 | positive | 151.48 | 18.23 | 11.10 | 8.31 | negative | 12 |  |  |
| 924 | female | 66 | positive | 156.65 | 15.85 | 6.95 | 9.88 | negative | 12 |  |  |
| 925 | male | 66 | negative | 179.74 | 16.54 | 2.19 | 10.87 | negative | 13 |  |  |
| 926 | male | 66 | negative | 70.55 | 8.58 | 2.93 | 8.22 | negative | 13 |  |  |
| 927 | male | 66 | positive | 157.14 | 13.28 | 6.30 | 11.83 | negative | 16 |  |  |
| 928 | male | 67 | negative | 84.80 | 8.04 | 1.77 | 10.55 | negative | 13 |  |  |
| 929 | male | 66 | positive | 188.68 | 30.84 | 9.95 | 6.12 | negative | 16 |  |  |
| 930 | male | 66 | negative | 139.75 | 13.06 | 3.15 | 10.70 | negative | 13 |  |  |
| 931 | female | 66 | positive | 135.61 | 23.29 | 9.13 | 5.82 | negative | 12 |  |  |
| 932 | male | 66 | positive | 86.46 | 16.76 | 2.63 | 5.16 | negative | 14 |  |  |
| 933 | male | 66 | positive | 46.86 | 14.05 | 3.77 | 3.34 | negative | 17 |  |  |
| 934 | female | 67 | positive | 126.88 | 14.51 | 13.71 | 8.74 | negative | 12 |  |  |
| 935 | male | 66 | negative | 111.12 | 6.53 | 2.52 | 17.02 | negative | 13 |  |  |
| 936 | female | 66 | positive | 217.69 | 39.40 | 57.31 | 5.53 | negative | 12 |  |  |
| 937 | male | 66 | negative | 91.11 | 6.62 | 2.99 | 13.76 | negative | 13 |  |  |
| 938 | male | 66 | positive | 152.17 | 27.87 | 3.02 | 5.46 | negative | 14 |  |  |
| 939 | male | 67 | positive | 79.21 | 16.72 | 2.22 | 4.74 | negative | 14 |  |  |
| 940 | male | 66 | negative | 191.49 | 18.96 | 5.63 | 10.10 | negative | 13 |  |  |
| 941 | male | 66 | positive | 123.14 | 21.77 | 4.09 | 5.66 | negative | 14 |  |  |
| 942 | male | 66 | positive | 209.62 | 17.57 | 6.95 | 11.93 | negative | 16 |  |  |
| 943 | female | 66 | positive | 147.94 | 19.15 | 12.57 | 7.73 | negative | 12 |  |  |
| 944 | female | 66 | positive | 273.93 | 18.38 | 8.72 | 14.90 | negative | 12 |  |  |
| 945 | male | 66 | positive | 98.10 | 26.02 | 2.01 | 3.77 | negative | 17 |  |  |
| 946 | male | 66 | negative | 145.65 | 13.51 | 2.68 | 10.78 | negative | 13 |  |  |
| 947 | male | 66 | negative | 101.41 | 9.76 | 5.09 | 10.39 | negative | 13 |  |  |
| 948 | male | 67 | negative | 74.62 | 3.25 | 1.61 | 22.96 | negative | 13 |  |  |
| 949 | male | 66 | negative | 101.26 | 9.77 | 5.02 | 10.36 | negative | 13 |  |  |
| 950 | male | 66 | negative | 32.70 | 4.60 | 25.10 | 7.11 | negative | 15 |  |  |
| 951 | male | 66 | negative | 175.63 | 19.50 | 6.11 | 9.01 | negative | 15 |  |  |
| 952 | female | 66 | positive | 110.65 | 10.93 | 6.27 | 10.12 | negative | 12 |  |  |
| 953 | male | 66 | positive | 138.39 | 20.84 | 3.50 | 6.64 | negative | 14 |  |  |
| 954 | female | 66 | positive | 109.74 | 17.60 | 7.22 | 6.24 | negative | 12 |  |  |
| 955 | male | 66 | positive | 52.06 | 8.96 | 8.38 | 5.81 | negative | 16 |  |  |
| 956 | male | 66 | positive | 425.36 | 34.26 | 7.10 | 12.42 | negative | 16 |  |  |
| 957 | male | 67 | negative | 119.26 | 11.14 | 1.64 | 10.71 | negative | 13 |  |  |
| 958 | male | 66 | negative | 159.81 | 19.31 | 4.74 | 8.28 | negative | 13 |  |  |
| 959 | female | 66 | positive | 128.83 | 13.74 | 21.01 | 9.38 | negative | 12 |  |  |
| 960 | male | 66 | negative | 130.69 | 8.21 | 5.57 | 15.92 | negative | 13 |  |  |
| 961 | male | 66 | negative | 325.65 | 17.79 | 27.39 | 18.31 | negative | 15 |  |  |
| 962 | male | 66 | negative | 127.04 | 9.31 | 2.76 | 13.65 | negative | 13 |  |  |
| 963 | male | 66 | positive | 134.22 | 22.09 | 12.09 | 6.08 | negative | 16 |  |  |
| 964 | male | 66 | positive | 132.22 | 33.09 | 42.90 | 4.00 | negative | 16 |  |  |
| 965 | male | 66 | negative | 121.12 | 10.74 | 2.07 | 11.28 | negative | 13 |  |  |
| 966 | male | 65 | negative | 121.37 | 7.60 | 3.34 | 15.97 | negative | 13 |  |  |
| 967 | male | 65 | negative | 160.88 | 14.37 | 16.58 | 11.20 | negative | 15 |  |  |
| 968 | male | 65 | positive | 110.48 | 15.06 | 3.54 | 7.34 | negative | 14 |  |  |
| 969 | male | 65 | negative | 123.57 | 7.31 | 4.28 | 16.90 | negative | 13 |  |  |
| 970 | male | 66 | negative | 73.40 | 5.60 | 1.80 | 13.11 | negative | 13 |  |  |
| 971 | male | 65 | positive | 72.40 | 12.90 | 9.10 | 5.61 | negative | 16 |  |  |
| 972 | male | 65 | negative | 73.34 | 9.45 | 4.59 | 7.76 | negative | 13 |  |  |
| 973 | male | 65 | negative | 83.46 | 7.40 | 2.86 | 11.28 | negative | 13 |  |  |
| 974 | female | 65 | positive | 136.49 | 14.34 | 9.48 | 9.52 | negative | 12 |  |  |
| 975 | female | 65 | positive | 106.48 | 30.35 | 33.12 | 3.51 | negative | 15 |  |  |
| 976 | male | 65 | negative | 70.86 | 7.69 | 2.85 | 9.21 | negative | 13 |  |  |
| 977 | male | 65 | negative | 143.12 | 8.19 | 2.53 | 17.47 | negative | 13 |  |  |
| 978 | male | 65 | positive | 233.70 | 31.75 | 6.71 | 7.36 | negative | 16 |  |  |
| 979 | male | 65 | positive | 121.60 | 21.60 | 15.50 | 5.63 | negative | 16 |  |  |
| 980 | male | 65 | negative | 96.07 | 6.97 | 2.22 | 13.78 | negative | 13 |  |  |
| 981 | female | 65 | positive | 97.59 | 12.57 | 7.95 | 7.76 | negative | 12 |  |  |
| 982 | male | 66 | negative | 148.67 | 12.59 | 1.56 | 11.81 | negative | 13 |  |  |
| 983 | male | 65 | positive | 88.60 | 14.50 | 9.90 | 6.11 | negative | 16 |  |  |
| 984 | male | 66 | negative | 76.67 | 6.46 | 1.91 | 11.87 | negative | 13 |  |  |
| 985 | male | 65 | negative | 300.00 | 31.34 | 9.18 | 9.57 | negative | 15 |  |  |
| 986 | male | 65 | negative | 61.00 | 9.10 | 2.80 | 6.70 | negative | 13 |  |  |
| 987 | male | 65 | negative | 166.12 | 10.47 | 3.25 | 15.87 | negative | 13 |  |  |
| 988 | male | 65 | negative | 182.52 | 12.60 | 12.48 | 14.49 | negative | 15 |  |  |
| 989 | male | 65 | positive | 66.18 | 5.61 | 7.41 | 11.80 | negative | 16 |  |  |
| 990 | male | 65 | negative | 91.06 | 6.59 | 6.38 | 13.82 | negative | 15 |  |  |
| 991 | female | 66 | positive | 82.47 | 7.27 | 10.47 | 11.34 | negative | 12 |  |  |
| 992 | male | 65 | negative | 103.33 | 7.95 | 2.76 | 13.00 | negative | 13 |  |  |
| 993 | male | 65 | positive | 180.78 | 16.13 | 4.72 | 11.21 | negative | 14 |  |  |
| 994 | male | 65 | negative | 152.73 | 14.05 | 3.02 | 10.87 | negative | 13 |  |  |
| 995 | male | 65 | negative | 176.23 | 17.30 | 4.30 | 10.19 | negative | 13 |  |  |
| 996 | male | 65 | negative | 61.20 | 7.98 | 7.65 | 7.67 | negative | 15 |  |  |
| 997 | male | 65 | negative | 279.07 | 17.41 | 3.25 | 16.03 | negative | 13 |  |  |
| 998 | male | 65 | positive | 103.54 | 11.75 | 2.67 | 8.81 | negative | 14 |  |  |
| 999 | female | 65 | positive | 92.04 | 16.75 | 16.22 | 5.49 | negative | 12 |  |  |
| 1000 | male | 65 | negative | 277.82 | 18.03 | 36.59 | 15.41 | negative | 15 |  |  |
| 1001 | male | 66 | negative | 124.82 | 7.88 | 1.92 | 15.84 | negative | 13 |  |  |
| 1002 | female | 65 | positive | 151.30 | 16.25 | 6.78 | 9.31 | negative | 12 |  |  |
| 1003 | male | 65 | negative | 56.94 | 12.59 | 2.63 | 4.52 | negative | 13 |  |  |
| 1004 | male | 65 | negative | 47.88 | 28.22 | 5.16 | 1.70 | negative | 16 |  |  |
| 1005 | male | 65 | negative | 60.07 | 10.10 | 4.37 | 5.95 | negative | 13 |  |  |
| 1006 | male | 65 | positive | 158.99 | 18.05 | 3.01 | 8.81 | negative | 14 |  |  |
| 1007 | female | 65 | positive | 123.94 | 24.94 | 15.29 | 4.97 | negative | 12 |  |  |
| 1008 | male | 65 | positive | 118.85 | 10.05 | 3.89 | 11.83 | negative | 14 |  |  |
| 1009 | male | 65 | negative | 115.19 | 11.89 | 3.14 | 9.69 | negative | 13 |  |  |
| 1010 | male | 65 | negative | 155.45 | 12.13 | 3.60 | 12.82 | negative | 13 |  |  |
| 1011 | male | 65 | positive | 196.82 | 37.95 | 13.73 | 5.19 | negative | 16 |  |  |
| 1012 | male | 65 | negative | 155.25 | 5.92 | 14.31 | 26.22 | negative | 15 |  |  |
| 1013 | female | 65 | positive | 181.27 | 31.78 | 14.73 | 5.70 | negative | 12 |  |  |
| 1014 | male | 66 | negative | 91.84 | 16.63 | 10.02 | 5.52 | negative | 15 |  |  |
| 1015 | male | 66 | negative | 122.35 | 9.11 | 1.93 | 13.43 | negative | 13 |  |  |
| 1016 | male | 65 | positive | 81.90 | 14.90 | 4.70 | 5.50 | negative | 14 |  |  |
| 1017 | male | 65 | negative | 91.00 | 9.10 | 3.70 | 10.00 | negative | 13 |  |  |
| 1018 | male | 65 | positive | 79.67 | 10.42 | 2.34 | 7.65 | negative | 14 |  |  |
| 1019 | male | 65 | positive | 112.50 | 18.50 | 8.90 | 6.08 | negative | 16 |  |  |
| 1020 | male | 65 | positive | 153.55 | 19.78 | 9.35 | 7.76 | negative | 16 |  |  |
| 1021 | female | 65 | negative | 29.44 | 11.53 | 19.77 | 2.55 | negative | 14 |  |  |
| 1022 | male | 65 | positive | 103.11 | 12.91 | 36.89 | 7.99 | negative | 16 |  |  |
| 1023 | male | 65 | positive | 69.30 | 21.50 | 5.10 | 3.22 | negative | 17 |  |  |
| 1024 | male | 66 | positive | 113.75 | 15.38 | 10.71 | 7.40 | negative | 16 |  |  |
| 1025 | male | 66 | negative | 105.84 | 8.14 | 1.52 | 13.00 | negative | 13 |  |  |
| 1026 | male | 65 | negative | 130.26 | 9.62 | 2.73 | 13.54 | negative | 13 |  |  |
| 1027 | male | 65 | negative | 36.05 | 8.91 | 2.22 | 4.05 | negative | 13 |  |  |
| 1028 | male | 65 | negative | 59.02 | 9.73 | 7.70 | 6.07 | negative | 15 |  |  |
| 1029 | male | 65 | negative | 99.80 | 10.80 | 5.40 | 9.24 | negative | 13 |  |  |
| 1030 | male | 65 | negative | 297.68 | 16.40 | 58.98 | 18.15 | negative | 15 |  |  |
| 1031 | male | 65 | negative | 89.69 | 8.66 | 6.83 | 10.36 | negative | 15 |  |  |
| 1032 | female | 65 | positive | 166.70 | 25.64 | 18.78 | 6.50 | negative | 12 |  |  |
| 1033 | male | 65 | negative | 72.40 | 4.60 | 2.52 | 15.74 | negative | 13 |  |  |
| 1034 | male | 65 | positive | 80.27 | 12.30 | 5.23 | 6.53 | negative | 14 |  |  |
| 1035 | male | 65 | negative | 101.57 | 11.17 | 6.92 | 9.09 | negative | 15 |  |  |
| 1036 | male | 66 | positive | 75.88 | 9.88 | 10.67 | 7.68 | negative | 16 |  |  |
| 1037 | male | 65 | negative | 117.35 | 11.06 | 2.82 | 10.61 | negative | 13 |  |  |
| 1038 | male | 66 | negative | 63.96 | 9.98 | 10.73 | 6.41 | negative | 15 |  |  |
| 1039 | male | 65 | positive | 119.00 | 22.60 | 7.50 | 5.27 | negative | 16 |  |  |
| 1040 | female | 65 | positive | 168.50 | 23.83 | 7.87 | 7.07 | negative | 12 |  |  |
| 1041 | male | 65 | positive | 109.64 | 10.06 | 7.47 | 10.90 | negative | 16 |  |  |
| 1042 | male | 65 | positive | 155.32 | 23.54 | 4.15 | 6.60 | negative | 14 |  |  |
| 1043 | female | 65 | positive | 118.20 | 14.72 | 7.39 | 8.03 | negative | 12 |  |  |
| 1044 | male | 65 | negative | 113.20 | 14.90 | 3.40 | 7.60 | negative | 13 |  |  |
| 1045 | male | 65 | negative | 125.14 | 12.76 | 5.91 | 9.81 | negative | 15 |  |  |
| 1046 | male | 65 | negative | 256.33 | 15.85 | 4.97 | 16.17 | negative | 13 |  |  |
| 1047 | male | 66 | negative | 79.26 | 4.34 | 1.68 | 18.26 | negative | 13 |  |  |
| 1048 | female | 65 | positive | 226.20 | 30.44 | 7.26 | 7.43 | negative | 12 |  |  |
| 1049 | male | 66 | positive | 55.90 | 6.80 | 11.20 | 8.22 | negative | 16 |  |  |
| 1050 | male | 65 | positive | 66.04 | 17.11 | 2.91 | 3.86 | negative | 17 |  |  |
| 1051 | male | 65 | negative | 123.51 | 9.18 | 2.90 | 13.45 | negative | 13 |  |  |
| 1052 | male | 65 | positive | 127.00 | 13.68 | 5.57 | 9.28 | negative | 14 |  |  |
| 1053 | male | 65 | negative | 172.65 | 13.91 | 3.76 | 12.41 | negative | 13 |  |  |
| 1054 | male | 66 | positive | 215.67 | 21.31 | 11.47 | 10.12 | negative | 16 |  |  |
| 1055 | male | 65 | negative | 58.02 | 3.83 | 2.77 | 15.15 | negative | 13 |  |  |
| 1056 | male | 65 | negative | 191.29 | 14.04 | 6.63 | 13.62 | negative | 15 |  |  |
| 1057 | male | 65 | positive | 55.55 | 15.32 | 7.28 | 3.63 | negative | 19 |  |  |
| 1058 | male | 65 | positive | 102.65 | 22.75 | 2.61 | 4.51 | negative | 14 |  |  |
| 1059 | male | 65 | positive | 136.93 | 18.50 | 3.06 | 7.40 | negative | 14 |  |  |
| 1060 | male | 65 | positive | 140.93 | 18.42 | 2.05 | 7.65 | negative | 14 |  |  |
| 1061 | male | 65 | positive | 48.90 | 11.04 | 6.58 | 4.43 | negative | 16 |  |  |
| 1062 | male | 65 | negative | 114.13 | 14.33 | 4.19 | 7.96 | negative | 13 |  |  |
| 1063 | female | 65 | negative | 17.90 | 11.38 | 51.29 | 1.57 | negative | 14 |  |  |
| 1064 | male | 65 | positive | 102.25 | 45.63 | 7.75 | 2.24 | negative | 19 |  |  |
| 1065 | female | 65 | positive | 86.47 | 13.41 | 6.39 | 6.45 | negative | 12 |  |  |
| 1066 | male | 65 | negative | 147.98 | 11.40 | 4.93 | 12.98 | negative | 13 |  |  |
| 1067 | male | 65 | negative | 126.92 | 7.73 | 2.25 | 16.42 | negative | 13 |  |  |
| 1068 | male | 65 | negative | 132.92 | 5.38 | 3.53 | 24.71 | negative | 13 |  |  |
| 1069 | male | 65 | negative | 128.59 | 11.60 | 2.01 | 11.09 | negative | 13 |  |  |
| 1070 | male | 66 | positive | 182.53 | 25.37 | 10.15 | 7.19 | negative | 16 |  |  |
| 1071 | male | 65 | positive | 75.03 | 10.49 | 18.31 | 7.15 | negative | 16 |  |  |
| 1072 | male | 65 | negative | 129.99 | 7.01 | 2.31 | 18.54 | negative | 13 |  |  |
| 1073 | male | 65 | negative | 87.90 | 17.00 | 4.80 | 5.17 | negative | 13 |  |  |
| 1074 | male | 65 | negative | 124.79 | 10.56 | 3.86 | 11.82 | negative | 13 |  |  |
| 1075 | female | 65 | positive | 187.06 | 12.09 | 9.28 | 15.47 | negative | 12 |  |  |
| 1076 | female | 65 | positive | 128.81 | 12.69 | 9.19 | 10.15 | negative | 12 |  |  |
| 1077 | female | 66 | positive | 140.16 | 22.48 | 10.54 | 6.23 | negative | 12 |  |  |
| 1078 | male | 65 | positive | 154.84 | 11.55 | 2.60 | 13.41 | negative | 14 |  |  |
| 1079 | male | 65 | negative | 158.00 | 9.04 | 4.57 | 17.48 | negative | 13 |  |  |
| 1080 | male | 66 | negative | 162.00 | 9.16 | 1.59 | 17.69 | negative | 13 |  |  |
| 1081 | male | 65 | positive | 101.10 | 24.60 | 14.40 | 4.11 | negative | 16 |  |  |
| 1082 | male | 65 | positive | 118.72 | 14.06 | 4.50 | 8.44 | negative | 14 |  |  |
| 1083 | male | 65 | negative | 142.14 | 11.27 | 2.19 | 12.61 | negative | 13 |  |  |
| 1084 | male | 65 | negative | 102.15 | 18.92 | 9.28 | 5.40 | negative | 15 |  |  |
| 1085 | male | 65 | positive | 95.94 | 12.01 | 13.55 | 7.99 | negative | 16 |  |  |
| 1086 | male | 65 | positive | 63.37 | 16.85 | 13.23 | 3.76 | negative | 19 |  |  |
| 1087 | male | 65 | negative | 120.25 | 12.07 | 4.63 | 9.96 | negative | 13 |  |  |
| 1088 | male | 65 | negative | 89.08 | 10.56 | 23.01 | 8.44 | negative | 15 |  |  |
| 1089 | male | 65 | positive | 252.22 | 22.94 | 4.67 | 10.99 | negative | 14 |  |  |
| 1090 | male | 65 | positive | 98.00 | 19.00 | 7.60 | 5.16 | negative | 16 |  |  |
| 1091 | male | 63 | positive | 125.71 | 15.23 | 7.86 | 8.25 | negative | 16 |  |  |
| 1092 | male | 64 | negative | 128.50 | 9.10 | 2.60 | 14.12 | negative | 13 |  |  |
| 1093 | male | 64 | positive | 90.42 | 11.12 | 4.47 | 8.13 | negative | 14 |  |  |
| 1094 | male | 64 | negative | 16.58 | 4.69 | 57.86 | 3.54 | negative | 18 |  |  |
| 1095 | male | 64 | negative | 97.80 | 12.30 | 7.20 | 7.95 | negative | 15 |  |  |
| 1096 | male | 64 | negative | 76.12 | 8.75 | 4.54 | 8.70 | negative | 13 |  |  |
| 1097 | male | 64 | negative | 280.61 | 15.73 | 46.89 | 17.84 | negative | 15 |  |  |
| 1098 | male | 64 | negative | 40.30 | 13.40 | 44.60 | 3.01 | negative | 18 |  |  |
| 1099 | male | 65 | negative | 77.70 | 6.31 | 12.30 | 12.31 | negative | 15 |  |  |
| 1100 | male | 64 | positive | 135.02 | 11.31 | 12.33 | 11.94 | negative | 16 |  |  |
| 1101 | male | 63 | positive | 113.47 | 26.23 | 9.62 | 4.33 | negative | 16 |  |  |
| 1102 | male | 64 | positive | 199.05 | 14.86 | 3.81 | 13.40 | negative | 14 |  |  |
| 1103 | male | 65 | negative | 67.05 | 14.89 | 1.69 | 4.50 | negative | 13 |  |  |
| 1104 | male | 64 | negative | 115.09 | 9.84 | 2.61 | 11.70 | negative | 13 |  |  |
| 1105 | male | 64 | negative | 68.13 | 5.27 | 2.54 | 12.93 | negative | 13 |  |  |
| 1106 | male | 64 | negative | 64.86 | 5.40 | 2.10 | 12.01 | negative | 13 |  |  |
| 1107 | male | 64 | negative | 94.39 | 8.68 | 4.02 | 10.87 | negative | 13 |  |  |
| 1108 | male | 64 | negative | 124.80 | 10.87 | 2.00 | 11.48 | negative | 13 |  |  |
| 1109 | male | 64 | negative | 96.46 | 5.81 | 2.63 | 16.60 | negative | 13 |  |  |
| 1110 | female | 64 | negative | 66.47 | 18.06 | 7.33 | 3.68 | negative | 14 |  |  |
| 1111 | male | 64 | positive | 133.67 | 14.27 | 2.43 | 9.37 | negative | 14 |  |  |
| 1112 | male | 64 | negative | 178.41 | 8.21 | 1.54 | 21.73 | negative | 13 |  |  |
| 1113 | male | 64 | negative | 81.05 | 8.20 | 9.20 | 9.88 | negative | 15 |  |  |
| 1114 | male | 64 | negative | 146.45 | 12.19 | 4.43 | 12.01 | negative | 13 |  |  |
| 1115 | male | 63 | positive | 88.50 | 18.10 | 7.80 | 4.89 | negative | 16 |  |  |
| 1116 | female | 64 | positive | 295.60 | 63.50 | 14.96 | 4.66 | negative | 12 |  |  |
| 1117 | male | 64 | negative | 110.44 | 9.59 | 3.80 | 11.52 | negative | 13 |  |  |
| 1118 | male | 64 | negative | 296.21 | 15.76 | 10.90 | 18.80 | negative | 15 |  |  |
| 1119 | male | 64 | negative | 127.26 | 11.51 | 17.49 | 11.06 | negative | 15 |  |  |
| 1120 | female | 64 | negative | 13.85 | 5.42 | 57.35 | 2.56 | negative | 14 |  |  |
| 1121 | male | 64 | positive | 41.53 | 31.60 | 10.67 | 1.31 | negative | 19 |  |  |
| 1122 | male | 64 | negative | 171.72 | 15.13 | 9.43 | 11.35 | negative | 15 |  |  |
| 1123 | male | 64 | positive | 133.87 | 17.98 | 8.75 | 7.45 | negative | 16 |  |  |
| 1124 | male | 64 | negative | 111.39 | 4.06 | 2.79 | 27.44 | negative | 13 |  |  |
| 1125 | male | 65 | positive | 142.39 | 18.16 | 11.81 | 7.84 | negative | 16 |  |  |
| 1126 | female | 64 | positive | 101.20 | 14.40 | 6.80 | 7.03 | negative | 12 |  |  |
| 1127 | male | 64 | negative | 148.40 | 8.11 | 2.82 | 18.30 | negative | 13 |  |  |
| 1128 | male | 63 | negative | 46.15 | 4.75 | 7.90 | 9.72 | negative | 15 |  |  |
| 1129 | male | 64 | negative | 280.37 | 11.19 | 19.28 | 25.06 | negative | 15 |  |  |
| 1130 | male | 64 | negative | 177.62 | 11.83 | 1.99 | 15.01 | negative | 13 |  |  |
| 1131 | male | 64 | negative | 192.97 | 14.80 | 3.72 | 13.04 | negative | 13 |  |  |
| 1132 | male | 64 | negative | 79.36 | 9.56 | 6.90 | 8.30 | negative | 15 |  |  |
| 1133 | female | 64 | positive | 219.88 | 15.29 | 12.06 | 14.38 | negative | 12 |  |  |
| 1134 | male | 64 | negative | 143.34 | 13.19 | 4.27 | 10.87 | negative | 13 |  |  |
| 1135 | male | 64 | positive | 232.42 | 14.97 | 21.05 | 15.53 | negative | 16 |  |  |
| 1136 | male | 64 | positive | 179.93 | 15.37 | 10.10 | 11.71 | negative | 16 |  |  |
| 1137 | male | 64 | positive | 182.34 | 14.63 | 2.92 | 12.46 | negative | 14 |  |  |
| 1138 | male | 64 | positive | 72.55 | 7.51 | 4.97 | 9.66 | negative | 14 |  |  |
| 1139 | male | 64 | negative | 91.65 | 6.25 | 2.37 | 14.66 | negative | 13 |  |  |
| 1140 | male | 64 | negative | 77.47 | 13.50 | 4.55 | 5.74 | negative | 13 |  |  |
| 1141 | male | 63 | negative | 130.58 | 11.67 | 8.05 | 11.19 | negative | 15 |  |  |
| 1142 | male | 64 | negative | 93.66 | 8.10 | 2.54 | 11.56 | negative | 13 |  |  |
| 1143 | male | 64 | negative | 75.12 | 10.13 | 11.40 | 7.42 | negative | 15 |  |  |
| 1144 | male | 64 | negative | 36.80 | 12.23 | 1.43 | 3.01 | negative | 13 |  |  |
| 1145 | male | 64 | positive | 83.70 | 19.60 | 14.70 | 4.27 | negative | 16 |  |  |
| 1146 | male | 63 | positive | 113.58 | 16.86 | 8.34 | 6.74 | negative | 16 |  |  |
| 1147 | male | 64 | negative | 98.94 | 22.71 | 2.28 | 4.36 | negative | 13 |  |  |
| 1148 | female | 64 | positive | 187.12 | 16.74 | 12.54 | 11.18 | negative | 12 |  |  |
| 1149 | male | 63 | negative | 138.65 | 4.26 | 9.66 | 32.55 | negative | 15 |  |  |
| 1150 | male | 65 | negative | 210.13 | 13.06 | 1.87 | 16.09 | negative | 13 |  |  |
| 1151 | male | 64 | positive | 152.15 | 13.37 | 2.42 | 11.38 | negative | 14 |  |  |
| 1152 | male | 64 | negative | 142.93 | 14.48 | 6.47 | 9.87 | negative | 15 |  |  |
| 1153 | male | 64 | negative | 111.75 | 9.80 | 1.74 | 11.40 | negative | 13 |  |  |
| 1154 | female | 64 | positive | 94.36 | 20.55 | 11.12 | 4.59 | negative | 12 |  |  |
| 1155 | female | 64 | positive | 132.10 | 27.10 | 17.90 | 4.87 | negative | 12 |  |  |
| 1156 | male | 64 | negative | 289.64 | 25.65 | 3.54 | 11.29 | negative | 13 |  |  |
| 1157 | male | 64 | negative | 127.65 | 9.21 | 4.11 | 13.86 | negative | 13 |  |  |
| 1158 | male | 64 | negative | 206.14 | 10.37 | 4.54 | 19.88 | negative | 13 |  |  |
| 1159 | male | 64 | positive | 165.40 | 13.99 | 6.04 | 11.82 | negative | 16 |  |  |
| 1160 | male | 64 | negative | 68.15 | 4.29 | 1.80 | 15.89 | negative | 13 |  |  |
| 1161 | male | 63 | positive | 117.19 | 14.11 | 8.81 | 8.31 | negative | 16 |  |  |
| 1162 | female | 64 | positive | 109.15 | 36.66 | 25.50 | 2.98 | negative | 15 |  |  |
| 1163 | male | 64 | negative | 104.34 | 5.44 | 2.78 | 19.18 | negative | 13 |  |  |
| 1164 | female | 64 | positive | 97.34 | 31.85 | 5.39 | 3.06 | negative | 13 |  |  |
| 1165 | female | 64 | positive | 152.73 | 15.53 | 13.08 | 9.83 | negative | 12 |  |  |
| 1166 | male | 64 | negative | 137.65 | 7.41 | 2.22 | 18.58 | negative | 13 |  |  |
| 1167 | female | 64 | positive | 133.70 | 27.21 | 8.82 | 4.91 | negative | 12 |  |  |
| 1168 | male | 64 | negative | 90.17 | 3.15 | 1.87 | 28.63 | negative | 13 |  |  |
| 1169 | female | 63 | positive | 95.57 | 9.75 | 9.30 | 9.80 | negative | 12 |  |  |
| 1170 | female | 64 | positive | 104.38 | 16.21 | 10.41 | 6.44 | negative | 12 |  |  |
| 1171 | male | 64 | negative | 152.65 | 23.17 | 3.26 | 6.59 | negative | 13 |  |  |
| 1172 | male | 64 | positive | 96.89 | 11.38 | 5.41 | 8.51 | negative | 14 |  |  |
| 1173 | male | 65 | negative | 117.70 | 8.58 | 1.59 | 13.72 | negative | 13 |  |  |
| 1174 | male | 64 | negative | 124.10 | 16.72 | 4.31 | 7.42 | negative | 13 |  |  |
| 1175 | female | 64 | positive | 92.60 | 11.60 | 7.00 | 7.98 | negative | 12 |  |  |
| 1176 | male | 64 | negative | 137.63 | 6.99 | 3.22 | 19.69 | negative | 13 |  |  |
| 1177 | male | 64 | positive | 94.46 | 13.76 | 2.02 | 6.86 | negative | 14 |  |  |
| 1178 | male | 64 | negative | 122.80 | 12.50 | 3.00 | 9.82 | negative | 13 |  |  |
| 1179 | female | 64 | positive | 141.15 | 24.46 | 5.88 | 5.77 | negative | 12 |  |  |
| 1180 | male | 64 | negative | 88.53 | 8.93 | 2.39 | 9.91 | negative | 13 |  |  |
| 1181 | female | 64 | positive | 108.96 | 13.58 | 5.88 | 8.02 | negative | 12 |  |  |
| 1182 | female | 64 | negative | 77.40 | 20.67 | 7.62 | 3.74 | negative | 14 |  |  |
| 1183 | female | 63 | positive | 164.36 | 18.53 | 9.70 | 8.87 | negative | 12 |  |  |
| 1184 | male | 63 | negative | 81.30 | 5.51 | 7.77 | 14.75 | negative | 15 |  |  |
| 1185 | female | 64 | positive | 117.86 | 13.39 | 9.32 | 8.80 | negative | 12 |  |  |
| 1186 | male | 65 | negative | 27.22 | 4.15 | 1.82 | 6.56 | negative | 13 |  |  |
| 1187 | male | 64 | positive | 179.10 | 18.40 | 8.40 | 9.73 | negative | 16 |  |  |
| 1188 | female | 65 | positive | 110.60 | 38.20 | 11.70 | 2.90 | negative | 15 |  |  |
| 1189 | male | 64 | negative | 96.86 | 6.10 | 1.65 | 15.88 | negative | 13 |  |  |
| 1190 | male | 64 | negative | 123.59 | 8.24 | 7.03 | 15.00 | negative | 15 |  |  |
| 1191 | female | 64 | positive | 172.78 | 23.66 | 6.64 | 7.30 | negative | 12 |  |  |
| 1192 | male | 64 | positive | 94.00 | 8.03 | 12.15 | 11.71 | negative | 16 |  |  |
| 1193 | female | 64 | positive | 156.28 | 29.55 | 14.05 | 5.29 | negative | 12 |  |  |
| 1194 | male | 64 | negative | 141.67 | 12.21 | 8.27 | 11.60 | negative | 15 |  |  |
| 1195 | male | 64 | negative | 134.40 | 10.21 | 1.90 | 13.16 | negative | 13 |  |  |
| 1196 | male | 64 | negative | 84.03 | 7.77 | 2.06 | 10.81 | negative | 13 |  |  |
| 1197 | male | 64 | negative | 285.30 | 14.41 | 5.14 | 19.80 | negative | 13 |  |  |
| 1198 | male | 64 | negative | 74.30 | 9.31 | 19.26 | 7.98 | negative | 15 |  |  |
| 1199 | male | 64 | negative | 259.93 | 13.72 | 20.62 | 18.95 | negative | 15 |  |  |
| 1200 | female | 64 | positive | 65.69 | 15.85 | 27.94 | 4.14 | negative | 12 |  |  |
| 1201 | male | 64 | positive | 291.35 | 20.20 | 19.14 | 14.42 | negative | 16 |  |  |
| 1202 | male | 64 | positive | 94.00 | 14.54 | 7.60 | 6.46 | negative | 16 |  |  |
| 1203 | male | 64 | negative | 207.54 | 14.82 | 3.48 | 14.00 | negative | 13 |  |  |
| 1204 | male | 64 | negative | 190.68 | 9.94 | 2.79 | 19.18 | negative | 13 |  |  |
| 1205 | male | 64 | negative | 154.81 | 8.63 | 2.17 | 17.94 | negative | 13 |  |  |
| 1206 | male | 64 | negative | 168.43 | 9.84 | 2.77 | 17.12 | negative | 13 |  |  |
| 1207 | male | 64 | positive | 151.31 | 15.74 | 6.32 | 9.61 | negative | 16 |  |  |
| 1208 | female | 64 | positive | 148.42 | 24.72 | 17.02 | 6.00 | negative | 12 |  |  |
| 1209 | male | 64 | negative | 74.71 | 14.24 | 5.40 | 5.25 | negative | 13 |  |  |
| 1210 | male | 64 | negative | 78.30 | 9.70 | 4.80 | 8.07 | negative | 13 |  |  |
| 1211 | male | 64 | negative | 104.78 | 6.17 | 4.77 | 16.98 | negative | 13 |  |  |
| 1212 | male | 65 | negative | 184.69 | 27.07 | 11.18 | 6.82 | negative | 15 |  |  |
| 1213 | male | 64 | negative | 105.19 | 10.80 | 2.51 | 9.74 | negative | 13 |  |  |
| 1214 | male | 63 | negative | 66.08 | 12.31 | 8.80 | 5.37 | negative | 15 |  |  |
| 1215 | female | 64 | positive | 197.77 | 25.61 | 7.36 | 7.72 | negative | 12 |  |  |
| 1216 | male | 64 | negative | 149.13 | 11.07 | 2.43 | 13.47 | negative | 13 |  |  |
| 1217 | male | 65 | negative | 90.15 | 7.86 | 1.53 | 11.47 | negative | 13 |  |  |
| 1218 | male | 64 | negative | 149.75 | 6.74 | 2.81 | 22.22 | negative | 13 |  |  |
| 1219 | female | 63 | positive | 118.80 | 16.48 | 8.62 | 7.21 | negative | 12 |  |  |
| 1220 | female | 64 | positive | 43.50 | 8.85 | 18.02 | 4.92 | negative | 12 |  |  |
| 1221 | male | 64 | negative | 21.80 | 5.88 | 0.63 | 3.71 | negative | 13 |  |  |
| 1222 | male | 64 | negative | 167.12 | 11.81 | 2.93 | 14.15 | negative | 13 |  |  |
| 1223 | male | 65 | negative | 116.78 | 6.65 | 1.86 | 17.56 | negative | 13 |  |  |
| 1224 | male | 64 | positive | 155.87 | 10.21 | 5.72 | 15.27 | negative | 16 |  |  |
| 1225 | male | 65 | negative | 113.51 | 12.15 | 1.50 | 9.34 | negative | 13 |  |  |
| 1226 | male | 65 | negative | 57.91 | 3.01 | 1.74 | 19.24 | negative | 13 |  |  |
| 1227 | male | 64 | negative | 97.79 | 19.65 | 4.02 | 4.98 | negative | 13 |  |  |
| 1228 | male | 64 | positive | 102.06 | 14.80 | 4.16 | 6.90 | negative | 14 |  |  |
| 1229 | male | 64 | negative | 130.88 | 13.15 | 5.22 | 9.95 | negative | 13 |  |  |
| 1230 | male | 64 | negative | 197.20 | 8.57 | 1.66 | 23.01 | negative | 13 |  |  |
| 1231 | male | 63 | negative | 92.77 | 8.56 | 8.60 | 10.84 | negative | 15 |  |  |
| 1232 | male | 64 | negative | 113.41 | 7.20 | 1.83 | 15.75 | negative | 13 |  |  |
| 1233 | male | 64 | positive | 110.91 | 11.60 | 21.84 | 9.56 | negative | 16 |  |  |
| 1234 | male | 64 | positive | 155.12 | 16.71 | 2.07 | 9.28 | negative | 14 |  |  |
| 1235 | male | 64 | negative | 174.86 | 13.20 | 2.79 | 13.25 | negative | 13 |  |  |
| 1236 | male | 65 | negative | 56.40 | 7.40 | 1.60 | 7.62 | negative | 13 |  |  |
| 1237 | female | 63 | positive | 133.21 | 14.60 | 8.20 | 9.12 | negative | 12 |  |  |
| 1238 | male | 64 | negative | 235.03 | 20.91 | 2.13 | 11.24 | negative | 13 |  |  |
| 1239 | male | 65 | negative | 143.09 | 9.53 | 1.56 | 15.01 | negative | 13 |  |  |
| 1240 | male | 64 | negative | 179.39 | 14.45 | 3.00 | 12.41 | negative | 13 |  |  |
| 1241 | male | 64 | negative | 82.96 | 5.46 | 2.48 | 15.19 | negative | 13 |  |  |
| 1242 | female | 64 | positive | 229.27 | 29.56 | 16.02 | 7.76 | negative | 12 |  |  |
| 1243 | male | 64 | negative | 156.02 | 11.37 | 5.62 | 13.72 | negative | 13 |  |  |
| 1244 | female | 64 | positive | 51.31 | 13.34 | 13.75 | 3.85 | negative | 15 |  |  |
| 1245 | male | 65 | positive | 104.70 | 14.10 | 10.30 | 7.43 | negative | 16 |  |  |
| 1246 | male | 64 | negative | 160.80 | 19.21 | 2.97 | 8.37 | negative | 13 |  |  |
| 1247 | male | 64 | negative | 73.18 | 5.93 | 2.31 | 12.34 | negative | 13 |  |  |
| 1248 | male | 64 | positive | 238.09 | 27.03 | 18.73 | 8.81 | negative | 16 |  |  |
| 1249 | male | 64 | negative | 87.78 | 13.09 | 2.44 | 6.71 | negative | 13 |  |  |
| 1250 | male | 64 | negative | 96.52 | 4.24 | 3.64 | 22.76 | negative | 13 |  |  |
| 1251 | male | 64 | negative | 94.85 | 7.65 | 5.57 | 12.40 | negative | 13 |  |  |
| 1252 | male | 63 | negative | 237.73 | 23.94 | 5.22 | 9.93 | negative | 13 |  |  |
| 1253 | male | 63 | negative | 119.38 | 7.97 | 6.83 | 14.98 | negative | 15 |  |  |
| 1254 | male | 63 | positive | 91.40 | 7.83 | 3.05 | 11.67 | negative | 14 |  |  |
| 1255 | male | 63 | negative | 137.10 | 43.80 | 7.50 | 3.13 | negative | 18 |  |  |
| 1256 | female | 63 | positive | 151.33 | 20.20 | 7.28 | 7.49 | negative | 12 |  |  |
| 1257 | male | 63 | negative | 115.41 | 9.43 | 2.70 | 12.24 | negative | 13 |  |  |
| 1258 | male | 63 | negative | 94.65 | 5.38 | 3.01 | 17.59 | negative | 13 |  |  |
| 1259 | male | 63 | positive | 113.70 | 19.73 | 7.18 | 5.76 | negative | 16 |  |  |
| 1260 | male | 63 | negative | 97.08 | 5.88 | 2.46 | 16.51 | negative | 13 |  |  |
| 1261 | male | 63 | positive | 83.94 | 12.01 | 3.38 | 6.99 | negative | 14 |  |  |
| 1262 | male | 63 | negative | 99.78 | 10.50 | 4.70 | 9.50 | negative | 13 |  |  |
| 1263 | male | 63 | positive | 281.13 | 25.63 | 21.58 | 10.97 | negative | 16 |  |  |
| 1264 | male | 63 | negative | 38.67 | 4.60 | 2.58 | 8.41 | negative | 13 |  |  |
| 1265 | male | 63 | positive | 74.02 | 11.18 | 7.00 | 6.62 | negative | 16 |  |  |
| 1266 | female | 63 | positive | 112.12 | 20.09 | 6.81 | 5.58 | negative | 12 |  |  |
| 1267 | male | 63 | negative | 57.70 | 13.00 | 2.40 | 4.44 | negative | 13 |  |  |
| 1268 | male | 63 | negative | 124.74 | 13.35 | 4.14 | 9.34 | negative | 13 |  |  |
| 1269 | male | 63 | negative | 91.62 | 6.81 | 5.04 | 13.45 | negative | 13 |  |  |
| 1270 | female | 63 | positive | 171.55 | 16.53 | 6.48 | 10.38 | negative | 12 |  |  |
| 1271 | male | 63 | positive | 115.00 | 17.90 | 19.30 | 6.42 | negative | 16 |  |  |
| 1272 | male | 63 | positive | 75.18 | 9.48 | 4.43 | 7.93 | negative | 14 |  |  |
| 1273 | male | 63 | positive | 113.40 | 18.80 | 4.80 | 6.03 | negative | 14 |  |  |
| 1274 | female | 63 | positive | 169.42 | 26.21 | 5.88 | 6.46 | negative | 12 |  |  |
| 1275 | female | 63 | positive | 154.24 | 32.23 | 32.16 | 4.79 | negative | 12 |  |  |
| 1276 | male | 63 | negative | 127.71 | 4.85 | 2.79 | 26.33 | negative | 13 |  |  |
| 1277 | male | 63 | negative | 169.56 | 10.90 | 3.48 | 15.56 | negative | 13 |  |  |
| 1278 | female | 63 | positive | 95.50 | 22.89 | 6.04 | 4.17 | negative | 12 |  |  |
| 1279 | male | 63 | negative | 96.52 | 5.96 | 2.35 | 16.19 | negative | 13 |  |  |
| 1280 | male | 63 | negative | 94.04 | 6.16 | 6.66 | 15.27 | negative | 15 |  |  |
| 1281 | male | 63 | negative | 83.20 | 6.27 | 3.93 | 13.27 | negative | 13 |  |  |
| 1282 | male | 63 | negative | 147.13 | 10.62 | 7.29 | 13.85 | negative | 15 |  |  |
| 1283 | female | 63 | negative | 23.95 | 6.84 | 60.00 | 3.50 | negative | 14 |  |  |
| 1284 | male | 63 | positive | 100.40 | 12.83 | 5.80 | 7.83 | negative | 16 |  |  |
| 1285 | male | 63 | positive | 157.56 | 25.21 | 6.10 | 6.25 | negative | 16 |  |  |
| 1286 | male | 63 | negative | 256.40 | 39.90 | 22.50 | 6.43 | negative | 15 |  |  |
| 1287 | male | 63 | negative | 133.09 | 7.32 | 5.43 | 18.18 | negative | 13 |  |  |
| 1288 | male | 63 | negative | 100.36 | 6.98 | 2.32 | 14.38 | negative | 13 |  |  |
| 1289 | male | 63 | negative | 84.94 | 6.90 | 2.48 | 12.31 | negative | 13 |  |  |
| 1290 | male | 63 | positive | 71.07 | 11.44 | 7.63 | 6.21 | negative | 16 |  |  |
| 1291 | male | 63 | negative | 130.50 | 12.50 | 2.40 | 10.44 | negative | 13 |  |  |
| 1292 | male | 63 | negative | 162.06 | 12.43 | 2.36 | 13.04 | negative | 13 |  |  |
| 1293 | male | 63 | negative | 162.53 | 10.97 | 2.48 | 14.82 | negative | 13 |  |  |
| 1294 | male | 63 | positive | 192.76 | 33.14 | 23.90 | 5.82 | negative | 16 |  |  |
| 1295 | female | 63 | positive | 94.42 | 25.12 | 4.72 | 3.76 | negative | 13 |  |  |
| 1296 | male | 63 | positive | 134.56 | 22.04 | 3.86 | 6.11 | negative | 14 |  |  |
| 1297 | male | 63 | negative | 68.73 | 7.52 | 4.08 | 9.14 | negative | 13 |  |  |
| 1298 | male | 63 | negative | 126.39 | 5.89 | 3.76 | 21.46 | negative | 13 |  |  |
| 1299 | male | 63 | negative | 126.10 | 11.89 | 2.51 | 10.61 | negative | 13 |  |  |
| 1300 | male | 63 | positive | 300.00 | 30.95 | 6.02 | 9.69 | negative | 16 |  |  |
| 1301 | female | 63 | positive | 107.81 | 16.15 | 6.04 | 6.68 | negative | 12 |  |  |
| 1302 | male | 63 | negative | 97.19 | 8.76 | 2.70 | 11.09 | negative | 13 |  |  |
| 1303 | male | 63 | positive | 101.49 | 15.16 | 7.18 | 6.69 | negative | 16 |  |  |
| 1304 | male | 63 | negative | 110.74 | 25.09 | 59.16 | 4.41 | negative | 15 |  |  |
| 1305 | male | 63 | negative | 148.30 | 11.01 | 3.59 | 13.47 | negative | 13 |  |  |
| 1306 | female | 63 | positive | 100.89 | 10.58 | 7.14 | 9.54 | negative | 12 |  |  |
| 1307 | male | 63 | positive | 119.31 | 16.09 | 6.32 | 7.42 | negative | 16 |  |  |
| 1308 | male | 63 | negative | 56.07 | 7.06 | 4.35 | 7.94 | negative | 13 |  |  |
| 1309 | male | 63 | negative | 69.20 | 3.80 | 2.57 | 18.21 | negative | 13 |  |  |
| 1310 | male | 63 | negative | 47.46 | 5.18 | 3.30 | 9.16 | negative | 13 |  |  |
| 1311 | male | 63 | negative | 127.86 | 8.87 | 3.77 | 14.41 | negative | 13 |  |  |
| 1312 | male | 63 | negative | 121.55 | 6.32 | 2.96 | 19.23 | negative | 13 |  |  |
| 1313 | male | 63 | positive | 175.64 | 21.91 | 4.96 | 8.02 | negative | 14 |  |  |
| 1314 | male | 63 | negative | 76.18 | 9.21 | 2.18 | 8.27 | negative | 13 |  |  |
| 1315 | female | 63 | negative | 163.41 | 46.19 | 23.02 | 3.54 | negative | 14 |  |  |
| 1316 | male | 63 | negative | 62.72 | 6.74 | 2.75 | 9.31 | negative | 13 |  |  |
| 1317 | male | 63 | negative | 190.64 | 7.52 | 3.25 | 25.35 | negative | 13 |  |  |
| 1318 | female | 63 | negative | 28.37 | 7.31 | 42.35 | 3.88 | negative | 14 |  |  |
| 1319 | male | 63 | positive | 111.65 | 18.32 | 7.09 | 6.09 | negative | 16 |  |  |
| 1320 | male | 63 | negative | 80.42 | 8.90 | 5.12 | 9.04 | negative | 13 |  |  |
| 1321 | female | 63 | positive | 114.13 | 13.76 | 6.69 | 8.29 | negative | 12 |  |  |
| 1322 | male | 63 | negative | 88.60 | 6.86 | 5.43 | 12.92 | negative | 13 |  |  |
| 1323 | male | 63 | negative | 175.10 | 19.20 | 34.25 | 9.12 | negative | 15 |  |  |
| 1324 | male | 63 | negative | 105.09 | 10.69 | 4.58 | 9.83 | negative | 13 |  |  |
| 1325 | male | 63 | positive | 78.50 | 9.23 | 3.48 | 8.50 | negative | 14 |  |  |
| 1326 | male | 63 | negative | 144.03 | 13.76 | 6.47 | 10.47 | negative | 15 |  |  |
| 1327 | male | 63 | negative | 90.77 | 8.91 | 2.70 | 10.19 | negative | 13 |  |  |
| 1328 | female | 63 | positive | 141.38 | 23.75 | 5.98 | 5.95 | negative | 12 |  |  |
| 1329 | male | 63 | negative | 70.80 | 7.00 | 2.90 | 10.11 | negative | 13 |  |  |
| 1330 | male | 63 | positive | 142.01 | 16.79 | 5.08 | 8.46 | negative | 14 |  |  |
| 1331 | male | 63 | positive | 125.68 | 16.57 | 20.45 | 7.58 | negative | 16 |  |  |
| 1332 | male | 63 | negative | 110.30 | 32.10 | 2.80 | 3.44 | negative | 16 |  |  |
| 1333 | male | 63 | negative | 71.46 | 11.63 | 2.63 | 6.14 | negative | 13 |  |  |
| 1334 | male | 63 | positive | 101.60 | 18.49 | 39.05 | 5.49 | negative | 16 |  |  |
| 1335 | male | 63 | negative | 105.52 | 7.28 | 2.80 | 14.49 | negative | 13 |  |  |
| 1336 | male | 63 | negative | 89.25 | 7.15 | 2.75 | 12.48 | negative | 13 |  |  |
| 1337 | male | 63 | negative | 108.71 | 11.84 | 2.94 | 9.18 | negative | 13 |  |  |
| 1338 | male | 63 | positive | 260.58 | 35.96 | 36.06 | 7.25 | negative | 16 |  |  |
| 1339 | male | 63 | positive | 165.52 | 22.16 | 4.70 | 7.47 | negative | 14 |  |  |
| 1340 | male | 63 | negative | 143.36 | 12.17 | 2.97 | 11.78 | negative | 13 |  |  |
| 1341 | male | 63 | positive | 240.94 | 18.05 | 2.29 | 13.35 | negative | 14 |  |  |
| 1342 | male | 63 | positive | 75.63 | 14.28 | 3.56 | 5.30 | negative | 14 |  |  |
| 1343 | male | 63 | positive | 228.50 | 15.30 | 4.20 | 14.93 | negative | 14 |  |  |
| 1344 | female | 63 | positive | 71.62 | 16.87 | 6.18 | 4.25 | negative | 12 |  |  |
| 1345 | male | 63 | negative | 101.67 | 3.36 | 5.95 | 30.26 | negative | 15 |  |  |
| 1346 | male | 63 | negative | 144.97 | 14.50 | 3.92 | 10.00 | negative | 13 |  |  |
| 1347 | male | 63 | negative | 283.68 | 10.48 | 2.43 | 27.07 | negative | 13 |  |  |
| 1348 | male | 63 | negative | 68.44 | 10.34 | 2.18 | 6.62 | negative | 13 |  |  |
| 1349 | male | 63 | negative | 273.69 | 10.30 | 2.09 | 26.57 | negative | 13 |  |  |
| 1350 | male | 63 | negative | 159.14 | 12.75 | 3.27 | 12.48 | negative | 13 |  |  |
| 1351 | male | 63 | positive | 141.04 | 23.45 | 6.82 | 6.01 | negative | 16 |  |  |
| 1352 | female | 63 | positive | 88.92 | 13.16 | 6.29 | 6.76 | negative | 12 |  |  |
| 1353 | male | 63 | positive | 106.33 | 10.19 | 4.60 | 10.43 | negative | 14 |  |  |
| 1354 | male | 63 | positive | 79.56 | 12.28 | 5.97 | 6.48 | negative | 16 |  |  |
| 1355 | male | 63 | negative | 116.26 | 11.35 | 2.08 | 10.24 | negative | 13 |  |  |
| 1356 | male | 63 | negative | 165.41 | 13.17 | 3.26 | 12.56 | negative | 13 |  |  |
| 1357 | female | 63 | negative | 18.78 | 6.52 | 60.00 | 2.88 | negative | 14 |  |  |
| 1358 | male | 63 | negative | 97.60 | 7.40 | 3.60 | 13.19 | negative | 13 |  |  |
| 1359 | male | 63 | positive | 44.30 | 10.20 | 33.80 | 4.34 | negative | 16 |  |  |
| 1360 | male | 63 | positive | 164.31 | 16.17 | 3.65 | 10.16 | negative | 14 |  |  |
| 1361 | male | 63 | positive | 133.52 | 13.85 | 3.09 | 9.64 | negative | 14 |  |  |
| 1362 | male | 63 | negative | 124.84 | 11.91 | 3.72 | 10.48 | negative | 13 |  |  |
| 1363 | male | 63 | negative | 112.04 | 14.32 | 4.85 | 7.82 | negative | 13 |  |  |
| 1364 | male | 63 | negative | 137.50 | 11.20 | 4.80 | 12.28 | negative | 13 |  |  |
| 1365 | male | 63 | positive | 61.42 | 17.31 | 5.12 | 3.55 | negative | 17 |  |  |
| 1366 | male | 63 | negative | 119.69 | 6.85 | 2.25 | 17.47 | negative | 13 |  |  |
| 1367 | male | 63 | negative | 59.43 | 3.52 | 3.95 | 16.88 | negative | 13 |  |  |
| 1368 | male | 63 | positive | 123.47 | 15.65 | 7.25 | 7.89 | negative | 16 |  |  |
| 1369 | male | 63 | negative | 165.15 | 10.86 | 2.23 | 15.21 | negative | 13 |  |  |
| 1370 | male | 63 | negative | 247.90 | 25.62 | 22.63 | 9.68 | negative | 15 |  |  |
| 1371 | female | 63 | negative | 10.71 | 4.23 | 39.49 | 2.53 | negative | 14 |  |  |
| 1372 | male | 63 | negative | 70.81 | 21.89 | 22.20 | 3.23 | negative | 18 |  |  |
| 1373 | male | 63 | negative | 121.52 | 13.49 | 5.85 | 9.01 | negative | 15 |  |  |
| 1374 | male | 63 | positive | 136.21 | 14.48 | 3.15 | 9.41 | negative | 14 |  |  |
| 1375 | male | 63 | negative | 168.27 | 13.57 | 3.48 | 12.40 | negative | 13 |  |  |
| 1376 | male | 63 | negative | 133.44 | 11.74 | 2.04 | 11.37 | negative | 13 |  |  |
| 1377 | male | 63 | negative | 231.77 | 17.86 | 6.71 | 12.98 | negative | 15 |  |  |
| 1378 | female | 63 | positive | 70.11 | 10.38 | 19.17 | 6.75 | negative | 12 |  |  |
| 1379 | male | 63 | negative | 74.57 | 6.40 | 22.53 | 11.65 | negative | 15 |  |  |
| 1380 | male | 63 | negative | 181.57 | 13.95 | 7.25 | 13.02 | negative | 15 |  |  |
| 1381 | female | 63 | positive | 122.47 | 15.92 | 6.34 | 7.69 | negative | 12 |  |  |
| 1382 | male | 62 | positive | 239.29 | 28.87 | 8.78 | 8.29 | negative | 16 |  |  |
| 1383 | female | 62 | negative | 9.37 | 2.64 | 60.00 | 3.55 | negative | 14 |  |  |
| 1384 | female | 63 | positive | 110.16 | 18.62 | 17.60 | 5.92 | negative | 12 |  |  |
| 1385 | female | 62 | positive | 85.10 | 8.53 | 8.61 | 9.98 | negative | 12 |  |  |
| 1386 | male | 63 | negative | 147.07 | 11.33 | 1.99 | 12.98 | negative | 13 |  |  |
| 1387 | male | 62 | negative | 64.69 | 11.00 | 9.10 | 5.88 | negative | 15 |  |  |
| 1388 | male | 62 | negative | 134.20 | 7.80 | 7.79 | 17.21 | negative | 15 |  |  |
| 1389 | male | 62 | negative | 111.58 | 10.84 | 4.62 | 10.29 | negative | 13 |  |  |
| 1390 | male | 62 | negative | 108.66 | 14.06 | 6.83 | 7.73 | negative | 15 |  |  |
| 1391 | female | 63 | positive | 63.05 | 21.32 | 12.42 | 2.96 | negative | 15 |  |  |
| 1392 | female | 62 | positive | 118.40 | 21.64 | 7.69 | 5.47 | negative | 12 |  |  |
| 1393 | male | 63 | negative | 29.61 | 2.81 | 1.81 | 10.54 | negative | 13 |  |  |
| 1394 | male | 63 | positive | 93.96 | 19.44 | 12.89 | 4.83 | negative | 16 |  |  |
| 1395 | male | 62 | negative | 159.41 | 16.42 | 5.27 | 9.71 | negative | 13 |  |  |
| 1396 | female | 62 | positive | 265.22 | 16.64 | 9.35 | 15.94 | negative | 12 |  |  |
| 1397 | female | 62 | positive | 131.58 | 11.12 | 6.00 | 11.83 | negative | 12 |  |  |
| 1398 | male | 62 | negative | 109.80 | 8.28 | 5.32 | 13.26 | negative | 13 |  |  |
| 1399 | male | 62 | negative | 300.00 | 21.17 | 8.23 | 14.17 | negative | 15 |  |  |
| 1400 | male | 62 | positive | 142.47 | 14.76 | 5.42 | 9.65 | negative | 14 |  |  |
| 1401 | male | 62 | positive | 147.40 | 22.15 | 5.94 | 6.65 | negative | 16 |  |  |
| 1402 | male | 62 | negative | 300.00 | 24.00 | 5.70 | 12.50 | negative | 13 |  |  |
| 1403 | female | 63 | positive | 86.81 | 23.62 | 12.07 | 3.68 | negative | 15 |  |  |
| 1404 | male | 63 | negative | 266.21 | 13.38 | 1.98 | 19.90 | negative | 13 |  |  |
| 1405 | male | 62 | positive | 71.80 | 17.90 | 5.90 | 4.01 | negative | 16 |  |  |
| 1406 | male | 63 | negative | 167.15 | 10.24 | 12.32 | 16.32 | negative | 15 |  |  |
| 1407 | male | 62 | negative | 88.23 | 16.20 | 5.38 | 5.45 | negative | 13 |  |  |
| 1408 | male | 62 | positive | 53.28 | 14.00 | 7.00 | 3.81 | negative | 19 |  |  |
| 1409 | male | 63 | negative | 35.87 | 10.40 | 1.57 | 3.45 | negative | 16 |  |  |
| 1410 | male | 62 | positive | 219.88 | 13.82 | 6.72 | 15.91 | negative | 16 |  |  |
| 1411 | male | 63 | negative | 92.21 | 6.64 | 1.74 | 13.89 | negative | 13 |  |  |
| 1412 | male | 63 | positive | 125.32 | 13.96 | 1.62 | 8.98 | negative | 14 |  |  |
| 1413 | female | 63 | positive | 278.96 | 25.06 | 12.98 | 11.13 | negative | 12 |  |  |
| 1414 | male | 63 | positive | 143.48 | 17.55 | 1.92 | 8.18 | negative | 14 |  |  |
| 1415 | male | 63 | negative | 97.13 | 9.37 | 11.39 | 10.37 | negative | 15 |  |  |
| 1416 | male | 63 | negative | 103.31 | 8.83 | 1.87 | 11.70 | negative | 13 |  |  |
| 1417 | female | 62 | positive | 140.45 | 17.80 | 9.52 | 7.89 | negative | 12 |  |  |
| 1418 | female | 62 | positive | 166.19 | 16.11 | 7.22 | 10.32 | negative | 12 |  |  |
| 1419 | male | 63 | negative | 297.35 | 23.07 | 14.25 | 12.89 | negative | 15 |  |  |
| 1420 | male | 63 | negative | 53.26 | 4.15 | 1.75 | 12.83 | negative | 13 |  |  |
| 1421 | male | 62 | negative | 11.90 | 10.31 | 53.39 | 1.15 | negative | 18 |  |  |
| 1422 | male | 63 | negative | 49.71 | 15.71 | 11.35 | 3.16 | negative | 18 |  |  |
| 1423 | male | 63 | positive | 149.09 | 25.18 | 12.75 | 5.92 | negative | 16 |  |  |
| 1424 | male | 62 | positive | 221.74 | 23.76 | 8.97 | 9.33 | negative | 16 |  |  |
| 1425 | female | 62 | positive | 104.79 | 13.68 | 8.52 | 7.66 | negative | 12 |  |  |
| 1426 | male | 62 | positive | 44.77 | 7.85 | 5.85 | 5.70 | negative | 16 |  |  |
| 1427 | male | 62 | positive | 144.10 | 24.30 | 8.20 | 5.93 | negative | 16 |  |  |
| 1428 | male | 62 | positive | 115.35 | 17.12 | 9.59 | 6.74 | negative | 16 |  |  |
| 1429 | female | 62 | positive | 188.16 | 19.37 | 5.90 | 9.71 | negative | 12 |  |  |
| 1430 | male | 62 | negative | 101.95 | 8.39 | 5.00 | 12.15 | negative | 13 |  |  |
| 1431 | male | 62 | negative | 128.54 | 8.21 | 6.25 | 15.66 | negative | 15 |  |  |
| 1432 | male | 62 | positive | 192.78 | 15.79 | 5.57 | 12.21 | negative | 14 |  |  |
| 1433 | male | 63 | negative | 118.41 | 16.56 | 1.84 | 7.15 | negative | 13 |  |  |
| 1434 | male | 63 | positive | 286.53 | 29.42 | 17.59 | 9.74 | negative | 16 |  |  |
| 1435 | female | 62 | positive | 133.97 | 28.58 | 6.23 | 4.69 | negative | 12 |  |  |
| 1436 | male | 62 | positive | 100.29 | 11.23 | 5.09 | 8.93 | negative | 14 |  |  |
| 1437 | male | 63 | negative | 97.68 | 8.95 | 1.63 | 10.91 | negative | 13 |  |  |
| 1438 | male | 63 | negative | 123.81 | 11.22 | 10.67 | 11.03 | negative | 15 |  |  |
| 1439 | male | 63 | positive | 72.22 | 23.08 | 16.43 | 3.13 | negative | 19 |  |  |
| 1440 | male | 62 | positive | 180.72 | 21.20 | 6.62 | 8.52 | negative | 16 |  |  |
| 1441 | male | 62 | negative | 102.61 | 10.28 | 5.10 | 9.98 | negative | 13 |  |  |
| 1442 | male | 62 | negative | 84.40 | 5.97 | 8.81 | 14.14 | negative | 15 |  |  |
| 1443 | male | 62 | positive | 208.30 | 23.00 | 4.40 | 9.06 | negative | 14 |  |  |
| 1444 | female | 63 | positive | 238.76 | 32.45 | 10.33 | 7.36 | negative | 12 |  |  |
| 1445 | male | 62 | negative | 247.77 | 21.04 | 7.49 | 11.78 | negative | 15 |  |  |
| 1446 | male | 62 | negative | 82.89 | 7.24 | 4.42 | 11.45 | negative | 13 |  |  |
| 1447 | female | 63 | positive | 136.58 | 21.30 | 11.34 | 6.41 | negative | 12 |  |  |
| 1448 | male | 62 | positive | 154.36 | 17.83 | 5.19 | 8.66 | negative | 14 |  |  |
| 1449 | female | 63 | positive | 119.20 | 25.90 | 15.20 | 4.60 | negative | 12 |  |  |
| 1450 | female | 62 | positive | 137.91 | 31.19 | 6.85 | 4.42 | negative | 12 |  |  |
| 1451 | male | 62 | positive | 94.28 | 14.01 | 7.97 | 6.73 | negative | 16 |  |  |
| 1452 | male | 62 | positive | 118.82 | 12.40 | 6.73 | 9.58 | negative | 16 |  |  |
| 1453 | female | 63 | positive | 229.35 | 21.94 | 11.40 | 10.45 | negative | 12 |  |  |
| 1454 | male | 63 | negative | 137.36 | 9.21 | 1.67 | 14.91 | negative | 13 |  |  |
| 1455 | male | 62 | negative | 139.94 | 9.47 | 5.69 | 14.78 | negative | 13 |  |  |
| 1456 | male | 62 | negative | 45.08 | 5.03 | 4.62 | 8.96 | negative | 13 |  |  |
| 1457 | male | 62 | positive | 193.80 | 21.80 | 53.66 | 8.89 | negative | 16 |  |  |
| 1458 | male | 62 | negative | 113.88 | 7.46 | 8.29 | 15.27 | negative | 15 |  |  |
| 1459 | male | 62 | positive | 100.05 | 15.53 | 6.30 | 6.44 | negative | 16 |  |  |
| 1460 | female | 62 | positive | 170.98 | 24.81 | 6.26 | 6.89 | negative | 12 |  |  |
| 1461 | female | 62 | positive | 154.74 | 15.55 | 8.90 | 9.95 | negative | 12 |  |  |
| 1462 | female | 63 | positive | 147.19 | 20.59 | 12.68 | 7.15 | negative | 12 |  |  |
| 1463 | male | 63 | negative | 115.15 | 14.02 | 11.16 | 8.21 | negative | 15 |  |  |
| 1464 | male | 62 | negative | 16.33 | 9.65 | 54.56 | 1.69 | negative | 18 |  |  |
| 1465 | female | 62 | positive | 107.71 | 29.31 | 4.48 | 3.67 | negative | 13 |  |  |
| 1466 | male | 63 | positive | 113.18 | 19.72 | 18.60 | 5.74 | negative | 16 |  |  |
| 1467 | male | 62 | negative | 98.29 | 11.81 | 5.95 | 8.32 | negative | 15 |  |  |
| 1468 | male | 63 | negative | 120.54 | 8.89 | 1.72 | 13.56 | negative | 13 |  |  |
| 1469 | male | 63 | negative | 189.99 | 22.36 | 17.13 | 8.50 | negative | 15 |  |  |
| 1470 | male | 63 | negative | 124.20 | 6.48 | 1.77 | 19.17 | negative | 13 |  |  |
| 1471 | male | 63 | negative | 204.44 | 14.77 | 1.73 | 13.84 | negative | 13 |  |  |
| 1472 | male | 62 | positive | 127.92 | 17.99 | 4.93 | 7.11 | negative | 14 |  |  |
| 1473 | male | 62 | negative | 95.43 | 10.18 | 6.72 | 9.37 | negative | 15 |  |  |
| 1474 | male | 63 | negative | 78.89 | 4.73 | 1.53 | 16.68 | negative | 13 |  |  |
| 1475 | male | 62 | positive | 193.12 | 14.44 | 5.88 | 13.37 | negative | 16 |  |  |
| 1476 | male | 63 | positive | 92.57 | 11.48 | 16.85 | 8.06 | negative | 16 |  |  |
| 1477 | female | 63 | negative | 40.68 | 11.56 | 1.78 | 3.52 | negative | 12 |  |  |
| 1478 | male | 62 | negative | 113.55 | 10.00 | 7.52 | 11.36 | negative | 15 |  |  |
| 1479 | male | 63 | negative | 42.37 | 4.31 | 18.12 | 9.83 | negative | 15 |  |  |
| 1480 | male | 62 | negative | 109.40 | 10.15 | 5.20 | 10.78 | negative | 13 |  |  |
| 1481 | male | 63 | negative | 78.60 | 6.37 | 1.55 | 12.34 | negative | 13 |  |  |
| 1482 | male | 62 | negative | 73.10 | 8.70 | 4.50 | 8.40 | negative | 13 |  |  |
| 1483 | female | 62 | positive | 188.86 | 14.81 | 6.84 | 12.75 | negative | 12 |  |  |
| 1484 | female | 62 | positive | 39.49 | 11.40 | 5.04 | 3.46 | negative | 13 |  |  |
| 1485 | male | 62 | negative | 279.14 | 13.78 | 51.26 | 20.26 | negative | 15 |  |  |
| 1486 | male | 62 | positive | 166.12 | 23.51 | 7.30 | 7.07 | negative | 16 |  |  |
| 1487 | male | 63 | negative | 149.86 | 10.19 | 17.74 | 14.71 | negative | 15 |  |  |
| 1488 | female | 62 | positive | 53.70 | 21.72 | 5.35 | 2.47 | negative | 13 |  |  |
| 1489 | female | 62 | positive | 133.12 | 11.39 | 9.60 | 11.69 | negative | 12 |  |  |
| 1490 | male | 63 | negative | 120.34 | 7.68 | 1.87 | 15.67 | negative | 13 |  |  |
| 1491 | male | 63 | negative | 95.87 | 7.13 | 1.60 | 13.45 | negative | 13 |  |  |
| 1492 | female | 63 | positive | 130.76 | 20.91 | 10.28 | 6.25 | negative | 12 |  |  |
| 1493 | male | 62 | negative | 72.74 | 8.40 | 8.13 | 8.66 | negative | 15 |  |  |
| 1494 | male | 62 | negative | 101.73 | 9.25 | 6.38 | 11.00 | negative | 15 |  |  |
| 1495 | male | 63 | negative | 57.87 | 11.84 | 10.95 | 4.89 | negative | 15 |  |  |
| 1496 | male | 62 | positive | 154.22 | 13.22 | 5.28 | 11.67 | negative | 14 |  |  |
| 1497 | male | 63 | negative | 70.02 | 6.69 | 1.98 | 10.47 | negative | 13 |  |  |
| 1498 | male | 62 | negative | 83.64 | 4.00 | 4.99 | 20.91 | negative | 13 |  |  |
| 1499 | male | 63 | negative | 156.51 | 10.49 | 1.85 | 14.92 | negative | 13 |  |  |
| 1500 | male | 62 | positive | 127.80 | 14.66 | 4.44 | 8.72 | negative | 14 |  |  |
| 1501 | female | 63 | positive | 173.88 | 7.56 | 10.20 | 23.00 | negative | 12 |  |  |
| 1502 | male | 63 | negative | 131.95 | 8.98 | 11.24 | 14.69 | negative | 15 |  |  |
| 1503 | male | 62 | negative | 84.10 | 8.70 | 8.28 | 9.67 | negative | 15 |  |  |
| 1504 | male | 62 | positive | 87.99 | 11.75 | 8.24 | 7.49 | negative | 16 |  |  |
| 1505 | male | 63 | negative | 95.30 | 8.50 | 1.80 | 11.21 | negative | 13 |  |  |
| 1506 | male | 63 | positive | 60.13 | 9.47 | 1.56 | 6.35 | negative | 14 |  |  |
| 1507 | male | 62 | negative | 122.87 | 7.77 | 4.52 | 15.81 | negative | 13 |  |  |
| 1508 | male | 62 | negative | 144.56 | 10.03 | 5.56 | 14.41 | negative | 13 |  |  |
| 1509 | female | 62 | positive | 89.88 | 17.08 | 24.42 | 5.26 | negative | 12 |  |  |
| 1510 | male | 62 | positive | 94.51 | 15.16 | 3.07 | 6.23 | negative | 14 |  |  |
| 1511 | male | 61 | positive | 167.86 | 14.45 | 8.67 | 11.62 | negative | 16 |  |  |
| 1512 | male | 62 | negative | 79.96 | 8.02 | 2.39 | 9.97 | negative | 13 |  |  |
| 1513 | male | 62 | negative | 108.67 | 9.12 | 3.92 | 11.92 | negative | 13 |  |  |
| 1514 | male | 62 | negative | 99.95 | 7.33 | 2.87 | 13.64 | negative | 13 |  |  |
| 1515 | male | 62 | negative | 151.03 | 24.99 | 21.55 | 6.04 | negative | 15 |  |  |
| 1516 | female | 61 | positive | 95.05 | 12.75 | 9.80 | 7.45 | negative | 12 |  |  |
| 1517 | male | 61 | positive | 209.43 | 17.76 | 5.52 | 11.79 | negative | 14 |  |  |
| 1518 | male | 62 | negative | 186.23 | 26.73 | 15.24 | 6.97 | negative | 15 |  |  |
| 1519 | male | 61 | negative | 183.90 | 13.85 | 8.00 | 13.28 | negative | 15 |  |  |
| 1520 | male | 62 | negative | 93.87 | 10.09 | 2.69 | 9.30 | negative | 13 |  |  |
| 1521 | female | 61 | positive | 100.60 | 9.42 | 7.37 | 10.68 | negative | 12 |  |  |
| 1522 | male | 62 | negative | 92.00 | 6.40 | 23.60 | 14.38 | negative | 15 |  |  |
| 1523 | male | 61 | positive | 207.74 | 19.05 | 6.93 | 10.90 | negative | 16 |  |  |
| 1524 | male | 62 | negative | 84.80 | 12.30 | 2.20 | 6.89 | negative | 13 |  |  |
| 1525 | male | 61 | negative | 58.81 | 7.70 | 5.64 | 7.64 | negative | 13 |  |  |
| 1526 | male | 61 | negative | 120.68 | 6.57 | 6.27 | 18.37 | negative | 15 |  |  |
| 1527 | male | 62 | negative | 73.79 | 5.69 | 2.36 | 12.97 | negative | 13 |  |  |
| 1528 | male | 62 | negative | 166.42 | 13.12 | 3.38 | 12.68 | negative | 13 |  |  |
| 1529 | female | 62 | positive | 44.16 | 19.66 | 2.87 | 2.25 | negative | 13 |  |  |
| 1530 | male | 61 | positive | 118.87 | 18.03 | 7.42 | 6.59 | negative | 16 |  |  |
| 1531 | male | 61 | negative | 106.03 | 9.44 | 6.34 | 11.23 | negative | 15 |  |  |
| 1532 | male | 62 | positive | 31.61 | 7.48 | 2.60 | 4.23 | negative | 14 |  |  |
| 1533 | male | 62 | negative | 88.35 | 3.27 | 3.95 | 27.02 | negative | 13 |  |  |
| 1534 | female | 61 | positive | 240.87 | 17.96 | 9.52 | 13.41 | negative | 12 |  |  |
| 1535 | male | 62 | negative | 73.90 | 9.00 | 3.60 | 8.21 | negative | 13 |  |  |
| 1536 | male | 62 | negative | 78.89 | 6.07 | 2.81 | 13.00 | negative | 13 |  |  |
| 1537 | male | 62 | positive | 90.79 | 4.44 | 2.41 | 20.45 | negative | 14 |  |  |
| 1538 | male | 62 | negative | 128.88 | 9.45 | 2.02 | 13.64 | negative | 13 |  |  |
| 1539 | male | 62 | negative | 90.00 | 12.80 | 11.50 | 7.03 | negative | 15 |  |  |
| 1540 | male | 62 | positive | 130.04 | 27.76 | 13.93 | 4.68 | negative | 16 |  |  |
| 1541 | male | 61 | negative | 114.56 | 9.05 | 6.42 | 12.66 | negative | 15 |  |  |
| 1542 | male | 62 | positive | 41.93 | 7.10 | 1.79 | 5.91 | negative | 14 |  |  |
| 1543 | male | 61 | positive | 141.31 | 12.94 | 9.19 | 10.92 | negative | 16 |  |  |
| 1544 | female | 61 | positive | 167.83 | 23.24 | 7.99 | 7.22 | negative | 12 |  |  |
| 1545 | male | 62 | negative | 292.28 | 14.42 | 2.74 | 20.27 | negative | 13 |  |  |
| 1546 | female | 62 | positive | 61.87 | 4.47 | 30.76 | 13.84 | negative | 12 |  |  |
| 1547 | male | 62 | negative | 132.73 | 7.71 | 4.24 | 17.22 | negative | 13 |  |  |
| 1548 | male | 62 | negative | 121.69 | 7.33 | 3.53 | 16.60 | negative | 13 |  |  |
| 1549 | female | 62 | positive | 87.51 | 18.49 | 20.50 | 4.73 | negative | 12 |  |  |
| 1550 | male | 62 | positive | 87.83 | 18.02 | 19.42 | 4.87 | negative | 16 |  |  |
| 1551 | male | 62 | negative | 107.98 | 11.95 | 2.57 | 9.04 | negative | 13 |  |  |
| 1552 | male | 62 | negative | 88.99 | 4.93 | 2.41 | 18.05 | negative | 13 |  |  |
| 1553 | male | 62 | negative | 50.70 | 13.16 | 3.39 | 3.85 | negative | 16 |  |  |
| 1554 | male | 62 | negative | 92.04 | 5.91 | 1.56 | 15.57 | negative | 13 |  |  |
| 1555 | male | 62 | negative | 100.57 | 7.68 | 1.61 | 13.10 | negative | 13 |  |  |
| 1556 | male | 62 | negative | 272.36 | 13.70 | 3.92 | 19.88 | negative | 13 |  |  |
| 1557 | male | 61 | positive | 85.80 | 13.00 | 5.60 | 6.60 | negative | 14 |  |  |
| 1558 | male | 62 | negative | 118.02 | 14.75 | 3.71 | 8.00 | negative | 13 |  |  |
| 1559 | female | 61 | positive | 225.96 | 34.65 | 5.75 | 6.52 | negative | 12 |  |  |
| 1560 | male | 62 | negative | 77.87 | 4.24 | 1.99 | 18.37 | negative | 13 |  |  |
| 1561 | male | 62 | negative | 118.72 | 12.16 | 3.50 | 9.76 | negative | 13 |  |  |
| 1562 | male | 62 | negative | 90.69 | 11.22 | 3.49 | 8.08 | negative | 13 |  |  |
| 1563 | female | 62 | positive | 204.81 | 28.86 | 24.47 | 7.10 | negative | 12 |  |  |
| 1564 | male | 61 | positive | 158.73 | 12.92 | 5.30 | 12.29 | negative | 14 |  |  |
| 1565 | male | 62 | negative | 67.59 | 4.16 | 3.14 | 16.25 | negative | 13 |  |  |
| 1566 | male | 62 | negative | 150.95 | 7.10 | 4.40 | 21.26 | negative | 13 |  |  |
| 1567 | male | 62 | negative | 100.47 | 6.52 | 1.78 | 15.41 | negative | 13 |  |  |
| 1568 | female | 62 | positive | 291.57 | 23.39 | 14.50 | 12.47 | negative | 12 |  |  |
| 1569 | male | 62 | negative | 94.09 | 5.19 | 1.52 | 18.13 | negative | 13 |  |  |
| 1570 | male | 62 | positive | 74.34 | 11.76 | 2.80 | 6.32 | negative | 14 |  |  |
| 1571 | male | 62 | negative | 126.38 | 7.70 | 2.21 | 16.41 | negative | 13 |  |  |
| 1572 | male | 62 | positive | 83.68 | 13.81 | 3.47 | 6.06 | negative | 14 |  |  |
| 1573 | male | 62 | negative | 236.57 | 13.91 | 3.48 | 17.01 | negative | 13 |  |  |
| 1574 | male | 62 | negative | 12.66 | 8.65 | 24.62 | 1.46 | negative | 18 |  |  |
| 1575 | male | 62 | negative | 63.07 | 6.04 | 3.04 | 10.44 | negative | 13 |  |  |
| 1576 | female | 61 | positive | 74.52 | 12.47 | 6.49 | 5.98 | negative | 12 |  |  |
| 1577 | male | 61 | positive | 180.16 | 18.25 | 8.45 | 9.87 | negative | 16 |  |  |
| 1578 | male | 61 | negative | 300.00 | 30.20 | 9.87 | 9.93 | negative | 15 |  |  |
| 1579 | male | 62 | negative | 88.10 | 6.34 | 4.17 | 13.90 | negative | 13 |  |  |
| 1580 | male | 61 | positive | 76.80 | 14.30 | 9.00 | 5.37 | negative | 16 |  |  |
| 1581 | male | 62 | negative | 63.14 | 5.29 | 3.01 | 11.94 | negative | 13 |  |  |
| 1582 | male | 62 | negative | 105.00 | 7.96 | 15.06 | 13.19 | negative | 15 |  |  |
| 1583 | male | 62 | negative | 150.91 | 22.42 | 14.77 | 6.73 | negative | 15 |  |  |
| 1584 | male | 62 | negative | 136.43 | 9.12 | 4.11 | 14.96 | negative | 13 |  |  |
| 1585 | male | 62 | negative | 90.19 | 6.99 | 3.80 | 12.90 | negative | 13 |  |  |
| 1586 | male | 62 | negative | 38.97 | 5.84 | 4.18 | 6.67 | negative | 13 |  |  |
| 1587 | male | 62 | negative | 113.64 | 7.77 | 1.57 | 14.63 | negative | 13 |  |  |
| 1588 | male | 62 | positive | 320.60 | 78.80 | 17.10 | 4.07 | negative | 16 |  |  |
| 1589 | male | 62 | negative | 84.25 | 7.75 | 3.03 | 10.87 | negative | 13 |  |  |
| 1590 | male | 62 | negative | 90.98 | 11.06 | 17.53 | 8.23 | negative | 15 |  |  |
| 1591 | male | 62 | positive | 22.21 | 12.05 | 30.84 | 1.84 | negative | 19 |  |  |
| 1592 | male | 62 | negative | 194.83 | 16.87 | 2.60 | 11.55 | negative | 13 |  |  |
| 1593 | female | 62 | positive | 89.77 | 15.79 | 12.48 | 5.69 | negative | 12 |  |  |
| 1594 | female | 62 | positive | 62.16 | 17.85 | 14.63 | 3.48 | negative | 15 |  |  |
| 1595 | male | 62 | negative | 84.05 | 6.84 | 1.96 | 12.29 | negative | 13 |  |  |
| 1596 | male | 62 | negative | 104.97 | 6.92 | 2.37 | 15.17 | negative | 13 |  |  |
| 1597 | male | 62 | negative | 178.10 | 14.02 | 1.59 | 12.70 | negative | 13 |  |  |
| 1598 | male | 62 | negative | 202.01 | 20.53 | 3.66 | 9.84 | negative | 13 |  |  |
| 1599 | female | 61 | positive | 45.67 | 8.96 | 5.91 | 5.10 | negative | 12 |  |  |
| 1600 | male | 62 | negative | 82.44 | 4.63 | 2.32 | 17.81 | negative | 13 |  |  |
| 1601 | male | 61 | positive | 130.66 | 17.21 | 6.71 | 7.59 | negative | 16 |  |  |
| 1602 | male | 61 | positive | 120.86 | 15.02 | 5.42 | 8.05 | negative | 14 |  |  |
| 1603 | male | 62 | negative | 51.87 | 4.54 | 2.07 | 11.43 | negative | 13 |  |  |
| 1604 | male | 62 | negative | 240.07 | 19.09 | 4.11 | 12.58 | negative | 13 |  |  |
| 1605 | male | 62 | positive | 119.52 | 22.21 | 4.30 | 5.38 | negative | 14 |  |  |
| 1606 | male | 62 | positive | 252.17 | 21.43 | 10.32 | 11.77 | negative | 16 |  |  |
| 1607 | female | 61 | negative | 34.76 | 11.72 | 56.47 | 2.97 | negative | 14 |  |  |
| 1608 | male | 62 | positive | 122.27 | 13.01 | 18.28 | 9.40 | negative | 16 |  |  |
| 1609 | male | 62 | negative | 59.92 | 6.02 | 3.86 | 9.95 | negative | 13 |  |  |
| 1610 | male | 62 | negative | 85.82 | 7.69 | 1.52 | 11.16 | negative | 13 |  |  |
| 1611 | male | 61 | positive | 83.46 | 26.03 | 7.77 | 3.21 | negative | 19 |  |  |
| 1612 | male | 62 | positive | 268.54 | 35.69 | 18.65 | 7.52 | negative | 16 |  |  |
| 1613 | male | 61 | negative | 81.33 | 7.22 | 8.22 | 11.26 | negative | 15 |  |  |
| 1614 | male | 62 | negative | 70.40 | 6.40 | 1.60 | 11.00 | negative | 13 |  |  |
| 1615 | male | 62 | negative | 96.36 | 12.45 | 4.13 | 7.74 | negative | 13 |  |  |
| 1616 | male | 61 | positive | 162.50 | 18.64 | 6.37 | 8.72 | negative | 16 |  |  |
| 1617 | male | 62 | negative | 64.69 | 4.56 | 1.81 | 14.19 | negative | 13 |  |  |
| 1618 | male | 62 | negative | 84.94 | 14.54 | 19.87 | 5.84 | negative | 15 |  |  |
| 1619 | male | 62 | negative | 118.97 | 5.19 | 3.06 | 22.92 | negative | 13 |  |  |
| 1620 | male | 62 | negative | 183.15 | 9.99 | 3.15 | 18.33 | negative | 13 |  |  |
| 1621 | male | 62 | positive | 99.27 | 8.84 | 11.41 | 11.23 | negative | 16 |  |  |
| 1622 | male | 62 | negative | 218.76 | 10.07 | 2.34 | 21.72 | negative | 13 |  |  |
| 1623 | male | 62 | negative | 120.46 | 7.50 | 10.41 | 16.06 | negative | 15 |  |  |
| 1624 | male | 62 | negative | 124.66 | 7.14 | 2.58 | 17.46 | negative | 13 |  |  |
| 1625 | male | 61 | negative | 143.50 | 9.11 | 5.33 | 15.75 | negative | 13 |  |  |
| 1626 | female | 62 | positive | 104.31 | 24.32 | 14.22 | 4.29 | negative | 12 |  |  |
| 1627 | male | 61 | negative | 70.29 | 3.81 | 7.37 | 18.45 | negative | 15 |  |  |
| 1628 | male | 62 | positive | 130.43 | 11.60 | 4.12 | 11.24 | negative | 14 |  |  |
| 1629 | male | 62 | negative | 98.74 | 14.84 | 11.39 | 6.65 | negative | 15 |  |  |
| 1630 | male | 62 | positive | 101.20 | 13.20 | 2.70 | 7.67 | negative | 14 |  |  |
| 1631 | male | 62 | positive | 291.35 | 33.27 | 21.05 | 8.76 | negative | 16 |  |  |
| 1632 | male | 62 | positive | 300.00 | 47.70 | 24.41 | 6.29 | negative | 16 |  |  |
| 1633 | male | 61 | positive | 285.63 | 28.77 | 8.56 | 9.93 | negative | 16 |  |  |
| 1634 | male | 62 | negative | 116.37 | 11.93 | 2.64 | 9.75 | negative | 13 |  |  |
| 1635 | female | 62 | positive | 68.77 | 22.34 | 2.51 | 3.08 | negative | 13 |  |  |
| 1636 | male | 61 | negative | 142.76 | 10.45 | 6.27 | 13.66 | negative | 15 |  |  |
| 1637 | male | 61 | positive | 111.14 | 23.61 | 5.87 | 4.71 | negative | 16 |  |  |
| 1638 | male | 62 | negative | 43.09 | 10.06 | 31.47 | 4.28 | negative | 15 |  |  |
| 1639 | male | 62 | positive | 113.41 | 13.52 | 28.83 | 8.39 | negative | 16 |  |  |
| 1640 | male | 61 | negative | 104.21 | 10.03 | 5.51 | 10.39 | negative | 13 |  |  |
| 1641 | female | 62 | positive | 258.44 | 16.10 | 11.70 | 16.05 | negative | 12 |  |  |
| 1642 | male | 62 | positive | 46.93 | 17.74 | 3.07 | 2.65 | negative | 17 |  |  |
| 1643 | male | 62 | negative | 167.65 | 7.96 | 3.17 | 21.06 | negative | 13 |  |  |
| 1644 | male | 62 | negative | 99.86 | 7.00 | 2.12 | 14.27 | negative | 13 |  |  |
| 1645 | female | 61 | positive | 159.18 | 17.92 | 8.32 | 8.88 | negative | 12 |  |  |
| 1646 | male | 62 | negative | 88.54 | 9.81 | 1.88 | 9.03 | negative | 13 |  |  |
| 1647 | male | 62 | positive | 101.08 | 12.68 | 2.36 | 7.97 | negative | 14 |  |  |
| 1648 | male | 62 | negative | 228.81 | 12.14 | 3.82 | 18.85 | negative | 13 |  |  |
| 1649 | male | 62 | negative | 278.29 | 30.53 | 2.24 | 9.12 | negative | 13 |  |  |
| 1650 | male | 62 | negative | 62.92 | 7.33 | 1.65 | 8.58 | negative | 13 |  |  |
| 1651 | male | 62 | positive | 145.12 | 15.83 | 3.54 | 9.17 | negative | 14 |  |  |
| 1652 | male | 62 | negative | 75.70 | 4.65 | 2.54 | 16.28 | negative | 13 |  |  |
| 1653 | male | 61 | negative | 107.44 | 13.34 | 6.42 | 8.05 | negative | 15 |  |  |
| 1654 | male | 62 | positive | 137.10 | 12.90 | 12.40 | 10.63 | negative | 16 |  |  |
| 1655 | male | 62 | negative | 105.42 | 10.05 | 2.66 | 10.49 | negative | 13 |  |  |
| 1656 | male | 61 | negative | 91.88 | 11.48 | 9.41 | 8.00 | negative | 15 |  |  |
| 1657 | male | 62 | negative | 182.55 | 16.18 | 3.15 | 11.28 | negative | 13 |  |  |
| 1658 | male | 62 | negative | 35.82 | 16.23 | 18.28 | 2.21 | negative | 18 |  |  |
| 1659 | male | 62 | positive | 177.47 | 44.68 | 11.56 | 3.97 | negative | 16 |  |  |
| 1660 | male | 62 | positive | 202.69 | 15.07 | 3.26 | 13.45 | negative | 14 |  |  |
| 1661 | male | 62 | positive | 43.27 | 19.21 | 13.75 | 2.25 | negative | 19 |  |  |
| 1662 | male | 62 | negative | 113.49 | 8.79 | 2.68 | 12.91 | negative | 13 |  |  |
| 1663 | male | 62 | positive | 72.50 | 15.80 | 29.10 | 4.59 | negative | 16 |  |  |
| 1664 | male | 62 | negative | 104.80 | 15.68 | 3.09 | 6.68 | negative | 13 |  |  |
| 1665 | male | 61 | negative | 98.47 | 11.41 | 6.17 | 8.63 | negative | 15 |  |  |
| 1666 | male | 62 | positive | 277.84 | 30.79 | 34.94 | 9.02 | negative | 16 |  |  |
| 1667 | female | 61 | positive | 119.17 | 25.57 | 9.76 | 4.66 | negative | 12 |  |  |
| 1668 | male | 62 | negative | 119.19 | 6.23 | 3.60 | 19.13 | negative | 13 |  |  |
| 1669 | male | 62 | negative | 78.38 | 9.17 | 2.58 | 8.55 | negative | 13 |  |  |
| 1670 | male | 61 | negative | 85.80 | 11.16 | 6.08 | 7.69 | negative | 15 |  |  |
| 1671 | male | 62 | negative | 134.48 | 12.13 | 3.20 | 11.09 | negative | 13 |  |  |
| 1672 | male | 62 | negative | 62.19 | 14.64 | 16.25 | 4.25 | negative | 15 |  |  |
| 1673 | female | 61 | positive | 111.07 | 12.38 | 10.85 | 8.97 | negative | 12 |  |  |
| 1674 | female | 60 | positive | 138.56 | 11.63 | 6.20 | 11.91 | negative | 12 |  |  |
| 1675 | male | 60 | negative | 146.17 | 12.92 | 3.54 | 11.31 | negative | 13 |  |  |
| 1676 | male | 61 | negative | 77.93 | 8.44 | 25.63 | 9.23 | negative | 15 |  |  |
| 1677 | female | 60 | positive | 128.30 | 18.40 | 6.00 | 6.97 | negative | 12 |  |  |
| 1678 | male | 60 | negative | 136.10 | 7.97 | 7.47 | 17.08 | negative | 15 |  |  |
| 1679 | male | 60 | positive | 69.81 | 15.67 | 8.76 | 4.46 | negative | 16 |  |  |
| 1680 | male | 61 | positive | 111.14 | 11.15 | 3.20 | 9.97 | negative | 14 |  |  |
| 1681 | male | 61 | negative | 148.27 | 12.30 | 3.26 | 12.05 | negative | 13 |  |  |
| 1682 | male | 61 | positive | 60.15 | 13.19 | 2.71 | 4.56 | negative | 14 |  |  |
| 1683 | male | 61 | negative | 269.70 | 23.10 | 20.10 | 11.68 | negative | 15 |  |  |
| 1684 | male | 61 | negative | 118.80 | 11.00 | 4.56 | 10.80 | negative | 13 |  |  |
| 1685 | male | 60 | negative | 207.25 | 16.54 | 3.78 | 12.53 | negative | 13 |  |  |
| 1686 | male | 61 | negative | 112.64 | 7.76 | 2.25 | 14.52 | negative | 13 |  |  |
| 1687 | male | 60 | positive | 148.77 | 11.56 | 5.19 | 12.87 | negative | 14 |  |  |
| 1688 | male | 61 | negative | 53.25 | 3.16 | 3.88 | 16.85 | negative | 13 |  |  |
| 1689 | male | 60 | positive | 109.33 | 9.73 | 5.23 | 11.24 | negative | 14 |  |  |
| 1690 | male | 61 | negative | 120.68 | 11.47 | 4.41 | 10.52 | negative | 13 |  |  |
| 1691 | male | 60 | positive | 187.76 | 25.30 | 46.64 | 7.42 | negative | 16 |  |  |
| 1692 | female | 61 | positive | 112.62 | 19.59 | 20.17 | 5.75 | negative | 12 |  |  |
| 1693 | male | 60 | negative | 93.38 | 6.82 | 3.81 | 13.69 | negative | 13 |  |  |
| 1694 | male | 61 | negative | 87.89 | 4.56 | 2.31 | 19.27 | negative | 13 |  |  |
| 1695 | male | 61 | positive | 179.32 | 28.29 | 3.41 | 6.34 | negative | 14 |  |  |
| 1696 | male | 61 | negative | 27.26 | 9.58 | 3.27 | 2.85 | negative | 16 |  |  |
| 1697 | male | 61 | positive | 243.18 | 24.96 | 1.73 | 9.74 | negative | 14 |  |  |
| 1698 | male | 61 | negative | 190.13 | 17.68 | 2.91 | 10.75 | negative | 13 |  |  |
| 1699 | male | 61 | positive | 141.50 | 14.93 | 2.68 | 9.48 | negative | 14 |  |  |
| 1700 | female | 61 | positive | 214.98 | 24.25 | 22.52 | 8.87 | negative | 12 |  |  |
| 1701 | male | 61 | positive | 69.03 | 14.75 | 1.96 | 4.68 | negative | 14 |  |  |
| 1702 | male | 61 | positive | 80.35 | 17.47 | 4.35 | 4.60 | negative | 14 |  |  |
| 1703 | male | 61 | positive | 126.70 | 14.04 | 10.98 | 9.02 | negative | 16 |  |  |
| 1704 | male | 61 | negative | 116.97 | 8.19 | 2.41 | 14.28 | negative | 13 |  |  |
| 1705 | male | 61 | positive | 46.18 | 6.94 | 14.08 | 6.65 | negative | 16 |  |  |
| 1706 | male | 61 | positive | 161.41 | 16.13 | 4.49 | 10.01 | negative | 14 |  |  |
| 1707 | male | 61 | positive | 150.60 | 14.60 | 3.90 | 10.32 | negative | 14 |  |  |
| 1708 | male | 61 | positive | 71.80 | 15.40 | 17.50 | 4.66 | negative | 16 |  |  |
| 1709 | male | 61 | negative | 80.83 | 8.77 | 2.60 | 9.22 | negative | 13 |  |  |
| 1710 | male | 60 | negative | 199.53 | 23.37 | 6.43 | 8.54 | negative | 15 |  |  |
| 1711 | male | 61 | positive | 173.86 | 7.87 | 2.71 | 22.09 | negative | 14 |  |  |
| 1712 | male | 61 | negative | 70.38 | 8.09 | 2.29 | 8.70 | negative | 13 |  |  |
| 1713 | male | 61 | negative | 127.52 | 4.85 | 2.09 | 26.29 | negative | 13 |  |  |
| 1714 | male | 61 | positive | 75.80 | 12.50 | 3.40 | 6.06 | negative | 14 |  |  |
| 1715 | male | 61 | negative | 44.17 | 13.38 | 36.71 | 3.30 | negative | 18 |  |  |
| 1716 | male | 61 | negative | 288.39 | 20.40 | 3.47 | 14.14 | negative | 13 |  |  |
| 1717 | female | 61 | positive | 151.59 | 21.75 | 15.23 | 6.97 | negative | 12 |  |  |
| 1718 | male | 61 | negative | 125.83 | 6.70 | 2.50 | 18.78 | negative | 13 |  |  |
| 1719 | male | 61 | positive | 71.39 | 6.54 | 3.33 | 10.92 | negative | 14 |  |  |
| 1720 | male | 60 | negative | 124.71 | 7.81 | 3.45 | 15.97 | negative | 13 |  |  |
| 1721 | male | 61 | negative | 115.06 | 10.11 | 2.70 | 11.38 | negative | 13 |  |  |
| 1722 | male | 60 | negative | 128.55 | 6.42 | 4.81 | 20.02 | negative | 13 |  |  |
| 1723 | male | 60 | negative | 75.10 | 16.39 | 8.56 | 4.58 | negative | 15 |  |  |
| 1724 | male | 61 | negative | 147.92 | 15.21 | 3.83 | 9.73 | negative | 13 |  |  |
| 1725 | female | 60 | positive | 127.47 | 14.30 | 7.14 | 8.91 | negative | 12 |  |  |
| 1726 | male | 60 | positive | 135.31 | 20.84 | 6.22 | 6.49 | negative | 16 |  |  |
| 1727 | female | 60 | positive | 97.85 | 20.33 | 8.84 | 4.81 | negative | 12 |  |  |
| 1728 | male | 60 | negative | 286.34 | 45.16 | 6.01 | 6.34 | negative | 15 |  |  |
| 1729 | male | 61 | negative | 50.66 | 4.18 | 2.74 | 12.12 | negative | 13 |  |  |
| 1730 | male | 60 | negative | 146.59 | 6.69 | 3.53 | 21.91 | negative | 13 |  |  |
| 1731 | male | 60 | positive | 76.78 | 10.97 | 6.34 | 7.00 | negative | 16 |  |  |
| 1732 | male | 61 | positive | 119.21 | 16.57 | 2.35 | 7.19 | negative | 14 |  |  |
| 1733 | female | 61 | positive | 50.22 | 15.84 | 1.60 | 3.17 | negative | 13 |  |  |
| 1734 | male | 61 | negative | 87.06 | 7.09 | 2.61 | 12.28 | negative | 13 |  |  |
| 1735 | male | 61 | negative | 123.48 | 7.41 | 1.75 | 16.66 | negative | 13 |  |  |
| 1736 | male | 60 | negative | 215.64 | 13.50 | 4.87 | 15.97 | negative | 13 |  |  |
| 1737 | male | 60 | positive | 113.26 | 16.63 | 45.37 | 6.81 | negative | 16 |  |  |
| 1738 | male | 60 | positive | 74.08 | 10.67 | 3.60 | 6.94 | negative | 14 |  |  |
| 1739 | male | 61 | positive | 108.80 | 8.77 | 13.77 | 12.41 | negative | 16 |  |  |
| 1740 | male | 61 | negative | 82.12 | 8.21 | 3.99 | 10.00 | negative | 13 |  |  |
| 1741 | male | 61 | negative | 38.55 | 17.95 | 3.69 | 2.15 | negative | 16 |  |  |
| 1742 | female | 60 | positive | 189.99 | 14.71 | 7.28 | 12.92 | negative | 12 |  |  |
| 1743 | male | 60 | positive | 221.53 | 10.44 | 6.21 | 21.22 | negative | 16 |  |  |
| 1744 | male | 61 | positive | 124.01 | 11.63 | 5.14 | 10.66 | negative | 14 |  |  |
| 1745 | female | 60 | positive | 84.18 | 7.01 | 6.44 | 12.01 | negative | 12 |  |  |
| 1746 | male | 61 | negative | 44.02 | 4.11 | 2.86 | 10.71 | negative | 13 |  |  |
| 1747 | male | 60 | negative | 110.92 | 8.17 | 7.25 | 13.58 | negative | 15 |  |  |
| 1748 | female | 60 | positive | 181.69 | 21.14 | 5.80 | 8.59 | negative | 12 |  |  |
| 1749 | male | 60 | negative | 76.04 | 32.94 | 58.64 | 2.31 | negative | 18 |  |  |
| 1750 | male | 61 | negative | 103.27 | 9.13 | 4.39 | 11.31 | negative | 13 |  |  |
| 1751 | male | 60 | positive | 267.35 | 34.26 | 6.98 | 7.80 | negative | 16 |  |  |
| 1752 | female | 61 | positive | 126.60 | 19.00 | 12.10 | 6.66 | negative | 12 |  |  |
| 1753 | male | 60 | positive | 137.47 | 20.76 | 5.05 | 6.62 | negative | 14 |  |  |
| 1754 | male | 61 | negative | 75.54 | 5.28 | 2.71 | 14.31 | negative | 13 |  |  |
| 1755 | male | 61 | negative | 160.74 | 10.55 | 1.60 | 15.24 | negative | 13 |  |  |
| 1756 | male | 60 | positive | 173.39 | 23.74 | 8.05 | 7.30 | negative | 16 |  |  |
| 1757 | male | 61 | negative | 158.21 | 13.01 | 2.18 | 12.16 | negative | 13 |  |  |
| 1758 | female | 60 | positive | 157.60 | 45.27 | 9.38 | 3.48 | negative | 15 |  |  |
| 1759 | male | 61 | negative | 87.36 | 6.73 | 2.56 | 12.98 | negative | 13 |  |  |
| 1760 | male | 60 | positive | 124.71 | 8.54 | 9.29 | 14.60 | negative | 16 |  |  |
| 1761 | male | 61 | positive | 148.47 | 14.76 | 2.15 | 10.06 | negative | 14 |  |  |
| 1762 | male | 60 | negative | 52.08 | 2.87 | 58.34 | 18.15 | negative | 15 |  |  |
| 1763 | male | 61 | positive | 130.06 | 7.81 | 13.73 | 16.65 | negative | 16 |  |  |
| 1764 | male | 60 | positive | 156.82 | 18.47 | 6.17 | 8.49 | negative | 16 |  |  |
| 1765 | male | 61 | negative | 300.00 | 23.05 | 15.86 | 13.02 | negative | 15 |  |  |
| 1766 | male | 61 | negative | 107.04 | 7.85 | 1.94 | 13.64 | negative | 13 |  |  |
| 1767 | male | 61 | negative | 89.28 | 13.65 | 2.53 | 6.54 | negative | 13 |  |  |
| 1768 | female | 61 | positive | 157.70 | 22.48 | 10.17 | 7.02 | negative | 12 |  |  |
| 1769 | male | 61 | positive | 42.76 | 11.03 | 1.61 | 3.88 | negative | 17 |  |  |
| 1770 | male | 60 | negative | 136.44 | 12.40 | 8.10 | 11.00 | negative | 15 |  |  |
| 1771 | male | 61 | negative | 182.12 | 14.07 | 5.11 | 12.94 | negative | 13 |  |  |
| 1772 | male | 61 | negative | 84.83 | 6.46 | 2.74 | 13.13 | negative | 13 |  |  |
| 1773 | female | 60 | positive | 118.12 | 13.84 | 7.59 | 8.53 | negative | 12 |  |  |
| 1774 | male | 60 | negative | 141.66 | 10.80 | 8.59 | 13.12 | negative | 15 |  |  |
| 1775 | male | 61 | negative | 112.10 | 12.50 | 1.80 | 8.97 | negative | 13 |  |  |
| 1776 | male | 61 | negative | 208.92 | 13.60 | 2.95 | 15.36 | negative | 13 |  |  |
| 1777 | male | 61 | positive | 96.91 | 12.21 | 3.11 | 7.94 | negative | 14 |  |  |
| 1778 | male | 60 | negative | 72.86 | 3.58 | 4.02 | 20.35 | negative | 13 |  |  |
| 1779 | female | 60 | positive | 242.77 | 21.98 | 9.36 | 11.05 | negative | 12 |  |  |
| 1780 | male | 61 | positive | 106.03 | 12.31 | 18.11 | 8.61 | negative | 16 |  |  |
| 1781 | female | 61 | positive | 171.96 | 21.68 | 38.59 | 7.93 | negative | 12 |  |  |
| 1782 | male | 61 | negative | 73.36 | 5.58 | 1.79 | 13.15 | negative | 13 |  |  |
| 1783 | male | 60 | negative | 194.54 | 16.30 | 3.82 | 11.93 | negative | 13 |  |  |
| 1784 | male | 61 | positive | 148.64 | 17.16 | 3.96 | 8.66 | negative | 14 |  |  |
| 1785 | male | 60 | negative | 55.94 | 23.73 | 59.64 | 2.36 | negative | 18 |  |  |
| 1786 | male | 61 | negative | 136.39 | 8.43 | 4.55 | 16.18 | negative | 13 |  |  |
| 1787 | male | 60 | negative | 227.68 | 13.00 | 9.86 | 17.51 | negative | 15 |  |  |
| 1788 | male | 61 | positive | 138.92 | 16.97 | 5.27 | 8.19 | negative | 14 |  |  |
| 1789 | male | 61 | positive | 191.14 | 14.42 | 13.08 | 13.26 | negative | 16 |  |  |
| 1790 | male | 60 | positive | 109.29 | 13.42 | 4.13 | 8.14 | negative | 14 |  |  |
| 1791 | male | 61 | negative | 143.00 | 23.40 | 16.20 | 6.11 | negative | 15 |  |  |
| 1792 | male | 61 | negative | 58.56 | 4.95 | 1.52 | 11.83 | negative | 13 |  |  |
| 1793 | male | 61 | negative | 122.80 | 14.40 | 2.50 | 8.53 | negative | 13 |  |  |
| 1794 | male | 61 | negative | 268.89 | 22.13 | 27.27 | 12.15 | negative | 15 |  |  |
| 1795 | male | 61 | positive | 225.88 | 20.83 | 3.84 | 10.84 | negative | 14 |  |  |
| 1796 | male | 60 | negative | 241.52 | 14.69 | 6.62 | 16.44 | negative | 15 |  |  |
| 1797 | male | 60 | negative | 110.50 | 14.30 | 6.78 | 7.73 | negative | 15 |  |  |
| 1798 | male | 61 | negative | 97.53 | 12.16 | 2.05 | 8.02 | negative | 13 |  |  |
| 1799 | female | 61 | positive | 83.89 | 24.85 | 15.62 | 3.38 | negative | 15 |  |  |
| 1800 | female | 61 | positive | 260.54 | 25.92 | 13.96 | 10.05 | negative | 12 |  |  |
| 1801 | male | 61 | negative | 84.67 | 7.16 | 3.73 | 11.83 | negative | 13 |  |  |
| 1802 | male | 61 | negative | 117.23 | 12.27 | 2.57 | 9.55 | negative | 13 |  |  |
| 1803 | male | 60 | positive | 148.48 | 15.23 | 5.18 | 9.75 | negative | 14 |  |  |
| 1804 | female | 60 | positive | 213.60 | 44.86 | 8.82 | 4.76 | negative | 12 |  |  |
| 1805 | male | 61 | negative | 78.58 | 7.46 | 5.04 | 10.53 | negative | 13 |  |  |
| 1806 | male | 61 | negative | 113.06 | 11.29 | 1.98 | 10.01 | negative | 13 |  |  |
| 1807 | male | 60 | negative | 121.29 | 9.85 | 3.38 | 12.31 | negative | 13 |  |  |
| 1808 | male | 61 | negative | 120.31 | 9.59 | 2.76 | 12.55 | negative | 13 |  |  |
| 1809 | male | 60 | negative | 58.39 | 6.67 | 6.53 | 8.75 | negative | 15 |  |  |
| 1810 | male | 61 | negative | 126.68 | 15.58 | 3.65 | 8.13 | negative | 13 |  |  |
| 1811 | female | 60 | positive | 92.19 | 22.29 | 5.80 | 4.14 | negative | 12 |  |  |
| 1812 | male | 60 | negative | 108.87 | 16.55 | 5.04 | 6.58 | negative | 13 |  |  |
| 1813 | male | 61 | negative | 53.10 | 5.40 | 1.80 | 9.83 | negative | 13 |  |  |
| 1814 | male | 60 | positive | 156.27 | 22.94 | 4.68 | 6.81 | negative | 14 |  |  |
| 1815 | male | 61 | negative | 98.10 | 11.40 | 2.60 | 8.61 | negative | 13 |  |  |
| 1816 | male | 61 | negative | 111.16 | 10.53 | 4.14 | 10.56 | negative | 13 |  |  |
| 1817 | male | 59 | negative | 148.51 | 15.66 | 5.27 | 9.48 | negative | 12 |  |  |
| 1818 | male | 60 | negative | 135.52 | 12.61 | 2.96 | 10.75 | negative | 13 |  |  |
| 1819 | male | 59 | negative | 106.91 | 7.96 | 7.33 | 13.43 | negative | 14 |  |  |
| 1820 | male | 59 | negative | 151.39 | 17.86 | 3.30 | 8.48 | negative | 12 |  |  |
| 1821 | male | 60 | positive | 300.00 | 44.00 | 11.21 | 6.82 | negative | 16 |  |  |
| 1822 | male | 59 | negative | 70.90 | 5.20 | 6.10 | 13.63 | negative | 14 |  |  |
| 1823 | male | 60 | positive | 199.63 | 20.32 | 10.92 | 9.82 | negative | 16 |  |  |
| 1824 | male | 60 | positive | 291.32 | 23.39 | 15.52 | 12.45 | negative | 16 |  |  |
| 1825 | male | 60 | negative | 282.00 | 11.86 | 2.20 | 23.78 | negative | 13 |  |  |
| 1826 | male | 60 | negative | 82.69 | 6.35 | 1.82 | 13.02 | negative | 13 |  |  |
| 1827 | male | 59 | positive | 192.85 | 18.19 | 5.03 | 10.60 | negative | 13 |  |  |
| 1828 | male | 59 | negative | 98.82 | 5.38 | 2.54 | 18.37 | negative | 12 |  |  |
| 1829 | male | 59 | negative | 87.68 | 10.29 | 2.63 | 8.52 | negative | 12 |  |  |
| 1830 | male | 60 | negative | 128.83 | 11.38 | 2.97 | 11.32 | negative | 13 |  |  |
| 1831 | male | 60 | negative | 106.58 | 9.19 | 3.23 | 11.60 | negative | 13 |  |  |
| 1832 | male | 60 | negative | 49.21 | 4.76 | 2.24 | 10.34 | negative | 13 |  |  |
| 1833 | male | 59 | negative | 124.34 | 9.08 | 6.27 | 13.69 | negative | 14 |  |  |
| 1834 | male | 60 | negative | 168.89 | 15.52 | 2.88 | 10.88 | negative | 13 |  |  |
| 1835 | male | 59 | negative | 90.14 | 8.88 | 4.48 | 10.15 | negative | 12 |  |  |
| 1836 | male | 60 | positive | 125.88 | 16.65 | 3.34 | 7.56 | negative | 14 |  |  |
| 1837 | male | 59 | negative | 100.94 | 7.87 | 2.97 | 12.83 | negative | 12 |  |  |
| 1838 | male | 60 | negative | 177.20 | 7.25 | 2.28 | 24.44 | negative | 13 |  |  |
| 1839 | male | 59 | negative | 90.14 | 13.98 | 6.63 | 6.45 | negative | 14 |  |  |
| 1840 | male | 60 | positive | 57.49 | 14.36 | 12.14 | 4.00 | negative | 16 |  |  |
| 1841 | male | 59 | positive | 142.13 | 17.21 | 6.06 | 8.26 | negative | 15 |  |  |
| 1842 | male | 59 | negative | 53.19 | 7.19 | 2.59 | 7.40 | negative | 12 |  |  |
| 1843 | male | 59 | positive | 242.36 | 29.06 | 2.82 | 8.34 | negative | 13 |  |  |
| 1844 | male | 59 | negative | 103.72 | 8.48 | 5.53 | 12.23 | negative | 12 |  |  |
| 1845 | male | 60 | negative | 105.04 | 10.28 | 19.35 | 10.22 | negative | 15 |  |  |
| 1846 | male | 59 | positive | 191.34 | 18.75 | 4.53 | 10.20 | negative | 13 |  |  |
| 1847 | male | 59 | positive | 169.96 | 13.72 | 4.57 | 12.39 | negative | 13 |  |  |
| 1848 | male | 60 | negative | 91.24 | 7.82 | 1.59 | 11.67 | negative | 13 |  |  |
| 1849 | male | 59 | negative | 300.00 | 42.93 | 24.43 | 6.99 | negative | 14 |  |  |
| 1850 | male | 60 | negative | 92.25 | 9.01 | 3.28 | 10.24 | negative | 13 |  |  |
| 1851 | male | 59 | negative | 48.92 | 7.57 | 4.71 | 6.46 | negative | 12 |  |  |
| 1852 | male | 60 | negative | 227.33 | 12.09 | 1.59 | 18.80 | negative | 13 |  |  |
| 1853 | male | 59 | negative | 111.78 | 11.53 | 4.24 | 9.69 | negative | 12 |  |  |
| 1854 | male | 60 | negative | 127.26 | 6.05 | 2.12 | 21.03 | negative | 13 |  |  |
| 1855 | male | 59 | negative | 144.29 | 6.28 | 3.10 | 22.98 | negative | 12 |  |  |
| 1856 | male | 60 | positive | 116.83 | 11.60 | 1.64 | 10.07 | negative | 14 |  |  |
| 1857 | male | 59 | positive | 112.63 | 14.99 | 5.97 | 7.51 | negative | 15 |  |  |
| 1858 | male | 59 | positive | 136.18 | 17.97 | 7.33 | 7.58 | negative | 15 |  |  |
| 1859 | male | 60 | negative | 125.83 | 7.79 | 2.87 | 16.15 | negative | 13 |  |  |
| 1860 | male | 59 | negative | 123.21 | 14.74 | 6.53 | 8.36 | negative | 14 |  |  |
| 1861 | male | 60 | positive | 116.74 | 8.56 | 1.69 | 13.64 | negative | 14 |  |  |
| 1862 | male | 59 | negative | 112.20 | 12.60 | 5.60 | 8.90 | negative | 12 |  |  |
| 1863 | male | 59 | positive | 155.25 | 19.57 | 9.40 | 7.93 | negative | 15 |  |  |
| 1864 | male | 60 | negative | 191.36 | 11.15 | 1.53 | 17.16 | negative | 13 |  |  |
| 1865 | male | 60 | positive | 217.27 | 16.58 | 2.30 | 13.10 | negative | 14 |  |  |
| 1866 | male | 60 | negative | 45.92 | 1.38 | 1.85 | 33.28 | negative | 13 |  |  |
| 1867 | male | 59 | positive | 72.07 | 8.90 | 3.27 | 8.10 | negative | 13 |  |  |
| 1868 | female | 60 | negative | 92.90 | 30.40 | 13.60 | 3.06 | negative | 14 |  |  |
| 1869 | male | 60 | positive | 234.56 | 30.77 | 12.28 | 7.62 | negative | 16 |  |  |
| 1870 | male | 60 | negative | 139.72 | 10.03 | 2.75 | 13.93 | negative | 13 |  |  |
| 1871 | male | 59 | negative | 79.68 | 11.02 | 3.81 | 7.23 | negative | 12 |  |  |
| 1872 | female | 60 | positive | 93.52 | 17.76 | 10.83 | 5.27 | negative | 12 |  |  |
| 1873 | male | 59 | positive | 170.68 | 25.88 | 8.49 | 6.60 | negative | 15 |  |  |
| 1874 | male | 59 | positive | 100.64 | 12.93 | 2.86 | 7.78 | negative | 13 |  |  |
| 1875 | male | 59 | negative | 99.62 | 11.42 | 3.48 | 8.72 | negative | 12 |  |  |
| 1876 | male | 60 | negative | 43.92 | 7.12 | 3.33 | 6.17 | negative | 13 |  |  |
| 1877 | male | 59 | positive | 107.61 | 16.48 | 7.67 | 6.53 | negative | 15 |  |  |
| 1878 | male | 60 | negative | 120.40 | 11.33 | 29.57 | 10.63 | negative | 15 |  |  |
| 1879 | male | 59 | positive | 163.65 | 18.49 | 4.07 | 8.85 | negative | 13 |  |  |
| 1880 | male | 59 | positive | 146.68 | 16.96 | 2.79 | 8.65 | negative | 13 |  |  |
| 1881 | male | 59 | negative | 141.00 | 7.59 | 3.45 | 18.58 | negative | 12 |  |  |
| 1882 | male | 60 | positive | 106.41 | 8.69 | 12.23 | 12.25 | negative | 16 |  |  |
| 1883 | male | 60 | negative | 216.26 | 8.58 | 3.10 | 25.21 | negative | 13 |  |  |
| 1884 | male | 59 | negative | 83.22 | 6.61 | 3.10 | 12.59 | negative | 12 |  |  |
| 1885 | male | 59 | positive | 99.96 | 9.30 | 3.05 | 10.75 | negative | 13 |  |  |
| 1886 | male | 60 | negative | 192.06 | 14.75 | 2.32 | 13.02 | negative | 13 |  |  |
| 1887 | female | 59 | negative | 45.67 | 16.85 | 43.49 | 2.71 | negative | 13 |  |  |
| 1888 | male | 59 | negative | 151.01 | 9.22 | 4.77 | 16.38 | negative | 12 |  |  |
| 1889 | male | 60 | negative | 103.70 | 5.75 | 2.99 | 18.03 | negative | 13 |  |  |
| 1890 | male | 59 | negative | 113.77 | 6.17 | 4.58 | 18.44 | negative | 12 |  |  |
| 1891 | male | 59 | positive | 137.50 | 21.60 | 6.00 | 6.37 | negative | 15 |  |  |
| 1892 | male | 60 | negative | 52.99 | 3.69 | 10.10 | 14.36 | negative | 15 |  |  |
| 1893 | male | 59 | positive | 92.50 | 20.80 | 8.60 | 4.45 | negative | 15 |  |  |
| 1894 | male | 59 | positive | 197.78 | 21.51 | 5.51 | 9.19 | negative | 13 |  |  |
| 1895 | male | 59 | negative | 191.58 | 10.93 | 3.18 | 17.53 | negative | 12 |  |  |
| 1896 | male | 60 | positive | 165.43 | 17.43 | 12.56 | 9.49 | negative | 16 |  |  |
| 1897 | male | 59 | positive | 74.50 | 14.17 | 20.66 | 5.26 | negative | 15 |  |  |
| 1898 | male | 59 | negative | 158.47 | 10.67 | 3.33 | 14.85 | negative | 12 |  |  |
| 1899 | male | 60 | positive | 60.07 | 14.98 | 11.41 | 4.01 | negative | 16 |  |  |
| 1900 | male | 60 | negative | 25.81 | 2.80 | 3.37 | 9.22 | negative | 13 |  |  |
| 1901 | male | 60 | negative | 107.31 | 10.30 | 1.77 | 10.42 | negative | 13 |  |  |
| 1902 | male | 60 | negative | 177.30 | 11.60 | 1.90 | 15.28 | negative | 13 |  |  |
| 1903 | male | 60 | positive | 98.79 | 14.62 | 3.13 | 6.76 | negative | 14 |  |  |
| 1904 | male | 60 | negative | 74.66 | 5.46 | 1.50 | 13.67 | negative | 13 |  |  |
| 1905 | male | 59 | negative | 147.39 | 12.08 | 3.29 | 12.20 | negative | 12 |  |  |
| 1906 | male | 59 | positive | 292.38 | 23.89 | 9.99 | 12.24 | negative | 15 |  |  |
| 1907 | male | 60 | negative | 107.53 | 9.56 | 10.39 | 11.25 | negative | 15 |  |  |
| 1908 | male | 60 | negative | 136.19 | 7.58 | 3.11 | 17.97 | negative | 13 |  |  |
| 1909 | male | 60 | negative | 275.68 | 28.03 | 11.51 | 9.84 | negative | 15 |  |  |
| 1910 | male | 60 | negative | 119.32 | 6.30 | 3.04 | 18.94 | negative | 13 |  |  |
| 1911 | male | 59 | negative | 151.95 | 9.97 | 2.62 | 15.24 | negative | 12 |  |  |
| 1912 | male | 59 | positive | 83.47 | 32.25 | 6.13 | 2.59 | negative | 18 |  |  |
| 1913 | male | 60 | positive | 91.35 | 21.61 | 12.53 | 4.23 | negative | 16 |  |  |
| 1914 | male | 59 | positive | 108.39 | 12.99 | 4.99 | 8.34 | negative | 13 |  |  |
| 1915 | male | 59 | positive | 89.63 | 6.62 | 8.02 | 13.54 | negative | 15 |  |  |
| 1916 | male | 60 | positive | 217.03 | 22.17 | 10.96 | 9.79 | negative | 16 |  |  |
| 1917 | male | 59 | negative | 59.89 | 7.26 | 4.42 | 8.25 | negative | 12 |  |  |
| 1918 | male | 60 | negative | 289.92 | 21.13 | 2.43 | 13.72 | negative | 13 |  |  |
| 1919 | male | 59 | negative | 84.58 | 7.34 | 5.44 | 11.52 | negative | 12 |  |  |
| 1920 | male | 60 | negative | 135.08 | 21.67 | 3.04 | 6.23 | negative | 13 |  |  |
| 1921 | male | 59 | negative | 67.43 | 6.78 | 3.20 | 9.95 | negative | 12 |  |  |
| 1922 | male | 59 | positive | 141.89 | 19.54 | 5.76 | 7.26 | negative | 15 |  |  |
| 1923 | male | 60 | positive | 201.43 | 21.53 | 20.53 | 9.36 | negative | 16 |  |  |
| 1924 | male | 60 | negative | 132.20 | 14.40 | 11.00 | 9.18 | negative | 15 |  |  |
| 1925 | female | 60 | positive | 139.31 | 27.51 | 15.53 | 5.06 | negative | 12 |  |  |
| 1926 | male | 59 | negative | 111.03 | 8.54 | 3.26 | 13.00 | negative | 12 |  |  |
| 1927 | male | 60 | negative | 121.18 | 6.12 | 3.24 | 19.80 | negative | 13 |  |  |
| 1928 | male | 60 | positive | 87.95 | 2.52 | 14.35 | 34.90 | negative | 16 |  |  |
| 1929 | female | 60 | positive | 118.40 | 18.65 | 12.08 | 6.35 | negative | 12 |  |  |
| 1930 | male | 60 | negative | 79.76 | 7.52 | 2.87 | 10.61 | negative | 13 |  |  |
| 1931 | male | 60 | positive | 69.63 | 11.53 | 2.15 | 6.04 | negative | 14 |  |  |
| 1932 | male | 60 | positive | 129.48 | 13.72 | 2.95 | 9.44 | negative | 14 |  |  |
| 1933 | female | 60 | positive | 77.90 | 14.90 | 11.30 | 5.23 | negative | 12 |  |  |
| 1934 | male | 59 | positive | 291.26 | 16.94 | 7.94 | 17.19 | negative | 15 |  |  |
| 1935 | female | 60 | positive | 134.23 | 29.34 | 10.41 | 4.57 | negative | 12 |  |  |
| 1936 | male | 59 | negative | 203.17 | 9.54 | 10.27 | 21.30 | negative | 14 |  |  |
| 1937 | male | 58 | positive | 92.14 | 9.63 | 5.64 | 9.57 | negative | 13 |  |  |
| 1938 | male | 58 | positive | 96.05 | 8.69 | 3.17 | 11.05 | negative | 13 |  |  |
| 1939 | male | 58 | negative | 260.29 | 38.08 | 20.47 | 6.84 | negative | 14 |  |  |
| 1940 | male | 59 | negative | 125.20 | 24.92 | 12.96 | 5.02 | negative | 14 |  |  |
| 1941 | female | 58 | negative | 100.03 | 30.85 | 17.35 | 3.24 | negative | 13 |  |  |
| 1942 | male | 58 | positive | 74.00 | 13.40 | 5.10 | 5.52 | negative | 13 |  |  |
| 1943 | male | 59 | negative | 90.13 | 5.01 | 2.49 | 17.99 | negative | 12 |  |  |
| 1944 | male | 58 | positive | 143.44 | 13.05 | 4.25 | 10.99 | negative | 13 |  |  |
| 1945 | male | 58 | positive | 133.96 | 20.88 | 2.71 | 6.42 | negative | 13 |  |  |
| 1946 | male | 58 | positive | 122.33 | 10.60 | 3.36 | 11.54 | negative | 13 |  |  |
| 1947 | male | 58 | negative | 82.26 | 6.63 | 3.79 | 12.41 | negative | 12 |  |  |
| 1948 | male | 58 | negative | 122.81 | 11.90 | 6.24 | 10.32 | negative | 14 |  |  |
| 1949 | male | 59 | positive | 126.50 | 8.89 | 19.23 | 14.23 | negative | 15 |  |  |
| 1950 | male | 58 | negative | 136.26 | 7.88 | 2.43 | 17.29 | negative | 12 |  |  |
| 1951 | male | 58 | negative | 205.14 | 12.00 | 6.08 | 17.09 | negative | 14 |  |  |
| 1952 | male | 58 | negative | 114.28 | 7.52 | 3.13 | 15.20 | negative | 12 |  |  |
| 1953 | male | 58 | negative | 130.61 | 9.62 | 3.21 | 13.58 | negative | 12 |  |  |
| 1954 | male | 58 | negative | 140.42 | 6.73 | 3.13 | 20.86 | negative | 12 |  |  |
| 1955 | male | 58 | positive | 299.41 | 26.23 | 7.81 | 11.41 | negative | 15 |  |  |
| 1956 | male | 58 | negative | 75.39 | 5.93 | 4.60 | 12.71 | negative | 12 |  |  |
| 1957 | male | 58 | positive | 89.04 | 9.26 | 5.75 | 9.62 | negative | 15 |  |  |
| 1958 | female | 58 | negative | 84.20 | 24.34 | 57.61 | 3.46 | negative | 13 |  |  |
| 1959 | male | 58 | negative | 78.40 | 12.20 | 4.80 | 6.43 | negative | 12 |  |  |
| 1960 | male | 58 | negative | 90.83 | 7.80 | 2.04 | 11.64 | negative | 12 |  |  |
| 1961 | male | 58 | positive | 81.75 | 11.21 | 8.67 | 7.29 | negative | 15 |  |  |
| 1962 | male | 59 | negative | 101.54 | 7.86 | 1.66 | 12.92 | negative | 12 |  |  |
| 1963 | male | 58 | positive | 123.45 | 13.27 | 5.65 | 9.30 | negative | 13 |  |  |
| 1964 | male | 58 | negative | 99.10 | 30.00 | 29.30 | 3.30 | negative | 17 |  |  |
| 1965 | male | 58 | negative | 162.91 | 11.38 | 2.71 | 14.32 | negative | 12 |  |  |
| 1966 | male | 58 | positive | 81.57 | 7.83 | 3.14 | 10.42 | negative | 13 |  |  |
| 1967 | male | 59 | positive | 71.58 | 6.57 | 1.62 | 10.89 | negative | 13 |  |  |
| 1968 | male | 58 | negative | 173.83 | 9.45 | 3.14 | 18.39 | negative | 12 |  |  |
| 1969 | male | 58 | positive | 115.33 | 10.78 | 3.61 | 10.70 | negative | 13 |  |  |
| 1970 | male | 58 | negative | 58.79 | 7.54 | 2.06 | 7.80 | negative | 12 |  |  |
| 1971 | male | 59 | negative | 137.45 | 12.33 | 1.87 | 11.15 | negative | 12 |  |  |
| 1972 | male | 58 | negative | 187.53 | 8.98 | 3.70 | 20.88 | negative | 12 |  |  |
| 1973 | male | 58 | negative | 21.55 | 7.04 | 42.14 | 3.06 | negative | 17 |  |  |
| 1974 | male | 58 | positive | 145.06 | 20.46 | 7.91 | 7.09 | negative | 15 |  |  |
| 1975 | male | 58 | negative | 194.01 | 24.80 | 4.17 | 7.82 | negative | 12 |  |  |
| 1976 | male | 58 | positive | 74.92 | 9.00 | 3.34 | 8.32 | negative | 13 |  |  |
| 1977 | male | 58 | negative | 87.14 | 6.94 | 3.37 | 12.56 | negative | 12 |  |  |
| 1978 | male | 58 | negative | 70.76 | 8.13 | 6.88 | 8.70 | negative | 14 |  |  |
| 1979 | male | 59 | negative | 68.52 | 3.97 | 2.53 | 17.26 | negative | 12 |  |  |
| 1980 | male | 58 | positive | 108.02 | 13.73 | 5.00 | 7.87 | negative | 13 |  |  |
| 1981 | male | 59 | negative | 77.60 | 9.80 | 1.50 | 7.92 | negative | 12 |  |  |
| 1982 | male | 58 | negative | 184.05 | 12.21 | 2.50 | 15.07 | negative | 12 |  |  |
| 1983 | male | 58 | positive | 52.80 | 6.62 | 2.94 | 7.98 | negative | 13 |  |  |
| 1984 | male | 58 | negative | 287.29 | 22.03 | 5.02 | 13.04 | negative | 12 |  |  |
| 1985 | male | 58 | negative | 120.25 | 9.09 | 6.04 | 13.23 | negative | 14 |  |  |
| 1986 | male | 59 | negative | 69.83 | 8.87 | 2.40 | 7.87 | negative | 12 |  |  |
| 1987 | male | 59 | negative | 97.96 | 6.76 | 2.37 | 14.49 | negative | 12 |  |  |
| 1988 | male | 58 | positive | 171.39 | 17.55 | 5.44 | 9.77 | negative | 13 |  |  |
| 1989 | male | 58 | positive | 141.41 | 15.47 | 2.22 | 9.14 | negative | 13 |  |  |
| 1990 | male | 58 | positive | 192.52 | 19.02 | 8.22 | 10.12 | negative | 15 |  |  |
| 1991 | male | 58 | positive | 137.21 | 11.19 | 9.72 | 12.26 | negative | 15 |  |  |
| 1992 | male | 58 | negative | 71.85 | 8.24 | 3.16 | 8.72 | negative | 12 |  |  |
| 1993 | male | 58 | positive | 70.21 | 16.78 | 4.86 | 4.18 | negative | 13 |  |  |
| 1994 | male | 58 | negative | 61.34 | 6.83 | 2.35 | 8.98 | negative | 12 |  |  |
| 1995 | male | 58 | positive | 199.21 | 24.09 | 4.97 | 8.27 | negative | 13 |  |  |
| 1996 | male | 59 | negative | 50.67 | 7.08 | 2.07 | 7.16 | negative | 12 |  |  |
| 1997 | male | 58 | positive | 104.31 | 9.21 | 5.28 | 11.33 | negative | 13 |  |  |
| 1998 | male | 59 | negative | 100.69 | 8.78 | 1.79 | 11.47 | negative | 12 |  |  |
| 1999 | male | 58 | negative | 81.20 | 4.70 | 3.07 | 17.28 | negative | 12 |  |  |
| 2000 | male | 58 | positive | 136.90 | 11.72 | 4.40 | 11.68 | negative | 13 |  |  |
| 2001 | male | 58 | negative | 104.30 | 9.34 | 2.04 | 11.17 | negative | 12 |  |  |
| 2002 | male | 58 | negative | 89.54 | 7.55 | 2.02 | 11.86 | negative | 12 |  |  |
| 2003 | male | 58 | positive | 126.22 | 10.63 | 3.43 | 11.87 | negative | 13 |  |  |
| 2004 | male | 58 | negative | 143.67 | 7.12 | 4.96 | 20.18 | negative | 12 |  |  |
| 2005 | male | 58 | positive | 266.48 | 33.15 | 3.59 | 8.04 | negative | 13 |  |  |
| 2006 | male | 58 | negative | 59.67 | 6.76 | 2.68 | 8.83 | negative | 12 |  |  |
| 2007 | male | 58 | negative | 173.64 | 13.63 | 3.81 | 12.74 | negative | 12 |  |  |
| 2008 | male | 58 | negative | 106.47 | 5.39 | 2.52 | 19.75 | negative | 12 |  |  |
| 2009 | male | 58 | negative | 24.17 | 10.20 | 58.65 | 2.37 | negative | 17 |  |  |
| 2010 | male | 59 | positive | 86.30 | 15.60 | 11.60 | 5.53 | negative | 15 |  |  |
| 2011 | female | 58 | negative | 96.81 | 30.25 | 20.19 | 3.20 | negative | 13 |  |  |
| 2012 | male | 58 | negative | 84.98 | 3.84 | 3.30 | 22.13 | negative | 12 |  |  |
| 2013 | male | 58 | positive | 141.04 | 24.12 | 11.91 | 5.85 | negative | 15 |  |  |
| 2014 | male | 58 | negative | 226.96 | 22.43 | 7.70 | 10.12 | negative | 14 |  |  |
| 2015 | male | 58 | negative | 124.54 | 8.72 | 5.52 | 14.28 | negative | 12 |  |  |
| 2016 | male | 58 | negative | 131.61 | 13.75 | 2.35 | 9.57 | negative | 12 |  |  |
| 2017 | male | 58 | negative | 67.27 | 10.67 | 2.60 | 6.30 | negative | 12 |  |  |
| 2018 | male | 58 | negative | 117.31 | 8.80 | 2.16 | 13.33 | negative | 12 |  |  |
| 2019 | male | 58 | positive | 179.73 | 26.59 | 4.26 | 6.76 | negative | 13 |  |  |
| 2020 | male | 58 | positive | 117.79 | 13.78 | 7.53 | 8.55 | negative | 15 |  |  |
| 2021 | male | 58 | positive | 16.72 | 8.07 | 60.00 | 2.07 | negative | 18 |  |  |
| 2022 | male | 58 | positive | 219.35 | 26.35 | 19.88 | 8.32 | negative | 15 |  |  |
| 2023 | male | 59 | positive | 300.00 | 27.65 | 14.90 | 10.85 | negative | 15 |  |  |
| 2024 | male | 58 | negative | 114.47 | 12.48 | 3.88 | 9.17 | negative | 12 |  |  |
| 2025 | male | 58 | negative | 182.40 | 11.94 | 2.98 | 15.28 | negative | 12 |  |  |
| 2026 | male | 59 | negative | 203.85 | 13.56 | 1.54 | 15.03 | negative | 12 |  |  |
| 2027 | male | 59 | positive | 121.76 | 19.73 | 14.99 | 6.17 | negative | 15 |  |  |
| 2028 | male | 58 | positive | 89.96 | 27.21 | 6.07 | 3.31 | negative | 18 |  |  |
| 2029 | male | 59 | negative | 52.27 | 2.69 | 1.78 | 19.43 | negative | 12 |  |  |
| 2030 | male | 59 | positive | 157.75 | 11.84 | 14.02 | 13.32 | negative | 15 |  |  |
| 2031 | male | 58 | negative | 72.10 | 7.20 | 2.50 | 10.01 | negative | 12 |  |  |
| 2032 | male | 58 | negative | 100.34 | 6.95 | 2.47 | 14.44 | negative | 12 |  |  |
| 2033 | male | 58 | negative | 66.62 | 10.03 | 13.83 | 6.64 | negative | 14 |  |  |
| 2034 | male | 58 | positive | 139.60 | 17.40 | 4.10 | 8.02 | negative | 13 |  |  |
| 2035 | female | 58 | negative | 19.11 | 11.86 | 60.00 | 1.61 | negative | 13 |  |  |
| 2036 | male | 58 | positive | 43.67 | 9.54 | 2.59 | 4.58 | negative | 13 |  |  |
| 2037 | male | 59 | positive | 223.16 | 19.02 | 14.39 | 11.73 | negative | 15 |  |  |
| 2038 | male | 58 | negative | 115.13 | 8.40 | 4.90 | 13.71 | negative | 12 |  |  |
| 2039 | male | 58 | negative | 99.35 | 8.04 | 7.26 | 12.36 | negative | 14 |  |  |
| 2040 | male | 58 | positive | 108.18 | 17.23 | 6.53 | 6.28 | negative | 15 |  |  |
| 2041 | male | 58 | positive | 103.53 | 14.41 | 7.52 | 7.18 | negative | 15 |  |  |
| 2042 | male | 58 | negative | 158.50 | 21.50 | 14.70 | 7.37 | negative | 14 |  |  |
| 2043 | male | 58 | positive | 101.53 | 16.93 | 13.94 | 6.00 | negative | 15 |  |  |
| 2044 | male | 58 | negative | 88.45 | 5.53 | 2.25 | 15.99 | negative | 12 |  |  |
| 2045 | male | 58 | negative | 113.26 | 8.35 | 3.37 | 13.56 | negative | 12 |  |  |
| 2046 | male | 58 | positive | 177.82 | 15.06 | 9.72 | 11.81 | negative | 15 |  |  |
| 2047 | male | 58 | negative | 131.50 | 15.56 | 4.29 | 8.45 | negative | 12 |  |  |
| 2048 | male | 58 | negative | 78.30 | 4.76 | 2.05 | 16.45 | negative | 12 |  |  |
| 2049 | male | 58 | negative | 238.07 | 14.71 | 2.04 | 16.18 | negative | 12 |  |  |
| 2050 | male | 59 | negative | 99.24 | 11.35 | 10.92 | 8.74 | negative | 14 |  |  |
| 2051 | male | 58 | negative | 23.36 | 4.29 | 60.00 | 5.45 | negative | 14 |  |  |
| 2052 | male | 59 | positive | 207.16 | 15.14 | 10.00 | 13.68 | negative | 15 |  |  |
| 2053 | male | 58 | negative | 118.66 | 7.94 | 2.32 | 14.94 | negative | 12 |  |  |
| 2054 | male | 58 | negative | 297.77 | 15.73 | 22.02 | 18.93 | negative | 14 |  |  |
| 2055 | male | 58 | negative | 161.70 | 7.02 | 3.40 | 23.03 | negative | 12 |  |  |
| 2056 | male | 58 | negative | 156.48 | 12.05 | 3.30 | 12.99 | negative | 12 |  |  |
| 2057 | male | 58 | positive | 93.47 | 6.01 | 6.88 | 15.55 | negative | 15 |  |  |
| 2058 | male | 58 | positive | 63.97 | 4.86 | 5.07 | 13.16 | negative | 13 |  |  |
| 2059 | male | 58 | positive | 114.49 | 14.90 | 5.84 | 7.68 | negative | 15 |  |  |
| 2060 | male | 59 | negative | 74.78 | 10.02 | 1.95 | 7.46 | negative | 12 |  |  |
| 2061 | male | 58 | negative | 44.11 | 9.63 | 3.97 | 4.58 | negative | 12 |  |  |
| 2062 | male | 58 | positive | 83.58 | 9.21 | 5.63 | 9.07 | negative | 13 |  |  |
| 2063 | male | 58 | negative | 168.12 | 9.52 | 2.42 | 17.66 | negative | 12 |  |  |
| 2064 | male | 58 | negative | 97.97 | 9.57 | 3.70 | 10.24 | negative | 12 |  |  |
| 2065 | male | 59 | positive | 122.19 | 16.18 | 15.86 | 7.55 | negative | 15 |  |  |
| 2066 | male | 58 | negative | 124.93 | 6.92 | 2.03 | 18.05 | negative | 12 |  |  |
| 2067 | male | 59 | negative | 96.53 | 8.31 | 1.94 | 11.62 | negative | 12 |  |  |
| 2068 | male | 58 | negative | 115.94 | 12.35 | 3.86 | 9.39 | negative | 12 |  |  |
| 2069 | male | 58 | positive | 90.65 | 14.54 | 9.49 | 6.23 | negative | 15 |  |  |
| 2070 | male | 58 | negative | 98.98 | 5.09 | 2.37 | 19.45 | negative | 12 |  |  |
| 2071 | male | 58 | positive | 119.50 | 13.90 | 15.60 | 8.60 | negative | 15 |  |  |
| 2072 | male | 58 | positive | 13.03 | 18.28 | 48.33 | 0.71 | negative | 18 |  |  |
| 2073 | male | 58 | positive | 135.65 | 11.32 | 8.26 | 11.98 | negative | 15 |  |  |
| 2074 | male | 58 | negative | 132.10 | 13.16 | 4.88 | 10.04 | negative | 12 |  |  |
| 2075 | male | 58 | negative | 74.70 | 6.03 | 2.43 | 12.39 | negative | 12 |  |  |
| 2076 | male | 59 | negative | 86.90 | 14.30 | 19.70 | 6.08 | negative | 14 |  |  |
| 2077 | male | 58 | positive | 265.35 | 14.09 | 12.21 | 18.83 | negative | 15 |  |  |
| 2078 | male | 58 | negative | 148.50 | 11.03 | 15.26 | 13.46 | negative | 14 |  |  |
| 2079 | male | 58 | positive | 155.75 | 19.83 | 4.52 | 7.85 | negative | 13 |  |  |
| 2080 | male | 58 | positive | 140.35 | 10.23 | 5.09 | 13.72 | negative | 13 |  |  |
| 2081 | male | 58 | positive | 90.30 | 9.50 | 6.30 | 9.51 | negative | 15 |  |  |
| 2082 | male | 57 | negative | 59.06 | 7.74 | 41.03 | 7.63 | negative | 14 |  |  |
| 2083 | male | 57 | positive | 140.87 | 29.58 | 4.67 | 4.76 | negative | 13 |  |  |
| 2084 | male | 57 | negative | 58.52 | 4.21 | 8.36 | 13.90 | negative | 14 |  |  |
| 2085 | male | 57 | negative | 132.46 | 17.28 | 58.91 | 7.67 | negative | 14 |  |  |
| 2086 | male | 57 | negative | 78.23 | 5.45 | 5.15 | 14.35 | negative | 12 |  |  |
| 2087 | male | 57 | positive | 245.30 | 19.70 | 7.54 | 12.45 | negative | 15 |  |  |
| 2088 | male | 57 | positive | 98.66 | 11.57 | 6.68 | 8.53 | negative | 15 |  |  |
| 2089 | male | 58 | negative | 126.71 | 13.36 | 1.75 | 9.48 | negative | 12 |  |  |
| 2090 | male | 57 | negative | 207.85 | 13.85 | 4.48 | 15.01 | negative | 12 |  |  |
| 2091 | male | 57 | negative | 127.04 | 10.67 | 5.06 | 11.91 | negative | 12 |  |  |
| 2092 | male | 57 | positive | 100.86 | 13.67 | 7.54 | 7.38 | negative | 15 |  |  |
| 2093 | male | 58 | positive | 178.29 | 16.63 | 10.01 | 10.72 | negative | 15 |  |  |
| 2094 | male | 57 | negative | 174.76 | 18.16 | 5.27 | 9.62 | negative | 12 |  |  |
| 2095 | male | 57 | negative | 187.94 | 9.80 | 9.19 | 19.18 | negative | 14 |  |  |
| 2096 | male | 57 | positive | 143.05 | 17.21 | 4.84 | 8.31 | negative | 13 |  |  |
| 2097 | male | 58 | negative | 153.91 | 8.49 | 1.71 | 18.13 | negative | 12 |  |  |
| 2098 | male | 57 | positive | 98.25 | 11.18 | 5.75 | 8.79 | negative | 15 |  |  |
| 2099 | male | 57 | negative | 285.08 | 16.28 | 5.80 | 17.51 | negative | 14 |  |  |
| 2100 | male | 57 | negative | 115.56 | 13.28 | 6.57 | 8.70 | negative | 14 |  |  |
| 2101 | male | 57 | positive | 147.12 | 16.25 | 5.90 | 9.05 | negative | 15 |  |  |
| 2102 | male | 57 | positive | 143.64 | 12.10 | 6.97 | 11.87 | negative | 15 |  |  |
| 2103 | male | 58 | positive | 94.30 | 12.89 | 1.80 | 7.32 | negative | 13 |  |  |
| 2104 | male | 58 | negative | 61.60 | 10.10 | 1.60 | 6.10 | negative | 12 |  |  |
| 2105 | male | 57 | positive | 225.11 | 25.04 | 8.24 | 8.99 | negative | 15 |  |  |
| 2106 | male | 58 | positive | 129.30 | 9.61 | 10.11 | 13.45 | negative | 15 |  |  |
| 2107 | male | 57 | negative | 115.20 | 13.91 | 8.31 | 8.28 | negative | 14 |  |  |
| 2108 | male | 57 | positive | 203.50 | 20.50 | 8.20 | 9.93 | negative | 15 |  |  |
| 2109 | male | 57 | negative | 112.41 | 9.25 | 6.02 | 12.15 | negative | 14 |  |  |
| 2110 | female | 57 | positive | 81.86 | 24.02 | 9.08 | 3.41 | negative | 14 |  |  |
| 2111 | male | 57 | positive | 191.06 | 16.96 | 8.33 | 11.27 | negative | 15 |  |  |
| 2112 | male | 57 | positive | 118.01 | 15.30 | 7.92 | 7.71 | negative | 15 |  |  |
| 2113 | male | 57 | positive | 127.82 | 13.29 | 7.63 | 9.62 | negative | 15 |  |  |
| 2114 | male | 57 | positive | 105.39 | 7.74 | 5.14 | 13.62 | negative | 13 |  |  |
| 2115 | female | 57 | positive | 102.06 | 26.74 | 4.84 | 3.82 | negative | 12 |  |  |
| 2116 | male | 57 | negative | 166.59 | 9.67 | 7.12 | 17.23 | negative | 14 |  |  |
| 2117 | male | 57 | negative | 83.56 | 8.72 | 6.88 | 9.58 | negative | 14 |  |  |
| 2118 | male | 57 | negative | 116.78 | 5.89 | 4.73 | 19.83 | negative | 12 |  |  |
| 2119 | male | 57 | negative | 49.99 | 12.20 | 8.65 | 4.10 | negative | 14 |  |  |
| 2120 | male | 58 | negative | 73.80 | 3.15 | 1.93 | 23.43 | negative | 12 |  |  |
| 2121 | male | 58 | negative | 133.90 | 6.58 | 1.81 | 20.35 | negative | 12 |  |  |
| 2122 | male | 58 | negative | 124.52 | 10.75 | 1.52 | 11.58 | negative | 12 |  |  |
| 2123 | male | 57 | positive | 255.65 | 24.07 | 9.70 | 10.62 | negative | 15 |  |  |
| 2124 | male | 57 | positive | 194.33 | 21.47 | 5.24 | 9.05 | negative | 13 |  |  |
| 2125 | male | 57 | positive | 300.00 | 28.79 | 8.30 | 10.42 | negative | 15 |  |  |
| 2126 | male | 58 | positive | 130.31 | 20.07 | 10.36 | 6.49 | negative | 15 |  |  |
| 2127 | male | 57 | positive | 82.33 | 8.88 | 5.52 | 9.27 | negative | 13 |  |  |
| 2128 | male | 57 | negative | 218.06 | 10.80 | 5.62 | 20.19 | negative | 12 |  |  |
| 2129 | male | 57 | positive | 185.74 | 16.90 | 6.29 | 10.99 | negative | 15 |  |  |
| 2130 | male | 58 | positive | 94.28 | 9.09 | 10.55 | 10.37 | negative | 15 |  |  |
| 2131 | male | 57 | negative | 58.62 | 4.25 | 5.10 | 13.79 | negative | 12 |  |  |
| 2132 | male | 57 | positive | 127.85 | 20.88 | 6.76 | 6.12 | negative | 15 |  |  |
| 2133 | male | 57 | positive | 64.26 | 9.87 | 7.40 | 6.51 | negative | 15 |  |  |
| 2134 | male | 57 | negative | 249.21 | 16.29 | 5.76 | 15.30 | negative | 14 |  |  |
| 2135 | male | 57 | negative | 140.05 | 9.10 | 6.06 | 15.39 | negative | 14 |  |  |
| 2136 | male | 57 | negative | 133.28 | 20.90 | 6.70 | 6.38 | negative | 14 |  |  |
| 2137 | male | 57 | positive | 180.12 | 26.52 | 9.59 | 6.79 | negative | 15 |  |  |
| 2138 | male | 57 | negative | 127.83 | 6.88 | 4.42 | 18.58 | negative | 12 |  |  |
| 2139 | male | 57 | positive | 123.42 | 18.84 | 7.05 | 6.55 | negative | 15 |  |  |
| 2140 | male | 57 | negative | 128.10 | 11.20 | 8.90 | 11.44 | negative | 14 |  |  |
| 2141 | male | 57 | positive | 161.00 | 19.70 | 5.00 | 8.17 | negative | 13 |  |  |
| 2142 | male | 58 | negative | 138.08 | 11.13 | 1.51 | 12.41 | negative | 12 |  |  |
| 2143 | male | 57 | positive | 300.00 | 22.66 | 7.10 | 13.24 | negative | 15 |  |  |
| 2144 | male | 58 | negative | 116.54 | 8.69 | 1.83 | 13.41 | negative | 12 |  |  |
| 2145 | male | 57 | positive | 90.49 | 8.34 | 7.00 | 10.85 | negative | 15 |  |  |
| 2146 | male | 57 | negative | 293.64 | 13.26 | 9.99 | 22.14 | negative | 14 |  |  |
| 2147 | male | 57 | negative | 67.73 | 1.77 | 53.60 | 38.27 | negative | 14 |  |  |
| 2148 | male | 57 | negative | 121.53 | 25.01 | 43.38 | 4.86 | negative | 14 |  |  |
| 2149 | male | 57 | negative | 95.57 | 4.38 | 9.34 | 21.82 | negative | 14 |  |  |
| 2150 | female | 57 | negative | 38.96 | 11.91 | 58.39 | 3.27 | negative | 13 |  |  |
| 2151 | male | 57 | negative | 267.56 | 14.62 | 5.00 | 18.30 | negative | 12 |  |  |
| 2152 | male | 57 | positive | 231.09 | 18.48 | 8.58 | 12.50 | negative | 15 |  |  |
| 2153 | male | 57 | positive | 273.09 | 28.41 | 5.20 | 9.61 | negative | 13 |  |  |
| 2154 | male | 57 | positive | 138.29 | 18.61 | 8.03 | 7.43 | negative | 15 |  |  |
| 2155 | male | 57 | negative | 155.50 | 13.05 | 5.43 | 11.92 | negative | 12 |  |  |
| 2156 | male | 57 | negative | 79.37 | 5.18 | 4.73 | 15.32 | negative | 12 |  |  |
| 2157 | male | 57 | negative | 125.00 | 14.41 | 5.75 | 8.67 | negative | 14 |  |  |
| 2158 | male | 57 | negative | 300.00 | 22.13 | 7.90 | 13.56 | negative | 14 |  |  |
| 2159 | male | 57 | negative | 78.63 | 15.41 | 4.73 | 5.10 | negative | 12 |  |  |
| 2160 | male | 57 | negative | 300.00 | 28.68 | 8.62 | 10.46 | negative | 14 |  |  |
| 2161 | male | 57 | negative | 128.93 | 5.39 | 4.45 | 23.92 | negative | 12 |  |  |
| 2162 | male | 57 | positive | 82.73 | 9.65 | 5.66 | 8.57 | negative | 13 |  |  |
| 2163 | male | 57 | negative | 93.67 | 7.88 | 4.69 | 11.89 | negative | 12 |  |  |
| 2164 | male | 57 | negative | 129.32 | 12.19 | 5.61 | 10.61 | negative | 12 |  |  |
| 2165 | male | 57 | positive | 78.80 | 15.53 | 8.74 | 5.07 | negative | 15 |  |  |
| 2166 | female | 57 | negative | 18.78 | 9.05 | 41.42 | 2.08 | negative | 13 |  |  |
| 2167 | male | 57 | positive | 104.71 | 17.45 | 9.82 | 6.00 | negative | 15 |  |  |
| 2168 | male | 58 | negative | 85.20 | 9.70 | 1.50 | 8.78 | negative | 12 |  |  |
| 2169 | male | 57 | positive | 139.79 | 15.43 | 8.87 | 9.06 | negative | 15 |  |  |
| 2170 | male | 57 | negative | 73.18 | 3.62 | 4.98 | 20.22 | negative | 12 |  |  |
| 2171 | male | 57 | positive | 60.40 | 14.63 | 6.12 | 4.13 | negative | 15 |  |  |
| 2172 | female | 57 | positive | 28.08 | 16.15 | 5.47 | 1.74 | negative | 12 |  |  |
| 2173 | male | 57 | positive | 102.06 | 8.35 | 6.58 | 12.22 | negative | 15 |  |  |
| 2174 | male | 57 | negative | 174.99 | 19.74 | 8.04 | 8.86 | negative | 14 |  |  |
| 2175 | male | 57 | negative | 102.88 | 21.77 | 7.52 | 4.73 | negative | 14 |  |  |
| 2176 | male | 58 | negative | 185.16 | 13.20 | 1.83 | 14.03 | negative | 12 |  |  |
| 2177 | male | 57 | positive | 159.08 | 14.04 | 6.72 | 11.33 | negative | 15 |  |  |
| 2178 | male | 57 | negative | 146.04 | 9.46 | 5.02 | 15.44 | negative | 12 |  |  |
| 2179 | male | 58 | negative | 71.47 | 9.09 | 1.96 | 7.86 | negative | 12 |  |  |
| 2180 | male | 57 | negative | 152.70 | 7.17 | 5.32 | 21.30 | negative | 12 |  |  |
| 2181 | male | 58 | negative | 93.43 | 6.47 | 1.64 | 14.44 | negative | 12 |  |  |
| 2182 | male | 57 | positive | 128.48 | 12.63 | 7.68 | 10.17 | negative | 15 |  |  |
| 2183 | male | 58 | positive | 206.36 | 16.79 | 1.64 | 12.29 | negative | 13 |  |  |
| 2184 | female | 57 | negative | 14.19 | 9.38 | 45.56 | 1.51 | negative | 13 |  |  |
| 2185 | male | 57 | positive | 127.80 | 20.00 | 9.50 | 6.39 | negative | 15 |  |  |
| 2186 | male | 57 | positive | 124.59 | 15.26 | 8.44 | 8.16 | negative | 15 |  |  |
| 2187 | male | 57 | positive | 135.89 | 14.94 | 4.70 | 9.10 | negative | 13 |  |  |
| 2188 | male | 57 | negative | 83.42 | 7.81 | 4.33 | 10.68 | negative | 12 |  |  |
| 2189 | male | 57 | negative | 133.12 | 9.72 | 9.01 | 13.70 | negative | 14 |  |  |
| 2190 | male | 57 | negative | 98.30 | 13.04 | 6.97 | 7.54 | negative | 14 |  |  |
| 2191 | male | 57 | negative | 110.25 | 8.64 | 5.81 | 12.76 | negative | 14 |  |  |
| 2192 | female | 57 | negative | 11.34 | 8.16 | 56.39 | 1.39 | negative | 13 |  |  |
| 2193 | male | 57 | positive | 121.53 | 16.46 | 9.35 | 7.38 | negative | 15 |  |  |
| 2194 | male | 58 | negative | 96.60 | 13.95 | 10.86 | 6.92 | negative | 14 |  |  |
| 2195 | male | 57 | negative | 81.91 | 8.18 | 6.38 | 10.01 | negative | 14 |  |  |
| 2196 | male | 57 | positive | 158.81 | 13.84 | 5.56 | 11.47 | negative | 13 |  |  |
| 2197 | male | 57 | negative | 47.23 | 3.58 | 5.33 | 13.19 | negative | 12 |  |  |
| 2198 | male | 57 | positive | 188.30 | 18.50 | 7.05 | 10.18 | negative | 15 |  |  |
| 2199 | male | 57 | negative | 169.21 | 11.69 | 7.29 | 14.47 | negative | 14 |  |  |
| 2200 | male | 58 | negative | 212.19 | 11.81 | 1.82 | 17.97 | negative | 12 |  |  |
| 2201 | male | 57 | negative | 70.12 | 7.15 | 6.31 | 9.81 | negative | 14 |  |  |
| 2202 | male | 58 | negative | 77.20 | 4.60 | 1.80 | 16.78 | negative | 12 |  |  |
| 2203 | male | 57 | negative | 99.40 | 13.00 | 4.70 | 7.65 | negative | 12 |  |  |
| 2204 | male | 57 | positive | 300.00 | 41.69 | 49.97 | 7.20 | negative | 15 |  |  |
| 2205 | male | 57 | positive | 89.52 | 5.72 | 7.97 | 15.65 | negative | 15 |  |  |
| 2206 | male | 57 | negative | 183.91 | 10.54 | 4.33 | 17.45 | negative | 12 |  |  |
| 2207 | male | 58 | negative | 78.55 | 3.47 | 1.67 | 22.64 | negative | 12 |  |  |
| 2208 | male | 57 | positive | 189.21 | 7.64 | 9.38 | 24.77 | negative | 15 |  |  |
| 2209 | male | 57 | positive | 156.64 | 33.57 | 42.20 | 4.67 | negative | 15 |  |  |
| 2210 | male | 57 | positive | 282.46 | 25.75 | 7.14 | 10.97 | negative | 15 |  |  |
| 2211 | male | 57 | positive | 167.67 | 20.41 | 9.28 | 8.22 | negative | 15 |  |  |
| 2212 | male | 57 | positive | 223.00 | 18.56 | 8.92 | 12.02 | negative | 15 |  |  |
| 2213 | male | 57 | negative | 86.32 | 11.84 | 4.35 | 7.29 | negative | 12 |  |  |
| 2214 | male | 57 | negative | 73.20 | 10.10 | 4.60 | 7.25 | negative | 12 |  |  |
| 2215 | male | 57 | positive | 141.33 | 15.73 | 5.90 | 8.98 | negative | 15 |  |  |
| 2216 | male | 57 | negative | 106.43 | 5.43 | 4.40 | 19.60 | negative | 12 |  |  |
| 2217 | male | 57 | negative | 89.20 | 6.40 | 6.81 | 13.94 | negative | 14 |  |  |
| 2218 | male | 57 | negative | 291.62 | 20.35 | 9.37 | 14.33 | negative | 14 |  |  |
| 2219 | male | 57 | positive | 128.91 | 23.68 | 9.19 | 5.44 | negative | 15 |  |  |
| 2220 | male | 57 | negative | 117.80 | 9.10 | 5.40 | 12.95 | negative | 12 |  |  |
| 2221 | female | 57 | negative | 17.22 | 7.69 | 59.37 | 2.24 | negative | 13 |  |  |
| 2222 | male | 57 | negative | 107.13 | 13.73 | 7.79 | 7.80 | negative | 14 |  |  |
| 2223 | male | 57 | positive | 225.87 | 23.21 | 4.42 | 9.73 | negative | 13 |  |  |
| 2224 | male | 57 | negative | 201.52 | 21.71 | 6.33 | 9.28 | negative | 14 |  |  |
| 2225 | male | 57 | negative | 88.61 | 9.44 | 5.07 | 9.39 | negative | 12 |  |  |
| 2226 | male | 57 | negative | 114.06 | 11.44 | 5.71 | 9.97 | negative | 14 |  |  |
| 2227 | male | 58 | negative | 281.94 | 29.81 | 11.16 | 9.46 | negative | 14 |  |  |
| 2228 | male | 57 | positive | 117.29 | 13.97 | 9.99 | 8.40 | negative | 15 |  |  |
| 2229 | male | 57 | negative | 86.32 | 6.18 | 4.76 | 13.97 | negative | 12 |  |  |
| 2230 | male | 57 | negative | 150.50 | 42.00 | 9.30 | 3.58 | negative | 17 |  |  |
| 2231 | male | 57 | negative | 122.89 | 11.25 | 7.41 | 10.92 | negative | 14 |  |  |
| 2232 | male | 57 | negative | 107.55 | 10.21 | 3.97 | 10.53 | negative | 12 |  |  |
| 2233 | male | 57 | negative | 300.00 | 39.30 | 4.27 | 7.63 | negative | 12 |  |  |
| 2234 | male | 57 | negative | 83.81 | 9.12 | 2.21 | 9.19 | negative | 12 |  |  |
| 2235 | male | 57 | positive | 216.10 | 25.10 | 3.10 | 8.61 | negative | 13 |  |  |
| 2236 | male | 57 | negative | 92.46 | 14.30 | 3.68 | 6.47 | negative | 12 |  |  |
| 2237 | male | 57 | negative | 105.50 | 7.05 | 3.04 | 14.96 | negative | 12 |  |  |
| 2238 | male | 57 | negative | 179.05 | 13.76 | 4.18 | 13.01 | negative | 12 |  |  |
| 2239 | male | 57 | negative | 64.84 | 4.31 | 2.87 | 15.04 | negative | 12 |  |  |
| 2240 | male | 57 | negative | 152.17 | 12.52 | 2.36 | 12.15 | negative | 12 |  |  |
| 2241 | male | 57 | negative | 105.38 | 9.94 | 2.55 | 10.60 | negative | 12 |  |  |
| 2242 | male | 57 | negative | 251.30 | 25.79 | 27.44 | 9.74 | negative | 14 |  |  |
| 2243 | male | 57 | positive | 69.36 | 10.70 | 2.57 | 6.48 | negative | 13 |  |  |
| 2244 | male | 57 | negative | 185.49 | 14.65 | 3.85 | 12.66 | negative | 12 |  |  |
| 2245 | male | 57 | negative | 89.78 | 6.85 | 3.16 | 13.11 | negative | 12 |  |  |
| 2246 | male | 57 | positive | 141.45 | 11.37 | 2.54 | 12.44 | negative | 13 |  |  |
| 2247 | male | 57 | positive | 91.74 | 14.47 | 2.99 | 6.34 | negative | 13 |  |  |
| 2248 | male | 57 | negative | 175.40 | 34.10 | 25.80 | 5.14 | negative | 14 |  |  |
| 2249 | male | 57 | negative | 102.33 | 8.89 | 3.30 | 11.51 | negative | 12 |  |  |
| 2250 | male | 57 | positive | 62.48 | 7.21 | 3.18 | 8.67 | negative | 13 |  |  |
| 2251 | male | 57 | negative | 58.75 | 3.21 | 3.33 | 18.30 | negative | 12 |  |  |
| 2252 | male | 57 | negative | 124.39 | 7.86 | 3.27 | 15.83 | negative | 12 |  |  |
| 2253 | male | 57 | negative | 133.29 | 4.82 | 2.23 | 27.65 | negative | 12 |  |  |
| 2254 | male | 57 | positive | 180.98 | 31.27 | 23.61 | 5.79 | negative | 15 |  |  |
| 2255 | male | 57 | negative | 115.22 | 5.86 | 2.57 | 19.66 | negative | 12 |  |  |
| 2256 | male | 57 | negative | 73.21 | 11.25 | 2.57 | 6.51 | negative | 12 |  |  |
| 2257 | male | 57 | negative | 71.90 | 5.70 | 2.40 | 12.61 | negative | 12 |  |  |
| 2258 | male | 57 | negative | 158.33 | 12.97 | 3.34 | 12.21 | negative | 12 |  |  |
| 2259 | male | 57 | negative | 120.92 | 9.23 | 3.74 | 13.10 | negative | 12 |  |  |
| 2260 | male | 57 | negative | 80.41 | 4.91 | 2.35 | 16.38 | negative | 12 |  |  |
| 2261 | male | 57 | negative | 189.88 | 10.37 | 3.29 | 18.31 | negative | 12 |  |  |
| 2262 | male | 57 | negative | 137.52 | 9.29 | 3.83 | 14.80 | negative | 12 |  |  |
| 2263 | male | 57 | negative | 132.15 | 10.89 | 2.32 | 12.13 | negative | 12 |  |  |
| 2264 | male | 57 | negative | 300.00 | 31.71 | 36.86 | 9.46 | negative | 14 |  |  |
| 2265 | male | 57 | negative | 104.41 | 9.10 | 3.46 | 11.47 | negative | 12 |  |  |
| 2266 | male | 57 | negative | 66.45 | 4.30 | 2.35 | 15.45 | negative | 12 |  |  |
| 2267 | male | 57 | positive | 257.24 | 39.06 | 25.83 | 6.59 | negative | 15 |  |  |
| 2268 | male | 57 | negative | 140.63 | 10.99 | 2.76 | 12.80 | negative | 12 |  |  |
| 2269 | male | 57 | negative | 278.63 | 20.75 | 2.31 | 13.43 | negative | 12 |  |  |
| 2270 | male | 57 | negative | 112.60 | 11.60 | 2.80 | 9.71 | negative | 12 |  |  |
| 2271 | male | 57 | positive | 175.30 | 18.08 | 3.61 | 9.70 | negative | 13 |  |  |
| 2272 | male | 57 | negative | 105.43 | 11.41 | 4.19 | 9.24 | negative | 12 |  |  |
| 2273 | male | 57 | negative | 143.89 | 10.48 | 3.99 | 13.73 | negative | 12 |  |  |
| 2274 | male | 57 | negative | 146.21 | 12.74 | 2.34 | 11.48 | negative | 12 |  |  |
| 2275 | male | 57 | positive | 83.48 | 7.76 | 3.54 | 10.76 | negative | 13 |  |  |
| 2276 | male | 57 | negative | 132.70 | 12.80 | 4.00 | 10.37 | negative | 12 |  |  |
| 2277 | male | 57 | negative | 78.15 | 5.10 | 4.27 | 15.32 | negative | 12 |  |  |
| 2278 | male | 57 | negative | 93.88 | 7.68 | 3.23 | 12.22 | negative | 12 |  |  |
| 2279 | male | 57 | negative | 89.93 | 8.00 | 3.63 | 11.24 | negative | 12 |  |  |
| 2280 | male | 57 | negative | 120.12 | 7.51 | 2.55 | 15.99 | negative | 12 |  |  |
| 2281 | male | 57 | negative | 60.27 | 4.52 | 2.60 | 13.33 | negative | 12 |  |  |
| 2282 | male | 57 | negative | 98.07 | 22.64 | 30.48 | 4.33 | negative | 14 |  |  |
| 2283 | male | 57 | positive | 70.58 | 9.54 | 33.65 | 7.40 | negative | 15 |  |  |
| 2284 | male | 57 | negative | 69.28 | 5.70 | 2.22 | 12.15 | negative | 12 |  |  |
| 2285 | male | 57 | negative | 84.45 | 11.50 | 2.90 | 7.34 | negative | 12 |  |  |
| 2286 | male | 57 | negative | 144.54 | 12.03 | 3.93 | 12.01 | negative | 12 |  |  |
| 2287 | female | 57 | positive | 80.65 | 23.12 | 30.11 | 3.49 | negative | 14 |  |  |
| 2288 | male | 57 | negative | 143.28 | 13.06 | 2.84 | 10.97 | negative | 12 |  |  |
| 2289 | male | 57 | negative | 67.07 | 5.69 | 3.10 | 11.79 | negative | 12 |  |  |
| 2290 | male | 57 | positive | 106.00 | 30.40 | 28.00 | 3.49 | negative | 18 |  |  |
| 2291 | male | 57 | negative | 92.16 | 6.68 | 2.27 | 13.80 | negative | 12 |  |  |
| 2292 | male | 57 | negative | 46.65 | 3.00 | 2.49 | 15.55 | negative | 12 |  |  |
| 2293 | male | 57 | positive | 120.29 | 11.67 | 2.39 | 10.31 | negative | 13 |  |  |
| 2294 | male | 57 | negative | 119.06 | 11.84 | 3.08 | 10.06 | negative | 12 |  |  |
| 2295 | male | 57 | positive | 143.92 | 14.06 | 3.63 | 10.24 | negative | 13 |  |  |
| 2296 | male | 57 | positive | 95.61 | 11.10 | 3.60 | 8.61 | negative | 13 |  |  |
| 2297 | male | 57 | positive | 158.85 | 12.10 | 3.64 | 13.13 | negative | 13 |  |  |
| 2298 | male | 57 | negative | 86.21 | 6.31 | 2.33 | 13.66 | negative | 12 |  |  |
| 2299 | male | 57 | positive | 95.71 | 7.24 | 3.57 | 13.22 | negative | 13 |  |  |
| 2300 | male | 57 | negative | 86.94 | 4.86 | 3.48 | 17.89 | negative | 12 |  |  |
| 2301 | male | 57 | negative | 58.96 | 12.52 | 2.38 | 4.71 | negative | 12 |  |  |
| 2302 | male | 57 | negative | 103.31 | 9.01 | 3.70 | 11.47 | negative | 12 |  |  |
| 2303 | male | 57 | negative | 128.25 | 10.84 | 4.17 | 11.83 | negative | 12 |  |  |
| 2304 | male | 57 | positive | 19.35 | 8.21 | 3.49 | 2.36 | negative | 16 |  |  |
| 2305 | male | 57 | positive | 218.45 | 13.60 | 2.67 | 16.06 | negative | 13 |  |  |
| 2306 | male | 57 | negative | 83.59 | 4.94 | 2.59 | 16.92 | negative | 12 |  |  |
| 2307 | male | 57 | negative | 76.68 | 6.33 | 2.29 | 12.11 | negative | 12 |  |  |
| 2308 | male | 57 | negative | 112.08 | 3.60 | 2.41 | 31.13 | negative | 12 |  |  |
| 2309 | male | 57 | negative | 147.29 | 9.77 | 3.07 | 15.08 | negative | 12 |  |  |
| 2310 | male | 57 | negative | 176.30 | 11.62 | 4.06 | 15.17 | negative | 12 |  |  |
| 2311 | male | 57 | positive | 84.65 | 10.84 | 2.23 | 7.81 | negative | 13 |  |  |
| 2312 | male | 57 | negative | 119.28 | 12.74 | 4.02 | 9.36 | negative | 12 |  |  |
| 2313 | male | 57 | negative | 127.56 | 6.38 | 4.06 | 19.99 | negative | 12 |  |  |
| 2314 | male | 57 | positive | 100.91 | 11.01 | 3.11 | 9.17 | negative | 13 |  |  |
| 2315 | male | 57 | negative | 75.20 | 8.90 | 2.50 | 8.45 | negative | 12 |  |  |
| 2316 | male | 57 | positive | 127.37 | 16.92 | 3.72 | 7.53 | negative | 13 |  |  |
| 2317 | male | 57 | negative | 125.93 | 10.49 | 3.44 | 12.00 | negative | 12 |  |  |
| 2318 | male | 57 | positive | 111.41 | 8.11 | 3.41 | 13.74 | negative | 13 |  |  |
| 2319 | male | 57 | negative | 91.39 | 7.65 | 2.70 | 11.95 | negative | 12 |  |  |
| 2320 | male | 57 | negative | 89.19 | 7.71 | 3.00 | 11.57 | negative | 12 |  |  |
| 2321 | male | 57 | negative | 145.85 | 16.71 | 3.55 | 8.73 | negative | 12 |  |  |
| 2322 | male | 57 | negative | 74.80 | 6.70 | 2.50 | 11.16 | negative | 12 |  |  |
| 2323 | male | 57 | positive | 144.13 | 22.50 | 2.47 | 6.41 | negative | 13 |  |  |
| 2324 | male | 57 | negative | 115.33 | 7.18 | 3.35 | 16.06 | negative | 12 |  |  |
| 2325 | male | 57 | negative | 41.89 | 10.97 | 29.99 | 3.82 | negative | 17 |  |  |
| 2326 | male | 57 | negative | 109.56 | 27.60 | 20.75 | 3.97 | negative | 14 |  |  |
| 2327 | male | 57 | positive | 110.66 | 9.97 | 2.76 | 11.10 | negative | 13 |  |  |
| 2328 | male | 57 | negative | 123.52 | 7.25 | 3.42 | 17.04 | negative | 12 |  |  |
| 2329 | male | 57 | negative | 78.10 | 14.40 | 2.80 | 5.42 | negative | 12 |  |  |
| 2330 | male | 57 | positive | 197.79 | 21.76 | 3.15 | 9.09 | negative | 13 |  |  |
| 2331 | male | 57 | negative | 83.51 | 5.52 | 2.93 | 15.13 | negative | 12 |  |  |
| 2332 | male | 57 | negative | 113.98 | 9.24 | 2.97 | 12.34 | negative | 12 |  |  |
| 2333 | male | 57 | positive | 71.70 | 9.70 | 3.90 | 7.39 | negative | 13 |  |  |
| 2334 | male | 57 | negative | 104.80 | 9.40 | 3.40 | 11.15 | negative | 12 |  |  |
| 2335 | male | 57 | negative | 111.72 | 7.68 | 3.49 | 14.55 | negative | 12 |  |  |
| 2336 | male | 57 | negative | 140.09 | 6.26 | 2.41 | 22.38 | negative | 12 |  |  |
| 2337 | male | 57 | positive | 160.46 | 13.00 | 3.18 | 12.34 | negative | 13 |  |  |
| 2338 | male | 57 | negative | 159.54 | 10.34 | 2.89 | 15.43 | negative | 12 |  |  |
| 2339 | male | 57 | negative | 77.50 | 5.21 | 2.26 | 14.88 | negative | 12 |  |  |
| 2340 | male | 57 | negative | 228.05 | 17.36 | 4.13 | 13.14 | negative | 12 |  |  |
| 2341 | male | 57 | positive | 100.58 | 9.47 | 22.36 | 10.62 | negative | 15 |  |  |
| 2342 | male | 57 | positive | 257.56 | 23.68 | 3.04 | 10.88 | negative | 13 |  |  |
| 2343 | male | 57 | positive | 62.93 | 6.05 | 2.37 | 10.40 | negative | 13 |  |  |
| 2344 | male | 57 | negative | 82.84 | 7.22 | 2.45 | 11.47 | negative | 12 |  |  |
| 2345 | male | 57 | positive | 76.08 | 6.04 | 3.71 | 12.60 | negative | 13 |  |  |
| 2346 | male | 57 | negative | 127.26 | 6.59 | 3.07 | 19.31 | negative | 12 |  |  |
| 2347 | male | 57 | negative | 155.42 | 8.33 | 2.72 | 18.66 | negative | 12 |  |  |
| 2348 | male | 57 | negative | 179.15 | 8.66 | 4.09 | 20.69 | negative | 12 |  |  |
| 2349 | male | 57 | negative | 161.50 | 11.40 | 2.60 | 14.17 | negative | 12 |  |  |
| 2350 | male | 57 | negative | 124.22 | 4.45 | 2.25 | 27.91 | negative | 12 |  |  |
| 2351 | male | 57 | negative | 81.85 | 8.40 | 3.33 | 9.74 | negative | 12 |  |  |
| 2352 | male | 57 | positive | 70.00 | 11.20 | 4.20 | 6.25 | negative | 13 |  |  |
| 2353 | male | 57 | negative | 81.35 | 6.80 | 3.06 | 11.96 | negative | 12 |  |  |
| 2354 | male | 57 | negative | 156.53 | 7.89 | 2.48 | 19.84 | negative | 12 |  |  |
| 2355 | male | 57 | negative | 175.61 | 9.58 | 4.26 | 18.33 | negative | 12 |  |  |
| 2356 | male | 57 | negative | 96.52 | 5.52 | 2.42 | 17.49 | negative | 12 |  |  |
| 2357 | male | 57 | positive | 63.94 | 10.80 | 3.55 | 5.92 | negative | 13 |  |  |
| 2358 | male | 57 | positive | 66.71 | 9.47 | 4.12 | 7.04 | negative | 13 |  |  |
| 2359 | male | 57 | negative | 100.66 | 5.89 | 3.08 | 17.09 | negative | 12 |  |  |
| 2360 | male | 57 | negative | 114.80 | 5.62 | 3.19 | 20.43 | negative | 12 |  |  |
| 2361 | male | 57 | negative | 67.64 | 7.90 | 2.63 | 8.56 | negative | 12 |  |  |
| 2362 | male | 57 | negative | 142.69 | 9.54 | 2.63 | 14.96 | negative | 12 |  |  |
| 2363 | male | 57 | positive | 65.63 | 9.65 | 3.64 | 6.80 | negative | 13 |  |  |
| 2364 | male | 57 | negative | 35.72 | 2.14 | 3.08 | 16.69 | negative | 12 |  |  |
| 2365 | male | 57 | positive | 95.45 | 17.08 | 4.14 | 5.59 | negative | 13 |  |  |
| 2366 | male | 57 | negative | 138.79 | 13.24 | 2.25 | 10.48 | negative | 12 |  |  |
| 2367 | male | 57 | negative | 40.37 | 3.97 | 3.44 | 10.17 | negative | 12 |  |  |
| 2368 | male | 57 | negative | 95.35 | 10.46 | 2.55 | 9.12 | negative | 12 |  |  |
| 2369 | male | 57 | negative | 162.00 | 8.17 | 2.02 | 19.83 | negative | 12 |  |  |
| 2370 | male | 57 | positive | 81.77 | 6.86 | 1.93 | 11.92 | negative | 13 |  |  |
| 2371 | male | 57 | negative | 180.87 | 11.64 | 1.87 | 15.54 | negative | 12 |  |  |
| 2372 | female | 56 | positive | 45.24 | 12.13 | 8.37 | 3.73 | negative | 14 |  |  |
| 2373 | female | 57 | positive | 45.24 | 15.28 | 11.60 | 2.96 | negative | 14 |  |  |
| 2374 | male | 57 | positive | 99.54 | 10.19 | 10.03 | 9.77 | negative | 15 |  |  |
| 2375 | male | 57 | positive | 127.58 | 15.87 | 10.59 | 8.04 | negative | 15 |  |  |
| 2376 | male | 57 | negative | 224.79 | 29.51 | 15.43 | 7.62 | negative | 14 |  |  |
| 2377 | male | 57 | positive | 186.73 | 22.70 | 11.82 | 8.23 | negative | 15 |  |  |
| 2378 | male | 56 | negative | 109.82 | 16.80 | 9.39 | 6.54 | negative | 14 |  |  |
| 2379 | male | 57 | negative | 82.94 | 3.71 | 1.97 | 22.36 | negative | 12 |  |  |
| 2380 | male | 57 | positive | 127.02 | 11.95 | 15.88 | 10.63 | negative | 15 |  |  |
| 2381 | male | 57 | negative | 226.35 | 11.30 | 12.15 | 20.03 | negative | 14 |  |  |
| 2382 | female | 57 | positive | 86.26 | 24.51 | 15.92 | 3.52 | negative | 14 |  |  |
| 2383 | male | 57 | negative | 107.60 | 10.60 | 1.85 | 10.15 | negative | 12 |  |  |
| 2384 | male | 57 | negative | 76.92 | 5.29 | 2.03 | 14.54 | negative | 12 |  |  |
| 2385 | male | 57 | negative | 102.60 | 5.60 | 2.16 | 18.32 | negative | 12 |  |  |
| 2386 | male | 57 | negative | 131.50 | 13.70 | 2.10 | 9.60 | negative | 12 |  |  |
| 2387 | male | 57 | positive | 282.63 | 41.26 | 10.08 | 6.85 | negative | 15 |  |  |
| 2388 | male | 57 | negative | 59.61 | 5.96 | 2.15 | 10.00 | negative | 12 |  |  |
| 2389 | male | 57 | positive | 137.43 | 13.74 | 14.14 | 10.00 | negative | 15 |  |  |
| 2390 | male | 57 | negative | 13.67 | 5.54 | 60.00 | 2.47 | negative | 17 |  |  |
| 2391 | male | 57 | negative | 95.10 | 7.42 | 1.95 | 12.82 | negative | 12 |  |  |
| 2392 | male | 57 | negative | 104.93 | 5.94 | 1.70 | 17.66 | negative | 12 |  |  |
| 2393 | male | 57 | positive | 73.61 | 10.69 | 12.77 | 6.89 | negative | 15 |  |  |
| 2394 | male | 57 | negative | 56.83 | 6.26 | 12.57 | 9.08 | negative | 14 |  |  |
| 2395 | male | 57 | negative | 86.31 | 7.71 | 1.86 | 11.19 | negative | 12 |  |  |
| 2396 | male | 57 | positive | 141.01 | 9.91 | 1.72 | 14.23 | negative | 13 |  |  |
| 2397 | male | 57 | negative | 146.22 | 17.89 | 10.69 | 8.17 | negative | 14 |  |  |
| 2398 | male | 57 | negative | 139.91 | 10.38 | 1.88 | 13.48 | negative | 12 |  |  |
| 2399 | male | 57 | negative | 90.22 | 6.30 | 2.20 | 14.32 | negative | 12 |  |  |
| 2400 | male | 57 | negative | 61.53 | 7.61 | 1.68 | 8.09 | negative | 12 |  |  |
| 2401 | male | 57 | positive | 126.63 | 20.02 | 16.54 | 6.33 | negative | 15 |  |  |
| 2402 | male | 56 | negative | 147.27 | 11.29 | 8.24 | 13.04 | negative | 14 |  |  |
| 2403 | male | 56 | negative | 107.95 | 7.31 | 8.41 | 14.77 | negative | 14 |  |  |
| 2404 | male | 56 | negative | 120.87 | 12.30 | 8.53 | 9.83 | negative | 14 |  |  |
| 2405 | male | 56 | negative | 129.93 | 7.90 | 9.65 | 16.45 | negative | 14 |  |  |
| 2406 | male | 57 | positive | 103.53 | 14.66 | 10.87 | 7.06 | negative | 15 |  |  |
| 2407 | male | 57 | negative | 157.77 | 10.44 | 1.77 | 15.11 | negative | 12 |  |  |
| 2408 | male | 57 | negative | 134.49 | 9.17 | 1.80 | 14.67 | negative | 12 |  |  |
| 2409 | male | 57 | negative | 63.00 | 4.74 | 2.13 | 13.29 | negative | 12 |  |  |
| 2410 | male | 57 | negative | 92.12 | 4.23 | 2.21 | 21.78 | negative | 12 |  |  |
| 2411 | male | 56 | positive | 166.05 | 25.34 | 8.47 | 6.55 | negative | 15 |  |  |
| 2412 | male | 57 | positive | 49.87 | 6.64 | 1.87 | 7.51 | negative | 13 |  |  |
| 2413 | male | 57 | negative | 84.96 | 6.74 | 1.54 | 12.61 | negative | 12 |  |  |
| 2414 | male | 56 | negative | 120.43 | 12.46 | 8.20 | 9.67 | negative | 14 |  |  |
| 2415 | male | 57 | positive | 121.30 | 18.20 | 1.90 | 6.66 | negative | 13 |  |  |
| 2416 | male | 57 | positive | 169.43 | 21.96 | 10.04 | 7.72 | negative | 15 |  |  |
| 2417 | male | 57 | negative | 78.87 | 3.89 | 2.12 | 20.28 | negative | 12 |  |  |
| 2418 | male | 57 | negative | 300.00 | 11.61 | 19.28 | 25.84 | negative | 14 |  |  |
| 2419 | male | 57 | positive | 148.00 | 15.00 | 13.80 | 9.87 | negative | 15 |  |  |
| 2420 | male | 57 | negative | 300.00 | 36.05 | 11.97 | 8.32 | negative | 14 |  |  |
| 2421 | male | 57 | negative | 68.20 | 3.60 | 1.69 | 18.94 | negative | 12 |  |  |
| 2422 | male | 57 | negative | 60.10 | 5.20 | 2.20 | 11.56 | negative | 12 |  |  |
| 2423 | male | 57 | negative | 180.31 | 15.52 | 2.12 | 11.62 | negative | 12 |  |  |
| 2424 | male | 56 | negative | 143.18 | 12.56 | 8.53 | 11.40 | negative | 14 |  |  |
| 2425 | male | 57 | negative | 105.68 | 7.63 | 1.94 | 13.85 | negative | 12 |  |  |
| 2426 | male | 57 | positive | 96.68 | 16.75 | 11.39 | 5.77 | negative | 15 |  |  |
| 2427 | male | 56 | positive | 118.91 | 15.49 | 8.57 | 7.68 | negative | 15 |  |  |
| 2428 | male | 56 | positive | 172.66 | 14.02 | 8.51 | 12.32 | negative | 15 |  |  |
| 2429 | male | 57 | positive | 146.86 | 15.98 | 11.70 | 9.19 | negative | 15 |  |  |
| 2430 | male | 57 | positive | 163.22 | 19.56 | 15.31 | 8.34 | negative | 15 |  |  |
| 2431 | male | 57 | negative | 281.77 | 19.17 | 10.23 | 14.70 | negative | 14 |  |  |
| 2432 | female | 57 | positive | 104.20 | 27.00 | 11.50 | 3.86 | negative | 14 |  |  |
| 2433 | male | 57 | negative | 109.98 | 4.25 | 1.88 | 25.88 | negative | 12 |  |  |
| 2434 | male | 57 | positive | 99.30 | 19.70 | 17.10 | 5.04 | negative | 15 |  |  |
| 2435 | male | 57 | negative | 115.50 | 11.00 | 1.80 | 10.50 | negative | 12 |  |  |
| 2436 | male | 57 | negative | 119.40 | 9.80 | 1.90 | 12.18 | negative | 12 |  |  |
| 2437 | male | 57 | positive | 187.02 | 18.11 | 16.61 | 10.33 | negative | 15 |  |  |
| 2438 | male | 57 | negative | 126.03 | 8.04 | 2.02 | 15.68 | negative | 12 |  |  |
| 2439 | male | 57 | negative | 167.76 | 20.72 | 2.00 | 8.10 | negative | 12 |  |  |
| 2440 | male | 56 | positive | 111.41 | 16.31 | 8.45 | 6.83 | negative | 15 |  |  |
| 2441 | male | 57 | negative | 62.56 | 8.07 | 11.22 | 7.75 | negative | 14 |  |  |
| 2442 | male | 56 | positive | 122.22 | 14.72 | 8.57 | 8.30 | negative | 15 |  |  |
| 2443 | male | 57 | positive | 126.10 | 7.52 | 10.68 | 16.77 | negative | 15 |  |  |
| 2444 | male | 57 | negative | 261.34 | 16.36 | 12.09 | 15.97 | negative | 14 |  |  |
| 2445 | male | 57 | negative | 142.10 | 10.56 | 2.21 | 13.46 | negative | 12 |  |  |
| 2446 | male | 57 | positive | 124.04 | 23.10 | 14.86 | 5.37 | negative | 15 |  |  |
| 2447 | male | 57 | negative | 94.38 | 9.74 | 1.60 | 9.69 | negative | 12 |  |  |
| 2448 | male | 57 | negative | 79.74 | 4.77 | 1.69 | 16.72 | negative | 12 |  |  |
| 2449 | male | 57 | negative | 73.42 | 4.10 | 1.63 | 17.91 | negative | 12 |  |  |
| 2450 | male | 57 | negative | 15.32 | 2.71 | 11.47 | 5.65 | negative | 14 |  |  |
| 2451 | male | 57 | positive | 141.97 | 20.70 | 10.50 | 6.86 | negative | 15 |  |  |
| 2452 | male | 57 | negative | 93.70 | 6.80 | 2.20 | 13.78 | negative | 12 |  |  |
| 2453 | male | 57 | negative | 177.44 | 13.76 | 2.00 | 12.90 | negative | 12 |  |  |
| 2454 | male | 57 | negative | 100.70 | 7.32 | 2.05 | 13.76 | negative | 12 |  |  |
| 2455 | male | 57 | positive | 81.91 | 11.49 | 16.99 | 7.13 | negative | 15 |  |  |
| 2456 | male | 57 | negative | 146.45 | 9.79 | 1.53 | 14.96 | negative | 12 |  |  |
| 2457 | male | 57 | negative | 64.52 | 5.87 | 2.10 | 10.99 | negative | 12 |  |  |
| 2458 | male | 57 | negative | 119.78 | 11.68 | 1.66 | 10.26 | negative | 12 |  |  |
| 2459 | male | 57 | positive | 287.36 | 26.64 | 18.42 | 10.79 | negative | 15 |  |  |
| 2460 | male | 57 | negative | 74.42 | 4.71 | 1.98 | 15.80 | negative | 12 |  |  |
| 2461 | male | 57 | negative | 77.60 | 10.60 | 2.10 | 7.32 | negative | 12 |  |  |
| 2462 | male | 57 | negative | 130.34 | 5.94 | 2.10 | 21.94 | negative | 12 |  |  |
| 2463 | male | 57 | negative | 84.34 | 9.42 | 2.20 | 8.95 | negative | 12 |  |  |
| 2464 | male | 57 | negative | 58.68 | 4.32 | 1.69 | 13.58 | negative | 12 |  |  |
| 2465 | male | 57 | negative | 107.33 | 4.09 | 2.02 | 26.24 | negative | 12 |  |  |
| 2466 | male | 57 | negative | 106.46 | 7.06 | 2.18 | 15.08 | negative | 12 |  |  |
| 2467 | male | 56 | negative | 104.10 | 10.27 | 8.74 | 10.14 | negative | 14 |  |  |
| 2468 | female | 57 | positive | 44.87 | 18.46 | 13.48 | 2.43 | negative | 14 |  |  |
| 2469 | male | 57 | negative | 152.96 | 10.66 | 12.42 | 14.35 | negative | 14 |  |  |
| 2470 | male | 56 | negative | 179.56 | 17.79 | 8.25 | 10.09 | negative | 14 |  |  |
| 2471 | male | 57 | negative | 51.90 | 4.49 | 1.79 | 11.56 | negative | 12 |  |  |
| 2472 | male | 57 | negative | 146.79 | 9.98 | 14.76 | 14.71 | negative | 14 |  |  |
| 2473 | male | 57 | positive | 110.41 | 27.58 | 15.85 | 4.00 | negative | 15 |  |  |
| 2474 | male | 57 | negative | 94.61 | 4.72 | 1.56 | 20.04 | negative | 12 |  |  |
| 2475 | male | 57 | negative | 174.49 | 8.44 | 2.15 | 20.67 | negative | 12 |  |  |
| 2476 | male | 57 | positive | 117.70 | 20.78 | 11.57 | 5.66 | negative | 15 |  |  |
| 2477 | male | 57 | negative | 299.44 | 12.73 | 14.62 | 23.52 | negative | 14 |  |  |
| 2478 | male | 56 | positive | 128.43 | 10.93 | 9.54 | 11.75 | negative | 15 |  |  |
| 2479 | male | 57 | negative | 86.80 | 13.10 | 10.00 | 6.63 | negative | 14 |  |  |
| 2480 | male | 57 | negative | 72.05 | 8.49 | 10.82 | 8.49 | negative | 14 |  |  |
| 2481 | male | 56 | positive | 256.71 | 21.95 | 9.41 | 11.70 | negative | 15 |  |  |
| 2482 | male | 57 | negative | 95.56 | 5.62 | 1.53 | 17.00 | negative | 12 |  |  |
| 2483 | male | 57 | negative | 47.18 | 2.63 | 1.57 | 17.94 | negative | 12 |  |  |
| 2484 | male | 56 | positive | 251.87 | 16.42 | 9.55 | 15.34 | negative | 15 |  |  |
| 2485 | male | 57 | positive | 97.59 | 11.15 | 15.23 | 8.75 | negative | 15 |  |  |
| 2486 | male | 57 | negative | 132.70 | 6.69 | 1.96 | 19.84 | negative | 12 |  |  |
| 2487 | male | 57 | negative | 106.40 | 25.60 | 10.10 | 4.16 | negative | 14 |  |  |
| 2488 | male | 57 | negative | 90.13 | 10.41 | 1.64 | 8.66 | negative | 12 |  |  |
| 2489 | male | 57 | positive | 130.58 | 21.60 | 13.46 | 6.05 | negative | 15 |  |  |
| 2490 | male | 56 | positive | 134.52 | 14.23 | 8.19 | 9.45 | negative | 15 |  |  |
| 2491 | male | 57 | negative | 77.78 | 5.18 | 1.58 | 15.02 | negative | 12 |  |  |
| 2492 | male | 57 | negative | 104.20 | 8.92 | 1.80 | 11.68 | negative | 12 |  |  |
| 2493 | male | 56 | negative | 99.90 | 7.15 | 4.15 | 13.97 | negative | 12 |  |  |
| 2494 | male | 56 | positive | 144.94 | 13.17 | 4.34 | 11.01 | negative | 13 |  |  |
| 2495 | female | 56 | negative | 18.30 | 12.68 | 60.00 | 1.44 | negative | 13 |  |  |
| 2496 | male | 56 | negative | 116.19 | 14.98 | 4.87 | 7.76 | negative | 12 |  |  |
| 2497 | male | 56 | negative | 119.74 | 11.27 | 6.80 | 10.62 | negative | 14 |  |  |
| 2498 | male | 56 | negative | 129.59 | 9.89 | 4.63 | 13.10 | negative | 12 |  |  |
| 2499 | male | 56 | negative | 91.80 | 16.30 | 6.10 | 5.63 | negative | 14 |  |  |
| 2500 | male | 56 | negative | 150.06 | 17.99 | 4.37 | 8.34 | negative | 12 |  |  |
| 2501 | male | 56 | negative | 117.06 | 8.90 | 5.55 | 13.15 | negative | 12 |  |  |
| 2502 | male | 56 | positive | 120.40 | 14.30 | 4.30 | 8.42 | negative | 13 |  |  |
| 2503 | male | 56 | negative | 125.34 | 14.05 | 6.07 | 8.92 | negative | 14 |  |  |
| 2504 | male | 56 | negative | 115.30 | 17.30 | 7.10 | 6.66 | negative | 14 |  |  |
| 2505 | male | 56 | negative | 98.30 | 7.50 | 3.90 | 13.11 | negative | 12 |  |  |
| 2506 | male | 56 | positive | 131.84 | 19.61 | 46.21 | 6.72 | negative | 15 |  |  |
| 2507 | male | 56 | negative | 199.28 | 8.98 | 6.31 | 22.19 | negative | 14 |  |  |
| 2508 | male | 56 | negative | 110.24 | 12.90 | 3.87 | 8.55 | negative | 12 |  |  |
| 2509 | male | 56 | negative | 134.26 | 9.46 | 5.50 | 14.19 | negative | 12 |  |  |
| 2510 | female | 56 | negative | 8.27 | 4.35 | 60.00 | 1.90 | negative | 13 |  |  |
| 2511 | male | 56 | positive | 165.38 | 15.80 | 4.33 | 10.47 | negative | 13 |  |  |
| 2512 | male | 56 | negative | 108.61 | 8.89 | 4.81 | 12.22 | negative | 12 |  |  |
| 2513 | male | 56 | positive | 119.29 | 7.07 | 4.06 | 16.87 | negative | 13 |  |  |
| 2514 | male | 56 | negative | 127.27 | 5.97 | 7.81 | 21.32 | negative | 14 |  |  |
| 2515 | male | 56 | positive | 83.44 | 14.26 | 5.74 | 5.85 | negative | 15 |  |  |
| 2516 | male | 56 | negative | 62.76 | 6.61 | 4.72 | 9.49 | negative | 12 |  |  |
| 2517 | male | 56 | negative | 72.20 | 5.30 | 7.80 | 13.62 | negative | 14 |  |  |
| 2518 | male | 56 | negative | 79.64 | 11.48 | 7.02 | 6.94 | negative | 14 |  |  |
| 2519 | male | 56 | positive | 207.42 | 19.91 | 7.69 | 10.42 | negative | 15 |  |  |
| 2520 | male | 56 | positive | 171.25 | 24.34 | 6.68 | 7.04 | negative | 15 |  |  |
| 2521 | female | 56 | negative | 18.75 | 8.69 | 60.00 | 2.16 | negative | 13 |  |  |
| 2522 | male | 56 | positive | 137.28 | 26.81 | 6.18 | 5.12 | negative | 15 |  |  |
| 2523 | male | 56 | negative | 81.36 | 8.80 | 6.90 | 9.25 | negative | 14 |  |  |
| 2524 | male | 56 | negative | 143.54 | 13.10 | 7.40 | 10.96 | negative | 14 |  |  |
| 2525 | male | 56 | positive | 122.85 | 14.61 | 4.76 | 8.41 | negative | 13 |  |  |
| 2526 | male | 56 | positive | 104.76 | 12.68 | 5.55 | 8.26 | negative | 13 |  |  |
| 2527 | male | 56 | negative | 243.91 | 21.44 | 5.01 | 11.38 | negative | 12 |  |  |
| 2528 | male | 56 | negative | 89.00 | 8.40 | 4.40 | 10.60 | negative | 12 |  |  |
| 2529 | male | 56 | negative | 153.59 | 12.09 | 4.56 | 12.70 | negative | 12 |  |  |
| 2530 | female | 56 | positive | 37.99 | 23.40 | 5.97 | 1.62 | negative | 14 |  |  |
| 2531 | male | 56 | positive | 50.00 | 10.80 | 5.10 | 4.63 | negative | 13 |  |  |
| 2532 | male | 56 | positive | 300.00 | 26.68 | 5.46 | 11.24 | negative | 13 |  |  |
| 2533 | male | 56 | negative | 125.08 | 16.88 | 4.56 | 7.41 | negative | 12 |  |  |
| 2534 | male | 56 | negative | 99.03 | 5.60 | 4.53 | 17.68 | negative | 12 |  |  |
| 2535 | male | 56 | negative | 134.86 | 9.16 | 6.20 | 14.72 | negative | 14 |  |  |
| 2536 | male | 56 | negative | 130.80 | 8.90 | 6.71 | 14.70 | negative | 14 |  |  |
| 2537 | male | 56 | negative | 130.06 | 10.57 | 4.31 | 12.30 | negative | 12 |  |  |
| 2538 | male | 56 | negative | 171.32 | 11.84 | 7.10 | 14.47 | negative | 14 |  |  |
| 2539 | male | 56 | positive | 197.90 | 13.12 | 6.20 | 15.08 | negative | 15 |  |  |
| 2540 | male | 56 | positive | 120.75 | 16.08 | 3.89 | 7.51 | negative | 13 |  |  |
| 2541 | male | 56 | negative | 145.90 | 18.97 | 4.06 | 7.69 | negative | 12 |  |  |
| 2542 | male | 56 | negative | 134.01 | 12.27 | 3.91 | 10.92 | negative | 12 |  |  |
| 2543 | male | 56 | negative | 277.43 | 20.03 | 3.84 | 13.85 | negative | 12 |  |  |
| 2544 | male | 56 | negative | 103.92 | 5.44 | 5.57 | 19.10 | negative | 12 |  |  |
| 2545 | male | 56 | positive | 117.24 | 15.20 | 7.21 | 7.71 | negative | 15 |  |  |
| 2546 | male | 56 | positive | 129.28 | 12.84 | 5.70 | 10.07 | negative | 13 |  |  |
| 2547 | male | 56 | negative | 85.87 | 13.15 | 4.91 | 6.53 | negative | 12 |  |  |
| 2548 | male | 56 | negative | 150.52 | 10.97 | 7.53 | 13.72 | negative | 14 |  |  |
| 2549 | male | 56 | positive | 137.54 | 6.95 | 3.98 | 19.79 | negative | 13 |  |  |
| 2550 | male | 56 | positive | 140.97 | 12.87 | 4.35 | 10.95 | negative | 13 |  |  |
| 2551 | male | 56 | positive | 74.26 | 9.84 | 7.54 | 7.55 | negative | 15 |  |  |
| 2552 | male | 56 | negative | 262.74 | 9.02 | 4.17 | 29.13 | negative | 12 |  |  |
| 2553 | male | 56 | negative | 223.72 | 12.31 | 3.96 | 18.17 | negative | 12 |  |  |
| 2554 | male | 56 | negative | 144.91 | 5.67 | 4.15 | 25.56 | negative | 12 |  |  |
| 2555 | male | 56 | negative | 97.93 | 9.09 | 6.53 | 10.77 | negative | 14 |  |  |
| 2556 | male | 56 | negative | 75.78 | 5.87 | 6.86 | 12.91 | negative | 14 |  |  |
| 2557 | male | 56 | negative | 106.04 | 9.94 | 6.14 | 10.67 | negative | 14 |  |  |
| 2558 | male | 56 | negative | 51.28 | 5.13 | 4.51 | 10.00 | negative | 12 |  |  |
| 2559 | male | 56 | negative | 144.71 | 10.99 | 3.87 | 13.17 | negative | 12 |  |  |
| 2560 | male | 56 | negative | 216.92 | 17.60 | 5.35 | 12.32 | negative | 12 |  |  |
| 2561 | male | 56 | positive | 121.63 | 8.48 | 3.84 | 14.34 | negative | 13 |  |  |
| 2562 | male | 56 | positive | 147.22 | 11.67 | 6.00 | 12.62 | negative | 15 |  |  |
| 2563 | male | 56 | negative | 87.66 | 9.21 | 6.28 | 9.52 | negative | 14 |  |  |
| 2564 | male | 56 | negative | 173.27 | 10.14 | 4.00 | 17.09 | negative | 12 |  |  |
| 2565 | male | 56 | negative | 300.00 | 13.94 | 30.39 | 21.52 | negative | 14 |  |  |
| 2566 | male | 56 | negative | 56.00 | 5.90 | 5.60 | 9.49 | negative | 12 |  |  |
| 2567 | male | 56 | positive | 127.69 | 15.03 | 5.33 | 8.50 | negative | 13 |  |  |
| 2568 | male | 56 | positive | 87.90 | 9.94 | 7.65 | 8.84 | negative | 15 |  |  |
| 2569 | male | 56 | negative | 88.76 | 17.79 | 5.66 | 4.99 | negative | 12 |  |  |
| 2570 | male | 56 | negative | 130.31 | 14.24 | 7.01 | 9.15 | negative | 14 |  |  |
| 2571 | female | 56 | negative | 13.23 | 3.80 | 54.64 | 3.48 | negative | 13 |  |  |
| 2572 | male | 56 | negative | 62.44 | 8.53 | 6.79 | 7.32 | negative | 14 |  |  |
| 2573 | female | 56 | negative | 22.28 | 13.28 | 60.00 | 1.68 | negative | 13 |  |  |
| 2574 | male | 56 | negative | 168.22 | 11.82 | 6.81 | 14.23 | negative | 14 |  |  |
| 2575 | male | 56 | positive | 259.47 | 8.73 | 3.82 | 29.72 | negative | 13 |  |  |
| 2576 | male | 56 | positive | 74.44 | 10.28 | 6.75 | 7.24 | negative | 15 |  |  |
| 2577 | male | 56 | negative | 147.47 | 15.42 | 4.19 | 9.56 | negative | 12 |  |  |
| 2578 | male | 56 | negative | 85.15 | 5.40 | 4.71 | 15.77 | negative | 12 |  |  |
| 2579 | male | 56 | negative | 62.28 | 8.26 | 4.36 | 7.54 | negative | 12 |  |  |
| 2580 | male | 56 | positive | 100.27 | 14.85 | 7.27 | 6.75 | negative | 15 |  |  |
| 2581 | male | 56 | positive | 93.12 | 15.63 | 5.68 | 5.96 | negative | 13 |  |  |
| 2582 | male | 56 | negative | 185.40 | 14.58 | 5.19 | 12.72 | negative | 12 |  |  |
| 2583 | male | 56 | negative | 122.95 | 13.89 | 7.29 | 8.85 | negative | 14 |  |  |
| 2584 | male | 56 | negative | 181.85 | 6.30 | 58.76 | 28.87 | negative | 14 |  |  |
| 2585 | male | 56 | negative | 243.50 | 19.01 | 4.69 | 12.81 | negative | 12 |  |  |
| 2586 | male | 56 | negative | 119.69 | 6.57 | 6.07 | 18.22 | negative | 14 |  |  |
| 2587 | male | 56 | negative | 186.42 | 14.17 | 5.68 | 13.16 | negative | 12 |  |  |
| 2588 | male | 56 | negative | 137.19 | 11.62 | 6.08 | 11.81 | negative | 14 |  |  |
| 2589 | male | 56 | positive | 105.69 | 10.02 | 6.30 | 10.55 | negative | 15 |  |  |
| 2590 | male | 56 | positive | 70.91 | 11.54 | 5.09 | 6.14 | negative | 13 |  |  |
| 2591 | male | 56 | positive | 73.80 | 18.50 | 37.90 | 3.99 | negative | 15 |  |  |
| 2592 | female | 56 | positive | 62.24 | 16.31 | 3.85 | 3.82 | negative | 12 |  |  |
| 2593 | male | 56 | positive | 43.80 | 8.35 | 55.63 | 5.25 | negative | 15 |  |  |
| 2594 | male | 56 | negative | 57.16 | 8.56 | 32.47 | 6.68 | negative | 14 |  |  |
| 2595 | male | 56 | negative | 71.14 | 5.64 | 4.17 | 12.61 | negative | 12 |  |  |
| 2596 | male | 56 | positive | 278.97 | 21.24 | 4.10 | 13.13 | negative | 13 |  |  |
| 2597 | male | 56 | negative | 153.07 | 4.02 | 4.15 | 38.08 | negative | 12 |  |  |
| 2598 | male | 56 | positive | 280.86 | 18.66 | 7.30 | 15.05 | negative | 15 |  |  |
| 2599 | male | 56 | positive | 258.45 | 23.02 | 5.17 | 11.23 | negative | 13 |  |  |
| 2600 | male | 56 | positive | 167.78 | 15.07 | 6.32 | 11.13 | negative | 15 |  |  |
| 2601 | male | 56 | positive | 162.47 | 15.33 | 7.76 | 10.60 | negative | 15 |  |  |
| 2602 | male | 56 | negative | 105.84 | 7.03 | 4.25 | 15.06 | negative | 12 |  |  |
| 2603 | male | 56 | positive | 171.99 | 15.11 | 5.82 | 11.38 | negative | 15 |  |  |
| 2604 | male | 56 | negative | 90.31 | 6.86 | 5.90 | 13.16 | negative | 14 |  |  |
| 2605 | male | 56 | positive | 77.83 | 2.16 | 5.89 | 36.03 | negative | 15 |  |  |
| 2606 | male | 56 | positive | 145.63 | 21.08 | 6.35 | 6.91 | negative | 15 |  |  |
| 2607 | male | 56 | negative | 138.85 | 9.63 | 4.04 | 14.42 | negative | 12 |  |  |
| 2608 | male | 56 | negative | 86.31 | 14.52 | 7.46 | 5.94 | negative | 14 |  |  |
| 2609 | male | 56 | positive | 105.24 | 12.43 | 7.24 | 8.47 | negative | 15 |  |  |
| 2610 | male | 56 | negative | 106.10 | 8.80 | 4.50 | 12.06 | negative | 12 |  |  |
| 2611 | male | 56 | positive | 120.10 | 14.70 | 4.80 | 8.17 | negative | 13 |  |  |
| 2612 | male | 56 | negative | 68.03 | 7.73 | 5.77 | 8.80 | negative | 14 |  |  |
| 2613 | male | 56 | negative | 108.38 | 16.90 | 6.71 | 6.41 | negative | 14 |  |  |
| 2614 | male | 56 | negative | 108.76 | 8.22 | 4.56 | 13.23 | negative | 12 |  |  |
| 2615 | male | 56 | negative | 104.32 | 7.97 | 5.82 | 13.09 | negative | 14 |  |  |
| 2616 | male | 56 | negative | 84.60 | 5.20 | 6.70 | 16.27 | negative | 14 |  |  |
| 2617 | male | 56 | positive | 111.73 | 13.99 | 7.58 | 7.99 | negative | 15 |  |  |
| 2618 | male | 56 | negative | 134.04 | 11.50 | 7.01 | 11.66 | negative | 14 |  |  |
| 2619 | male | 56 | negative | 198.30 | 23.50 | 3.80 | 8.44 | negative | 12 |  |  |
| 2620 | male | 56 | negative | 110.14 | 8.67 | 6.86 | 12.70 | negative | 14 |  |  |
| 2621 | male | 56 | negative | 46.74 | 13.57 | 5.35 | 3.44 | negative | 15 |  |  |
| 2622 | male | 56 | negative | 52.40 | 13.60 | 49.30 | 3.85 | negative | 17 |  |  |
| 2623 | male | 56 | positive | 242.67 | 16.75 | 4.93 | 14.49 | negative | 13 |  |  |
| 2624 | male | 56 | negative | 35.70 | 3.30 | 35.00 | 10.82 | negative | 14 |  |  |
| 2625 | male | 56 | negative | 191.66 | 9.81 | 4.21 | 19.54 | negative | 12 |  |  |
| 2626 | male | 56 | negative | 84.16 | 10.97 | 4.21 | 7.67 | negative | 12 |  |  |
| 2627 | male | 56 | positive | 56.78 | 12.51 | 4.83 | 4.54 | negative | 13 |  |  |
| 2628 | male | 56 | positive | 211.54 | 14.53 | 6.83 | 14.56 | negative | 15 |  |  |
| 2629 | male | 56 | negative | 85.00 | 10.17 | 3.96 | 8.36 | negative | 12 |  |  |
| 2630 | male | 56 | positive | 140.90 | 18.22 | 6.48 | 7.73 | negative | 15 |  |  |
| 2631 | male | 56 | negative | 170.48 | 14.74 | 6.35 | 11.57 | negative | 14 |  |  |
| 2632 | male | 56 | positive | 154.17 | 14.80 | 5.75 | 10.42 | negative | 15 |  |  |
| 2633 | male | 56 | negative | 178.85 | 12.55 | 6.75 | 14.25 | negative | 14 |  |  |
| 2634 | male | 56 | negative | 70.86 | 5.28 | 6.37 | 13.42 | negative | 14 |  |  |
| 2635 | male | 56 | negative | 123.21 | 11.85 | 4.31 | 10.40 | negative | 12 |  |  |
| 2636 | male | 56 | negative | 135.97 | 7.15 | 6.10 | 19.02 | negative | 14 |  |  |
| 2637 | male | 56 | negative | 92.55 | 8.63 | 6.20 | 10.72 | negative | 14 |  |  |
| 2638 | male | 56 | positive | 110.25 | 20.44 | 6.02 | 5.39 | negative | 15 |  |  |
| 2639 | male | 56 | negative | 300.00 | 20.15 | 54.68 | 14.89 | negative | 14 |  |  |
| 2640 | male | 56 | negative | 123.17 | 7.34 | 5.28 | 16.78 | negative | 12 |  |  |
| 2641 | male | 56 | negative | 94.80 | 7.99 | 4.76 | 11.86 | negative | 12 |  |  |
| 2642 | male | 56 | positive | 177.50 | 25.35 | 7.59 | 7.00 | negative | 15 |  |  |
| 2643 | male | 56 | positive | 141.06 | 13.10 | 6.40 | 10.77 | negative | 15 |  |  |
| 2644 | male | 56 | positive | 102.64 | 13.49 | 6.70 | 7.61 | negative | 15 |  |  |
| 2645 | female | 56 | positive | 67.09 | 22.81 | 8.13 | 2.94 | negative | 14 |  |  |
| 2646 | male | 56 | positive | 56.16 | 7.63 | 3.89 | 7.36 | negative | 13 |  |  |
| 2647 | male | 56 | positive | 93.15 | 10.15 | 7.02 | 9.18 | negative | 15 |  |  |
| 2648 | male | 56 | negative | 94.60 | 11.30 | 3.90 | 8.37 | negative | 12 |  |  |
| 2649 | male | 56 | negative | 115.51 | 10.01 | 4.71 | 11.54 | negative | 12 |  |  |
| 2650 | male | 56 | negative | 87.92 | 8.25 | 3.80 | 10.66 | negative | 12 |  |  |
| 2651 | male | 56 | negative | 94.69 | 8.28 | 2.23 | 11.44 | negative | 12 |  |  |
| 2652 | male | 56 | negative | 110.65 | 7.47 | 3.68 | 14.81 | negative | 12 |  |  |
| 2653 | male | 56 | positive | 111.53 | 14.00 | 3.05 | 7.97 | negative | 13 |  |  |
| 2654 | male | 56 | negative | 161.26 | 10.77 | 2.34 | 14.97 | negative | 12 |  |  |
| 2655 | male | 56 | negative | 97.31 | 7.48 | 2.87 | 13.01 | negative | 12 |  |  |
| 2656 | male | 56 | negative | 98.08 | 6.95 | 3.61 | 14.11 | negative | 12 |  |  |
| 2657 | male | 56 | negative | 101.86 | 14.00 | 2.57 | 7.28 | negative | 12 |  |  |
| 2658 | male | 56 | negative | 175.60 | 12.35 | 3.60 | 14.22 | negative | 12 |  |  |
| 2659 | male | 56 | negative | 122.45 | 7.31 | 3.07 | 16.75 | negative | 12 |  |  |
| 2660 | male | 56 | negative | 117.21 | 6.59 | 3.63 | 17.79 | negative | 12 |  |  |
| 2661 | male | 56 | negative | 149.69 | 7.29 | 3.65 | 20.53 | negative | 12 |  |  |
| 2662 | male | 56 | negative | 64.86 | 4.31 | 3.10 | 15.05 | negative | 12 |  |  |
| 2663 | male | 56 | positive | 227.66 | 20.66 | 2.73 | 11.02 | negative | 13 |  |  |
| 2664 | male | 56 | negative | 116.62 | 9.84 | 3.21 | 11.85 | negative | 12 |  |  |
| 2665 | male | 56 | negative | 92.98 | 5.35 | 2.54 | 17.38 | negative | 12 |  |  |
| 2666 | male | 56 | negative | 67.86 | 4.54 | 2.84 | 14.95 | negative | 12 |  |  |
| 2667 | male | 56 | positive | 124.90 | 16.70 | 3.00 | 7.48 | negative | 13 |  |  |
| 2668 | male | 56 | negative | 146.46 | 9.53 | 3.39 | 15.37 | negative | 12 |  |  |
| 2669 | male | 56 | negative | 72.52 | 3.95 | 2.78 | 18.36 | negative | 12 |  |  |
| 2670 | male | 56 | negative | 96.89 | 7.02 | 2.78 | 13.80 | negative | 12 |  |  |
| 2671 | male | 56 | positive | 147.68 | 14.76 | 3.13 | 10.01 | negative | 13 |  |  |
| 2672 | male | 56 | negative | 97.25 | 4.70 | 2.58 | 20.69 | negative | 12 |  |  |
| 2673 | male | 56 | positive | 76.10 | 12.50 | 3.20 | 6.09 | negative | 13 |  |  |
| 2674 | male | 56 | negative | 165.05 | 11.33 | 3.27 | 14.57 | negative | 12 |  |  |
| 2675 | male | 56 | negative | 87.48 | 8.29 | 2.44 | 10.55 | negative | 12 |  |  |
| 2676 | male | 56 | negative | 86.04 | 7.40 | 2.40 | 11.63 | negative | 12 |  |  |
| 2677 | male | 56 | negative | 125.17 | 10.45 | 2.81 | 11.98 | negative | 12 |  |  |
| 2678 | male | 56 | negative | 77.68 | 6.97 | 3.14 | 11.14 | negative | 12 |  |  |
| 2679 | male | 56 | negative | 134.72 | 7.53 | 3.50 | 17.89 | negative | 12 |  |  |
| 2680 | male | 56 | positive | 168.65 | 8.18 | 2.49 | 20.62 | negative | 13 |  |  |
| 2681 | male | 56 | positive | 155.66 | 9.29 | 2.93 | 16.76 | negative | 13 |  |  |
| 2682 | male | 56 | negative | 112.46 | 8.22 | 3.63 | 13.68 | negative | 12 |  |  |
| 2683 | male | 56 | negative | 75.38 | 6.10 | 2.61 | 12.36 | negative | 12 |  |  |
| 2684 | male | 56 | negative | 279.83 | 12.39 | 3.37 | 22.59 | negative | 12 |  |  |
| 2685 | male | 56 | negative | 146.32 | 6.28 | 2.34 | 23.30 | negative | 12 |  |  |
| 2686 | male | 56 | negative | 81.80 | 7.57 | 2.31 | 10.81 | negative | 12 |  |  |
| 2687 | male | 56 | negative | 171.40 | 7.30 | 3.10 | 23.48 | negative | 12 |  |  |
| 2688 | male | 56 | positive | 138.12 | 8.02 | 2.50 | 17.22 | negative | 13 |  |  |
| 2689 | male | 56 | negative | 300.00 | 22.82 | 3.55 | 13.15 | negative | 12 |  |  |
| 2690 | male | 56 | negative | 69.43 | 4.93 | 3.16 | 14.08 | negative | 12 |  |  |
| 2691 | male | 56 | positive | 297.55 | 40.83 | 21.91 | 7.29 | negative | 15 |  |  |
| 2692 | male | 56 | negative | 97.18 | 10.62 | 3.10 | 9.15 | negative | 12 |  |  |
| 2693 | male | 56 | negative | 192.04 | 11.45 | 2.78 | 16.77 | negative | 12 |  |  |
| 2694 | male | 56 | negative | 105.34 | 8.20 | 2.86 | 12.85 | negative | 12 |  |  |
| 2695 | male | 56 | negative | 162.38 | 7.89 | 2.60 | 20.58 | negative | 12 |  |  |
| 2696 | male | 56 | negative | 73.30 | 4.70 | 3.60 | 15.60 | negative | 12 |  |  |
| 2697 | male | 56 | positive | 150.81 | 17.70 | 3.01 | 8.52 | negative | 13 |  |  |
| 2698 | male | 56 | negative | 127.50 | 9.11 | 2.19 | 14.00 | negative | 12 |  |  |
| 2699 | male | 56 | positive | 300.00 | 22.23 | 3.20 | 13.50 | negative | 13 |  |  |
| 2700 | male | 56 | negative | 98.01 | 15.47 | 3.09 | 6.34 | negative | 12 |  |  |
| 2701 | male | 56 | negative | 78.51 | 6.51 | 2.17 | 12.06 | negative | 12 |  |  |
| 2702 | male | 56 | negative | 159.59 | 11.79 | 2.31 | 13.54 | negative | 12 |  |  |
| 2703 | male | 56 | negative | 99.60 | 8.10 | 3.20 | 12.30 | negative | 12 |  |  |
| 2704 | male | 56 | negative | 120.65 | 12.18 | 2.26 | 9.91 | negative | 12 |  |  |
| 2705 | male | 56 | negative | 201.83 | 9.01 | 3.19 | 22.40 | negative | 12 |  |  |
| 2706 | male | 56 | positive | 281.08 | 49.71 | 27.02 | 5.65 | negative | 15 |  |  |
| 2707 | male | 56 | positive | 122.30 | 26.69 | 20.32 | 4.58 | negative | 15 |  |  |
| 2708 | male | 56 | negative | 81.63 | 8.05 | 2.51 | 10.14 | negative | 12 |  |  |
| 2709 | male | 56 | negative | 160.63 | 11.71 | 2.81 | 13.72 | negative | 12 |  |  |
| 2710 | male | 56 | negative | 69.86 | 6.21 | 2.96 | 11.25 | negative | 12 |  |  |
| 2711 | male | 56 | positive | 225.54 | 20.25 | 22.95 | 11.14 | negative | 15 |  |  |
| 2712 | male | 56 | negative | 99.00 | 7.53 | 2.74 | 13.15 | negative | 12 |  |  |
| 2713 | male | 56 | negative | 136.15 | 13.25 | 2.62 | 10.28 | negative | 12 |  |  |
| 2714 | male | 56 | negative | 140.13 | 8.77 | 3.68 | 15.98 | negative | 12 |  |  |
| 2715 | male | 56 | negative | 126.65 | 11.48 | 2.56 | 11.03 | negative | 12 |  |  |
| 2716 | male | 56 | negative | 120.42 | 7.57 | 3.50 | 15.91 | negative | 12 |  |  |
| 2717 | male | 56 | negative | 70.88 | 8.14 | 3.16 | 8.71 | negative | 12 |  |  |
| 2718 | male | 56 | negative | 100.71 | 7.44 | 2.94 | 13.54 | negative | 12 |  |  |
| 2719 | male | 56 | negative | 161.79 | 10.55 | 3.19 | 15.34 | negative | 12 |  |  |
| 2720 | male | 56 | positive | 214.54 | 22.13 | 21.78 | 9.69 | negative | 15 |  |  |
| 2721 | male | 56 | negative | 152.31 | 6.99 | 3.38 | 21.79 | negative | 12 |  |  |
| 2722 | male | 56 | negative | 224.64 | 19.61 | 2.73 | 11.46 | negative | 12 |  |  |
| 2723 | male | 56 | negative | 94.78 | 7.53 | 2.30 | 12.59 | negative | 12 |  |  |
| 2724 | male | 56 | negative | 123.60 | 12.92 | 3.10 | 9.57 | negative | 12 |  |  |
| 2725 | male | 56 | negative | 84.02 | 6.32 | 2.50 | 13.29 | negative | 12 |  |  |
| 2726 | male | 56 | negative | 106.58 | 18.84 | 3.19 | 5.66 | negative | 12 |  |  |
| 2727 | male | 56 | negative | 116.67 | 10.17 | 3.38 | 11.47 | negative | 12 |  |  |
| 2728 | male | 56 | negative | 72.75 | 5.26 | 2.67 | 13.83 | negative | 12 |  |  |
| 2729 | male | 56 | negative | 145.43 | 8.15 | 3.80 | 17.84 | negative | 12 |  |  |
| 2730 | male | 56 | negative | 108.44 | 8.89 | 3.68 | 12.20 | negative | 12 |  |  |
| 2731 | male | 56 | negative | 99.20 | 21.20 | 3.10 | 4.68 | negative | 12 |  |  |
| 2732 | male | 56 | negative | 102.35 | 7.60 | 2.86 | 13.47 | negative | 12 |  |  |
| 2733 | male | 56 | negative | 92.60 | 5.20 | 3.30 | 17.81 | negative | 12 |  |  |
| 2734 | male | 56 | negative | 100.89 | 5.15 | 2.72 | 19.59 | negative | 12 |  |  |
| 2735 | male | 56 | negative | 247.66 | 14.29 | 3.21 | 17.33 | negative | 12 |  |  |
| 2736 | male | 56 | negative | 72.00 | 7.66 | 3.41 | 9.40 | negative | 12 |  |  |
| 2737 | male | 56 | negative | 152.94 | 6.48 | 2.74 | 23.60 | negative | 12 |  |  |
| 2738 | male | 56 | negative | 86.43 | 8.73 | 2.72 | 9.90 | negative | 12 |  |  |
| 2739 | male | 56 | positive | 181.65 | 15.79 | 26.00 | 11.50 | negative | 15 |  |  |
| 2740 | male | 56 | negative | 132.77 | 10.28 | 3.40 | 12.92 | negative | 12 |  |  |
| 2741 | male | 56 | positive | 55.20 | 12.70 | 2.70 | 4.35 | negative | 13 |  |  |
| 2742 | male | 56 | negative | 124.06 | 7.14 | 3.71 | 17.38 | negative | 12 |  |  |
| 2743 | male | 56 | negative | 117.92 | 4.60 | 3.75 | 25.63 | negative | 12 |  |  |
| 2744 | male | 56 | positive | 136.58 | 11.26 | 2.63 | 12.13 | negative | 13 |  |  |
| 2745 | male | 56 | negative | 74.02 | 10.50 | 3.04 | 7.05 | negative | 12 |  |  |
| 2746 | male | 56 | negative | 108.48 | 8.34 | 2.80 | 13.01 | negative | 12 |  |  |
| 2747 | male | 56 | positive | 142.39 | 31.19 | 26.96 | 4.57 | negative | 15 |  |  |
| 2748 | male | 56 | negative | 52.04 | 4.49 | 2.16 | 11.59 | negative | 12 |  |  |
| 2749 | male | 56 | positive | 83.34 | 13.44 | 3.70 | 6.20 | negative | 13 |  |  |
| 2750 | male | 56 | negative | 194.91 | 11.12 | 2.27 | 17.53 | negative | 12 |  |  |
| 2751 | male | 56 | negative | 201.44 | 15.39 | 3.44 | 13.09 | negative | 12 |  |  |
| 2752 | male | 56 | negative | 300.00 | 22.12 | 2.56 | 13.56 | negative | 12 |  |  |
| 2753 | male | 56 | positive | 69.97 | 13.15 | 2.52 | 5.32 | negative | 13 |  |  |
| 2754 | male | 56 | negative | 163.30 | 14.90 | 2.70 | 10.96 | negative | 12 |  |  |
| 2755 | male | 56 | negative | 75.24 | 5.58 | 2.37 | 13.48 | negative | 12 |  |  |
| 2756 | male | 56 | negative | 290.70 | 19.10 | 25.80 | 15.22 | negative | 14 |  |  |
| 2757 | male | 56 | negative | 140.62 | 11.08 | 2.51 | 12.69 | negative | 12 |  |  |
| 2758 | male | 56 | negative | 77.30 | 6.80 | 2.80 | 11.37 | negative | 12 |  |  |
| 2759 | male | 56 | negative | 117.36 | 10.11 | 2.58 | 11.61 | negative | 12 |  |  |
| 2760 | male | 56 | negative | 113.69 | 6.94 | 3.15 | 16.38 | negative | 12 |  |  |
| 2761 | male | 56 | negative | 196.96 | 17.93 | 2.66 | 10.98 | negative | 12 |  |  |
| 2762 | male | 56 | positive | 230.98 | 22.72 | 2.18 | 10.17 | negative | 13 |  |  |
| 2763 | male | 56 | negative | 100.23 | 14.85 | 3.67 | 6.75 | negative | 12 |  |  |
| 2764 | male | 56 | positive | 144.26 | 17.01 | 22.42 | 8.48 | negative | 15 |  |  |
| 2765 | male | 56 | negative | 96.37 | 8.18 | 2.89 | 11.78 | negative | 12 |  |  |
| 2766 | male | 56 | positive | 95.48 | 8.50 | 2.58 | 11.23 | negative | 13 |  |  |
| 2767 | male | 56 | negative | 76.52 | 4.66 | 2.50 | 16.42 | negative | 12 |  |  |
| 2768 | male | 56 | negative | 285.68 | 15.86 | 2.26 | 18.01 | negative | 12 |  |  |
| 2769 | male | 56 | negative | 78.46 | 7.31 | 3.45 | 10.73 | negative | 12 |  |  |
| 2770 | male | 56 | positive | 300.00 | 19.16 | 3.08 | 15.66 | negative | 13 |  |  |
| 2771 | male | 56 | negative | 96.40 | 7.92 | 2.70 | 12.17 | negative | 12 |  |  |
| 2772 | male | 56 | negative | 80.49 | 5.79 | 2.97 | 13.90 | negative | 12 |  |  |
| 2773 | male | 56 | negative | 98.68 | 17.09 | 2.19 | 5.77 | negative | 12 |  |  |
| 2774 | male | 56 | positive | 133.72 | 11.52 | 2.61 | 11.61 | negative | 13 |  |  |
| 2775 | male | 56 | positive | 93.85 | 17.03 | 2.83 | 5.51 | negative | 13 |  |  |
| 2776 | male | 56 | positive | 188.59 | 15.53 | 3.11 | 12.14 | negative | 13 |  |  |
| 2777 | male | 56 | negative | 158.57 | 12.56 | 2.81 | 12.63 | negative | 12 |  |  |
| 2778 | male | 56 | negative | 91.40 | 13.60 | 2.40 | 6.72 | negative | 12 |  |  |
| 2779 | male | 56 | negative | 109.78 | 8.55 | 3.41 | 12.84 | negative | 12 |  |  |
| 2780 | male | 56 | negative | 162.77 | 13.20 | 3.42 | 12.33 | negative | 12 |  |  |
| 2781 | male | 56 | negative | 94.14 | 9.91 | 2.22 | 9.50 | negative | 12 |  |  |
| 2782 | male | 56 | negative | 69.40 | 8.50 | 22.30 | 8.16 | negative | 14 |  |  |
| 2783 | male | 56 | negative | 300.00 | 32.29 | 2.37 | 9.29 | negative | 12 |  |  |
| 2784 | male | 56 | positive | 67.10 | 9.34 | 2.76 | 7.18 | negative | 13 |  |  |
| 2785 | male | 56 | negative | 125.30 | 7.57 | 2.42 | 16.55 | negative | 12 |  |  |
| 2786 | male | 56 | negative | 194.40 | 21.40 | 10.28 | 9.08 | negative | 14 |  |  |
| 2787 | male | 56 | positive | 190.60 | 19.00 | 11.76 | 10.03 | negative | 15 |  |  |
| 2788 | male | 56 | negative | 60.74 | 6.66 | 1.58 | 9.12 | negative | 12 |  |  |
| 2789 | male | 56 | positive | 84.89 | 11.82 | 11.45 | 7.18 | negative | 15 |  |  |
| 2790 | male | 56 | negative | 78.54 | 11.13 | 14.65 | 7.06 | negative | 14 |  |  |
| 2791 | male | 56 | positive | 98.22 | 22.90 | 11.57 | 4.29 | negative | 15 |  |  |
| 2792 | male | 56 | negative | 86.91 | 4.12 | 1.97 | 21.09 | negative | 12 |  |  |
| 2793 | male | 55 | positive | 263.77 | 20.79 | 9.05 | 12.69 | negative | 15 |  |  |
| 2794 | male | 56 | negative | 141.62 | 5.74 | 1.94 | 24.67 | negative | 12 |  |  |
| 2795 | male | 55 | positive | 196.99 | 28.65 | 9.34 | 6.88 | negative | 15 |  |  |
| 2796 | male | 56 | negative | 121.45 | 22.33 | 17.05 | 5.44 | negative | 14 |  |  |
| 2797 | male | 56 | positive | 173.36 | 3.53 | 12.57 | 49.11 | negative | 15 |  |  |
| 2798 | male | 55 | positive | 205.49 | 12.70 | 9.99 | 16.18 | negative | 15 |  |  |
| 2799 | male | 56 | positive | 258.49 | 22.73 | 14.62 | 11.37 | negative | 15 |  |  |
| 2800 | male | 56 | negative | 148.82 | 9.06 | 2.10 | 16.43 | negative | 12 |  |  |
| 2801 | male | 55 | positive | 199.23 | 17.63 | 9.89 | 11.30 | negative | 15 |  |  |
| 2802 | male | 56 | positive | 143.20 | 20.50 | 18.39 | 6.99 | negative | 15 |  |  |
| 2803 | male | 56 | negative | 171.83 | 5.41 | 1.50 | 31.76 | negative | 12 |  |  |
| 2804 | male | 56 | negative | 125.17 | 9.50 | 1.93 | 13.18 | negative | 12 |  |  |
| 2805 | male | 55 | positive | 160.88 | 18.43 | 8.62 | 8.73 | negative | 15 |  |  |
| 2806 | male | 56 | positive | 142.36 | 11.79 | 12.81 | 12.07 | negative | 15 |  |  |
| 2807 | male | 56 | negative | 151.20 | 9.70 | 11.22 | 15.59 | negative | 14 |  |  |
| 2808 | male | 56 | positive | 109.91 | 14.15 | 16.96 | 7.77 | negative | 15 |  |  |
| 2809 | male | 55 | negative | 122.66 | 14.40 | 9.83 | 8.52 | negative | 14 |  |  |
| 2810 | male | 56 | negative | 120.80 | 13.60 | 1.78 | 8.88 | negative | 12 |  |  |
| 2811 | male | 56 | positive | 24.05 | 11.05 | 12.39 | 2.18 | negative | 18 |  |  |
| 2812 | male | 55 | positive | 166.67 | 24.61 | 8.62 | 6.77 | negative | 15 |  |  |
| 2813 | male | 56 | negative | 200.15 | 13.94 | 1.56 | 14.36 | negative | 12 |  |  |
| 2814 | male | 56 | positive | 51.38 | 12.18 | 1.96 | 4.22 | negative | 13 |  |  |
| 2815 | male | 56 | negative | 130.70 | 10.20 | 12.60 | 12.81 | negative | 14 |  |  |
| 2816 | male | 56 | negative | 48.28 | 9.83 | 2.12 | 4.91 | negative | 12 |  |  |
| 2817 | male | 56 | negative | 47.01 | 8.79 | 1.82 | 5.35 | negative | 12 |  |  |
| 2818 | female | 56 | negative | 62.80 | 18.20 | 14.80 | 3.45 | negative | 13 |  |  |
| 2819 | male | 56 | positive | 148.30 | 14.80 | 10.80 | 10.02 | negative | 15 |  |  |
| 2820 | male | 56 | positive | 150.20 | 11.60 | 12.00 | 12.95 | negative | 15 |  |  |
| 2821 | male | 56 | negative | 109.53 | 5.78 | 1.62 | 18.95 | negative | 12 |  |  |
| 2822 | male | 56 | positive | 140.73 | 14.47 | 11.56 | 9.73 | negative | 15 |  |  |
| 2823 | male | 56 | positive | 102.65 | 18.35 | 13.47 | 5.59 | negative | 15 |  |  |
| 2824 | male | 56 | negative | 71.62 | 15.40 | 13.08 | 4.65 | negative | 14 |  |  |
| 2825 | male | 55 | positive | 60.47 | 11.22 | 9.33 | 5.39 | negative | 15 |  |  |
| 2826 | male | 55 | positive | 107.60 | 20.10 | 8.60 | 5.35 | negative | 15 |  |  |
| 2827 | male | 56 | negative | 143.36 | 9.85 | 2.10 | 14.55 | negative | 12 |  |  |
| 2828 | male | 56 | negative | 122.93 | 15.30 | 2.10 | 8.03 | negative | 12 |  |  |
| 2829 | male | 56 | negative | 66.32 | 5.11 | 1.95 | 12.98 | negative | 12 |  |  |
| 2830 | male | 55 | positive | 168.66 | 16.32 | 8.36 | 10.33 | negative | 15 |  |  |
| 2831 | male | 56 | negative | 144.00 | 10.88 | 1.87 | 13.24 | negative | 12 |  |  |
| 2832 | male | 55 | negative | 120.02 | 20.95 | 8.46 | 5.73 | negative | 14 |  |  |
| 2833 | male | 56 | positive | 141.27 | 14.97 | 15.37 | 9.44 | negative | 15 |  |  |
| 2834 | male | 56 | negative | 117.05 | 10.67 | 2.02 | 10.97 | negative | 12 |  |  |
| 2835 | male | 56 | negative | 287.65 | 30.18 | 17.93 | 9.53 | negative | 14 |  |  |
| 2836 | male | 55 | positive | 299.39 | 19.01 | 9.72 | 15.75 | negative | 15 |  |  |
| 2837 | male | 56 | positive | 289.67 | 18.98 | 2.09 | 15.26 | negative | 13 |  |  |
| 2838 | male | 56 | positive | 159.60 | 9.75 | 18.73 | 16.37 | negative | 15 |  |  |
| 2839 | male | 55 | positive | 94.40 | 14.40 | 8.50 | 6.56 | negative | 15 |  |  |
| 2840 | male | 55 | positive | 89.09 | 18.56 | 9.77 | 4.80 | negative | 15 |  |  |
| 2841 | male | 56 | negative | 148.54 | 6.25 | 1.99 | 23.77 | negative | 12 |  |  |
| 2842 | male | 56 | positive | 173.67 | 17.72 | 19.67 | 9.80 | negative | 15 |  |  |
| 2843 | male | 56 | positive | 109.16 | 13.00 | 14.99 | 8.40 | negative | 15 |  |  |
| 2844 | male | 56 | negative | 122.15 | 5.74 | 1.80 | 21.28 | negative | 12 |  |  |
| 2845 | male | 55 | negative | 72.01 | 6.89 | 9.81 | 10.45 | negative | 14 |  |  |
| 2846 | male | 56 | negative | 60.42 | 23.77 | 12.63 | 2.54 | negative | 17 |  |  |
| 2847 | male | 56 | negative | 86.03 | 7.20 | 1.50 | 11.95 | negative | 12 |  |  |
| 2848 | male | 56 | negative | 114.71 | 19.20 | 10.72 | 5.97 | negative | 14 |  |  |
| 2849 | male | 55 | positive | 75.27 | 5.12 | 9.05 | 14.70 | negative | 15 |  |  |
| 2850 | male | 56 | negative | 42.67 | 19.75 | 14.22 | 2.16 | negative | 17 |  |  |
| 2851 | male | 56 | positive | 95.88 | 19.48 | 10.37 | 4.92 | negative | 15 |  |  |
| 2852 | male | 56 | negative | 87.14 | 7.97 | 1.99 | 10.93 | negative | 12 |  |  |
| 2853 | male | 56 | negative | 37.79 | 3.68 | 1.99 | 10.27 | negative | 12 |  |  |
| 2854 | male | 56 | negative | 97.38 | 9.00 | 1.63 | 10.82 | negative | 12 |  |  |
| 2855 | male | 56 | positive | 286.49 | 33.61 | 14.08 | 8.52 | negative | 15 |  |  |
| 2856 | male | 55 | negative | 59.50 | 8.29 | 9.66 | 7.18 | negative | 14 |  |  |
| 2857 | male | 56 | negative | 124.50 | 9.67 | 1.50 | 12.87 | negative | 12 |  |  |
| 2858 | male | 56 | negative | 145.17 | 13.75 | 10.46 | 10.56 | negative | 14 |  |  |
| 2859 | male | 56 | negative | 128.46 | 9.41 | 1.52 | 13.65 | negative | 12 |  |  |
| 2860 | male | 56 | negative | 78.49 | 5.48 | 12.23 | 14.32 | negative | 14 |  |  |
| 2861 | female | 55 | positive | 60.56 | 15.83 | 9.56 | 3.83 | negative | 14 |  |  |
| 2862 | male | 55 | positive | 96.65 | 11.26 | 8.74 | 8.58 | negative | 15 |  |  |
| 2863 | male | 56 | negative | 94.35 | 13.84 | 11.05 | 6.82 | negative | 14 |  |  |
| 2864 | male | 56 | positive | 159.71 | 23.67 | 14.99 | 6.75 | negative | 15 |  |  |
| 2865 | male | 56 | negative | 112.91 | 6.39 | 2.01 | 17.67 | negative | 12 |  |  |
| 2866 | female | 56 | positive | 87.14 | 23.01 | 17.38 | 3.79 | negative | 14 |  |  |
| 2867 | male | 56 | positive | 110.16 | 21.59 | 12.19 | 5.10 | negative | 15 |  |  |
| 2868 | male | 56 | negative | 117.05 | 8.11 | 12.19 | 14.43 | negative | 14 |  |  |
| 2869 | male | 56 | negative | 110.30 | 9.55 | 1.64 | 11.55 | negative | 12 |  |  |
| 2870 | male | 56 | negative | 160.96 | 15.13 | 1.65 | 10.64 | negative | 12 |  |  |
| 2871 | male | 56 | positive | 233.10 | 19.36 | 10.08 | 12.04 | negative | 15 |  |  |
| 2872 | male | 56 | negative | 63.08 | 3.72 | 1.96 | 16.96 | negative | 12 |  |  |
| 2873 | male | 56 | positive | 216.22 | 23.87 | 12.92 | 9.06 | negative | 15 |  |  |
| 2874 | male | 55 | positive | 173.22 | 15.89 | 8.12 | 10.90 | negative | 15 |  |  |
| 2875 | male | 56 | negative | 300.00 | 23.95 | 1.55 | 12.53 | negative | 12 |  |  |
| 2876 | male | 56 | negative | 124.18 | 12.93 | 1.82 | 9.60 | negative | 12 |  |  |
| 2877 | male | 56 | positive | 189.52 | 30.91 | 11.87 | 6.13 | negative | 15 |  |  |
| 2878 | male | 56 | negative | 58.98 | 8.12 | 15.96 | 7.26 | negative | 14 |  |  |
| 2879 | male | 56 | negative | 88.22 | 5.75 | 1.76 | 15.34 | negative | 12 |  |  |
| 2880 | male | 56 | negative | 105.50 | 4.29 | 1.53 | 24.59 | negative | 12 |  |  |
| 2881 | male | 56 | positive | 300.00 | 43.74 | 17.34 | 6.86 | negative | 15 |  |  |
| 2882 | male | 56 | negative | 91.32 | 7.62 | 1.59 | 11.98 | negative | 12 |  |  |
| 2883 | male | 56 | positive | 69.54 | 15.27 | 16.36 | 4.55 | negative | 15 |  |  |
| 2884 | male | 56 | positive | 250.29 | 31.28 | 13.35 | 8.00 | negative | 15 |  |  |
| 2885 | male | 56 | positive | 61.08 | 8.20 | 14.36 | 7.45 | negative | 15 |  |  |
| 2886 | male | 56 | negative | 58.15 | 4.18 | 10.59 | 13.91 | negative | 14 |  |  |
| 2887 | male | 56 | negative | 93.53 | 5.45 | 1.76 | 17.16 | negative | 12 |  |  |
| 2888 | male | 55 | negative | 130.84 | 12.72 | 9.51 | 10.29 | negative | 14 |  |  |
| 2889 | male | 55 | positive | 153.91 | 18.05 | 9.80 | 8.53 | negative | 15 |  |  |
| 2890 | male | 55 | negative | 300.00 | 37.03 | 8.92 | 8.10 | negative | 14 |  |  |
| 2891 | male | 56 | negative | 130.29 | 6.01 | 1.73 | 21.68 | negative | 12 |  |  |
| 2892 | male | 55 | negative | 126.05 | 10.77 | 9.32 | 11.70 | negative | 14 |  |  |
| 2893 | male | 56 | negative | 65.78 | 4.50 | 1.64 | 14.62 | negative | 12 |  |  |
| 2894 | male | 56 | negative | 153.69 | 15.02 | 1.96 | 10.23 | negative | 12 |  |  |
| 2895 | male | 56 | negative | 95.65 | 8.17 | 1.73 | 11.71 | negative | 12 |  |  |
| 2896 | male | 56 | positive | 92.03 | 7.98 | 1.86 | 11.53 | negative | 13 |  |  |
| 2897 | male | 56 | negative | 60.11 | 4.32 | 1.67 | 13.91 | negative | 12 |  |  |
| 2898 | male | 56 | negative | 169.10 | 21.39 | 16.13 | 7.91 | negative | 14 |  |  |
| 2899 | male | 56 | negative | 79.72 | 2.96 | 1.67 | 26.93 | negative | 12 |  |  |
| 2900 | male | 55 | positive | 136.92 | 22.80 | 8.73 | 6.01 | negative | 15 |  |  |
| 2901 | male | 56 | positive | 130.10 | 7.61 | 2.09 | 17.10 | negative | 13 |  |  |
| 2902 | male | 56 | negative | 67.66 | 9.34 | 1.56 | 7.24 | negative | 12 |  |  |
| 2903 | male | 56 | negative | 367.89 | 24.68 | 14.04 | 14.91 | negative | 14 |  |  |
| 2904 | male | 56 | positive | 300.00 | 20.61 | 11.05 | 14.56 | negative | 15 |  |  |
| 2905 | male | 56 | negative | 60.81 | 3.90 | 1.52 | 15.59 | negative | 12 |  |  |
| 2906 | male | 55 | positive | 128.30 | 16.26 | 9.24 | 7.89 | negative | 15 |  |  |
| 2907 | male | 56 | negative | 125.97 | 12.29 | 1.77 | 10.25 | negative | 12 |  |  |
| 2908 | male | 56 | positive | 120.94 | 5.34 | 2.10 | 22.65 | negative | 13 |  |  |
| 2909 | male | 56 | positive | 57.08 | 12.26 | 13.74 | 4.66 | negative | 15 |  |  |
| 2910 | male | 56 | positive | 102.79 | 7.04 | 11.58 | 14.60 | negative | 15 |  |  |
| 2911 | male | 56 | negative | 208.77 | 11.89 | 12.18 | 17.56 | negative | 14 |  |  |
| 2912 | male | 56 | negative | 197.85 | 14.74 | 1.85 | 13.42 | negative | 12 |  |  |
| 2913 | male | 56 | negative | 106.66 | 6.97 | 1.70 | 15.30 | negative | 12 |  |  |
| 2914 | male | 55 | negative | 105.90 | 12.96 | 6.01 | 8.17 | negative | 14 |  |  |
| 2915 | male | 55 | negative | 245.35 | 9.15 | 32.95 | 26.81 | negative | 14 |  |  |
| 2916 | male | 55 | negative | 90.61 | 8.46 | 3.70 | 10.71 | negative | 12 |  |  |
| 2917 | male | 55 | positive | 300.00 | 16.41 | 6.76 | 18.28 | negative | 15 |  |  |
| 2918 | male | 55 | positive | 93.50 | 13.55 | 6.42 | 6.90 | negative | 15 |  |  |
| 2919 | male | 55 | negative | 83.60 | 9.90 | 4.10 | 8.44 | negative | 12 |  |  |
| 2920 | male | 55 | positive | 94.10 | 15.57 | 3.83 | 6.04 | negative | 13 |  |  |
| 2921 | male | 55 | positive | 67.29 | 10.99 | 5.76 | 6.12 | negative | 15 |  |  |
| 2922 | male | 55 | positive | 61.56 | 15.31 | 4.69 | 4.02 | negative | 13 |  |  |
| 2923 | male | 55 | negative | 56.61 | 6.01 | 4.03 | 9.42 | negative | 12 |  |  |
| 2924 | male | 55 | negative | 80.98 | 10.26 | 4.97 | 7.89 | negative | 12 |  |  |
| 2925 | male | 55 | positive | 97.43 | 6.38 | 5.04 | 15.27 | negative | 13 |  |  |
| 2926 | male | 55 | negative | 107.28 | 7.30 | 7.51 | 14.70 | negative | 14 |  |  |
| 2927 | male | 55 | negative | 108.69 | 10.00 | 6.33 | 10.87 | negative | 14 |  |  |
| 2928 | male | 55 | positive | 111.08 | 6.87 | 6.28 | 16.17 | negative | 15 |  |  |
| 2929 | male | 55 | negative | 82.02 | 3.94 | 3.61 | 20.82 | negative | 12 |  |  |
| 2930 | male | 55 | negative | 120.83 | 12.63 | 4.98 | 9.57 | negative | 12 |  |  |
| 2931 | male | 55 | positive | 69.13 | 13.72 | 3.28 | 5.04 | negative | 13 |  |  |
| 2932 | male | 55 | positive | 127.39 | 14.02 | 5.56 | 9.09 | negative | 13 |  |  |
| 2933 | female | 55 | negative | 56.55 | 20.79 | 32.99 | 2.72 | negative | 13 |  |  |
| 2934 | male | 55 | negative | 101.17 | 12.53 | 5.70 | 8.07 | negative | 12 |  |  |
| 2935 | male | 55 | negative | 122.30 | 7.92 | 6.86 | 15.44 | negative | 14 |  |  |
| 2936 | male | 55 | positive | 284.05 | 29.28 | 7.51 | 9.70 | negative | 15 |  |  |
| 2937 | male | 55 | positive | 124.39 | 12.56 | 6.28 | 9.90 | negative | 15 |  |  |
| 2938 | male | 55 | positive | 177.64 | 20.35 | 7.41 | 8.73 | negative | 15 |  |  |
| 2939 | male | 55 | positive | 111.36 | 13.91 | 3.81 | 8.01 | negative | 13 |  |  |
| 2940 | male | 55 | positive | 135.89 | 18.55 | 5.15 | 7.33 | negative | 13 |  |  |
| 2941 | male | 55 | negative | 98.61 | 11.90 | 7.40 | 8.29 | negative | 14 |  |  |
| 2942 | male | 55 | positive | 81.65 | 15.99 | 5.74 | 5.11 | negative | 15 |  |  |
| 2943 | male | 55 | negative | 162.21 | 12.32 | 3.54 | 13.17 | negative | 12 |  |  |
| 2944 | male | 55 | positive | 204.06 | 11.65 | 3.43 | 17.52 | negative | 13 |  |  |
| 2945 | male | 55 | positive | 162.20 | 16.65 | 5.07 | 9.74 | negative | 13 |  |  |
| 2946 | male | 55 | negative | 108.91 | 17.20 | 5.16 | 6.33 | negative | 12 |  |  |
| 2947 | male | 55 | positive | 151.41 | 12.05 | 31.80 | 12.57 | negative | 15 |  |  |
| 2948 | male | 55 | negative | 99.95 | 6.74 | 5.34 | 14.83 | negative | 12 |  |  |
| 2949 | male | 55 | negative | 87.58 | 7.28 | 4.99 | 12.03 | negative | 12 |  |  |
| 2950 | male | 55 | negative | 184.93 | 7.14 | 4.23 | 25.90 | negative | 12 |  |  |
| 2951 | male | 55 | negative | 96.19 | 8.50 | 4.27 | 11.32 | negative | 12 |  |  |
| 2952 | male | 55 | positive | 123.35 | 15.77 | 5.61 | 7.82 | negative | 13 |  |  |
| 2953 | male | 55 | positive | 90.33 | 11.98 | 3.45 | 7.54 | negative | 13 |  |  |
| 2954 | male | 55 | positive | 300.00 | 32.60 | 6.33 | 9.20 | negative | 15 |  |  |
| 2955 | male | 55 | negative | 112.55 | 17.67 | 5.88 | 6.37 | negative | 14 |  |  |
| 2956 | male | 55 | negative | 114.27 | 8.16 | 4.57 | 14.00 | negative | 12 |  |  |
| 2957 | male | 55 | negative | 126.13 | 8.59 | 3.61 | 14.68 | negative | 12 |  |  |
| 2958 | male | 55 | negative | 143.17 | 10.68 | 7.58 | 13.41 | negative | 14 |  |  |
| 2959 | male | 55 | negative | 99.65 | 14.12 | 3.61 | 7.06 | negative | 12 |  |  |
| 2960 | male | 55 | negative | 155.96 | 11.90 | 4.02 | 13.11 | negative | 12 |  |  |
| 2961 | male | 55 | positive | 31.18 | 5.02 | 5.12 | 6.21 | negative | 13 |  |  |
| 2962 | male | 55 | positive | 110.60 | 13.03 | 5.23 | 8.49 | negative | 13 |  |  |
| 2963 | male | 55 | positive | 107.90 | 16.30 | 5.50 | 6.62 | negative | 13 |  |  |
| 2964 | male | 55 | positive | 117.76 | 20.22 | 4.53 | 5.82 | negative | 13 |  |  |
| 2965 | male | 55 | positive | 154.12 | 11.70 | 3.96 | 13.17 | negative | 13 |  |  |
| 2966 | male | 55 | negative | 149.05 | 11.53 | 3.44 | 12.93 | negative | 12 |  |  |
| 2967 | male | 55 | positive | 47.33 | 6.42 | 4.44 | 7.37 | negative | 13 |  |  |
| 2968 | male | 55 | positive | 185.77 | 23.98 | 3.52 | 7.75 | negative | 13 |  |  |
| 2969 | male | 55 | negative | 120.81 | 7.54 | 4.59 | 16.02 | negative | 12 |  |  |
| 2970 | male | 55 | positive | 103.27 | 13.36 | 7.60 | 7.73 | negative | 15 |  |  |
| 2971 | male | 55 | negative | 170.32 | 15.89 | 5.51 | 10.72 | negative | 12 |  |  |
| 2972 | male | 55 | positive | 114.80 | 14.90 | 4.20 | 7.70 | negative | 13 |  |  |
| 2973 | male | 55 | negative | 67.88 | 7.84 | 3.45 | 8.66 | negative | 12 |  |  |
| 2974 | male | 55 | positive | 147.98 | 10.24 | 5.71 | 14.45 | negative | 15 |  |  |
| 2975 | female | 55 | negative | 27.46 | 7.86 | 60.00 | 3.49 | negative | 13 |  |  |
| 2976 | male | 55 | positive | 300.00 | 17.04 | 4.01 | 17.61 | negative | 13 |  |  |
| 2977 | male | 55 | positive | 97.19 | 9.54 | 5.85 | 10.19 | negative | 15 |  |  |
| 2978 | male | 55 | positive | 155.72 | 16.89 | 5.71 | 9.22 | negative | 15 |  |  |
| 2979 | male | 55 | negative | 156.06 | 11.37 | 4.54 | 13.73 | negative | 12 |  |  |
| 2980 | male | 55 | positive | 138.30 | 16.70 | 5.20 | 8.28 | negative | 13 |  |  |
| 2981 | male | 55 | negative | 144.32 | 8.90 | 5.38 | 16.22 | negative | 12 |  |  |
| 2982 | female | 55 | positive | 55.24 | 17.18 | 3.68 | 3.22 | negative | 12 |  |  |
| 2983 | male | 55 | positive | 125.55 | 7.73 | 5.78 | 16.24 | negative | 15 |  |  |
| 2984 | male | 55 | negative | 60.75 | 5.14 | 7.44 | 11.82 | negative | 14 |  |  |
| 2985 | male | 55 | positive | 97.61 | 16.55 | 7.14 | 5.90 | negative | 15 |  |  |
| 2986 | male | 55 | negative | 103.77 | 6.35 | 3.22 | 16.34 | negative | 12 |  |  |
| 2987 | male | 55 | positive | 108.29 | 9.57 | 5.27 | 11.32 | negative | 13 |  |  |
| 2988 | male | 55 | negative | 262.46 | 22.81 | 5.10 | 11.51 | negative | 12 |  |  |
| 2989 | male | 55 | positive | 118.02 | 8.05 | 4.81 | 14.66 | negative | 13 |  |  |
| 2990 | male | 55 | positive | 26.14 | 20.92 | 6.31 | 1.25 | negative | 18 |  |  |
| 2991 | male | 55 | positive | 163.84 | 22.66 | 5.38 | 7.23 | negative | 13 |  |  |
| 2992 | male | 55 | negative | 24.55 | 10.07 | 49.47 | 2.44 | negative | 17 |  |  |
| 2993 | male | 55 | positive | 99.50 | 17.29 | 8.09 | 5.75 | negative | 15 |  |  |
| 2994 | male | 55 | positive | 189.27 | 18.97 | 7.22 | 9.98 | negative | 15 |  |  |
| 2995 | male | 55 | negative | 96.80 | 14.20 | 3.70 | 6.82 | negative | 12 |  |  |
| 2996 | male | 55 | negative | 92.67 | 3.72 | 3.25 | 24.91 | negative | 12 |  |  |
| 2997 | male | 55 | negative | 84.23 | 6.24 | 3.60 | 13.50 | negative | 12 |  |  |
| 2998 | male | 55 | negative | 334.40 | 26.80 | 36.30 | 12.48 | negative | 14 |  |  |
| 2999 | male | 55 | positive | 105.09 | 21.77 | 6.55 | 4.83 | negative | 15 |  |  |
| 3000 | female | 55 | negative | 42.24 | 11.30 | 57.89 | 3.74 | negative | 13 |  |  |
| 3001 | male | 55 | positive | 96.85 | 13.87 | 3.57 | 6.98 | negative | 13 |  |  |
| 3002 | male | 55 | negative | 190.22 | 13.07 | 3.29 | 14.55 | negative | 12 |  |  |
| 3003 | male | 55 | negative | 152.34 | 15.76 | 4.16 | 9.67 | negative | 12 |  |  |
| 3004 | male | 55 | positive | 53.72 | 16.00 | 5.58 | 3.36 | negative | 16 |  |  |
| 3005 | male | 55 | positive | 130.80 | 12.60 | 5.30 | 10.38 | negative | 13 |  |  |
| 3006 | male | 55 | positive | 295.24 | 28.92 | 5.87 | 10.21 | negative | 15 |  |  |
| 3007 | male | 55 | positive | 116.18 | 12.40 | 6.67 | 9.37 | negative | 15 |  |  |
| 3008 | male | 55 | positive | 89.49 | 9.47 | 3.84 | 9.45 | negative | 13 |  |  |
| 3009 | male | 55 | negative | 131.01 | 14.26 | 7.75 | 9.19 | negative | 14 |  |  |
| 3010 | male | 55 | positive | 140.73 | 15.74 | 6.05 | 8.94 | negative | 15 |  |  |
| 3011 | male | 55 | positive | 287.20 | 67.60 | 36.00 | 4.25 | negative | 15 |  |  |
| 3012 | male | 55 | negative | 127.33 | 9.69 | 3.52 | 13.14 | negative | 12 |  |  |
| 3013 | female | 55 | negative | 23.94 | 24.57 | 60.00 | 0.97 | negative | 13 |  |  |
| 3014 | male | 55 | positive | 76.90 | 6.31 | 6.35 | 12.19 | negative | 15 |  |  |
| 3015 | male | 55 | negative | 285.63 | 27.40 | 4.75 | 10.42 | negative | 12 |  |  |
| 3016 | male | 55 | positive | 130.83 | 16.48 | 4.19 | 7.94 | negative | 13 |  |  |
| 3017 | male | 55 | positive | 195.96 | 18.55 | 4.79 | 10.56 | negative | 13 |  |  |
| 3018 | male | 55 | negative | 45.67 | 5.07 | 3.73 | 9.01 | negative | 12 |  |  |
| 3019 | male | 55 | negative | 168.04 | 11.40 | 7.63 | 14.74 | negative | 14 |  |  |
| 3020 | male | 55 | positive | 71.66 | 10.76 | 4.68 | 6.66 | negative | 13 |  |  |
| 3021 | male | 55 | positive | 163.73 | 10.19 | 4.57 | 16.07 | negative | 13 |  |  |
| 3022 | male | 55 | negative | 64.22 | 11.28 | 4.73 | 5.69 | negative | 12 |  |  |
| 3023 | female | 55 | positive | 53.36 | 14.80 | 3.27 | 3.61 | negative | 12 |  |  |
| 3024 | male | 55 | positive | 85.88 | 11.12 | 5.82 | 7.72 | negative | 15 |  |  |
| 3025 | male | 55 | positive | 250.63 | 16.31 | 4.12 | 15.37 | negative | 13 |  |  |
| 3026 | male | 55 | positive | 78.17 | 14.08 | 7.73 | 5.55 | negative | 15 |  |  |
| 3027 | male | 55 | negative | 116.46 | 6.91 | 3.30 | 16.85 | negative | 12 |  |  |
| 3028 | male | 55 | positive | 150.99 | 13.11 | 6.17 | 11.52 | negative | 15 |  |  |
| 3029 | male | 55 | positive | 118.09 | 19.49 | 3.46 | 6.06 | negative | 13 |  |  |
| 3030 | male | 55 | negative | 133.90 | 20.70 | 7.30 | 6.47 | negative | 14 |  |  |
| 3031 | male | 55 | positive | 82.88 | 7.38 | 3.59 | 11.23 | negative | 13 |  |  |
| 3032 | male | 55 | positive | 133.13 | 17.78 | 4.89 | 7.49 | negative | 13 |  |  |
| 3033 | male | 55 | negative | 118.64 | 10.45 | 5.27 | 11.35 | negative | 12 |  |  |
| 3034 | male | 55 | positive | 61.49 | 10.99 | 4.76 | 5.60 | negative | 13 |  |  |
| 3035 | male | 55 | negative | 100.10 | 11.00 | 7.60 | 9.10 | negative | 14 |  |  |
| 3036 | male | 55 | positive | 131.20 | 13.40 | 6.60 | 9.79 | negative | 15 |  |  |
| 3037 | male | 55 | negative | 61.53 | 9.48 | 30.10 | 6.49 | negative | 14 |  |  |
| 3038 | male | 55 | negative | 83.59 | 7.73 | 3.25 | 10.81 | negative | 12 |  |  |
| 3039 | male | 55 | negative | 226.66 | 18.64 | 6.20 | 12.16 | negative | 14 |  |  |
| 3040 | male | 55 | positive | 186.02 | 21.29 | 4.64 | 8.74 | negative | 13 |  |  |
| 3041 | male | 55 | positive | 137.18 | 15.73 | 5.90 | 8.72 | negative | 15 |  |  |
| 3042 | male | 55 | positive | 116.08 | 10.33 | 5.49 | 11.24 | negative | 13 |  |  |
| 3043 | male | 55 | positive | 111.99 | 11.73 | 6.24 | 9.55 | negative | 15 |  |  |
| 3044 | male | 55 | negative | 154.66 | 7.98 | 6.59 | 19.38 | negative | 14 |  |  |
| 3045 | male | 55 | positive | 148.87 | 11.68 | 4.21 | 12.75 | negative | 13 |  |  |
| 3046 | male | 55 | negative | 64.89 | 3.26 | 3.42 | 19.90 | negative | 12 |  |  |
| 3047 | male | 55 | positive | 17.58 | 11.32 | 60.00 | 1.55 | negative | 18 |  |  |
| 3048 | male | 55 | positive | 190.49 | 20.00 | 5.48 | 9.52 | negative | 13 |  |  |
| 3049 | male | 55 | negative | 23.19 | 7.13 | 57.45 | 3.25 | negative | 17 |  |  |
| 3050 | male | 55 | negative | 82.21 | 9.19 | 7.49 | 8.95 | negative | 14 |  |  |
| 3051 | male | 55 | positive | 124.34 | 10.50 | 6.71 | 11.84 | negative | 15 |  |  |
| 3052 | male | 55 | negative | 77.06 | 7.08 | 3.26 | 10.88 | negative | 12 |  |  |
| 3053 | male | 55 | positive | 90.82 | 8.16 | 6.32 | 11.13 | negative | 15 |  |  |
| 3054 | male | 55 | negative | 278.32 | 16.52 | 5.10 | 16.85 | negative | 12 |  |  |
| 3055 | male | 55 | positive | 98.24 | 9.67 | 5.56 | 10.16 | negative | 13 |  |  |
| 3056 | male | 55 | positive | 111.40 | 13.08 | 6.30 | 8.52 | negative | 15 |  |  |
| 3057 | male | 55 | negative | 187.46 | 15.57 | 4.73 | 12.04 | negative | 12 |  |  |
| 3058 | male | 55 | positive | 142.87 | 11.43 | 7.41 | 12.50 | negative | 15 |  |  |
| 3059 | male | 55 | negative | 98.57 | 4.57 | 3.94 | 21.57 | negative | 12 |  |  |
| 3060 | male | 55 | negative | 163.18 | 11.95 | 7.61 | 13.66 | negative | 14 |  |  |
| 3061 | female | 55 | negative | 44.70 | 19.79 | 36.61 | 2.26 | negative | 13 |  |  |
| 3062 | male | 55 | negative | 112.53 | 6.93 | 5.71 | 16.24 | negative | 14 |  |  |
| 3063 | male | 55 | positive | 209.93 | 17.61 | 4.82 | 11.92 | negative | 13 |  |  |
| 3064 | male | 55 | positive | 300.00 | 34.56 | 8.10 | 8.68 | negative | 15 |  |  |
| 3065 | male | 55 | positive | 81.96 | 9.64 | 5.14 | 8.50 | negative | 13 |  |  |
| 3066 | male | 55 | negative | 235.90 | 18.67 | 6.03 | 12.64 | negative | 14 |  |  |
| 3067 | male | 55 | positive | 122.23 | 8.79 | 3.38 | 13.91 | negative | 13 |  |  |
| 3068 | male | 55 | negative | 235.63 | 10.62 | 6.26 | 22.19 | negative | 14 |  |  |
| 3069 | male | 55 | negative | 100.50 | 10.56 | 3.31 | 9.52 | negative | 12 |  |  |
| 3070 | male | 55 | positive | 193.58 | 8.90 | 5.19 | 21.75 | negative | 13 |  |  |
| 3071 | male | 55 | negative | 145.57 | 7.37 | 4.70 | 19.75 | negative | 12 |  |  |
| 3072 | male | 55 | negative | 76.97 | 8.39 | 4.00 | 9.17 | negative | 12 |  |  |
| 3073 | male | 55 | negative | 54.97 | 11.89 | 3.94 | 4.62 | negative | 12 |  |  |
| 3074 | male | 55 | negative | 124.14 | 12.32 | 3.65 | 10.08 | negative | 12 |  |  |
| 3075 | male | 55 | negative | 124.82 | 10.98 | 3.26 | 11.37 | negative | 12 |  |  |
| 3076 | male | 55 | positive | 113.89 | 7.48 | 7.03 | 15.23 | negative | 15 |  |  |
| 3077 | male | 55 | negative | 87.77 | 18.58 | 4.48 | 4.72 | negative | 12 |  |  |
| 3078 | male | 55 | negative | 102.50 | 10.60 | 4.90 | 9.67 | negative | 12 |  |  |
| 3079 | male | 55 | negative | 90.33 | 3.68 | 2.19 | 24.55 | negative | 12 |  |  |
| 3080 | male | 55 | positive | 93.91 | 12.42 | 3.09 | 7.56 | negative | 13 |  |  |
| 3081 | male | 55 | negative | 47.19 | 6.80 | 2.70 | 6.94 | negative | 12 |  |  |
| 3082 | male | 55 | negative | 106.92 | 9.34 | 2.56 | 11.45 | negative | 12 |  |  |
| 3083 | male | 55 | positive | 60.20 | 12.20 | 19.20 | 4.93 | negative | 15 |  |  |
| 3084 | male | 55 | positive | 146.00 | 9.21 | 2.77 | 15.85 | negative | 13 |  |  |
| 3085 | male | 55 | positive | 75.22 | 18.86 | 2.30 | 3.99 | negative | 13 |  |  |
| 3086 | male | 55 | negative | 109.12 | 10.38 | 2.99 | 10.51 | negative | 12 |  |  |
| 3087 | male | 55 | negative | 79.79 | 5.52 | 2.72 | 14.45 | negative | 12 |  |  |
| 3088 | male | 55 | negative | 129.81 | 7.27 | 2.35 | 17.86 | negative | 12 |  |  |
| 3089 | male | 55 | positive | 173.73 | 9.95 | 2.07 | 17.46 | negative | 13 |  |  |
| 3090 | male | 55 | negative | 148.46 | 13.53 | 2.54 | 10.97 | negative | 12 |  |  |
| 3091 | male | 55 | negative | 94.33 | 4.85 | 2.81 | 19.45 | negative | 12 |  |  |
| 3092 | male | 55 | positive | 105.56 | 16.33 | 24.66 | 6.46 | negative | 15 |  |  |
| 3093 | male | 55 | negative | 250.41 | 17.44 | 23.08 | 14.36 | negative | 14 |  |  |
| 3094 | male | 55 | positive | 112.24 | 16.04 | 21.44 | 7.00 | negative | 15 |  |  |
| 3095 | male | 55 | negative | 57.89 | 2.51 | 2.46 | 23.06 | negative | 12 |  |  |
| 3096 | male | 55 | negative | 108.85 | 7.59 | 2.05 | 14.34 | negative | 12 |  |  |
| 3097 | male | 55 | negative | 125.47 | 8.86 | 2.45 | 14.16 | negative | 12 |  |  |
| 3098 | male | 55 | negative | 53.35 | 8.02 | 2.46 | 6.65 | negative | 12 |  |  |
| 3099 | male | 55 | negative | 75.09 | 5.16 | 2.27 | 14.55 | negative | 12 |  |  |
| 3100 | male | 55 | negative | 95.95 | 6.80 | 2.14 | 14.11 | negative | 12 |  |  |
| 3101 | male | 55 | positive | 281.09 | 17.98 | 2.27 | 15.63 | negative | 13 |  |  |
| 3102 | female | 55 | positive | 17.91 | 15.62 | 2.60 | 1.15 | negative | 12 |  |  |
| 3103 | male | 55 | negative | 107.92 | 7.45 | 2.24 | 14.49 | negative | 12 |  |  |
| 3104 | male | 55 | negative | 160.78 | 14.54 | 2.73 | 11.06 | negative | 12 |  |  |
| 3105 | male | 55 | positive | 61.56 | 5.97 | 3.20 | 10.31 | negative | 13 |  |  |
| 3106 | male | 55 | negative | 101.43 | 7.21 | 2.57 | 14.07 | negative | 12 |  |  |
| 3107 | male | 55 | negative | 50.40 | 6.60 | 2.20 | 7.64 | negative | 12 |  |  |
| 3108 | male | 55 | negative | 72.55 | 8.78 | 2.35 | 8.26 | negative | 12 |  |  |
| 3109 | male | 55 | negative | 101.18 | 7.75 | 2.37 | 13.06 | negative | 12 |  |  |
| 3110 | male | 55 | positive | 71.21 | 15.84 | 3.16 | 4.50 | negative | 13 |  |  |
| 3111 | male | 55 | positive | 83.03 | 8.28 | 18.61 | 10.03 | negative | 15 |  |  |
| 3112 | male | 55 | positive | 112.23 | 10.67 | 3.17 | 10.52 | negative | 13 |  |  |
| 3113 | male | 55 | positive | 174.11 | 23.74 | 2.18 | 7.33 | negative | 13 |  |  |
| 3114 | male | 55 | negative | 49.03 | 3.93 | 2.90 | 12.48 | negative | 12 |  |  |
| 3115 | male | 55 | negative | 90.91 | 9.45 | 2.29 | 9.62 | negative | 12 |  |  |
| 3116 | male | 55 | negative | 97.66 | 7.62 | 3.02 | 12.82 | negative | 12 |  |  |
| 3117 | male | 55 | negative | 133.27 | 11.60 | 2.20 | 11.49 | negative | 12 |  |  |
| 3118 | male | 55 | negative | 98.91 | 7.63 | 2.90 | 12.96 | negative | 12 |  |  |
| 3119 | male | 55 | negative | 66.40 | 6.90 | 2.30 | 9.62 | negative | 12 |  |  |
| 3120 | male | 55 | positive | 58.03 | 12.51 | 2.55 | 4.64 | negative | 13 |  |  |
| 3121 | male | 55 | negative | 135.71 | 7.14 | 2.06 | 19.01 | negative | 12 |  |  |
| 3122 | male | 55 | negative | 92.91 | 9.69 | 2.56 | 9.59 | negative | 12 |  |  |
| 3123 | male | 55 | negative | 130.97 | 9.33 | 2.33 | 14.04 | negative | 12 |  |  |
| 3124 | male | 55 | negative | 120.84 | 8.96 | 2.84 | 13.49 | negative | 12 |  |  |
| 3125 | male | 55 | negative | 242.89 | 32.04 | 28.25 | 7.58 | negative | 14 |  |  |
| 3126 | male | 55 | negative | 140.97 | 14.42 | 18.57 | 9.78 | negative | 14 |  |  |
| 3127 | male | 55 | negative | 117.34 | 10.02 | 2.96 | 11.71 | negative | 12 |  |  |
| 3128 | male | 55 | positive | 133.73 | 11.36 | 2.06 | 11.77 | negative | 13 |  |  |
| 3129 | male | 55 | positive | 291.23 | 25.72 | 2.50 | 11.32 | negative | 13 |  |  |
| 3130 | female | 55 | positive | 111.46 | 35.58 | 2.08 | 3.13 | negative | 12 |  |  |
| 3131 | male | 55 | negative | 80.96 | 3.73 | 2.08 | 21.71 | negative | 12 |  |  |
| 3132 | male | 55 | negative | 147.10 | 13.30 | 3.10 | 11.06 | negative | 12 |  |  |
| 3133 | male | 55 | positive | 88.84 | 9.73 | 3.20 | 9.13 | negative | 13 |  |  |
| 3134 | male | 55 | negative | 100.17 | 7.79 | 2.24 | 12.86 | negative | 12 |  |  |
| 3135 | male | 55 | negative | 74.35 | 8.16 | 2.68 | 9.11 | negative | 12 |  |  |
| 3136 | male | 55 | negative | 264.65 | 14.49 | 2.50 | 18.26 | negative | 12 |  |  |
| 3137 | male | 55 | negative | 88.16 | 4.03 | 2.27 | 21.88 | negative | 12 |  |  |
| 3138 | male | 55 | negative | 85.60 | 13.70 | 3.20 | 6.25 | negative | 12 |  |  |
| 3139 | male | 55 | positive | 91.49 | 8.22 | 3.02 | 11.13 | negative | 13 |  |  |
| 3140 | male | 55 | negative | 94.21 | 5.82 | 2.37 | 16.19 | negative | 12 |  |  |
| 3141 | male | 55 | negative | 118.56 | 5.54 | 2.27 | 21.40 | negative | 12 |  |  |
| 3142 | male | 55 | negative | 77.20 | 5.78 | 3.20 | 13.36 | negative | 12 |  |  |
| 3143 | male | 55 | positive | 155.77 | 17.56 | 21.60 | 8.87 | negative | 15 |  |  |
| 3144 | male | 55 | negative | 7.63 | 8.39 | 22.25 | 0.91 | negative | 17 |  |  |
| 3145 | male | 55 | negative | 164.04 | 8.24 | 2.78 | 19.91 | negative | 12 |  |  |
| 3146 | male | 55 | negative | 102.93 | 3.86 | 2.07 | 26.67 | negative | 12 |  |  |
| 3147 | male | 55 | positive | 128.69 | 12.99 | 2.94 | 9.91 | negative | 13 |  |  |
| 3148 | male | 55 | negative | 101.80 | 14.36 | 3.02 | 7.09 | negative | 12 |  |  |
| 3149 | male | 55 | negative | 137.76 | 12.31 | 2.32 | 11.19 | negative | 12 |  |  |
| 3150 | male | 55 | negative | 86.58 | 5.49 | 2.95 | 15.77 | negative | 12 |  |  |
| 3151 | male | 55 | negative | 181.77 | 21.13 | 2.60 | 8.60 | negative | 12 |  |  |
| 3152 | male | 55 | negative | 70.32 | 5.89 | 2.16 | 11.94 | negative | 12 |  |  |
| 3153 | male | 55 | negative | 62.19 | 7.64 | 2.69 | 8.14 | negative | 12 |  |  |
| 3154 | male | 55 | negative | 97.02 | 6.26 | 2.20 | 15.50 | negative | 12 |  |  |
| 3155 | male | 55 | negative | 80.49 | 6.43 | 2.10 | 12.52 | negative | 12 |  |  |
| 3156 | male | 55 | negative | 83.77 | 8.16 | 2.35 | 10.27 | negative | 12 |  |  |
| 3157 | male | 55 | negative | 178.74 | 10.71 | 2.33 | 16.69 | negative | 12 |  |  |
| 3158 | male | 55 | negative | 145.51 | 5.50 | 2.84 | 26.46 | negative | 12 |  |  |
| 3159 | male | 55 | negative | 115.38 | 7.66 | 2.93 | 15.06 | negative | 12 |  |  |
| 3160 | male | 55 | negative | 135.15 | 7.14 | 2.70 | 18.93 | negative | 12 |  |  |
| 3161 | male | 55 | negative | 121.30 | 9.20 | 2.54 | 13.18 | negative | 12 |  |  |
| 3162 | male | 55 | positive | 85.50 | 14.70 | 2.50 | 5.82 | negative | 13 |  |  |
| 3163 | male | 55 | negative | 83.02 | 11.07 | 2.78 | 7.50 | negative | 12 |  |  |
| 3164 | male | 55 | negative | 86.13 | 9.21 | 2.31 | 9.35 | negative | 12 |  |  |
| 3165 | male | 55 | negative | 103.50 | 6.59 | 2.09 | 15.71 | negative | 12 |  |  |
| 3166 | male | 55 | negative | 93.97 | 8.82 | 2.18 | 10.65 | negative | 12 |  |  |
| 3167 | male | 55 | negative | 200.54 | 9.29 | 2.34 | 21.59 | negative | 12 |  |  |
| 3168 | male | 55 | negative | 136.06 | 8.08 | 2.25 | 16.84 | negative | 12 |  |  |
| 3169 | male | 55 | negative | 136.09 | 5.95 | 2.07 | 22.87 | negative | 12 |  |  |
| 3170 | male | 55 | negative | 166.46 | 11.52 | 2.79 | 14.45 | negative | 12 |  |  |
| 3171 | male | 55 | positive | 228.44 | 27.95 | 2.98 | 8.17 | negative | 13 |  |  |
| 3172 | male | 55 | negative | 129.49 | 10.46 | 2.80 | 12.38 | negative | 12 |  |  |
| 3173 | male | 55 | positive | 232.70 | 22.23 | 2.96 | 10.47 | negative | 13 |  |  |
| 3174 | male | 55 | negative | 132.80 | 12.06 | 2.54 | 11.01 | negative | 12 |  |  |
| 3175 | male | 55 | negative | 119.99 | 11.53 | 2.27 | 10.41 | negative | 12 |  |  |
| 3176 | male | 55 | negative | 75.43 | 6.58 | 2.04 | 11.46 | negative | 12 |  |  |
| 3177 | male | 55 | positive | 89.02 | 14.56 | 2.54 | 6.11 | negative | 13 |  |  |
| 3178 | male | 55 | positive | 149.48 | 14.27 | 2.19 | 10.48 | negative | 13 |  |  |
| 3179 | male | 55 | positive | 56.34 | 15.52 | 27.47 | 3.63 | negative | 18 |  |  |
| 3180 | male | 55 | negative | 141.93 | 6.42 | 2.03 | 22.11 | negative | 12 |  |  |
| 3181 | male | 55 | negative | 112.67 | 7.01 | 3.14 | 16.07 | negative | 12 |  |  |
| 3182 | male | 55 | negative | 84.00 | 4.20 | 2.90 | 20.00 | negative | 12 |  |  |
| 3183 | male | 55 | negative | 150.50 | 14.60 | 2.60 | 10.31 | negative | 12 |  |  |
| 3184 | male | 55 | negative | 94.07 | 3.46 | 2.35 | 27.19 | negative | 12 |  |  |
| 3185 | male | 55 | negative | 111.20 | 9.42 | 3.07 | 11.80 | negative | 12 |  |  |
| 3186 | male | 55 | negative | 120.74 | 11.45 | 2.39 | 10.54 | negative | 12 |  |  |
| 3187 | male | 55 | negative | 70.90 | 8.65 | 2.87 | 8.20 | negative | 12 |  |  |
| 3188 | male | 55 | negative | 81.80 | 9.50 | 3.20 | 8.61 | negative | 12 |  |  |
| 3189 | male | 55 | negative | 182.50 | 9.55 | 2.15 | 19.11 | negative | 12 |  |  |
| 3190 | male | 55 | negative | 96.26 | 11.06 | 3.19 | 8.70 | negative | 12 |  |  |
| 3191 | male | 55 | negative | 167.04 | 15.26 | 2.26 | 10.95 | negative | 12 |  |  |
| 3192 | male | 55 | negative | 85.32 | 5.28 | 2.19 | 16.16 | negative | 12 |  |  |
| 3193 | male | 55 | negative | 58.45 | 4.83 | 3.04 | 12.10 | negative | 12 |  |  |
| 3194 | male | 54 | negative | 109.59 | 11.61 | 7.20 | 9.44 | negative | 14 |  |  |
| 3195 | male | 54 | positive | 131.34 | 14.49 | 6.95 | 9.06 | negative | 15 |  |  |
| 3196 | male | 54 | positive | 51.76 | 9.82 | 8.47 | 5.27 | negative | 15 |  |  |
| 3197 | male | 55 | negative | 96.00 | 6.67 | 1.60 | 14.39 | negative | 12 |  |  |
| 3198 | male | 55 | negative | 87.41 | 6.01 | 1.74 | 14.54 | negative | 12 |  |  |
| 3199 | male | 54 | positive | 97.80 | 5.15 | 5.60 | 18.99 | negative | 13 |  |  |
| 3200 | male | 54 | positive | 251.54 | 18.97 | 8.93 | 13.26 | negative | 15 |  |  |
| 3201 | male | 55 | negative | 83.41 | 9.43 | 1.89 | 8.85 | negative | 12 |  |  |
| 3202 | male | 54 | negative | 107.32 | 8.84 | 6.79 | 12.14 | negative | 14 |  |  |
| 3203 | male | 54 | positive | 110.15 | 11.95 | 5.77 | 9.22 | negative | 15 |  |  |
| 3204 | male | 54 | positive | 76.57 | 10.72 | 9.90 | 7.14 | negative | 15 |  |  |
| 3205 | male | 54 | negative | 245.72 | 14.70 | 8.17 | 16.72 | negative | 14 |  |  |
| 3206 | male | 55 | negative | 90.65 | 6.34 | 1.89 | 14.30 | negative | 12 |  |  |
| 3207 | male | 55 | negative | 80.37 | 12.08 | 1.58 | 6.65 | negative | 12 |  |  |
| 3208 | male | 54 | positive | 64.38 | 19.25 | 7.27 | 3.34 | negative | 18 |  |  |
| 3209 | male | 55 | positive | 57.50 | 13.70 | 15.00 | 4.20 | negative | 15 |  |  |
| 3210 | male | 54 | positive | 130.47 | 28.71 | 7.30 | 4.54 | negative | 15 |  |  |
| 3211 | male | 55 | negative | 133.59 | 9.71 | 18.40 | 13.76 | negative | 14 |  |  |
| 3212 | male | 55 | negative | 278.33 | 13.44 | 13.88 | 20.71 | negative | 14 |  |  |
| 3213 | male | 54 | negative | 126.06 | 9.23 | 8.26 | 13.66 | negative | 14 |  |  |
| 3214 | male | 54 | positive | 106.38 | 14.77 | 7.41 | 7.20 | negative | 15 |  |  |
| 3215 | male | 55 | negative | 141.58 | 8.10 | 1.85 | 17.48 | negative | 12 |  |  |
| 3216 | male | 54 | positive | 103.42 | 11.86 | 5.42 | 8.72 | negative | 13 |  |  |
| 3217 | female | 54 | negative | 8.06 | 4.89 | 60.00 | 1.65 | negative | 13 |  |  |
| 3218 | male | 55 | negative | 205.00 | 21.20 | 11.80 | 9.67 | negative | 14 |  |  |
| 3219 | male | 54 | negative | 200.79 | 21.46 | 5.68 | 9.36 | negative | 12 |  |  |
| 3220 | male | 54 | positive | 246.28 | 21.16 | 6.49 | 11.64 | negative | 15 |  |  |
| 3221 | male | 55 | negative | 146.10 | 41.60 | 1.80 | 3.51 | negative | 15 |  |  |
| 3222 | male | 55 | negative | 62.66 | 16.78 | 1.80 | 3.73 | negative | 15 |  |  |
| 3223 | male | 55 | positive | 88.61 | 1.54 | 11.02 | 57.54 | negative | 15 |  |  |
| 3224 | male | 55 | negative | 134.85 | 16.23 | 13.36 | 8.31 | negative | 14 |  |  |
| 3225 | male | 55 | positive | 135.55 | 11.99 | 1.50 | 11.31 | negative | 13 |  |  |
| 3226 | male | 55 | positive | 128.20 | 16.30 | 13.40 | 7.87 | negative | 15 |  |  |
| 3227 | male | 54 | negative | 73.67 | 16.60 | 6.70 | 4.44 | negative | 14 |  |  |
| 3228 | male | 54 | positive | 95.56 | 13.08 | 9.31 | 7.31 | negative | 15 |  |  |
| 3229 | male | 54 | negative | 142.87 | 14.92 | 7.15 | 9.58 | negative | 14 |  |  |
| 3230 | male | 55 | positive | 112.57 | 9.41 | 1.54 | 11.96 | negative | 13 |  |  |
| 3231 | male | 54 | negative | 146.36 | 7.43 | 5.51 | 19.70 | negative | 12 |  |  |
| 3232 | male | 55 | negative | 104.72 | 9.11 | 1.57 | 11.50 | negative | 12 |  |  |
| 3233 | male | 55 | negative | 94.47 | 12.15 | 1.63 | 7.78 | negative | 12 |  |  |
| 3234 | male | 54 | positive | 190.45 | 12.85 | 8.62 | 14.82 | negative | 15 |  |  |
| 3235 | male | 55 | negative | 93.12 | 6.96 | 1.72 | 13.38 | negative | 12 |  |  |
| 3236 | male | 54 | negative | 85.50 | 6.24 | 6.09 | 13.70 | negative | 14 |  |  |
| 3237 | male | 54 | negative | 155.60 | 12.60 | 7.84 | 12.35 | negative | 14 |  |  |
| 3238 | male | 55 | negative | 163.25 | 8.68 | 12.29 | 18.81 | negative | 14 |  |  |
| 3239 | male | 54 | negative | 83.64 | 9.80 | 5.91 | 8.53 | negative | 14 |  |  |
| 3240 | male | 54 | positive | 117.77 | 15.10 | 9.29 | 7.80 | negative | 15 |  |  |
| 3241 | male | 54 | negative | 126.09 | 7.91 | 6.02 | 15.94 | negative | 14 |  |  |
| 3242 | male | 55 | positive | 147.54 | 20.12 | 12.39 | 7.33 | negative | 15 |  |  |
| 3243 | female | 54 | negative | 31.20 | 12.20 | 60.00 | 2.56 | negative | 13 |  |  |
| 3244 | male | 55 | positive | 182.02 | 24.23 | 13.96 | 7.51 | negative | 15 |  |  |
| 3245 | male | 54 | positive | 110.81 | 18.73 | 6.35 | 5.92 | negative | 15 |  |  |
| 3246 | male | 54 | negative | 151.14 | 9.39 | 52.13 | 16.10 | negative | 14 |  |  |
| 3247 | male | 54 | negative | 32.23 | 5.33 | 52.67 | 6.05 | negative | 14 |  |  |
| 3248 | male | 55 | negative | 91.38 | 12.61 | 13.10 | 7.25 | negative | 14 |  |  |
| 3249 | male | 54 | positive | 111.42 | 13.02 | 7.89 | 8.56 | negative | 15 |  |  |
| 3250 | male | 54 | positive | 141.54 | 17.80 | 5.36 | 7.95 | negative | 13 |  |  |
| 3251 | male | 54 | positive | 247.32 | 22.01 | 9.20 | 11.24 | negative | 15 |  |  |
| 3252 | male | 55 | negative | 100.09 | 5.17 | 1.50 | 19.36 | negative | 12 |  |  |
| 3253 | male | 55 | negative | 513.60 | 60.00 | 10.80 | 8.56 | negative | 14 |  |  |
| 3254 | male | 54 | positive | 173.17 | 16.15 | 6.84 | 10.72 | negative | 15 |  |  |
| 3255 | male | 55 | negative | 86.40 | 11.80 | 1.80 | 7.32 | negative | 12 |  |  |
| 3256 | male | 54 | positive | 300.00 | 15.20 | 6.15 | 19.74 | negative | 15 |  |  |
| 3257 | male | 55 | negative | 100.72 | 6.05 | 1.83 | 16.65 | negative | 12 |  |  |
| 3258 | male | 55 | negative | 43.21 | 12.41 | 16.52 | 3.48 | negative | 17 |  |  |
| 3259 | male | 55 | negative | 90.60 | 8.65 | 1.80 | 10.47 | negative | 12 |  |  |
| 3260 | male | 55 | negative | 93.41 | 17.83 | 12.35 | 5.24 | negative | 14 |  |  |
| 3261 | male | 54 | negative | 106.95 | 9.50 | 6.29 | 11.26 | negative | 14 |  |  |
| 3262 | male | 54 | positive | 79.20 | 12.68 | 6.69 | 6.25 | negative | 15 |  |  |
| 3263 | male | 54 | negative | 102.60 | 14.70 | 8.50 | 6.98 | negative | 14 |  |  |
| 3264 | male | 54 | negative | 116.36 | 11.15 | 7.15 | 10.44 | negative | 14 |  |  |
| 3265 | male | 55 | positive | 91.04 | 6.21 | 1.84 | 14.66 | negative | 13 |  |  |
| 3266 | male | 55 | positive | 123.70 | 20.00 | 16.20 | 6.18 | negative | 15 |  |  |
| 3267 | male | 54 | positive | 55.98 | 10.58 | 9.25 | 5.29 | negative | 15 |  |  |
| 3268 | male | 55 | negative | 78.52 | 6.52 | 1.54 | 12.04 | negative | 12 |  |  |
| 3269 | male | 55 | negative | 256.61 | 15.86 | 12.37 | 16.18 | negative | 14 |  |  |
| 3270 | male | 55 | negative | 127.14 | 7.72 | 1.94 | 16.47 | negative | 12 |  |  |
| 3271 | male | 54 | positive | 236.54 | 16.52 | 7.19 | 14.32 | negative | 15 |  |  |
| 3272 | male | 54 | positive | 58.70 | 8.40 | 5.60 | 6.99 | negative | 13 |  |  |
| 3273 | male | 55 | negative | 107.75 | 6.28 | 1.91 | 17.16 | negative | 12 |  |  |
| 3274 | male | 54 | negative | 84.61 | 11.53 | 9.02 | 7.34 | negative | 14 |  |  |
| 3275 | male | 55 | negative | 142.48 | 10.34 | 1.75 | 13.78 | negative | 12 |  |  |
| 3276 | male | 55 | negative | 87.70 | 8.90 | 1.50 | 9.85 | negative | 12 |  |  |
| 3277 | male | 54 | negative | 149.35 | 11.56 | 5.61 | 12.92 | negative | 12 |  |  |
| 3278 | male | 54 | positive | 150.77 | 12.44 | 6.22 | 12.12 | negative | 15 |  |  |
| 3279 | male | 54 | positive | 145.60 | 23.41 | 5.57 | 6.22 | negative | 13 |  |  |
| 3280 | male | 55 | positive | 92.54 | 33.80 | 11.95 | 2.74 | negative | 18 |  |  |
| 3281 | male | 55 | negative | 64.18 | 4.61 | 1.77 | 13.92 | negative | 12 |  |  |
| 3282 | male | 54 | positive | 67.80 | 7.52 | 6.79 | 9.02 | negative | 15 |  |  |
| 3283 | male | 54 | negative | 158.97 | 10.01 | 5.20 | 15.88 | negative | 12 |  |  |
| 3284 | male | 54 | positive | 110.00 | 9.79 | 7.32 | 11.24 | negative | 15 |  |  |
| 3285 | male | 54 | positive | 133.72 | 15.12 | 5.28 | 8.84 | negative | 13 |  |  |
| 3286 | male | 54 | negative | 138.26 | 9.05 | 5.30 | 15.28 | negative | 12 |  |  |
| 3287 | male | 55 | positive | 75.10 | 21.20 | 10.70 | 3.54 | negative | 18 |  |  |
| 3288 | male | 54 | negative | 183.95 | 15.43 | 6.40 | 11.92 | negative | 14 |  |  |
| 3289 | male | 54 | positive | 89.83 | 10.17 | 8.80 | 8.83 | negative | 15 |  |  |
| 3290 | male | 55 | negative | 89.68 | 3.95 | 1.56 | 22.70 | negative | 12 |  |  |
| 3291 | male | 55 | negative | 83.78 | 3.26 | 1.92 | 25.70 | negative | 12 |  |  |
| 3292 | female | 54 | negative | 31.67 | 14.97 | 50.39 | 2.12 | negative | 13 |  |  |
| 3293 | male | 55 | negative | 74.00 | 10.60 | 1.70 | 6.98 | negative | 12 |  |  |
| 3294 | male | 55 | negative | 94.00 | 15.74 | 11.37 | 5.97 | negative | 14 |  |  |
| 3295 | male | 54 | negative | 140.20 | 9.55 | 7.15 | 14.68 | negative | 14 |  |  |
| 3296 | male | 54 | positive | 106.56 | 12.51 | 5.61 | 8.52 | negative | 13 |  |  |
| 3297 | male | 54 | positive | 154.19 | 20.60 | 5.88 | 7.48 | negative | 15 |  |  |
| 3298 | male | 54 | positive | 64.38 | 20.99 | 53.78 | 3.07 | negative | 18 |  |  |
| 3299 | male | 54 | positive | 117.66 | 13.65 | 7.22 | 8.62 | negative | 15 |  |  |
| 3300 | male | 55 | positive | 225.35 | 20.72 | 10.56 | 10.88 | negative | 15 |  |  |
| 3301 | male | 55 | negative | 79.56 | 4.70 | 1.68 | 16.93 | negative | 12 |  |  |
| 3302 | male | 55 | positive | 114.27 | 18.29 | 13.46 | 6.25 | negative | 15 |  |  |
| 3303 | male | 55 | negative | 71.07 | 7.39 | 1.82 | 9.62 | negative | 12 |  |  |
| 3304 | male | 54 | positive | 107.94 | 8.38 | 6.23 | 12.88 | negative | 15 |  |  |
| 3305 | male | 55 | negative | 116.06 | 5.57 | 1.94 | 20.84 | negative | 12 |  |  |
| 3306 | male | 54 | negative | 23.21 | 7.30 | 60.00 | 3.18 | negative | 17 |  |  |
| 3307 | male | 55 | positive | 112.89 | 9.43 | 13.29 | 11.97 | negative | 15 |  |  |
| 3308 | male | 54 | negative | 145.85 | 31.36 | 9.73 | 4.65 | negative | 14 |  |  |
| 3309 | male | 54 | positive | 79.07 | 27.64 | 9.34 | 2.86 | negative | 18 |  |  |
| 3310 | male | 54 | negative | 142.90 | 18.10 | 8.30 | 7.90 | negative | 14 |  |  |
| 3311 | male | 54 | positive | 90.43 | 6.93 | 5.31 | 13.05 | negative | 13 |  |  |
| 3312 | male | 54 | negative | 153.59 | 20.82 | 5.22 | 7.38 | negative | 12 |  |  |
| 3313 | male | 54 | positive | 158.23 | 17.76 | 6.29 | 8.91 | negative | 15 |  |  |
| 3314 | male | 54 | negative | 299.98 | 21.93 | 8.01 | 13.68 | negative | 14 |  |  |
| 3315 | male | 54 | positive | 58.81 | 8.46 | 7.00 | 6.95 | negative | 15 |  |  |
| 3316 | male | 54 | positive | 130.24 | 10.78 | 6.97 | 12.08 | negative | 15 |  |  |
| 3317 | male | 54 | negative | 150.06 | 18.19 | 7.21 | 8.25 | negative | 14 |  |  |
| 3318 | male | 54 | positive | 72.29 | 10.53 | 7.61 | 6.87 | negative | 15 |  |  |
| 3319 | male | 55 | negative | 95.94 | 6.25 | 1.74 | 15.35 | negative | 12 |  |  |
| 3320 | male | 54 | positive | 187.80 | 14.00 | 6.00 | 13.41 | negative | 15 |  |  |
| 3321 | male | 54 | negative | 157.54 | 11.26 | 8.73 | 13.99 | negative | 14 |  |  |
| 3322 | male | 54 | positive | 110.69 | 13.44 | 8.53 | 8.24 | negative | 15 |  |  |
| 3323 | male | 55 | negative | 88.36 | 19.47 | 12.45 | 4.54 | negative | 14 |  |  |
| 3324 | male | 54 | positive | 210.62 | 17.88 | 8.98 | 11.78 | negative | 15 |  |  |
| 3325 | male | 54 | positive | 120.11 | 13.17 | 7.70 | 9.12 | negative | 15 |  |  |
| 3326 | male | 54 | negative | 92.23 | 16.77 | 5.42 | 5.50 | negative | 12 |  |  |
| 3327 | male | 54 | positive | 263.29 | 33.51 | 7.39 | 7.86 | negative | 15 |  |  |
| 3328 | male | 54 | negative | 89.16 | 5.45 | 7.52 | 16.36 | negative | 14 |  |  |
| 3329 | male | 55 | positive | 243.56 | 32.70 | 18.50 | 7.45 | negative | 15 |  |  |
| 3330 | male | 55 | negative | 70.04 | 4.63 | 1.62 | 15.13 | negative | 12 |  |  |
| 3331 | male | 54 | positive | 145.70 | 33.60 | 8.50 | 4.34 | negative | 15 |  |  |
| 3332 | male | 55 | negative | 74.90 | 4.49 | 1.73 | 16.68 | negative | 12 |  |  |
| 3333 | male | 55 | negative | 181.17 | 16.54 | 11.18 | 10.95 | negative | 14 |  |  |
| 3334 | male | 54 | negative | 108.23 | 8.38 | 5.65 | 12.92 | negative | 12 |  |  |
| 3335 | male | 54 | negative | 114.57 | 13.72 | 6.28 | 8.35 | negative | 14 |  |  |
| 3336 | male | 54 | positive | 133.02 | 12.71 | 8.44 | 10.47 | negative | 15 |  |  |
| 3337 | male | 54 | positive | 169.06 | 11.60 | 7.91 | 14.57 | negative | 15 |  |  |
| 3338 | male | 54 | negative | 69.70 | 7.20 | 6.20 | 9.68 | negative | 14 |  |  |
| 3339 | female | 54 | negative | 19.86 | 8.87 | 58.64 | 2.24 | negative | 13 |  |  |
| 3340 | male | 54 | negative | 545.27 | 16.30 | 78.31 | 33.45 | negative | 14 |  |  |
| 3341 | male | 55 | negative | 140.50 | 23.60 | 17.30 | 5.95 | negative | 14 |  |  |
| 3342 | male | 54 | negative | 125.46 | 12.33 | 9.95 | 10.18 | negative | 14 |  |  |
| 3343 | male | 55 | negative | 105.28 | 5.83 | 1.84 | 18.06 | negative | 12 |  |  |
| 3344 | male | 54 | negative | 162.03 | 13.36 | 6.22 | 12.13 | negative | 14 |  |  |
| 3345 | male | 54 | negative | 70.90 | 5.80 | 6.20 | 12.22 | negative | 14 |  |  |
| 3346 | male | 54 | negative | 152.10 | 8.21 | 6.94 | 18.53 | negative | 14 |  |  |
| 3347 | male | 54 | positive | 80.12 | 8.82 | 8.28 | 9.08 | negative | 15 |  |  |
| 3348 | male | 55 | negative | 58.11 | 3.82 | 1.79 | 15.21 | negative | 12 |  |  |
| 3349 | male | 54 | positive | 155.45 | 24.43 | 3.92 | 6.36 | negative | 13 |  |  |
| 3350 | male | 54 | negative | 111.23 | 6.29 | 2.67 | 17.68 | negative | 12 |  |  |
| 3351 | male | 54 | positive | 113.03 | 9.14 | 3.61 | 12.37 | negative | 13 |  |  |
| 3352 | male | 54 | positive | 91.03 | 12.24 | 4.87 | 7.44 | negative | 13 |  |  |
| 3353 | male | 54 | negative | 72.14 | 6.23 | 5.08 | 11.58 | negative | 12 |  |  |
| 3354 | male | 54 | negative | 77.30 | 7.60 | 4.48 | 10.17 | negative | 12 |  |  |
| 3355 | male | 54 | positive | 68.50 | 8.90 | 4.00 | 7.70 | negative | 13 |  |  |
| 3356 | male | 54 | negative | 116.31 | 10.17 | 4.20 | 11.44 | negative | 12 |  |  |
| 3357 | male | 54 | negative | 152.17 | 9.53 | 4.49 | 15.97 | negative | 12 |  |  |
| 3358 | male | 54 | positive | 85.22 | 17.63 | 42.05 | 4.83 | negative | 15 |  |  |
| 3359 | male | 54 | negative | 139.00 | 15.11 | 5.02 | 9.20 | negative | 12 |  |  |
| 3360 | male | 54 | negative | 156.31 | 9.89 | 25.44 | 15.80 | negative | 14 |  |  |
| 3361 | male | 54 | negative | 160.24 | 11.57 | 4.40 | 13.85 | negative | 12 |  |  |
| 3362 | male | 54 | negative | 121.54 | 7.80 | 3.90 | 15.58 | negative | 12 |  |  |
| 3363 | male | 54 | negative | 63.42 | 4.08 | 3.22 | 15.54 | negative | 12 |  |  |
| 3364 | male | 54 | negative | 130.50 | 6.20 | 3.20 | 21.05 | negative | 12 |  |  |
| 3365 | male | 54 | negative | 141.86 | 8.80 | 3.95 | 16.12 | negative | 12 |  |  |
| 3366 | male | 54 | positive | 68.69 | 10.81 | 4.92 | 6.35 | negative | 13 |  |  |
| 3367 | male | 54 | negative | 224.70 | 16.07 | 4.96 | 13.98 | negative | 12 |  |  |
| 3368 | male | 54 | negative | 75.20 | 6.74 | 2.67 | 11.16 | negative | 12 |  |  |
| 3369 | male | 54 | negative | 114.65 | 7.40 | 4.15 | 15.49 | negative | 12 |  |  |
| 3370 | male | 54 | negative | 205.51 | 9.47 | 4.98 | 21.70 | negative | 12 |  |  |
| 3371 | male | 54 | negative | 111.53 | 7.53 | 3.30 | 14.81 | negative | 12 |  |  |
| 3372 | male | 54 | positive | 152.44 | 15.29 | 4.43 | 9.97 | negative | 13 |  |  |
| 3373 | male | 54 | negative | 68.40 | 5.48 | 3.88 | 12.48 | negative | 12 |  |  |
| 3374 | male | 54 | negative | 73.20 | 6.90 | 2.80 | 10.61 | negative | 12 |  |  |
| 3375 | male | 54 | positive | 74.12 | 21.21 | 4.31 | 3.49 | negative | 16 |  |  |
| 3376 | male | 54 | negative | 74.61 | 5.90 | 3.29 | 12.65 | negative | 12 |  |  |
| 3377 | male | 54 | negative | 300.00 | 22.46 | 37.24 | 13.36 | negative | 14 |  |  |
| 3378 | male | 54 | negative | 101.38 | 8.28 | 2.90 | 12.24 | negative | 12 |  |  |
| 3379 | male | 54 | negative | 148.34 | 12.31 | 3.56 | 12.05 | negative | 12 |  |  |
| 3380 | male | 54 | positive | 181.23 | 12.06 | 4.55 | 15.03 | negative | 13 |  |  |
| 3381 | male | 54 | positive | 242.40 | 17.16 | 2.99 | 14.13 | negative | 13 |  |  |
| 3382 | male | 54 | negative | 30.35 | 2.88 | 4.28 | 10.54 | negative | 12 |  |  |
| 3383 | male | 54 | negative | 143.68 | 15.22 | 3.59 | 9.44 | negative | 12 |  |  |
| 3384 | male | 54 | positive | 264.15 | 13.15 | 3.58 | 20.09 | negative | 13 |  |  |
| 3385 | male | 54 | negative | 105.55 | 8.19 | 3.88 | 12.89 | negative | 12 |  |  |
| 3386 | male | 54 | positive | 70.93 | 6.40 | 4.08 | 11.08 | negative | 13 |  |  |
| 3387 | male | 54 | negative | 74.38 | 3.19 | 2.96 | 23.32 | negative | 12 |  |  |
| 3388 | male | 54 | negative | 43.69 | 7.56 | 3.12 | 5.78 | negative | 12 |  |  |
| 3389 | male | 54 | negative | 121.08 | 11.66 | 3.27 | 10.38 | negative | 12 |  |  |
| 3390 | male | 54 | negative | 71.64 | 4.10 | 3.21 | 17.47 | negative | 12 |  |  |
| 3391 | male | 54 | negative | 121.84 | 6.77 | 2.76 | 18.00 | negative | 12 |  |  |
| 3392 | male | 54 | negative | 64.75 | 7.14 | 4.75 | 9.07 | negative | 12 |  |  |
| 3393 | male | 54 | negative | 130.23 | 11.54 | 3.63 | 11.29 | negative | 12 |  |  |
| 3394 | male | 54 | negative | 36.74 | 18.95 | 34.40 | 1.94 | negative | 17 |  |  |
| 3395 | male | 54 | negative | 121.68 | 6.14 | 3.50 | 19.82 | negative | 12 |  |  |
| 3396 | male | 54 | positive | 70.23 | 8.83 | 4.27 | 7.95 | negative | 13 |  |  |
| 3397 | male | 54 | positive | 116.97 | 9.34 | 4.51 | 12.52 | negative | 13 |  |  |
| 3398 | male | 54 | negative | 164.12 | 7.54 | 2.83 | 21.77 | negative | 12 |  |  |
| 3399 | male | 54 | negative | 185.60 | 18.19 | 3.91 | 10.20 | negative | 12 |  |  |
| 3400 | male | 54 | positive | 148.69 | 31.20 | 27.65 | 4.77 | negative | 15 |  |  |
| 3401 | male | 54 | negative | 75.88 | 6.71 | 2.98 | 11.31 | negative | 12 |  |  |
| 3402 | male | 54 | negative | 68.80 | 7.10 | 3.20 | 9.69 | negative | 12 |  |  |
| 3403 | male | 54 | positive | 146.78 | 13.66 | 4.46 | 10.75 | negative | 13 |  |  |
| 3404 | male | 54 | positive | 116.54 | 11.71 | 4.22 | 9.95 | negative | 13 |  |  |
| 3405 | male | 54 | negative | 110.86 | 8.91 | 3.70 | 12.44 | negative | 12 |  |  |
| 3406 | male | 54 | positive | 206.43 | 21.66 | 22.39 | 9.53 | negative | 15 |  |  |
| 3407 | male | 54 | negative | 87.98 | 6.60 | 3.05 | 13.33 | negative | 12 |  |  |
| 3408 | male | 54 | negative | 76.53 | 5.66 | 4.83 | 13.52 | negative | 12 |  |  |
| 3409 | male | 54 | negative | 139.80 | 26.39 | 2.96 | 5.30 | negative | 12 |  |  |
| 3410 | female | 54 | positive | 50.40 | 13.90 | 3.80 | 3.63 | negative | 12 |  |  |
| 3411 | male | 54 | positive | 143.35 | 15.51 | 3.22 | 9.24 | negative | 13 |  |  |
| 3412 | male | 54 | negative | 138.33 | 10.94 | 3.66 | 12.64 | negative | 12 |  |  |
| 3413 | male | 54 | positive | 67.82 | 9.98 | 4.77 | 6.80 | negative | 13 |  |  |
| 3414 | male | 54 | positive | 292.06 | 21.28 | 4.05 | 13.72 | negative | 13 |  |  |
| 3415 | male | 54 | positive | 72.80 | 10.10 | 3.20 | 7.21 | negative | 13 |  |  |
| 3416 | male | 54 | positive | 130.30 | 23.90 | 4.40 | 5.45 | negative | 13 |  |  |
| 3417 | male | 54 | positive | 166.83 | 13.05 | 3.33 | 12.78 | negative | 13 |  |  |
| 3418 | male | 54 | negative | 70.11 | 9.55 | 3.85 | 7.34 | negative | 12 |  |  |
| 3419 | male | 54 | negative | 95.97 | 7.20 | 3.60 | 13.33 | negative | 12 |  |  |
| 3420 | male | 54 | positive | 87.65 | 18.74 | 37.88 | 4.68 | negative | 15 |  |  |
| 3421 | male | 54 | negative | 84.71 | 7.06 | 2.93 | 12.00 | negative | 12 |  |  |
| 3422 | male | 54 | negative | 44.32 | 4.51 | 4.31 | 9.83 | negative | 12 |  |  |
| 3423 | male | 54 | negative | 84.47 | 11.73 | 4.20 | 7.20 | negative | 12 |  |  |
| 3424 | male | 54 | positive | 119.07 | 14.36 | 2.97 | 8.29 | negative | 13 |  |  |
| 3425 | male | 54 | negative | 157.28 | 13.07 | 5.19 | 12.03 | negative | 12 |  |  |
| 3426 | male | 54 | positive | 93.40 | 4.42 | 4.24 | 21.13 | negative | 13 |  |  |
| 3427 | male | 54 | negative | 57.64 | 16.01 | 25.05 | 3.60 | negative | 17 |  |  |
| 3428 | male | 54 | negative | 277.61 | 21.95 | 41.30 | 12.65 | negative | 14 |  |  |
| 3429 | male | 54 | positive | 129.70 | 13.78 | 43.55 | 9.41 | negative | 15 |  |  |
| 3430 | male | 54 | positive | 148.45 | 8.94 | 4.14 | 16.61 | negative | 13 |  |  |
| 3431 | male | 54 | negative | 142.86 | 5.77 | 3.99 | 24.76 | negative | 12 |  |  |
| 3432 | male | 54 | positive | 93.33 | 17.05 | 4.11 | 5.47 | negative | 13 |  |  |
| 3433 | male | 54 | negative | 52.47 | 3.01 | 3.20 | 17.43 | negative | 12 |  |  |
| 3434 | male | 54 | negative | 136.78 | 8.70 | 3.21 | 15.72 | negative | 12 |  |  |
| 3435 | male | 54 | positive | 224.22 | 22.79 | 4.43 | 9.84 | negative | 13 |  |  |
| 3436 | male | 54 | negative | 146.77 | 15.35 | 2.92 | 9.56 | negative | 12 |  |  |
| 3437 | male | 54 | negative | 150.67 | 8.90 | 2.77 | 16.93 | negative | 12 |  |  |
| 3438 | male | 54 | negative | 104.89 | 8.94 | 3.43 | 11.73 | negative | 12 |  |  |
| 3439 | male | 54 | negative | 59.65 | 5.08 | 2.74 | 11.74 | negative | 12 |  |  |
| 3440 | male | 54 | negative | 67.06 | 4.80 | 3.10 | 13.97 | negative | 12 |  |  |
| 3441 | male | 54 | negative | 109.63 | 8.70 | 3.23 | 12.60 | negative | 12 |  |  |
| 3442 | male | 54 | positive | 91.70 | 10.07 | 3.49 | 9.11 | negative | 13 |  |  |
| 3443 | male | 54 | negative | 127.75 | 9.58 | 2.82 | 13.34 | negative | 12 |  |  |
| 3444 | male | 54 | negative | 86.71 | 4.41 | 2.80 | 19.66 | negative | 12 |  |  |
| 3445 | male | 54 | negative | 87.94 | 8.49 | 4.80 | 10.36 | negative | 12 |  |  |
| 3446 | male | 54 | negative | 70.57 | 8.10 | 3.41 | 8.71 | negative | 12 |  |  |
| 3447 | male | 54 | negative | 133.60 | 5.75 | 4.63 | 23.23 | negative | 12 |  |  |
| 3448 | male | 54 | negative | 108.14 | 6.87 | 3.27 | 15.74 | negative | 12 |  |  |
| 3449 | male | 54 | negative | 72.10 | 5.58 | 3.29 | 12.92 | negative | 12 |  |  |
| 3450 | male | 54 | negative | 148.40 | 10.53 | 4.39 | 14.09 | negative | 12 |  |  |
| 3451 | male | 54 | positive | 89.22 | 15.74 | 3.04 | 5.67 | negative | 13 |  |  |
| 3452 | male | 54 | positive | 129.83 | 14.92 | 4.78 | 8.70 | negative | 13 |  |  |
| 3453 | male | 54 | negative | 113.59 | 15.60 | 2.76 | 7.28 | negative | 12 |  |  |
| 3454 | male | 54 | positive | 107.00 | 8.64 | 4.79 | 12.38 | negative | 13 |  |  |
| 3455 | male | 54 | negative | 76.85 | 7.12 | 3.11 | 10.79 | negative | 12 |  |  |
| 3456 | male | 54 | negative | 111.06 | 6.65 | 2.83 | 16.70 | negative | 12 |  |  |
| 3457 | male | 54 | negative | 89.46 | 11.92 | 4.08 | 7.51 | negative | 12 |  |  |
| 3458 | male | 54 | negative | 143.78 | 9.39 | 3.03 | 15.31 | negative | 12 |  |  |
| 3459 | male | 54 | negative | 70.70 | 9.60 | 3.40 | 7.36 | negative | 12 |  |  |
| 3460 | male | 54 | negative | 138.61 | 9.92 | 2.68 | 13.97 | negative | 12 |  |  |
| 3461 | male | 54 | negative | 51.49 | 18.27 | 39.51 | 2.82 | negative | 17 |  |  |
| 3462 | male | 54 | negative | 103.84 | 7.77 | 4.68 | 13.36 | negative | 12 |  |  |
| 3463 | male | 54 | negative | 117.83 | 6.52 | 4.12 | 18.07 | negative | 12 |  |  |
| 3464 | male | 54 | positive | 117.70 | 15.89 | 4.86 | 7.41 | negative | 13 |  |  |
| 3465 | male | 54 | negative | 156.33 | 14.38 | 2.71 | 10.87 | negative | 12 |  |  |
| 3466 | male | 54 | negative | 136.93 | 8.97 | 3.07 | 15.27 | negative | 12 |  |  |
| 3467 | male | 54 | negative | 89.21 | 7.39 | 3.00 | 12.07 | negative | 12 |  |  |
| 3468 | male | 54 | negative | 57.70 | 5.23 | 2.92 | 11.03 | negative | 12 |  |  |
| 3469 | male | 54 | negative | 47.35 | 8.46 | 45.02 | 5.60 | negative | 14 |  |  |
| 3470 | male | 54 | negative | 10.30 | 2.18 | 44.55 | 4.72 | negative | 14 |  |  |
| 3471 | male | 54 | negative | 83.03 | 4.56 | 4.56 | 18.21 | negative | 12 |  |  |
| 3472 | male | 54 | negative | 88.72 | 25.09 | 23.20 | 3.54 | negative | 17 |  |  |
| 3473 | male | 54 | negative | 118.19 | 18.72 | 4.11 | 6.31 | negative | 12 |  |  |
| 3474 | male | 54 | positive | 193.20 | 15.12 | 4.81 | 12.78 | negative | 13 |  |  |
| 3475 | male | 54 | negative | 127.07 | 8.47 | 3.43 | 15.00 | negative | 12 |  |  |
| 3476 | male | 54 | negative | 84.10 | 8.20 | 4.20 | 10.26 | negative | 12 |  |  |
| 3477 | male | 54 | negative | 57.37 | 5.73 | 5.18 | 10.01 | negative | 12 |  |  |
| 3478 | male | 54 | negative | 99.48 | 12.56 | 3.56 | 7.92 | negative | 12 |  |  |
| 3479 | male | 54 | negative | 26.77 | 12.58 | 24.30 | 2.13 | negative | 17 |  |  |
| 3480 | male | 54 | positive | 77.57 | 8.32 | 3.03 | 9.32 | negative | 13 |  |  |
| 3481 | male | 54 | negative | 138.22 | 11.00 | 3.39 | 12.57 | negative | 12 |  |  |
| 3482 | male | 54 | negative | 205.20 | 16.05 | 4.92 | 12.79 | negative | 12 |  |  |
| 3483 | male | 54 | positive | 141.83 | 15.79 | 4.29 | 8.98 | negative | 13 |  |  |
| 3484 | male | 54 | negative | 38.88 | 7.62 | 22.42 | 5.10 | negative | 14 |  |  |
| 3485 | male | 54 | positive | 184.36 | 13.20 | 3.13 | 13.97 | negative | 13 |  |  |
| 3486 | male | 54 | negative | 186.59 | 10.04 | 2.92 | 18.58 | negative | 12 |  |  |
| 3487 | male | 54 | negative | 74.00 | 5.60 | 3.50 | 13.21 | negative | 12 |  |  |
| 3488 | male | 54 | negative | 270.46 | 30.27 | 31.98 | 8.93 | negative | 14 |  |  |
| 3489 | male | 54 | positive | 250.20 | 19.23 | 3.70 | 13.01 | negative | 13 |  |  |
| 3490 | male | 54 | negative | 66.70 | 6.57 | 4.36 | 10.15 | negative | 12 |  |  |
| 3491 | male | 54 | negative | 56.21 | 3.20 | 1.78 | 17.57 | negative | 12 |  |  |
| 3492 | male | 54 | negative | 115.69 | 7.60 | 2.20 | 15.22 | negative | 12 |  |  |
| 3493 | male | 54 | negative | 105.40 | 6.92 | 2.22 | 15.23 | negative | 12 |  |  |
| 3494 | male | 54 | negative | 133.47 | 7.01 | 2.13 | 19.04 | negative | 12 |  |  |
| 3495 | male | 54 | negative | 81.50 | 5.17 | 2.16 | 15.76 | negative | 12 |  |  |
| 3496 | male | 54 | negative | 122.49 | 8.90 | 2.50 | 13.76 | negative | 12 |  |  |
| 3497 | male | 54 | positive | 113.65 | 25.71 | 13.13 | 4.42 | negative | 15 |  |  |
| 3498 | male | 54 | negative | 86.19 | 8.10 | 2.33 | 10.64 | negative | 12 |  |  |
| 3499 | female | 54 | negative | 21.66 | 8.44 | 11.73 | 2.57 | negative | 13 |  |  |
| 3500 | male | 54 | negative | 77.59 | 6.09 | 2.07 | 12.74 | negative | 12 |  |  |
| 3501 | male | 54 | negative | 278.60 | 9.91 | 17.93 | 28.11 | negative | 14 |  |  |
| 3502 | male | 54 | negative | 83.71 | 4.03 | 2.13 | 20.77 | negative | 12 |  |  |
| 3503 | male | 54 | positive | 287.32 | 24.54 | 10.28 | 11.71 | negative | 15 |  |  |
| 3504 | male | 54 | negative | 102.61 | 6.19 | 2.55 | 16.58 | negative | 12 |  |  |
| 3505 | male | 54 | negative | 137.12 | 11.22 | 2.19 | 12.22 | negative | 12 |  |  |
| 3506 | male | 54 | negative | 82.10 | 5.21 | 15.02 | 15.76 | negative | 14 |  |  |
| 3507 | male | 54 | negative | 138.51 | 14.97 | 1.99 | 9.25 | negative | 12 |  |  |
| 3508 | male | 54 | negative | 129.77 | 6.56 | 1.76 | 19.78 | negative | 12 |  |  |
| 3509 | male | 54 | positive | 108.12 | 19.03 | 12.57 | 5.68 | negative | 15 |  |  |
| 3510 | male | 54 | negative | 137.31 | 10.74 | 2.56 | 12.78 | negative | 12 |  |  |
| 3511 | male | 54 | negative | 140.74 | 11.04 | 2.10 | 12.75 | negative | 12 |  |  |
| 3512 | male | 54 | positive | 201.83 | 17.91 | 13.54 | 11.27 | negative | 15 |  |  |
| 3513 | male | 54 | negative | 169.27 | 10.48 | 2.36 | 16.15 | negative | 12 |  |  |
| 3514 | female | 54 | positive | 29.66 | 8.05 | 2.60 | 3.68 | negative | 12 |  |  |
| 3515 | male | 54 | negative | 113.10 | 6.71 | 2.41 | 16.86 | negative | 12 |  |  |
| 3516 | male | 54 | positive | 100.45 | 18.79 | 10.42 | 5.35 | negative | 15 |  |  |
| 3517 | male | 54 | negative | 71.92 | 6.25 | 2.30 | 11.51 | negative | 12 |  |  |
| 3518 | male | 54 | positive | 120.64 | 11.38 | 2.58 | 10.60 | negative | 13 |  |  |
| 3519 | male | 54 | positive | 165.64 | 16.04 | 11.44 | 10.33 | negative | 15 |  |  |
| 3520 | male | 54 | positive | 181.03 | 20.86 | 13.09 | 8.68 | negative | 15 |  |  |
| 3521 | male | 54 | positive | 90.81 | 6.08 | 2.43 | 14.94 | negative | 13 |  |  |
| 3522 | male | 54 | negative | 148.09 | 8.67 | 1.86 | 17.08 | negative | 12 |  |  |
| 3523 | male | 54 | positive | 114.90 | 13.80 | 1.80 | 8.33 | negative | 13 |  |  |
| 3524 | male | 54 | negative | 98.05 | 13.60 | 2.48 | 7.21 | negative | 12 |  |  |
| 3525 | male | 54 | positive | 178.88 | 15.74 | 13.56 | 11.36 | negative | 15 |  |  |
| 3526 | male | 54 | negative | 67.44 | 5.24 | 2.29 | 12.87 | negative | 12 |  |  |
| 3527 | male | 54 | negative | 149.96 | 8.93 | 2.24 | 16.79 | negative | 12 |  |  |
| 3528 | male | 54 | negative | 65.33 | 5.43 | 1.98 | 12.03 | negative | 12 |  |  |
| 3529 | male | 54 | negative | 90.55 | 5.42 | 2.38 | 16.71 | negative | 12 |  |  |
| 3530 | male | 54 | positive | 184.92 | 13.74 | 10.81 | 13.46 | negative | 15 |  |  |
| 3531 | male | 54 | negative | 86.48 | 8.49 | 14.48 | 10.19 | negative | 14 |  |  |
| 3532 | male | 54 | positive | 237.94 | 17.25 | 17.97 | 13.79 | negative | 15 |  |  |
| 3533 | male | 54 | negative | 55.86 | 4.75 | 2.59 | 11.76 | negative | 12 |  |  |
| 3534 | male | 54 | positive | 214.87 | 21.78 | 13.00 | 9.87 | negative | 15 |  |  |
| 3535 | male | 54 | negative | 173.50 | 9.81 | 1.80 | 17.69 | negative | 12 |  |  |
| 3536 | male | 54 | positive | 193.40 | 14.02 | 1.88 | 13.79 | negative | 13 |  |  |
| 3537 | male | 54 | negative | 99.72 | 7.04 | 1.77 | 14.16 | negative | 12 |  |  |
| 3538 | male | 54 | negative | 138.75 | 10.27 | 2.06 | 13.51 | negative | 12 |  |  |
| 3539 | male | 54 | negative | 15.76 | 5.69 | 16.00 | 2.77 | negative | 17 |  |  |
| 3540 | male | 54 | negative | 184.29 | 14.58 | 2.57 | 12.64 | negative | 12 |  |  |
| 3541 | male | 54 | negative | 66.02 | 4.71 | 1.99 | 14.02 | negative | 12 |  |  |
| 3542 | male | 54 | negative | 161.15 | 13.29 | 1.79 | 12.13 | negative | 12 |  |  |
| 3543 | male | 54 | positive | 300.00 | 21.73 | 2.58 | 13.81 | negative | 13 |  |  |
| 3544 | male | 54 | negative | 55.67 | 3.77 | 1.99 | 14.77 | negative | 12 |  |  |
| 3545 | male | 54 | negative | 198.19 | 14.47 | 2.52 | 13.70 | negative | 12 |  |  |
| 3546 | male | 54 | positive | 234.88 | 21.99 | 12.63 | 10.68 | negative | 15 |  |  |
| 3547 | male | 54 | negative | 118.47 | 9.15 | 2.03 | 12.95 | negative | 12 |  |  |
| 3548 | male | 54 | negative | 90.53 | 5.34 | 2.12 | 16.95 | negative | 12 |  |  |
| 3549 | male | 54 | negative | 99.55 | 6.34 | 2.40 | 15.70 | negative | 12 |  |  |
| 3550 | male | 54 | positive | 118.22 | 11.87 | 10.89 | 9.96 | negative | 15 |  |  |
| 3551 | male | 54 | negative | 145.73 | 6.00 | 1.88 | 24.29 | negative | 12 |  |  |
| 3552 | male | 54 | negative | 95.17 | 4.61 | 2.10 | 20.64 | negative | 12 |  |  |
| 3553 | male | 54 | negative | 123.32 | 13.23 | 2.48 | 9.32 | negative | 12 |  |  |
| 3554 | male | 54 | positive | 252.22 | 13.93 | 18.82 | 18.11 | negative | 15 |  |  |
| 3555 | male | 54 | negative | 104.97 | 5.88 | 2.50 | 17.85 | negative | 12 |  |  |
| 3556 | male | 54 | positive | 94.08 | 28.70 | 10.19 | 3.28 | negative | 18 |  |  |
| 3557 | female | 54 | positive | 125.39 | 36.00 | 11.40 | 3.48 | negative | 14 |  |  |
| 3558 | male | 54 | negative | 48.55 | 3.58 | 2.60 | 13.56 | negative | 12 |  |  |
| 3559 | male | 54 | positive | 60.85 | 30.24 | 14.46 | 2.01 | negative | 18 |  |  |
| 3560 | male | 54 | positive | 47.73 | 11.26 | 11.01 | 4.24 | negative | 15 |  |  |
| 3561 | male | 54 | negative | 162.34 | 6.50 | 2.15 | 24.98 | negative | 12 |  |  |
| 3562 | male | 54 | positive | 54.20 | 10.30 | 1.90 | 5.26 | negative | 13 |  |  |
| 3563 | male | 54 | negative | 165.18 | 18.86 | 12.91 | 8.76 | negative | 14 |  |  |
| 3564 | male | 54 | negative | 297.94 | 17.07 | 1.94 | 17.45 | negative | 12 |  |  |
| 3565 | male | 54 | positive | 90.70 | 11.81 | 13.15 | 7.68 | negative | 15 |  |  |
| 3566 | male | 54 | positive | 186.98 | 23.70 | 17.01 | 7.89 | negative | 15 |  |  |
| 3567 | male | 54 | positive | 290.05 | 26.23 | 14.54 | 11.06 | negative | 15 |  |  |
| 3568 | male | 54 | negative | 96.94 | 8.18 | 1.93 | 11.85 | negative | 12 |  |  |
| 3569 | male | 54 | negative | 125.97 | 5.48 | 2.58 | 22.99 | negative | 12 |  |  |
| 3570 | male | 54 | negative | 104.19 | 7.36 | 2.08 | 14.16 | negative | 12 |  |  |
| 3571 | male | 54 | negative | 154.37 | 13.09 | 2.11 | 11.79 | negative | 12 |  |  |
| 3572 | male | 54 | positive | 192.43 | 16.04 | 11.70 | 12.00 | negative | 15 |  |  |
| 3573 | male | 54 | positive | 206.04 | 34.49 | 14.90 | 5.97 | negative | 15 |  |  |
| 3574 | male | 54 | negative | 162.92 | 10.06 | 2.16 | 16.19 | negative | 12 |  |  |
| 3575 | male | 54 | positive | 120.76 | 10.66 | 11.14 | 11.33 | negative | 15 |  |  |
| 3576 | male | 54 | positive | 107.21 | 18.73 | 11.47 | 5.72 | negative | 15 |  |  |
| 3577 | male | 54 | negative | 123.27 | 13.37 | 2.07 | 9.22 | negative | 12 |  |  |
| 3578 | male | 54 | negative | 38.29 | 7.03 | 2.20 | 5.45 | negative | 12 |  |  |
| 3579 | male | 54 | negative | 241.03 | 15.47 | 12.92 | 15.58 | negative | 14 |  |  |
| 3580 | male | 54 | negative | 51.31 | 7.16 | 1.88 | 7.17 | negative | 12 |  |  |
| 3581 | female | 54 | positive | 87.98 | 25.81 | 14.34 | 3.41 | negative | 14 |  |  |
| 3582 | male | 54 | negative | 171.68 | 12.69 | 10.35 | 13.53 | negative | 14 |  |  |
| 3583 | male | 54 | negative | 64.76 | 6.95 | 2.58 | 9.32 | negative | 12 |  |  |
| 3584 | male | 54 | negative | 89.78 | 8.09 | 1.77 | 11.10 | negative | 12 |  |  |
| 3585 | male | 54 | positive | 292.35 | 25.37 | 19.25 | 11.52 | negative | 15 |  |  |
| 3586 | male | 54 | negative | 47.07 | 6.00 | 2.53 | 7.84 | negative | 12 |  |  |
| 3587 | male | 54 | negative | 146.24 | 12.56 | 2.23 | 11.64 | negative | 12 |  |  |
| 3588 | male | 54 | positive | 196.58 | 25.63 | 19.14 | 7.67 | negative | 15 |  |  |
| 3589 | male | 54 | negative | 97.97 | 8.50 | 2.05 | 11.53 | negative | 12 |  |  |
| 3590 | male | 54 | positive | 101.71 | 9.65 | 2.50 | 10.54 | negative | 13 |  |  |
| 3591 | male | 54 | positive | 97.77 | 10.71 | 2.29 | 9.13 | negative | 13 |  |  |
| 3592 | male | 54 | negative | 256.50 | 13.78 | 2.56 | 18.61 | negative | 12 |  |  |
| 3593 | male | 54 | negative | 103.09 | 6.78 | 2.15 | 15.21 | negative | 12 |  |  |
| 3594 | male | 54 | negative | 104.45 | 5.04 | 2.53 | 20.72 | negative | 12 |  |  |
| 3595 | male | 54 | positive | 223.17 | 31.26 | 18.40 | 7.14 | negative | 15 |  |  |
| 3596 | male | 54 | negative | 165.40 | 14.60 | 2.60 | 11.33 | negative | 12 |  |  |
| 3597 | male | 54 | negative | 82.60 | 7.74 | 1.86 | 10.67 | negative | 12 |  |  |
| 3598 | male | 54 | positive | 208.17 | 19.77 | 14.86 | 10.53 | negative | 15 |  |  |
| 3599 | male | 54 | negative | 89.36 | 7.08 | 2.42 | 12.62 | negative | 12 |  |  |
| 3600 | male | 54 | negative | 65.27 | 3.71 | 2.17 | 17.59 | negative | 12 |  |  |
| 3601 | male | 54 | negative | 88.18 | 8.34 | 10.66 | 10.57 | negative | 14 |  |  |
| 3602 | male | 54 | positive | 90.30 | 8.10 | 2.40 | 11.15 | negative | 13 |  |  |
| 3603 | male | 54 | positive | 66.29 | 4.32 | 1.81 | 15.34 | negative | 13 |  |  |
| 3604 | male | 54 | negative | 165.08 | 11.59 | 1.80 | 14.24 | negative | 12 |  |  |
| 3605 | male | 54 | negative | 154.13 | 27.65 | 14.65 | 5.57 | negative | 14 |  |  |
| 3606 | male | 54 | positive | 100.60 | 9.46 | 2.20 | 10.63 | negative | 13 |  |  |
| 3607 | male | 54 | positive | 143.27 | 23.95 | 10.66 | 5.98 | negative | 15 |  |  |
| 3608 | male | 54 | negative | 105.23 | 9.99 | 2.37 | 10.53 | negative | 12 |  |  |
| 3609 | male | 54 | negative | 131.06 | 5.82 | 2.58 | 22.52 | negative | 12 |  |  |
| 3610 | male | 54 | positive | 101.96 | 8.57 | 10.54 | 11.90 | negative | 15 |  |  |
| 3611 | male | 54 | negative | 110.89 | 5.88 | 2.23 | 18.86 | negative | 12 |  |  |
| 3612 | male | 54 | negative | 152.97 | 7.37 | 1.75 | 20.76 | negative | 12 |  |  |
| 3613 | male | 54 | negative | 87.98 | 7.50 | 2.54 | 11.73 | negative | 12 |  |  |
| 3614 | male | 54 | negative | 92.06 | 4.77 | 2.56 | 19.30 | negative | 12 |  |  |
| 3615 | male | 54 | negative | 222.75 | 15.25 | 2.40 | 14.61 | negative | 12 |  |  |
| 3616 | male | 53 | positive | 157.53 | 14.75 | 4.74 | 10.68 | negative | 13 |  |  |
| 3617 | male | 53 | negative | 146.28 | 24.52 | 9.87 | 5.97 | negative | 14 |  |  |
| 3618 | male | 53 | positive | 119.32 | 10.42 | 4.48 | 11.45 | negative | 13 |  |  |
| 3619 | male | 54 | negative | 116.80 | 10.80 | 1.50 | 10.81 | negative | 12 |  |  |
| 3620 | male | 53 | positive | 99.41 | 8.59 | 6.93 | 11.57 | negative | 15 |  |  |
| 3621 | male | 53 | positive | 124.90 | 29.40 | 7.00 | 4.25 | negative | 15 |  |  |
| 3622 | male | 53 | negative | 71.20 | 9.80 | 7.60 | 7.27 | negative | 14 |  |  |
| 3623 | male | 53 | negative | 75.71 | 5.15 | 4.22 | 14.70 | negative | 12 |  |  |
| 3624 | male | 53 | negative | 101.57 | 30.05 | 6.43 | 3.38 | negative | 17 |  |  |
| 3625 | male | 53 | positive | 96.64 | 12.98 | 5.36 | 7.45 | negative | 13 |  |  |
| 3626 | male | 53 | positive | 127.00 | 22.93 | 7.58 | 5.54 | negative | 15 |  |  |
| 3627 | male | 53 | positive | 148.26 | 19.75 | 5.26 | 7.51 | negative | 13 |  |  |
| 3628 | male | 53 | positive | 127.84 | 13.57 | 8.80 | 9.42 | negative | 15 |  |  |
| 3629 | male | 54 | negative | 139.07 | 7.56 | 1.74 | 18.40 | negative | 12 |  |  |
| 3630 | male | 54 | negative | 82.60 | 4.67 | 1.50 | 17.69 | negative | 12 |  |  |
| 3631 | male | 53 | positive | 138.65 | 13.86 | 6.54 | 10.00 | negative | 15 |  |  |
| 3632 | male | 53 | negative | 132.59 | 16.34 | 8.71 | 8.11 | negative | 14 |  |  |
| 3633 | male | 54 | positive | 149.47 | 16.11 | 1.69 | 9.28 | negative | 13 |  |  |
| 3634 | male | 53 | positive | 90.76 | 9.94 | 4.90 | 9.13 | negative | 13 |  |  |
| 3635 | male | 53 | negative | 60.67 | 7.68 | 4.50 | 7.90 | negative | 12 |  |  |
| 3636 | male | 53 | negative | 110.41 | 8.41 | 4.84 | 13.13 | negative | 12 |  |  |
| 3637 | male | 54 | negative | 110.20 | 11.60 | 1.50 | 9.50 | negative | 12 |  |  |
| 3638 | male | 54 | negative | 99.15 | 5.10 | 1.65 | 19.44 | negative | 12 |  |  |
| 3639 | male | 53 | negative | 87.90 | 15.70 | 5.70 | 5.60 | negative | 12 |  |  |
| 3640 | male | 53 | positive | 127.53 | 19.20 | 4.10 | 6.64 | negative | 13 |  |  |
| 3641 | male | 54 | negative | 89.98 | 3.82 | 1.52 | 23.55 | negative | 12 |  |  |
| 3642 | male | 54 | negative | 73.70 | 6.20 | 1.50 | 11.89 | negative | 12 |  |  |
| 3643 | male | 53 | positive | 148.61 | 15.51 | 8.91 | 9.58 | negative | 15 |  |  |
| 3644 | male | 53 | negative | 101.74 | 10.00 | 8.33 | 10.17 | negative | 14 |  |  |
| 3645 | male | 53 | positive | 122.04 | 12.51 | 5.99 | 9.76 | negative | 15 |  |  |
| 3646 | male | 53 | positive | 157.80 | 22.10 | 5.10 | 7.14 | negative | 13 |  |  |
| 3647 | male | 53 | positive | 190.25 | 14.62 | 8.89 | 13.01 | negative | 15 |  |  |
| 3648 | male | 54 | negative | 99.54 | 7.39 | 1.57 | 13.47 | negative | 12 |  |  |
| 3649 | male | 53 | positive | 230.68 | 14.29 | 7.68 | 16.14 | negative | 15 |  |  |
| 3650 | male | 53 | positive | 140.21 | 15.05 | 4.35 | 9.32 | negative | 13 |  |  |
| 3651 | male | 53 | negative | 25.31 | 4.60 | 60.00 | 5.50 | negative | 14 |  |  |
| 3652 | female | 53 | positive | 42.37 | 24.27 | 7.64 | 1.75 | negative | 14 |  |  |
| 3653 | male | 53 | negative | 51.90 | 5.75 | 8.74 | 9.03 | negative | 14 |  |  |
| 3654 | male | 53 | negative | 179.26 | 15.00 | 8.40 | 11.95 | negative | 14 |  |  |
| 3655 | male | 53 | negative | 214.14 | 10.91 | 5.64 | 19.63 | negative | 12 |  |  |
| 3656 | male | 53 | negative | 178.99 | 28.30 | 8.44 | 6.32 | negative | 14 |  |  |
| 3657 | male | 54 | negative | 153.97 | 10.00 | 1.74 | 15.40 | negative | 12 |  |  |
| 3658 | male | 54 | negative | 157.34 | 11.20 | 1.72 | 14.05 | negative | 12 |  |  |
| 3659 | male | 53 | positive | 152.65 | 12.67 | 4.94 | 12.05 | negative | 13 |  |  |
| 3660 | male | 53 | positive | 110.50 | 14.89 | 9.38 | 7.42 | negative | 15 |  |  |
| 3661 | male | 53 | negative | 121.61 | 9.11 | 4.60 | 13.35 | negative | 12 |  |  |
| 3662 | female | 53 | positive | 22.70 | 7.75 | 44.60 | 2.93 | negative | 14 |  |  |
| 3663 | male | 53 | positive | 61.24 | 9.84 | 9.66 | 6.22 | negative | 15 |  |  |
| 3664 | male | 53 | positive | 212.24 | 22.99 | 5.99 | 9.23 | negative | 15 |  |  |
| 3665 | male | 53 | negative | 137.10 | 13.00 | 5.10 | 10.55 | negative | 12 |  |  |
| 3666 | male | 53 | negative | 161.95 | 12.64 | 7.38 | 12.81 | negative | 14 |  |  |
| 3667 | male | 53 | positive | 93.21 | 7.29 | 5.01 | 12.79 | negative | 13 |  |  |
| 3668 | male | 53 | negative | 152.69 | 8.72 | 5.68 | 17.51 | negative | 12 |  |  |
| 3669 | male | 54 | negative | 92.37 | 8.37 | 1.67 | 11.04 | negative | 12 |  |  |
| 3670 | male | 53 | positive | 114.47 | 13.31 | 4.82 | 8.60 | negative | 13 |  |  |
| 3671 | male | 53 | negative | 211.57 | 13.37 | 4.86 | 15.82 | negative | 12 |  |  |
| 3672 | male | 53 | positive | 102.39 | 6.32 | 4.05 | 16.20 | negative | 13 |  |  |
| 3673 | male | 53 | negative | 63.02 | 3.81 | 4.45 | 16.54 | negative | 12 |  |  |
| 3674 | male | 53 | positive | 153.38 | 16.22 | 9.20 | 9.46 | negative | 15 |  |  |
| 3675 | male | 53 | negative | 72.71 | 4.40 | 7.41 | 16.52 | negative | 14 |  |  |
| 3676 | male | 54 | positive | 124.12 | 9.84 | 1.72 | 12.61 | negative | 13 |  |  |
| 3677 | male | 53 | positive | 118.32 | 8.67 | 9.76 | 13.65 | negative | 15 |  |  |
| 3678 | male | 53 | positive | 289.55 | 22.60 | 9.81 | 12.81 | negative | 15 |  |  |
| 3679 | male | 53 | negative | 245.42 | 19.74 | 8.34 | 12.43 | negative | 14 |  |  |
| 3680 | male | 54 | negative | 106.68 | 6.10 | 1.62 | 17.49 | negative | 12 |  |  |
| 3681 | male | 53 | negative | 82.26 | 7.98 | 5.04 | 10.31 | negative | 12 |  |  |
| 3682 | male | 53 | negative | 95.97 | 9.16 | 4.12 | 10.48 | negative | 12 |  |  |
| 3683 | male | 54 | negative | 212.95 | 11.88 | 1.73 | 17.93 | negative | 12 |  |  |
| 3684 | male | 53 | positive | 86.41 | 14.51 | 4.38 | 5.96 | negative | 13 |  |  |
| 3685 | male | 53 | negative | 121.36 | 7.51 | 4.99 | 16.16 | negative | 12 |  |  |
| 3686 | male | 53 | positive | 66.65 | 21.06 | 7.20 | 3.16 | negative | 18 |  |  |
| 3687 | male | 53 | positive | 109.92 | 12.76 | 5.35 | 8.61 | negative | 13 |  |  |
| 3688 | male | 53 | negative | 91.87 | 6.41 | 5.80 | 14.33 | negative | 14 |  |  |
| 3689 | male | 53 | negative | 76.13 | 7.25 | 4.19 | 10.50 | negative | 12 |  |  |
| 3690 | male | 53 | negative | 125.12 | 10.78 | 4.80 | 11.61 | negative | 12 |  |  |
| 3691 | male | 54 | negative | 129.10 | 7.77 | 1.58 | 16.62 | negative | 12 |  |  |
| 3692 | male | 53 | positive | 147.22 | 13.73 | 9.23 | 10.72 | negative | 15 |  |  |
| 3693 | male | 53 | positive | 180.18 | 12.90 | 5.21 | 13.97 | negative | 13 |  |  |
| 3694 | male | 53 | negative | 177.11 | 14.36 | 4.33 | 12.33 | negative | 12 |  |  |
| 3695 | male | 53 | positive | 149.90 | 16.00 | 4.10 | 9.37 | negative | 13 |  |  |
| 3696 | male | 53 | positive | 198.01 | 19.68 | 4.10 | 10.06 | negative | 13 |  |  |
| 3697 | male | 54 | negative | 126.28 | 10.99 | 1.64 | 11.49 | negative | 12 |  |  |
| 3698 | male | 53 | negative | 135.37 | 14.41 | 5.20 | 9.39 | negative | 12 |  |  |
| 3699 | male | 54 | negative | 110.81 | 4.75 | 1.51 | 23.33 | negative | 12 |  |  |
| 3700 | male | 53 | positive | 277.32 | 27.53 | 9.25 | 10.07 | negative | 15 |  |  |
| 3701 | male | 54 | negative | 208.81 | 9.24 | 1.52 | 22.60 | negative | 12 |  |  |
| 3702 | male | 54 | negative | 95.04 | 9.81 | 1.74 | 9.69 | negative | 12 |  |  |
| 3703 | male | 53 | negative | 117.74 | 14.76 | 7.91 | 7.98 | negative | 14 |  |  |
| 3704 | male | 53 | positive | 118.13 | 13.01 | 4.93 | 9.08 | negative | 13 |  |  |
| 3705 | male | 53 | positive | 230.80 | 48.80 | 8.10 | 4.73 | negative | 15 |  |  |
| 3706 | male | 53 | negative | 143.57 | 13.59 | 4.37 | 10.56 | negative | 12 |  |  |
| 3707 | male | 53 | positive | 88.65 | 26.07 | 4.61 | 3.40 | negative | 16 |  |  |
| 3708 | male | 53 | negative | 66.85 | 6.04 | 45.34 | 11.07 | negative | 14 |  |  |
| 3709 | male | 53 | negative | 92.78 | 5.74 | 6.17 | 16.16 | negative | 14 |  |  |
| 3710 | male | 53 | positive | 78.30 | 7.40 | 8.30 | 10.58 | negative | 15 |  |  |
| 3711 | male | 53 | positive | 154.52 | 11.05 | 6.08 | 13.98 | negative | 15 |  |  |
| 3712 | male | 53 | negative | 135.65 | 11.23 | 5.79 | 12.08 | negative | 14 |  |  |
| 3713 | male | 53 | negative | 106.47 | 7.91 | 4.30 | 13.46 | negative | 12 |  |  |
| 3714 | male | 53 | positive | 68.79 | 4.09 | 5.56 | 16.82 | negative | 13 |  |  |
| 3715 | male | 53 | positive | 112.30 | 10.25 | 5.33 | 10.96 | negative | 13 |  |  |
| 3716 | male | 53 | negative | 147.39 | 15.56 | 9.36 | 9.47 | negative | 14 |  |  |
| 3717 | male | 54 | negative | 194.30 | 7.69 | 1.64 | 25.27 | negative | 12 |  |  |
| 3718 | male | 53 | positive | 116.71 | 9.99 | 9.26 | 11.68 | negative | 15 |  |  |
| 3719 | male | 53 | positive | 224.25 | 24.26 | 9.84 | 9.24 | negative | 15 |  |  |
| 3720 | male | 53 | negative | 156.09 | 16.14 | 6.74 | 9.67 | negative | 14 |  |  |
| 3721 | male | 53 | negative | 92.15 | 12.69 | 7.72 | 7.26 | negative | 14 |  |  |
| 3722 | male | 53 | positive | 137.07 | 16.28 | 7.75 | 8.42 | negative | 15 |  |  |
| 3723 | male | 53 | positive | 66.82 | 10.80 | 6.72 | 6.19 | negative | 15 |  |  |
| 3724 | male | 53 | negative | 139.12 | 11.00 | 4.89 | 12.65 | negative | 12 |  |  |
| 3725 | male | 54 | negative | 140.85 | 5.10 | 1.64 | 27.62 | negative | 12 |  |  |
| 3726 | male | 53 | negative | 91.90 | 7.40 | 6.30 | 12.42 | negative | 14 |  |  |
| 3727 | male | 54 | negative | 157.65 | 14.79 | 1.55 | 10.66 | negative | 12 |  |  |
| 3728 | male | 54 | negative | 103.44 | 4.97 | 1.58 | 20.81 | negative | 12 |  |  |
| 3729 | male | 54 | positive | 28.91 | 11.10 | 1.11 | 2.60 | negative | 13 |  |  |
| 3730 | male | 53 | positive | 116.57 | 14.10 | 5.50 | 8.27 | negative | 13 |  |  |
| 3731 | male | 53 | positive | 76.27 | 9.65 | 9.08 | 7.90 | negative | 15 |  |  |
| 3732 | male | 53 | negative | 105.77 | 5.30 | 4.60 | 19.96 | negative | 12 |  |  |
| 3733 | male | 53 | positive | 129.65 | 14.97 | 6.91 | 8.66 | negative | 15 |  |  |
| 3734 | male | 53 | negative | 129.53 | 7.87 | 6.00 | 16.46 | negative | 14 |  |  |
| 3735 | male | 53 | negative | 130.23 | 8.63 | 7.82 | 15.09 | negative | 14 |  |  |
| 3736 | male | 53 | positive | 129.20 | 15.20 | 4.40 | 8.50 | negative | 13 |  |  |
| 3737 | male | 53 | positive | 173.81 | 12.72 | 7.00 | 13.66 | negative | 15 |  |  |
| 3738 | male | 53 | negative | 120.86 | 5.70 | 4.44 | 21.20 | negative | 12 |  |  |
| 3739 | male | 53 | negative | 117.22 | 18.33 | 7.77 | 6.39 | negative | 14 |  |  |
| 3740 | male | 53 | negative | 89.55 | 8.09 | 5.40 | 11.07 | negative | 12 |  |  |
| 3741 | male | 54 | negative | 102.45 | 5.83 | 1.52 | 17.57 | negative | 12 |  |  |
| 3742 | male | 53 | positive | 186.74 | 14.00 | 4.00 | 13.34 | negative | 13 |  |  |
| 3743 | male | 53 | positive | 158.49 | 14.98 | 4.31 | 10.58 | negative | 13 |  |  |
| 3744 | female | 53 | positive | 10.57 | 12.80 | 56.38 | 0.83 | negative | 14 |  |  |
| 3745 | male | 53 | positive | 166.18 | 27.89 | 4.47 | 5.96 | negative | 13 |  |  |
| 3746 | male | 54 | negative | 112.03 | 6.02 | 1.52 | 18.61 | negative | 12 |  |  |
| 3747 | male | 53 | positive | 189.49 | 27.83 | 42.49 | 6.81 | negative | 15 |  |  |
| 3748 | male | 53 | positive | 61.41 | 10.69 | 4.51 | 5.74 | negative | 13 |  |  |
| 3749 | male | 53 | negative | 115.73 | 13.04 | 5.54 | 8.88 | negative | 12 |  |  |
| 3750 | male | 53 | positive | 104.80 | 21.70 | 7.30 | 4.83 | negative | 15 |  |  |
| 3751 | male | 53 | positive | 93.09 | 13.22 | 8.18 | 7.04 | negative | 15 |  |  |
| 3752 | male | 53 | negative | 114.21 | 5.54 | 6.44 | 20.62 | negative | 14 |  |  |
| 3753 | male | 53 | positive | 160.73 | 21.10 | 6.70 | 7.62 | negative | 15 |  |  |
| 3754 | male | 54 | negative | 128.71 | 7.80 | 1.67 | 16.50 | negative | 12 |  |  |
| 3755 | male | 53 | positive | 82.63 | 13.30 | 4.11 | 6.21 | negative | 13 |  |  |
| 3756 | male | 53 | negative | 80.33 | 5.93 | 4.10 | 13.55 | negative | 12 |  |  |
| 3757 | male | 53 | positive | 63.76 | 20.17 | 9.02 | 3.16 | negative | 18 |  |  |
| 3758 | male | 53 | negative | 117.98 | 8.58 | 7.19 | 13.75 | negative | 14 |  |  |
| 3759 | male | 53 | negative | 134.21 | 12.35 | 5.74 | 10.87 | negative | 14 |  |  |
| 3760 | male | 53 | positive | 90.82 | 9.47 | 4.29 | 9.59 | negative | 13 |  |  |
| 3761 | male | 53 | positive | 164.01 | 19.70 | 5.99 | 8.33 | negative | 15 |  |  |
| 3762 | male | 53 | negative | 124.65 | 8.06 | 7.49 | 15.47 | negative | 14 |  |  |
| 3763 | male | 53 | positive | 258.04 | 21.96 | 6.47 | 11.75 | negative | 15 |  |  |
| 3764 | male | 53 | negative | 147.37 | 16.10 | 7.29 | 9.15 | negative | 14 |  |  |
| 3765 | male | 53 | positive | 387.56 | 13.19 | 5.10 | 29.38 | negative | 13 |  |  |
| 3766 | male | 53 | positive | 98.42 | 15.07 | 4.67 | 6.53 | negative | 13 |  |  |
| 3767 | male | 54 | negative | 128.18 | 14.88 | 1.58 | 8.61 | negative | 12 |  |  |
| 3768 | male | 53 | positive | 200.60 | 25.42 | 5.32 | 7.89 | negative | 13 |  |  |
| 3769 | male | 53 | negative | 26.89 | 4.48 | 60.00 | 6.00 | negative | 14 |  |  |
| 3770 | male | 53 | positive | 138.70 | 29.77 | 6.12 | 4.66 | negative | 15 |  |  |
| 3771 | male | 53 | positive | 122.20 | 20.50 | 4.80 | 5.96 | negative | 13 |  |  |
| 3772 | male | 53 | positive | 224.28 | 19.97 | 6.30 | 11.23 | negative | 15 |  |  |
| 3773 | male | 53 | negative | 92.60 | 7.90 | 2.90 | 11.72 | negative | 12 |  |  |
| 3774 | male | 53 | positive | 154.87 | 14.59 | 3.50 | 10.61 | negative | 13 |  |  |
| 3775 | male | 53 | negative | 98.10 | 8.02 | 2.69 | 12.23 | negative | 12 |  |  |
| 3776 | male | 53 | positive | 300.00 | 39.49 | 21.08 | 7.60 | negative | 15 |  |  |
| 3777 | male | 53 | negative | 106.35 | 16.45 | 16.27 | 6.47 | negative | 14 |  |  |
| 3778 | male | 53 | negative | 80.30 | 9.40 | 3.40 | 8.54 | negative | 12 |  |  |
| 3779 | male | 53 | negative | 83.43 | 6.10 | 2.62 | 13.68 | negative | 12 |  |  |
| 3780 | male | 53 | negative | 94.43 | 28.81 | 22.53 | 3.28 | negative | 17 |  |  |
| 3781 | male | 53 | negative | 125.05 | 8.39 | 2.39 | 14.90 | negative | 12 |  |  |
| 3782 | male | 53 | negative | 67.49 | 5.47 | 2.51 | 12.34 | negative | 12 |  |  |
| 3783 | male | 53 | negative | 89.30 | 12.30 | 23.90 | 7.26 | negative | 14 |  |  |
| 3784 | male | 53 | negative | 115.60 | 10.10 | 2.64 | 11.45 | negative | 12 |  |  |
| 3785 | male | 53 | negative | 190.09 | 13.55 | 2.91 | 14.03 | negative | 12 |  |  |
| 3786 | male | 53 | positive | 218.42 | 27.30 | 3.54 | 8.00 | negative | 13 |  |  |
| 3787 | male | 53 | negative | 101.80 | 10.40 | 2.10 | 9.79 | negative | 12 |  |  |
| 3788 | male | 53 | positive | 200.15 | 29.54 | 3.81 | 6.78 | negative | 13 |  |  |
| 3789 | male | 53 | negative | 91.70 | 13.40 | 15.80 | 6.84 | negative | 14 |  |  |
| 3790 | male | 53 | positive | 97.00 | 14.90 | 3.70 | 6.51 | negative | 13 |  |  |
| 3791 | male | 53 | negative | 102.96 | 5.07 | 2.00 | 20.31 | negative | 12 |  |  |
| 3792 | male | 53 | negative | 105.98 | 14.99 | 3.94 | 7.07 | negative | 12 |  |  |
| 3793 | male | 53 | negative | 135.56 | 11.36 | 3.43 | 11.93 | negative | 12 |  |  |
| 3794 | male | 53 | negative | 150.65 | 5.56 | 2.19 | 27.10 | negative | 12 |  |  |
| 3795 | male | 53 | negative | 57.60 | 6.20 | 2.40 | 9.29 | negative | 12 |  |  |
| 3796 | male | 53 | negative | 76.81 | 5.62 | 2.77 | 13.67 | negative | 12 |  |  |
| 3797 | male | 53 | negative | 57.42 | 4.29 | 2.43 | 13.38 | negative | 12 |  |  |
| 3798 | male | 53 | positive | 137.69 | 10.47 | 2.76 | 13.15 | negative | 13 |  |  |
| 3799 | male | 53 | negative | 103.90 | 9.90 | 3.70 | 10.49 | negative | 12 |  |  |
| 3800 | male | 53 | negative | 63.84 | 5.21 | 2.02 | 12.25 | negative | 12 |  |  |
| 3801 | male | 53 | positive | 141.33 | 21.42 | 23.24 | 6.60 | negative | 15 |  |  |
| 3802 | male | 53 | negative | 70.90 | 8.40 | 2.10 | 8.44 | negative | 12 |  |  |
| 3803 | male | 53 | negative | 93.77 | 9.50 | 2.76 | 9.87 | negative | 12 |  |  |
| 3804 | male | 53 | negative | 113.95 | 9.21 | 3.64 | 12.37 | negative | 12 |  |  |
| 3805 | male | 53 | negative | 50.20 | 2.80 | 3.30 | 17.93 | negative | 12 |  |  |
| 3806 | male | 53 | positive | 62.30 | 7.90 | 2.80 | 7.89 | negative | 13 |  |  |
| 3807 | male | 53 | negative | 166.60 | 14.40 | 2.90 | 11.57 | negative | 12 |  |  |
| 3808 | male | 53 | negative | 95.32 | 6.60 | 2.19 | 14.44 | negative | 12 |  |  |
| 3809 | male | 53 | negative | 87.10 | 4.37 | 2.59 | 19.93 | negative | 12 |  |  |
| 3810 | male | 53 | negative | 117.12 | 2.53 | 2.74 | 46.29 | negative | 12 |  |  |
| 3811 | male | 53 | negative | 84.69 | 7.26 | 2.02 | 11.67 | negative | 12 |  |  |
| 3812 | male | 53 | negative | 70.75 | 4.01 | 2.69 | 17.64 | negative | 12 |  |  |
| 3813 | male | 53 | positive | 98.34 | 9.59 | 3.93 | 10.25 | negative | 13 |  |  |
| 3814 | male | 53 | positive | 254.24 | 23.43 | 22.82 | 10.85 | negative | 15 |  |  |
| 3815 | male | 53 | negative | 188.11 | 10.65 | 2.57 | 17.66 | negative | 12 |  |  |
| 3816 | male | 53 | negative | 65.96 | 4.02 | 2.24 | 16.41 | negative | 12 |  |  |
| 3817 | male | 53 | negative | 82.16 | 8.84 | 17.70 | 9.29 | negative | 14 |  |  |
| 3818 | male | 53 | negative | 110.20 | 19.28 | 18.50 | 5.72 | negative | 14 |  |  |
| 3819 | male | 53 | negative | 78.60 | 7.20 | 2.40 | 10.92 | negative | 12 |  |  |
| 3820 | male | 53 | negative | 160.96 | 8.86 | 26.49 | 18.17 | negative | 14 |  |  |
| 3821 | male | 53 | negative | 181.46 | 11.64 | 3.75 | 15.59 | negative | 12 |  |  |
| 3822 | male | 53 | positive | 116.84 | 14.42 | 2.53 | 8.10 | negative | 13 |  |  |
| 3823 | male | 53 | positive | 61.77 | 18.01 | 16.51 | 3.43 | negative | 18 |  |  |
| 3824 | male | 53 | negative | 143.56 | 9.98 | 2.03 | 14.38 | negative | 12 |  |  |
| 3825 | male | 53 | negative | 81.21 | 7.76 | 2.13 | 10.47 | negative | 12 |  |  |
| 3826 | male | 53 | negative | 48.97 | 3.08 | 2.37 | 15.90 | negative | 12 |  |  |
| 3827 | male | 53 | negative | 106.52 | 9.25 | 3.48 | 11.52 | negative | 12 |  |  |
| 3828 | male | 53 | positive | 97.97 | 12.55 | 3.07 | 7.81 | negative | 13 |  |  |
| 3829 | male | 53 | negative | 96.62 | 11.14 | 3.39 | 8.67 | negative | 12 |  |  |
| 3830 | male | 53 | negative | 72.79 | 5.03 | 2.55 | 14.47 | negative | 12 |  |  |
| 3831 | male | 53 | negative | 96.85 | 10.72 | 2.65 | 9.03 | negative | 12 |  |  |
| 3832 | male | 53 | negative | 97.01 | 6.37 | 3.31 | 15.23 | negative | 12 |  |  |
| 3833 | male | 53 | positive | 92.66 | 12.39 | 3.87 | 7.48 | negative | 13 |  |  |
| 3834 | male | 53 | positive | 103.47 | 13.02 | 3.25 | 7.95 | negative | 13 |  |  |
| 3835 | male | 53 | positive | 93.20 | 15.00 | 2.10 | 6.21 | negative | 13 |  |  |
| 3836 | male | 53 | negative | 296.19 | 16.55 | 2.36 | 17.90 | negative | 12 |  |  |
| 3837 | male | 53 | negative | 106.43 | 7.45 | 2.64 | 14.29 | negative | 12 |  |  |
| 3838 | male | 53 | positive | 184.61 | 17.12 | 2.75 | 10.78 | negative | 13 |  |  |
| 3839 | male | 53 | negative | 78.08 | 8.34 | 2.37 | 9.36 | negative | 12 |  |  |
| 3840 | male | 53 | negative | 84.32 | 5.56 | 3.18 | 15.17 | negative | 12 |  |  |
| 3841 | male | 53 | negative | 124.80 | 11.90 | 2.70 | 10.49 | negative | 12 |  |  |
| 3842 | male | 53 | negative | 96.30 | 6.78 | 2.28 | 14.20 | negative | 12 |  |  |
| 3843 | male | 53 | negative | 125.73 | 9.49 | 3.87 | 13.25 | negative | 12 |  |  |
| 3844 | male | 53 | negative | 78.47 | 7.06 | 2.15 | 11.11 | negative | 12 |  |  |
| 3845 | male | 53 | negative | 103.09 | 6.88 | 3.98 | 14.98 | negative | 12 |  |  |
| 3846 | male | 53 | negative | 144.95 | 6.37 | 2.68 | 22.76 | negative | 12 |  |  |
| 3847 | male | 53 | negative | 113.43 | 9.62 | 2.26 | 11.79 | negative | 12 |  |  |
| 3848 | male | 53 | negative | 93.53 | 6.47 | 3.78 | 14.46 | negative | 12 |  |  |
| 3849 | male | 53 | negative | 284.69 | 12.92 | 16.51 | 22.03 | negative | 14 |  |  |
| 3850 | male | 53 | positive | 173.75 | 10.14 | 3.37 | 17.14 | negative | 13 |  |  |
| 3851 | male | 53 | negative | 74.02 | 6.63 | 2.00 | 11.16 | negative | 12 |  |  |
| 3852 | male | 53 | negative | 120.34 | 10.99 | 2.50 | 10.95 | negative | 12 |  |  |
| 3853 | male | 53 | negative | 72.66 | 5.47 | 2.56 | 13.28 | negative | 12 |  |  |
| 3854 | male | 53 | negative | 185.02 | 7.83 | 3.31 | 23.63 | negative | 12 |  |  |
| 3855 | male | 53 | negative | 107.20 | 7.82 | 3.28 | 13.71 | negative | 12 |  |  |
| 3856 | male | 53 | negative | 114.74 | 8.81 | 2.12 | 13.02 | negative | 12 |  |  |
| 3857 | male | 53 | negative | 80.26 | 7.17 | 2.86 | 11.19 | negative | 12 |  |  |
| 3858 | male | 53 | negative | 87.05 | 7.30 | 3.61 | 11.92 | negative | 12 |  |  |
| 3859 | male | 53 | negative | 119.52 | 9.42 | 3.25 | 12.69 | negative | 12 |  |  |
| 3860 | female | 53 | negative | 32.30 | 13.40 | 27.10 | 2.41 | negative | 13 |  |  |
| 3861 | male | 53 | negative | 97.58 | 8.05 | 3.34 | 12.12 | negative | 12 |  |  |
| 3862 | male | 53 | negative | 21.06 | 6.95 | 2.06 | 3.03 | negative | 15 |  |  |
| 3863 | male | 53 | negative | 43.67 | 4.53 | 2.12 | 9.64 | negative | 12 |  |  |
| 3864 | male | 53 | positive | 207.60 | 16.22 | 3.02 | 12.80 | negative | 13 |  |  |
| 3865 | male | 53 | negative | 220.28 | 4.90 | 3.16 | 44.96 | negative | 12 |  |  |
| 3866 | male | 53 | negative | 134.76 | 12.53 | 2.75 | 10.75 | negative | 12 |  |  |
| 3867 | male | 53 | negative | 144.64 | 9.98 | 2.82 | 14.49 | negative | 12 |  |  |
| 3868 | male | 53 | negative | 122.89 | 4.07 | 3.95 | 30.19 | negative | 12 |  |  |
| 3869 | male | 53 | negative | 130.02 | 8.18 | 2.70 | 15.89 | negative | 12 |  |  |
| 3870 | male | 53 | negative | 63.83 | 4.99 | 2.66 | 12.79 | negative | 12 |  |  |
| 3871 | male | 53 | negative | 100.63 | 9.92 | 3.02 | 10.14 | negative | 12 |  |  |
| 3872 | male | 53 | negative | 90.53 | 5.95 | 2.15 | 15.22 | negative | 12 |  |  |
| 3873 | male | 53 | negative | 44.93 | 1.89 | 2.08 | 23.77 | negative | 12 |  |  |
| 3874 | male | 53 | negative | 34.50 | 3.73 | 2.22 | 9.25 | negative | 12 |  |  |
| 3875 | male | 53 | negative | 171.99 | 8.12 | 2.02 | 21.18 | negative | 12 |  |  |
| 3876 | male | 53 | negative | 62.59 | 6.12 | 3.77 | 10.23 | negative | 12 |  |  |
| 3877 | male | 53 | negative | 92.82 | 5.57 | 2.25 | 16.66 | negative | 12 |  |  |
| 3878 | male | 53 | negative | 51.20 | 8.20 | 2.10 | 6.24 | negative | 12 |  |  |
| 3879 | male | 53 | negative | 57.00 | 4.90 | 2.90 | 11.63 | negative | 12 |  |  |
| 3880 | male | 53 | negative | 90.89 | 8.79 | 2.37 | 10.34 | negative | 12 |  |  |
| 3881 | male | 53 | positive | 98.88 | 10.44 | 2.30 | 9.47 | negative | 13 |  |  |
| 3882 | male | 53 | negative | 151.79 | 9.19 | 3.47 | 16.52 | negative | 12 |  |  |
| 3883 | male | 53 | negative | 60.79 | 13.54 | 15.87 | 4.49 | negative | 14 |  |  |
| 3884 | male | 53 | negative | 97.64 | 4.25 | 2.12 | 22.97 | negative | 12 |  |  |
| 3885 | male | 53 | negative | 124.39 | 19.76 | 2.52 | 6.30 | negative | 12 |  |  |
| 3886 | male | 53 | negative | 83.90 | 9.70 | 2.70 | 8.65 | negative | 12 |  |  |
| 3887 | male | 53 | negative | 136.90 | 12.10 | 2.10 | 11.31 | negative | 12 |  |  |
| 3888 | male | 53 | negative | 95.73 | 7.90 | 2.66 | 12.12 | negative | 12 |  |  |
| 3889 | male | 53 | negative | 123.45 | 9.24 | 2.48 | 13.36 | negative | 12 |  |  |
| 3890 | male | 53 | negative | 166.51 | 17.47 | 3.38 | 9.53 | negative | 12 |  |  |
| 3891 | male | 53 | negative | 139.90 | 9.80 | 2.70 | 14.28 | negative | 12 |  |  |
| 3892 | male | 53 | negative | 110.34 | 11.19 | 2.40 | 9.86 | negative | 12 |  |  |
| 3893 | male | 53 | negative | 86.90 | 5.70 | 19.30 | 15.25 | negative | 14 |  |  |
| 3894 | male | 53 | negative | 160.54 | 11.95 | 2.72 | 13.43 | negative | 12 |  |  |
| 3895 | male | 53 | negative | 237.47 | 9.03 | 18.42 | 26.30 | negative | 14 |  |  |
| 3896 | male | 53 | negative | 109.46 | 7.50 | 17.10 | 14.59 | negative | 14 |  |  |
| 3897 | male | 53 | positive | 101.95 | 10.52 | 3.04 | 9.69 | negative | 13 |  |  |
| 3898 | male | 53 | negative | 87.50 | 6.90 | 2.30 | 12.68 | negative | 12 |  |  |
| 3899 | male | 53 | positive | 142.03 | 15.14 | 19.41 | 9.38 | negative | 15 |  |  |
| 3900 | male | 53 | positive | 72.22 | 13.22 | 16.48 | 5.46 | negative | 15 |  |  |
| 3901 | male | 53 | negative | 138.44 | 14.30 | 2.39 | 9.68 | negative | 12 |  |  |
| 3902 | male | 53 | negative | 140.59 | 5.67 | 2.50 | 24.80 | negative | 12 |  |  |
| 3903 | male | 53 | positive | 287.60 | 22.22 | 2.73 | 12.94 | negative | 13 |  |  |
| 3904 | male | 53 | negative | 138.28 | 10.39 | 2.81 | 13.31 | negative | 12 |  |  |
| 3905 | male | 53 | positive | 68.68 | 17.42 | 33.05 | 3.94 | negative | 15 |  |  |
| 3906 | male | 52 | positive | 157.44 | 20.40 | 7.24 | 7.72 | negative | 15 |  |  |
| 3907 | male | 53 | negative | 40.74 | 13.75 | 14.73 | 2.96 | negative | 17 |  |  |
| 3908 | male | 53 | negative | 169.34 | 9.70 | 10.72 | 17.46 | negative | 14 |  |  |
| 3909 | male | 53 | negative | 75.82 | 2.75 | 1.75 | 27.57 | negative | 12 |  |  |
| 3910 | male | 53 | negative | 89.60 | 9.70 | 1.50 | 9.24 | negative | 12 |  |  |
| 3911 | male | 52 | negative | 176.95 | 16.34 | 8.01 | 10.83 | negative | 14 |  |  |
| 3912 | male | 53 | positive | 165.61 | 14.27 | 10.81 | 11.61 | negative | 15 |  |  |
| 3913 | male | 53 | negative | 47.22 | 13.11 | 12.84 | 3.60 | negative | 17 |  |  |
| 3914 | male | 52 | positive | 463.55 | 35.60 | 9.90 | 13.02 | negative | 15 |  |  |
| 3915 | male | 53 | negative | 78.25 | 48.15 | 0.50 | 1.63 | negative | 12 |  |  |
| 3916 | male | 52 | positive | 231.83 | 19.30 | 9.12 | 12.01 | negative | 15 |  |  |
| 3917 | male | 52 | positive | 104.59 | 8.29 | 9.34 | 12.62 | negative | 15 |  |  |
| 3918 | male | 52 | positive | 79.99 | 16.96 | 8.16 | 4.72 | negative | 15 |  |  |
| 3919 | male | 53 | negative | 123.04 | 10.16 | 1.97 | 12.11 | negative | 12 |  |  |
| 3920 | male | 52 | positive | 160.53 | 14.36 | 8.73 | 11.18 | negative | 15 |  |  |
| 3921 | male | 53 | negative | 73.78 | 4.06 | 1.77 | 18.17 | negative | 12 |  |  |
| 3922 | male | 53 | negative | 136.91 | 8.57 | 10.39 | 15.98 | negative | 14 |  |  |
| 3923 | male | 52 | positive | 120.60 | 15.78 | 8.41 | 7.64 | negative | 15 |  |  |
| 3924 | male | 53 | negative | 282.55 | 22.48 | 10.22 | 12.57 | negative | 14 |  |  |
| 3925 | male | 53 | positive | 218.91 | 17.68 | 15.79 | 12.38 | negative | 15 |  |  |
| 3926 | male | 53 | negative | 80.78 | 3.56 | 1.60 | 22.69 | negative | 12 |  |  |
| 3927 | female | 52 | negative | 19.69 | 17.74 | 60.00 | 1.11 | negative | 13 |  |  |
| 3928 | male | 53 | positive | 214.18 | 14.08 | 10.70 | 15.21 | negative | 15 |  |  |
| 3929 | male | 52 | negative | 284.63 | 14.17 | 9.94 | 20.09 | negative | 14 |  |  |
| 3930 | male | 53 | negative | 258.98 | 20.73 | 10.08 | 12.49 | negative | 14 |  |  |
| 3931 | male | 52 | positive | 183.53 | 22.18 | 7.35 | 8.27 | negative | 15 |  |  |
| 3932 | male | 53 | positive | 155.42 | 15.02 | 15.78 | 10.35 | negative | 15 |  |  |
| 3933 | male | 53 | negative | 103.60 | 5.93 | 1.58 | 17.47 | negative | 12 |  |  |
| 3934 | male | 52 | positive | 89.34 | 8.24 | 9.23 | 10.84 | negative | 15 |  |  |
| 3935 | male | 53 | negative | 61.80 | 9.60 | 1.70 | 6.44 | negative | 12 |  |  |
| 3936 | male | 53 | negative | 79.15 | 6.35 | 1.50 | 12.46 | negative | 12 |  |  |
| 3937 | male | 52 | positive | 164.65 | 13.86 | 9.75 | 11.88 | negative | 15 |  |  |
| 3938 | male | 52 | positive | 226.48 | 27.84 | 9.23 | 8.14 | negative | 15 |  |  |
| 3939 | male | 52 | negative | 127.72 | 15.88 | 9.31 | 8.04 | negative | 14 |  |  |
| 3940 | male | 52 | positive | 141.12 | 25.39 | 8.16 | 5.56 | negative | 15 |  |  |
| 3941 | male | 52 | positive | 192.54 | 29.66 | 6.91 | 6.49 | negative | 15 |  |  |
| 3942 | male | 53 | negative | 93.92 | 5.81 | 1.62 | 16.17 | negative | 12 |  |  |
| 3943 | male | 52 | negative | 150.67 | 11.16 | 7.41 | 13.50 | negative | 14 |  |  |
| 3944 | male | 53 | negative | 289.32 | 23.96 | 12.55 | 12.08 | negative | 14 |  |  |
| 3945 | male | 53 | negative | 77.65 | 5.05 | 1.54 | 15.38 | negative | 12 |  |  |
| 3946 | male | 53 | negative | 132.04 | 5.93 | 1.73 | 22.27 | negative | 12 |  |  |
| 3947 | male | 53 | negative | 124.13 | 8.33 | 12.19 | 14.90 | negative | 14 |  |  |
| 3948 | male | 52 | negative | 131.64 | 12.69 | 7.92 | 10.37 | negative | 14 |  |  |
| 3949 | male | 53 | negative | 109.87 | 5.89 | 1.56 | 18.65 | negative | 12 |  |  |
| 3950 | male | 52 | negative | 135.58 | 10.00 | 6.92 | 13.56 | negative | 14 |  |  |
| 3951 | male | 52 | negative | 254.62 | 4.34 | 7.81 | 58.67 | negative | 14 |  |  |
| 3952 | male | 52 | positive | 142.90 | 23.60 | 8.40 | 6.06 | negative | 15 |  |  |
| 3953 | male | 53 | negative | 118.25 | 11.30 | 11.78 | 10.46 | negative | 14 |  |  |
| 3954 | male | 52 | positive | 136.51 | 15.28 | 8.52 | 8.93 | negative | 15 |  |  |
| 3955 | male | 52 | positive | 104.81 | 7.76 | 8.43 | 13.51 | negative | 15 |  |  |
| 3956 | male | 53 | positive | 134.11 | 12.66 | 12.20 | 10.59 | negative | 15 |  |  |
| 3957 | male | 53 | negative | 88.76 | 10.07 | 1.82 | 8.81 | negative | 12 |  |  |
| 3958 | male | 53 | negative | 72.60 | 11.70 | 1.70 | 6.21 | negative | 12 |  |  |
| 3959 | male | 53 | negative | 110.44 | 6.97 | 1.74 | 15.85 | negative | 12 |  |  |
| 3960 | male | 53 | positive | 118.54 | 21.05 | 11.85 | 5.63 | negative | 15 |  |  |
| 3961 | male | 53 | negative | 93.92 | 3.27 | 1.96 | 28.72 | negative | 12 |  |  |
| 3962 | male | 52 | negative | 131.50 | 10.76 | 9.16 | 12.22 | negative | 14 |  |  |
| 3963 | male | 52 | negative | 177.38 | 10.07 | 7.90 | 17.61 | negative | 14 |  |  |
| 3964 | male | 53 | negative | 100.83 | 6.36 | 1.93 | 15.85 | negative | 12 |  |  |
| 3965 | male | 53 | negative | 122.22 | 5.94 | 1.65 | 20.58 | negative | 12 |  |  |
| 3966 | male | 53 | negative | 176.62 | 11.24 | 1.59 | 15.71 | negative | 12 |  |  |
| 3967 | male | 53 | positive | 217.21 | 20.80 | 10.10 | 10.44 | negative | 15 |  |  |
| 3968 | male | 53 | negative | 227.93 | 8.49 | 1.71 | 26.85 | negative | 12 |  |  |
| 3969 | male | 53 | negative | 81.42 | 4.21 | 10.52 | 19.34 | negative | 14 |  |  |
| 3970 | male | 52 | positive | 144.40 | 15.23 | 9.29 | 9.48 | negative | 15 |  |  |
| 3971 | male | 53 | negative | 88.40 | 6.67 | 12.36 | 13.25 | negative | 14 |  |  |
| 3972 | male | 53 | positive | 102.49 | 10.40 | 12.18 | 9.85 | negative | 15 |  |  |
| 3973 | male | 52 | negative | 150.43 | 17.61 | 7.03 | 8.54 | negative | 14 |  |  |
| 3974 | male | 52 | positive | 188.24 | 27.90 | 6.95 | 6.75 | negative | 15 |  |  |
| 3975 | male | 53 | positive | 215.50 | 17.56 | 10.94 | 12.27 | negative | 15 |  |  |
| 3976 | male | 52 | positive | 124.09 | 16.46 | 9.41 | 7.54 | negative | 15 |  |  |
| 3977 | male | 53 | positive | 147.97 | 17.48 | 12.63 | 8.47 | negative | 15 |  |  |
| 3978 | male | 52 | positive | 189.30 | 29.70 | 7.80 | 6.37 | negative | 15 |  |  |
| 3979 | male | 53 | negative | 204.07 | 12.20 | 1.65 | 16.73 | negative | 12 |  |  |
| 3980 | male | 53 | negative | 105.55 | 6.90 | 1.95 | 15.30 | negative | 12 |  |  |
| 3981 | male | 52 | positive | 131.55 | 12.26 | 8.50 | 10.73 | negative | 15 |  |  |
| 3982 | male | 53 | positive | 129.89 | 23.93 | 10.46 | 5.43 | negative | 15 |  |  |
| 3983 | male | 52 | positive | 156.70 | 18.88 | 7.24 | 8.30 | negative | 15 |  |  |
| 3984 | male | 53 | negative | 87.19 | 4.92 | 1.51 | 17.72 | negative | 12 |  |  |
| 3985 | male | 52 | positive | 168.39 | 18.30 | 6.87 | 9.20 | negative | 15 |  |  |
| 3986 | male | 52 | positive | 100.02 | 9.47 | 8.68 | 10.56 | negative | 15 |  |  |
| 3987 | male | 53 | negative | 100.38 | 11.93 | 10.00 | 8.41 | negative | 14 |  |  |
| 3988 | male | 53 | positive | 108.14 | 15.77 | 14.70 | 6.86 | negative | 15 |  |  |
| 3989 | male | 53 | positive | 92.46 | 13.42 | 11.84 | 6.89 | negative | 15 |  |  |
| 3990 | male | 53 | negative | 95.34 | 7.51 | 1.58 | 12.70 | negative | 12 |  |  |
| 3991 | male | 53 | positive | 137.60 | 14.00 | 10.10 | 9.83 | negative | 15 |  |  |
| 3992 | male | 53 | positive | 192.50 | 21.67 | 14.30 | 8.88 | negative | 15 |  |  |
| 3993 | male | 53 | negative | 221.30 | 14.02 | 1.76 | 15.78 | negative | 12 |  |  |
| 3994 | male | 53 | positive | 123.35 | 20.15 | 14.10 | 6.12 | negative | 15 |  |  |
| 3995 | male | 53 | negative | 66.96 | 5.14 | 1.61 | 13.03 | negative | 12 |  |  |
| 3996 | male | 52 | negative | 141.29 | 6.87 | 7.57 | 20.57 | negative | 14 |  |  |
| 3997 | male | 53 | positive | 165.10 | 21.20 | 14.24 | 7.79 | negative | 15 |  |  |
| 3998 | male | 53 | negative | 118.11 | 11.27 | 1.89 | 10.48 | negative | 12 |  |  |
| 3999 | male | 53 | negative | 142.19 | 9.63 | 1.64 | 14.77 | negative | 12 |  |  |
| 4000 | male | 53 | negative | 86.82 | 6.07 | 1.62 | 14.30 | negative | 12 |  |  |
| 4001 | male | 53 | negative | 156.90 | 24.60 | 11.30 | 6.38 | negative | 14 |  |  |
| 4002 | male | 53 | negative | 133.47 | 6.51 | 1.73 | 20.50 | negative | 12 |  |  |
| 4003 | male | 52 | negative | 206.50 | 23.39 | 9.49 | 8.83 | negative | 14 |  |  |
| 4004 | male | 53 | positive | 224.25 | 17.32 | 12.86 | 12.95 | negative | 15 |  |  |
| 4005 | male | 53 | negative | 125.92 | 6.74 | 1.95 | 18.68 | negative | 12 |  |  |
| 4006 | male | 52 | negative | 119.97 | 5.65 | 7.11 | 21.23 | negative | 14 |  |  |
| 4007 | male | 52 | negative | 97.76 | 9.61 | 8.90 | 10.17 | negative | 14 |  |  |
| 4008 | male | 53 | negative | 93.19 | 5.67 | 1.61 | 16.44 | negative | 12 |  |  |
| 4009 | male | 53 | negative | 88.48 | 6.88 | 1.63 | 12.86 | negative | 12 |  |  |
| 4010 | male | 52 | positive | 158.76 | 23.80 | 9.62 | 6.67 | negative | 15 |  |  |
| 4011 | male | 53 | negative | 81.50 | 10.50 | 11.70 | 7.76 | negative | 14 |  |  |
| 4012 | male | 53 | positive | 102.71 | 10.01 | 1.83 | 10.26 | negative | 13 |  |  |
| 4013 | male | 53 | negative | 110.46 | 5.79 | 1.56 | 19.08 | negative | 12 |  |  |
| 4014 | male | 53 | negative | 132.03 | 10.44 | 1.51 | 12.65 | negative | 12 |  |  |
| 4015 | male | 52 | negative | 124.39 | 9.66 | 8.09 | 12.88 | negative | 14 |  |  |
| 4016 | male | 53 | positive | 95.96 | 6.41 | 10.00 | 14.97 | negative | 15 |  |  |
| 4017 | male | 53 | negative | 70.10 | 9.00 | 1.90 | 7.79 | negative | 12 |  |  |
| 4018 | male | 53 | negative | 59.56 | 3.79 | 1.87 | 15.72 | negative | 12 |  |  |
| 4019 | male | 52 | positive | 127.84 | 13.31 | 7.36 | 9.60 | negative | 15 |  |  |
| 4020 | male | 53 | positive | 247.98 | 21.65 | 10.46 | 11.45 | negative | 15 |  |  |
| 4021 | male | 52 | positive | 154.40 | 17.58 | 7.15 | 8.78 | negative | 15 |  |  |
| 4022 | male | 52 | negative | 112.82 | 10.67 | 9.63 | 10.57 | negative | 14 |  |  |
| 4023 | male | 52 | positive | 259.19 | 40.01 | 7.46 | 6.48 | negative | 15 |  |  |
| 4024 | male | 52 | negative | 122.07 | 10.61 | 7.50 | 11.51 | negative | 14 |  |  |
| 4025 | male | 53 | positive | 221.21 | 28.18 | 10.86 | 7.85 | negative | 15 |  |  |
| 4026 | male | 52 | negative | 107.09 | 5.51 | 8.29 | 19.44 | negative | 14 |  |  |
| 4027 | male | 53 | negative | 97.19 | 5.38 | 1.76 | 18.07 | negative | 12 |  |  |
| 4028 | female | 53 | negative | 11.64 | 8.74 | 60.00 | 1.33 | negative | 13 |  |  |
| 4029 | male | 53 | negative | 60.86 | 3.70 | 1.68 | 16.45 | negative | 12 |  |  |
| 4030 | male | 53 | negative | 52.35 | 3.08 | 1.55 | 17.00 | negative | 12 |  |  |
| 4031 | male | 53 | negative | 207.36 | 15.91 | 1.92 | 13.03 | negative | 12 |  |  |
| 4032 | male | 53 | negative | 163.36 | 20.72 | 11.20 | 7.88 | negative | 14 |  |  |
| 4033 | male | 52 | negative | 127.95 | 13.84 | 7.25 | 9.24 | negative | 14 |  |  |
| 4034 | male | 53 | positive | 132.56 | 19.09 | 11.50 | 6.94 | negative | 15 |  |  |
| 4035 | male | 52 | positive | 150.07 | 16.81 | 6.44 | 8.93 | negative | 15 |  |  |
| 4036 | male | 52 | negative | 34.81 | 10.09 | 59.26 | 3.45 | negative | 17 |  |  |
| 4037 | male | 52 | positive | 111.23 | 17.92 | 3.18 | 6.21 | negative | 13 |  |  |
| 4038 | male | 52 | positive | 137.72 | 13.78 | 3.41 | 9.99 | negative | 13 |  |  |
| 4039 | male | 52 | negative | 224.73 | 26.32 | 6.55 | 8.54 | negative | 14 |  |  |
| 4040 | male | 52 | positive | 162.54 | 19.03 | 3.37 | 8.54 | negative | 13 |  |  |
| 4041 | male | 52 | negative | 158.90 | 21.00 | 4.40 | 7.57 | negative | 12 |  |  |
| 4042 | male | 52 | negative | 144.86 | 13.55 | 3.32 | 10.69 | negative | 12 |  |  |
| 4043 | male | 52 | positive | 172.67 | 16.22 | 5.35 | 10.65 | negative | 13 |  |  |
| 4044 | male | 52 | negative | 142.08 | 9.34 | 5.01 | 15.21 | negative | 12 |  |  |
| 4045 | male | 52 | negative | 73.27 | 5.73 | 3.22 | 12.79 | negative | 12 |  |  |
| 4046 | male | 52 | negative | 88.01 | 6.33 | 5.55 | 13.90 | negative | 12 |  |  |
| 4047 | male | 52 | negative | 53.43 | 15.78 | 4.96 | 3.39 | negative | 15 |  |  |
| 4048 | male | 52 | positive | 157.27 | 13.20 | 5.58 | 11.91 | negative | 13 |  |  |
| 4049 | male | 52 | positive | 76.04 | 7.61 | 3.22 | 9.99 | negative | 13 |  |  |
| 4050 | male | 52 | negative | 65.99 | 12.66 | 3.53 | 5.21 | negative | 12 |  |  |
| 4051 | male | 52 | positive | 81.92 | 13.38 | 36.93 | 6.12 | negative | 15 |  |  |
| 4052 | male | 52 | positive | 59.79 | 8.16 | 22.01 | 7.33 | negative | 15 |  |  |
| 4053 | male | 52 | positive | 110.68 | 16.15 | 5.02 | 6.85 | negative | 13 |  |  |
| 4054 | male | 52 | positive | 295.60 | 35.06 | 4.73 | 8.43 | negative | 13 |  |  |
| 4055 | male | 52 | negative | 105.59 | 5.99 | 3.03 | 17.63 | negative | 12 |  |  |
| 4056 | male | 52 | negative | 66.29 | 4.04 | 3.04 | 16.41 | negative | 12 |  |  |
| 4057 | male | 52 | negative | 74.34 | 11.24 | 3.29 | 6.61 | negative | 12 |  |  |
| 4058 | male | 52 | positive | 137.91 | 14.95 | 5.03 | 9.22 | negative | 13 |  |  |
| 4059 | male | 52 | positive | 149.02 | 14.85 | 6.67 | 10.04 | negative | 15 |  |  |
| 4060 | male | 52 | negative | 86.00 | 7.40 | 3.00 | 11.62 | negative | 12 |  |  |
| 4061 | male | 52 | negative | 109.74 | 8.95 | 2.93 | 12.26 | negative | 12 |  |  |
| 4062 | male | 52 | negative | 125.17 | 11.62 | 34.50 | 10.77 | negative | 14 |  |  |
| 4063 | male | 52 | positive | 134.47 | 12.56 | 6.28 | 10.71 | negative | 15 |  |  |
| 4064 | male | 52 | negative | 111.08 | 13.05 | 4.85 | 8.51 | negative | 12 |  |  |
| 4065 | male | 52 | positive | 100.74 | 9.59 | 3.22 | 10.50 | negative | 13 |  |  |
| 4066 | male | 52 | positive | 117.06 | 21.13 | 4.39 | 5.54 | negative | 13 |  |  |
| 4067 | male | 52 | negative | 61.28 | 5.16 | 3.12 | 11.88 | negative | 12 |  |  |
| 4068 | male | 52 | negative | 92.32 | 8.51 | 3.98 | 10.85 | negative | 12 |  |  |
| 4069 | male | 52 | positive | 96.22 | 12.08 | 4.31 | 7.97 | negative | 13 |  |  |
| 4070 | male | 52 | positive | 48.65 | 8.84 | 6.82 | 5.50 | negative | 15 |  |  |
| 4071 | male | 52 | positive | 110.99 | 12.65 | 5.84 | 8.77 | negative | 15 |  |  |
| 4072 | male | 52 | negative | 191.46 | 12.10 | 3.84 | 15.82 | negative | 12 |  |  |
| 4073 | male | 52 | negative | 109.32 | 6.37 | 2.99 | 17.16 | negative | 12 |  |  |
| 4074 | male | 52 | negative | 82.80 | 11.00 | 5.80 | 7.53 | negative | 14 |  |  |
| 4075 | male | 52 | negative | 83.50 | 16.10 | 6.40 | 5.19 | negative | 14 |  |  |
| 4076 | male | 52 | positive | 197.41 | 22.48 | 3.71 | 8.78 | negative | 13 |  |  |
| 4077 | male | 52 | negative | 72.64 | 7.96 | 4.40 | 9.13 | negative | 12 |  |  |
| 4078 | male | 52 | negative | 145.16 | 11.12 | 4.16 | 13.05 | negative | 12 |  |  |
| 4079 | female | 52 | negative | 12.58 | 5.16 | 52.30 | 2.44 | negative | 13 |  |  |
| 4080 | male | 52 | negative | 98.34 | 5.53 | 6.23 | 17.78 | negative | 14 |  |  |
| 4081 | female | 52 | negative | 7.92 | 4.67 | 59.68 | 1.70 | negative | 13 |  |  |
| 4082 | male | 52 | positive | 107.73 | 10.87 | 4.42 | 9.91 | negative | 13 |  |  |
| 4083 | male | 52 | negative | 95.00 | 16.03 | 39.13 | 5.93 | negative | 14 |  |  |
| 4084 | male | 52 | negative | 88.20 | 20.60 | 5.90 | 4.28 | negative | 14 |  |  |
| 4085 | male | 52 | positive | 159.32 | 15.38 | 4.20 | 10.36 | negative | 13 |  |  |
| 4086 | male | 52 | negative | 148.34 | 21.27 | 6.47 | 6.97 | negative | 14 |  |  |
| 4087 | male | 52 | negative | 97.02 | 9.70 | 4.02 | 10.00 | negative | 12 |  |  |
| 4088 | male | 52 | positive | 159.67 | 14.42 | 3.34 | 11.07 | negative | 13 |  |  |
| 4089 | male | 52 | negative | 134.41 | 5.36 | 4.29 | 25.08 | negative | 12 |  |  |
| 4090 | male | 52 | negative | 116.30 | 8.51 | 4.26 | 13.67 | negative | 12 |  |  |
| 4091 | male | 52 | positive | 152.64 | 21.39 | 6.22 | 7.14 | negative | 15 |  |  |
| 4092 | male | 52 | negative | 281.73 | 10.92 | 5.59 | 25.80 | negative | 12 |  |  |
| 4093 | male | 52 | negative | 27.51 | 2.89 | 4.02 | 9.52 | negative | 12 |  |  |
| 4094 | male | 52 | positive | 176.15 | 14.69 | 6.57 | 11.99 | negative | 15 |  |  |
| 4095 | male | 52 | negative | 126.28 | 6.63 | 2.91 | 19.05 | negative | 12 |  |  |
| 4096 | male | 52 | negative | 144.09 | 8.05 | 3.88 | 17.90 | negative | 12 |  |  |
| 4097 | male | 52 | negative | 249.71 | 33.06 | 37.88 | 7.55 | negative | 14 |  |  |
| 4098 | male | 52 | positive | 76.67 | 20.12 | 4.74 | 3.81 | negative | 16 |  |  |
| 4099 | male | 52 | negative | 68.05 | 3.51 | 4.53 | 19.39 | negative | 12 |  |  |
| 4100 | male | 52 | positive | 300.00 | 22.40 | 27.12 | 13.39 | negative | 15 |  |  |
| 4101 | male | 52 | positive | 178.65 | 12.78 | 5.03 | 13.98 | negative | 13 |  |  |
| 4102 | male | 52 | negative | 288.37 | 18.73 | 6.29 | 15.40 | negative | 14 |  |  |
| 4103 | male | 52 | positive | 75.10 | 13.00 | 5.70 | 5.78 | negative | 13 |  |  |
| 4104 | male | 52 | positive | 136.79 | 17.88 | 3.30 | 7.65 | negative | 13 |  |  |
| 4105 | male | 52 | negative | 71.91 | 3.89 | 4.10 | 18.49 | negative | 12 |  |  |
| 4106 | male | 52 | positive | 140.26 | 21.41 | 39.07 | 6.55 | negative | 15 |  |  |
| 4107 | male | 52 | negative | 100.23 | 10.13 | 4.53 | 9.89 | negative | 12 |  |  |
| 4108 | male | 52 | negative | 86.95 | 5.38 | 4.21 | 16.16 | negative | 12 |  |  |
| 4109 | male | 52 | negative | 74.27 | 6.86 | 3.42 | 10.83 | negative | 12 |  |  |
| 4110 | male | 52 | negative | 149.32 | 10.92 | 4.10 | 13.67 | negative | 12 |  |  |
| 4111 | male | 52 | positive | 119.45 | 9.07 | 6.78 | 13.17 | negative | 15 |  |  |
| 4112 | male | 52 | positive | 210.70 | 10.68 | 5.70 | 19.73 | negative | 13 |  |  |
| 4113 | male | 52 | negative | 147.57 | 10.05 | 4.83 | 14.68 | negative | 12 |  |  |
| 4114 | male | 52 | positive | 72.72 | 11.69 | 4.16 | 6.22 | negative | 13 |  |  |
| 4115 | male | 52 | negative | 186.06 | 9.57 | 3.19 | 19.44 | negative | 12 |  |  |
| 4116 | male | 52 | negative | 120.49 | 3.65 | 3.25 | 33.01 | negative | 12 |  |  |
| 4117 | male | 52 | negative | 67.12 | 19.85 | 5.15 | 3.38 | negative | 15 |  |  |
| 4118 | male | 52 | positive | 58.51 | 10.29 | 3.05 | 5.69 | negative | 13 |  |  |
| 4119 | male | 52 | negative | 88.59 | 6.88 | 6.35 | 12.88 | negative | 14 |  |  |
| 4120 | male | 52 | positive | 129.54 | 14.82 | 3.73 | 8.74 | negative | 13 |  |  |
| 4121 | male | 52 | positive | 167.47 | 19.20 | 5.08 | 8.72 | negative | 13 |  |  |
| 4122 | male | 52 | negative | 107.57 | 7.50 | 4.45 | 14.34 | negative | 12 |  |  |
| 4123 | male | 52 | positive | 117.38 | 8.89 | 3.35 | 13.20 | negative | 13 |  |  |
| 4124 | male | 52 | negative | 151.62 | 7.12 | 3.56 | 21.29 | negative | 12 |  |  |
| 4125 | male | 52 | positive | 266.77 | 29.13 | 6.33 | 9.16 | negative | 15 |  |  |
| 4126 | male | 52 | positive | 196.60 | 20.10 | 5.20 | 9.78 | negative | 13 |  |  |
| 4127 | male | 52 | negative | 157.49 | 17.68 | 20.70 | 8.91 | negative | 14 |  |  |
| 4128 | male | 52 | negative | 95.97 | 5.25 | 4.00 | 18.28 | negative | 12 |  |  |
| 4129 | male | 52 | negative | 89.58 | 8.45 | 5.26 | 10.60 | negative | 12 |  |  |
| 4130 | female | 52 | negative | 1.90 | 7.26 | 22.90 | 0.26 | negative | 13 |  |  |
| 4131 | male | 52 | negative | 140.65 | 13.37 | 5.49 | 10.52 | negative | 12 |  |  |
| 4132 | male | 52 | negative | 64.61 | 4.71 | 3.13 | 13.72 | negative | 12 |  |  |
| 4133 | male | 52 | positive | 144.68 | 19.50 | 4.69 | 7.42 | negative | 13 |  |  |
| 4134 | male | 52 | positive | 146.71 | 21.80 | 40.71 | 6.73 | negative | 15 |  |  |
| 4135 | male | 52 | positive | 149.30 | 14.00 | 6.50 | 10.66 | negative | 15 |  |  |
| 4136 | male | 52 | negative | 178.46 | 13.55 | 4.87 | 13.17 | negative | 12 |  |  |
| 4137 | male | 52 | positive | 111.50 | 22.40 | 6.40 | 4.98 | negative | 15 |  |  |
| 4138 | male | 52 | negative | 53.02 | 5.03 | 3.50 | 10.54 | negative | 12 |  |  |
| 4139 | male | 52 | positive | 62.56 | 25.06 | 24.95 | 2.50 | negative | 18 |  |  |
| 4140 | male | 52 | negative | 94.74 | 10.69 | 3.28 | 8.86 | negative | 12 |  |  |
| 4141 | male | 52 | positive | 94.80 | 7.55 | 4.07 | 12.56 | negative | 13 |  |  |
| 4142 | male | 52 | negative | 92.02 | 10.15 | 3.40 | 9.07 | negative | 12 |  |  |
| 4143 | male | 52 | negative | 90.62 | 4.58 | 4.52 | 19.79 | negative | 12 |  |  |
| 4144 | male | 52 | negative | 100.34 | 7.03 | 2.96 | 14.27 | negative | 12 |  |  |
| 4145 | male | 52 | positive | 123.31 | 6.69 | 6.78 | 18.43 | negative | 15 |  |  |
| 4146 | male | 52 | negative | 99.11 | 9.91 | 5.69 | 10.00 | negative | 12 |  |  |
| 4147 | male | 52 | positive | 111.09 | 11.46 | 3.80 | 9.69 | negative | 13 |  |  |
| 4148 | male | 52 | positive | 152.21 | 12.01 | 3.64 | 12.67 | negative | 13 |  |  |
| 4149 | male | 52 | negative | 107.95 | 9.51 | 5.95 | 11.35 | negative | 14 |  |  |
| 4150 | male | 52 | positive | 251.37 | 15.93 | 6.30 | 15.78 | negative | 15 |  |  |
| 4151 | male | 52 | positive | 144.71 | 19.78 | 5.88 | 7.32 | negative | 15 |  |  |
| 4152 | male | 52 | negative | 144.55 | 8.94 | 3.45 | 16.17 | negative | 12 |  |  |
| 4153 | male | 52 | negative | 281.39 | 13.45 | 4.80 | 20.92 | negative | 12 |  |  |
| 4154 | male | 52 | positive | 159.86 | 13.41 | 4.62 | 11.92 | negative | 13 |  |  |
| 4155 | male | 52 | negative | 118.06 | 11.77 | 5.36 | 10.03 | negative | 12 |  |  |
| 4156 | male | 52 | negative | 147.88 | 14.29 | 5.45 | 10.35 | negative | 12 |  |  |
| 4157 | male | 52 | negative | 74.37 | 5.66 | 3.09 | 13.14 | negative | 12 |  |  |
| 4158 | male | 52 | negative | 181.39 | 15.98 | 4.35 | 11.35 | negative | 12 |  |  |
| 4159 | male | 52 | positive | 128.30 | 12.10 | 4.10 | 10.60 | negative | 13 |  |  |
| 4160 | male | 52 | negative | 85.97 | 6.28 | 3.03 | 13.69 | negative | 12 |  |  |
| 4161 | male | 52 | positive | 93.62 | 9.61 | 5.30 | 9.74 | negative | 13 |  |  |
| 4162 | male | 52 | negative | 116.64 | 19.55 | 26.75 | 5.97 | negative | 14 |  |  |
| 4163 | male | 52 | positive | 107.15 | 19.01 | 5.76 | 5.64 | negative | 15 |  |  |
| 4164 | male | 52 | positive | 93.60 | 28.00 | 6.30 | 3.34 | negative | 18 |  |  |
| 4165 | male | 52 | positive | 89.20 | 12.80 | 5.20 | 6.97 | negative | 13 |  |  |
| 4166 | male | 52 | positive | 108.20 | 12.02 | 38.60 | 9.00 | negative | 15 |  |  |
| 4167 | male | 52 | positive | 108.52 | 17.77 | 5.46 | 6.11 | negative | 13 |  |  |
| 4168 | male | 52 | negative | 161.95 | 15.23 | 4.43 | 10.63 | negative | 12 |  |  |
| 4169 | male | 52 | negative | 234.40 | 24.36 | 4.59 | 9.62 | negative | 12 |  |  |
| 4170 | male | 52 | negative | 232.11 | 17.68 | 2.98 | 13.13 | negative | 12 |  |  |
| 4171 | male | 52 | positive | 132.05 | 14.21 | 3.09 | 9.29 | negative | 13 |  |  |
| 4172 | male | 52 | negative | 118.21 | 9.97 | 4.83 | 11.86 | negative | 12 |  |  |
| 4173 | male | 52 | negative | 94.53 | 8.50 | 2.95 | 11.12 | negative | 12 |  |  |
| 4174 | male | 52 | positive | 104.77 | 13.72 | 5.55 | 7.64 | negative | 13 |  |  |
| 4175 | male | 52 | negative | 113.29 | 8.87 | 3.57 | 12.77 | negative | 12 |  |  |
| 4176 | male | 52 | negative | 100.08 | 9.08 | 3.49 | 11.02 | negative | 12 |  |  |
| 4177 | male | 52 | negative | 50.68 | 9.46 | 57.80 | 5.36 | negative | 14 |  |  |
| 4178 | male | 52 | negative | 68.72 | 13.85 | 5.65 | 4.96 | negative | 12 |  |  |
| 4179 | male | 52 | negative | 139.90 | 16.38 | 3.91 | 8.54 | negative | 12 |  |  |
| 4180 | male | 52 | negative | 44.89 | 3.12 | 3.81 | 14.39 | negative | 12 |  |  |
| 4181 | male | 52 | negative | 67.38 | 5.58 | 4.21 | 12.08 | negative | 12 |  |  |
| 4182 | male | 52 | negative | 112.04 | 8.52 | 3.21 | 13.15 | negative | 12 |  |  |
| 4183 | male | 52 | positive | 161.64 | 9.62 | 5.49 | 16.80 | negative | 13 |  |  |
| 4184 | male | 52 | negative | 115.60 | 10.14 | 6.60 | 11.40 | negative | 14 |  |  |
| 4185 | male | 52 | positive | 145.49 | 7.99 | 6.10 | 18.21 | negative | 15 |  |  |
| 4186 | male | 52 | negative | 129.36 | 10.93 | 5.66 | 11.84 | negative | 12 |  |  |
| 4187 | male | 52 | negative | 89.03 | 7.57 | 3.11 | 11.76 | negative | 12 |  |  |
| 4188 | male | 52 | negative | 300.00 | 19.53 | 43.95 | 15.36 | negative | 14 |  |  |
| 4189 | male | 52 | negative | 51.84 | 3.76 | 3.79 | 13.79 | negative | 12 |  |  |
| 4190 | male | 52 | negative | 258.46 | 18.91 | 5.96 | 13.67 | negative | 14 |  |  |
| 4191 | male | 52 | positive | 289.63 | 34.02 | 28.69 | 8.51 | negative | 15 |  |  |
| 4192 | male | 52 | negative | 235.96 | 6.75 | 53.35 | 34.96 | negative | 14 |  |  |
| 4193 | male | 52 | positive | 58.27 | 11.16 | 3.72 | 5.22 | negative | 13 |  |  |
| 4194 | female | 52 | negative | 65.71 | 29.17 | 51.30 | 2.25 | negative | 13 |  |  |
| 4195 | male | 52 | positive | 111.72 | 7.63 | 4.81 | 14.64 | negative | 13 |  |  |
| 4196 | male | 52 | negative | 269.56 | 11.80 | 4.36 | 22.84 | negative | 12 |  |  |
| 4197 | male | 52 | negative | 172.89 | 10.53 | 5.91 | 16.42 | negative | 14 |  |  |
| 4198 | male | 52 | positive | 86.20 | 8.80 | 16.90 | 9.80 | negative | 15 |  |  |
| 4199 | male | 52 | positive | 122.83 | 27.30 | 14.80 | 4.50 | negative | 15 |  |  |
| 4200 | male | 52 | negative | 95.87 | 9.25 | 2.70 | 10.36 | negative | 12 |  |  |
| 4201 | male | 52 | negative | 275.38 | 12.65 | 2.49 | 21.77 | negative | 12 |  |  |
| 4202 | male | 52 | negative | 79.02 | 7.24 | 2.57 | 10.91 | negative | 12 |  |  |
| 4203 | male | 52 | positive | 225.89 | 17.77 | 11.22 | 12.71 | negative | 15 |  |  |
| 4204 | male | 52 | negative | 272.16 | 17.68 | 13.86 | 15.39 | negative | 14 |  |  |
| 4205 | male | 52 | negative | 122.02 | 6.69 | 1.94 | 18.24 | negative | 12 |  |  |
| 4206 | male | 52 | negative | 94.43 | 10.85 | 2.39 | 8.70 | negative | 12 |  |  |
| 4207 | male | 52 | positive | 206.56 | 26.98 | 10.10 | 7.66 | negative | 15 |  |  |
| 4208 | male | 52 | negative | 65.20 | 7.00 | 2.10 | 9.31 | negative | 12 |  |  |
| 4209 | male | 52 | negative | 86.08 | 8.39 | 2.91 | 10.26 | negative | 12 |  |  |
| 4210 | male | 52 | negative | 135.53 | 6.99 | 12.89 | 19.39 | negative | 14 |  |  |
| 4211 | male | 52 | positive | 120.20 | 20.10 | 12.50 | 5.98 | negative | 15 |  |  |
| 4212 | male | 52 | negative | 93.42 | 5.07 | 1.94 | 18.43 | negative | 12 |  |  |
| 4213 | male | 52 | negative | 100.54 | 5.45 | 2.60 | 18.45 | negative | 12 |  |  |
| 4214 | male | 52 | negative | 89.76 | 4.21 | 2.76 | 21.32 | negative | 12 |  |  |
| 4215 | male | 52 | negative | 88.90 | 7.30 | 2.30 | 12.18 | negative | 12 |  |  |
| 4216 | male | 52 | negative | 122.77 | 6.80 | 2.72 | 18.05 | negative | 12 |  |  |
| 4217 | male | 52 | negative | 225.61 | 21.87 | 18.13 | 10.32 | negative | 14 |  |  |
| 4218 | male | 52 | negative | 104.16 | 6.68 | 2.69 | 15.59 | negative | 12 |  |  |
| 4219 | male | 52 | negative | 161.13 | 18.56 | 18.28 | 8.68 | negative | 14 |  |  |
| 4220 | male | 52 | positive | 164.51 | 21.85 | 16.40 | 7.53 | negative | 15 |  |  |
| 4221 | male | 52 | negative | 40.16 | 3.07 | 2.62 | 13.08 | negative | 12 |  |  |
| 4222 | male | 52 | negative | 81.10 | 9.50 | 2.50 | 8.54 | negative | 12 |  |  |
| 4223 | male | 52 | positive | 85.58 | 11.46 | 2.59 | 7.47 | negative | 13 |  |  |
| 4224 | male | 52 | negative | 115.91 | 11.13 | 2.32 | 10.41 | negative | 12 |  |  |
| 4225 | male | 52 | negative | 89.00 | 13.30 | 1.80 | 6.69 | negative | 12 |  |  |
| 4226 | male | 52 | negative | 163.28 | 9.79 | 11.14 | 16.68 | negative | 14 |  |  |
| 4227 | male | 52 | positive | 158.24 | 14.47 | 18.41 | 10.94 | negative | 15 |  |  |
| 4228 | male | 52 | negative | 123.83 | 9.04 | 2.17 | 13.70 | negative | 12 |  |  |
| 4229 | male | 52 | negative | 130.95 | 5.20 | 2.88 | 25.18 | negative | 12 |  |  |
| 4230 | male | 52 | positive | 62.66 | 5.49 | 2.06 | 11.41 | negative | 13 |  |  |
| 4231 | male | 52 | negative | 164.53 | 12.11 | 2.26 | 13.59 | negative | 12 |  |  |
| 4232 | male | 52 | negative | 171.23 | 7.92 | 2.27 | 21.62 | negative | 12 |  |  |
| 4233 | male | 52 | positive | 118.57 | 16.44 | 2.24 | 7.21 | negative | 13 |  |  |
| 4234 | male | 52 | positive | 300.00 | 41.70 | 13.93 | 7.19 | negative | 15 |  |  |
| 4235 | male | 52 | positive | 142.81 | 19.09 | 16.87 | 7.48 | negative | 15 |  |  |
| 4236 | male | 52 | positive | 86.85 | 11.37 | 2.71 | 7.64 | negative | 13 |  |  |
| 4237 | male | 52 | positive | 178.07 | 16.65 | 16.06 | 10.69 | negative | 15 |  |  |
| 4238 | male | 52 | negative | 186.94 | 18.88 | 2.78 | 9.90 | negative | 12 |  |  |
| 4239 | male | 52 | positive | 152.98 | 11.06 | 2.08 | 13.83 | negative | 13 |  |  |
| 4240 | male | 52 | negative | 123.30 | 15.10 | 11.04 | 8.17 | negative | 14 |  |  |
| 4241 | male | 52 | positive | 87.01 | 10.38 | 2.58 | 8.38 | negative | 13 |  |  |
| 4242 | male | 52 | negative | 124.55 | 10.00 | 1.77 | 12.45 | negative | 12 |  |  |
| 4243 | male | 52 | negative | 84.43 | 2.46 | 2.32 | 34.32 | negative | 12 |  |  |
| 4244 | male | 52 | positive | 148.80 | 12.31 | 2.79 | 12.09 | negative | 13 |  |  |
| 4245 | male | 52 | negative | 216.74 | 8.40 | 2.27 | 25.80 | negative | 12 |  |  |
| 4246 | male | 52 | positive | 129.80 | 13.10 | 16.32 | 9.91 | negative | 15 |  |  |
| 4247 | male | 52 | negative | 53.90 | 7.70 | 2.70 | 7.00 | negative | 12 |  |  |
| 4248 | male | 52 | negative | 67.46 | 5.33 | 2.71 | 12.66 | negative | 12 |  |  |
| 4249 | male | 52 | negative | 109.03 | 10.52 | 2.39 | 10.36 | negative | 12 |  |  |
| 4250 | male | 52 | negative | 132.36 | 9.35 | 1.83 | 14.16 | negative | 12 |  |  |
| 4251 | male | 52 | negative | 121.50 | 11.00 | 1.90 | 11.05 | negative | 12 |  |  |
| 4252 | male | 52 | negative | 150.94 | 15.21 | 13.42 | 9.92 | negative | 14 |  |  |
| 4253 | male | 52 | negative | 118.74 | 12.57 | 15.50 | 9.45 | negative | 14 |  |  |
| 4254 | male | 52 | negative | 54.77 | 3.51 | 2.22 | 15.60 | negative | 12 |  |  |
| 4255 | male | 52 | negative | 125.20 | 11.80 | 2.80 | 10.61 | negative | 12 |  |  |
| 4256 | male | 52 | negative | 114.29 | 10.60 | 2.71 | 10.78 | negative | 12 |  |  |
| 4257 | male | 52 | negative | 98.70 | 10.85 | 2.47 | 9.10 | negative | 12 |  |  |
| 4258 | male | 52 | negative | 93.35 | 6.03 | 2.30 | 15.48 | negative | 12 |  |  |
| 4259 | male | 52 | negative | 92.02 | 10.31 | 2.66 | 8.93 | negative | 12 |  |  |
| 4260 | male | 52 | positive | 150.27 | 22.11 | 10.95 | 6.80 | negative | 15 |  |  |
| 4261 | male | 52 | negative | 165.65 | 7.28 | 2.12 | 22.75 | negative | 12 |  |  |
| 4262 | male | 52 | negative | 133.19 | 13.73 | 2.37 | 9.70 | negative | 12 |  |  |
| 4263 | male | 52 | positive | 164.35 | 13.23 | 2.40 | 12.42 | negative | 13 |  |  |
| 4264 | male | 52 | negative | 116.59 | 8.73 | 2.32 | 13.36 | negative | 12 |  |  |
| 4265 | male | 52 | negative | 78.59 | 4.20 | 16.69 | 18.71 | negative | 14 |  |  |
| 4266 | male | 52 | positive | 127.50 | 16.47 | 10.07 | 7.74 | negative | 15 |  |  |
| 4267 | male | 52 | negative | 41.95 | 2.67 | 2.56 | 15.71 | negative | 12 |  |  |
| 4268 | male | 52 | negative | 168.78 | 12.43 | 1.85 | 13.58 | negative | 12 |  |  |
| 4269 | male | 52 | negative | 77.26 | 3.17 | 2.02 | 24.37 | negative | 12 |  |  |
| 4270 | male | 52 | negative | 163.95 | 7.16 | 2.35 | 22.90 | negative | 12 |  |  |
| 4271 | male | 52 | negative | 56.80 | 4.60 | 1.90 | 12.35 | negative | 12 |  |  |
| 4272 | male | 52 | negative | 85.10 | 14.91 | 19.49 | 5.71 | negative | 14 |  |  |
| 4273 | male | 52 | positive | 203.94 | 10.75 | 12.05 | 18.97 | negative | 15 |  |  |
| 4274 | male | 52 | negative | 65.65 | 3.71 | 1.97 | 17.70 | negative | 12 |  |  |
| 4275 | male | 52 | negative | 86.70 | 7.90 | 2.10 | 10.97 | negative | 12 |  |  |
| 4276 | male | 52 | negative | 288.52 | 17.86 | 16.50 | 16.15 | negative | 14 |  |  |
| 4277 | male | 52 | negative | 60.02 | 4.97 | 2.53 | 12.08 | negative | 12 |  |  |
| 4278 | male | 52 | negative | 312.45 | 22.35 | 17.42 | 13.98 | negative | 14 |  |  |
| 4279 | male | 52 | negative | 98.96 | 5.73 | 2.22 | 17.27 | negative | 12 |  |  |
| 4280 | male | 52 | negative | 107.60 | 10.17 | 2.43 | 10.58 | negative | 12 |  |  |
| 4281 | male | 52 | negative | 93.88 | 6.75 | 1.86 | 13.91 | negative | 12 |  |  |
| 4282 | male | 52 | negative | 106.42 | 9.20 | 2.52 | 11.57 | negative | 12 |  |  |
| 4283 | male | 52 | negative | 168.24 | 9.68 | 2.06 | 17.38 | negative | 12 |  |  |
| 4284 | male | 52 | negative | 83.05 | 5.55 | 1.74 | 14.96 | negative | 12 |  |  |
| 4285 | male | 52 | negative | 163.38 | 9.98 | 1.81 | 16.37 | negative | 12 |  |  |
| 4286 | male | 52 | negative | 74.80 | 6.60 | 2.40 | 11.33 | negative | 12 |  |  |
| 4287 | male | 52 | positive | 185.59 | 13.34 | 2.41 | 13.91 | negative | 13 |  |  |
| 4288 | male | 52 | negative | 44.87 | 1.74 | 2.02 | 25.79 | negative | 12 |  |  |
| 4289 | male | 52 | negative | 103.88 | 12.51 | 1.97 | 8.30 | negative | 12 |  |  |
| 4290 | male | 52 | negative | 132.74 | 6.86 | 2.41 | 19.35 | negative | 12 |  |  |
| 4291 | male | 52 | negative | 78.95 | 8.46 | 1.86 | 9.33 | negative | 12 |  |  |
| 4292 | male | 52 | negative | 140.09 | 7.85 | 2.69 | 17.85 | negative | 12 |  |  |
| 4293 | male | 52 | negative | 177.23 | 11.00 | 11.43 | 16.11 | negative | 14 |  |  |
| 4294 | male | 52 | negative | 95.04 | 5.60 | 2.85 | 16.97 | negative | 12 |  |  |
| 4295 | male | 52 | negative | 140.42 | 9.53 | 2.20 | 14.73 | negative | 12 |  |  |
| 4296 | male | 52 | positive | 82.37 | 5.14 | 2.40 | 16.03 | negative | 13 |  |  |
| 4297 | male | 52 | negative | 192.91 | 12.58 | 2.20 | 15.33 | negative | 12 |  |  |
| 4298 | male | 52 | negative | 73.48 | 4.97 | 2.18 | 14.78 | negative | 12 |  |  |
| 4299 | male | 52 | negative | 51.44 | 3.56 | 2.63 | 14.45 | negative | 12 |  |  |
| 4300 | male | 52 | negative | 107.95 | 6.02 | 2.80 | 17.93 | negative | 12 |  |  |
| 4301 | male | 52 | positive | 147.21 | 17.32 | 12.98 | 8.50 | negative | 15 |  |  |
| 4302 | male | 52 | positive | 93.02 | 10.42 | 2.06 | 8.93 | negative | 13 |  |  |
| 4303 | male | 52 | negative | 97.24 | 18.83 | 13.97 | 5.16 | negative | 14 |  |  |
| 4304 | male | 52 | positive | 63.84 | 10.92 | 2.73 | 5.85 | negative | 13 |  |  |
| 4305 | male | 52 | negative | 72.20 | 7.20 | 1.90 | 10.03 | negative | 12 |  |  |
| 4306 | male | 52 | negative | 111.41 | 8.31 | 18.91 | 13.41 | negative | 14 |  |  |
| 4307 | male | 52 | negative | 140.78 | 17.56 | 18.28 | 8.02 | negative | 14 |  |  |
| 4308 | male | 52 | negative | 80.60 | 5.43 | 2.07 | 14.84 | negative | 12 |  |  |
| 4309 | male | 52 | positive | 93.03 | 16.77 | 17.60 | 5.55 | negative | 15 |  |  |
| 4310 | male | 52 | positive | 65.63 | 7.36 | 1.94 | 8.92 | negative | 13 |  |  |
| 4311 | male | 52 | positive | 225.66 | 12.90 | 2.03 | 17.49 | negative | 13 |  |  |
| 4312 | male | 52 | negative | 69.40 | 11.00 | 1.90 | 6.31 | negative | 12 |  |  |
| 4313 | male | 52 | negative | 104.93 | 6.98 | 2.07 | 15.03 | negative | 12 |  |  |
| 4314 | male | 52 | negative | 149.22 | 20.22 | 11.26 | 7.38 | negative | 14 |  |  |
| 4315 | male | 52 | positive | 119.23 | 19.24 | 16.63 | 6.20 | negative | 15 |  |  |
| 4316 | male | 52 | negative | 109.29 | 5.33 | 1.79 | 20.50 | negative | 12 |  |  |
| 4317 | male | 52 | negative | 121.14 | 6.04 | 1.90 | 20.06 | negative | 12 |  |  |
| 4318 | male | 52 | positive | 85.10 | 12.46 | 10.00 | 6.83 | negative | 15 |  |  |
| 4319 | male | 52 | negative | 48.40 | 4.70 | 2.82 | 10.30 | negative | 12 |  |  |
| 4320 | male | 52 | negative | 66.09 | 4.44 | 1.81 | 14.89 | negative | 12 |  |  |
| 4321 | male | 52 | positive | 112.77 | 10.76 | 2.83 | 10.48 | negative | 13 |  |  |
| 4322 | male | 52 | positive | 144.88 | 17.98 | 10.14 | 8.06 | negative | 15 |  |  |
| 4323 | male | 52 | negative | 92.76 | 9.58 | 10.93 | 9.68 | negative | 14 |  |  |
| 4324 | male | 52 | positive | 116.36 | 15.42 | 11.03 | 7.55 | negative | 15 |  |  |
| 4325 | male | 51 | negative | 165.32 | 13.54 | 9.03 | 12.21 | negative | 14 |  |  |
| 4326 | male | 51 | positive | 42.67 | 13.07 | 9.87 | 3.26 | negative | 18 |  |  |
| 4327 | male | 51 | negative | 154.70 | 13.70 | 8.60 | 11.29 | negative | 14 |  |  |
| 4328 | male | 51 | negative | 293.75 | 15.37 | 5.26 | 19.11 | negative | 12 |  |  |
| 4329 | male | 51 | negative | 123.12 | 11.20 | 6.64 | 10.99 | negative | 14 |  |  |
| 4330 | male | 51 | negative | 300.00 | 16.16 | 6.50 | 18.56 | negative | 14 |  |  |
| 4331 | male | 51 | negative | 46.32 | 2.32 | 9.41 | 19.97 | negative | 14 |  |  |
| 4332 | male | 51 | positive | 104.00 | 14.40 | 7.10 | 7.22 | negative | 15 |  |  |
| 4333 | male | 51 | positive | 188.58 | 20.34 | 7.56 | 9.27 | negative | 15 |  |  |
| 4334 | male | 51 | positive | 107.91 | 15.23 | 5.39 | 7.09 | negative | 13 |  |  |
| 4335 | male | 52 | negative | 115.92 | 7.47 | 1.58 | 15.52 | negative | 12 |  |  |
| 4336 | male | 51 | negative | 193.41 | 13.31 | 5.52 | 14.53 | negative | 12 |  |  |
| 4337 | male | 51 | negative | 113.45 | 9.40 | 4.87 | 12.07 | negative | 12 |  |  |
| 4338 | male | 51 | positive | 105.46 | 7.15 | 7.26 | 14.75 | negative | 15 |  |  |
| 4339 | male | 52 | negative | 138.36 | 9.96 | 1.65 | 13.89 | negative | 12 |  |  |
| 4340 | male | 51 | positive | 99.57 | 11.17 | 8.43 | 8.91 | negative | 15 |  |  |
| 4341 | male | 52 | positive | 129.53 | 11.24 | 1.61 | 11.52 | negative | 13 |  |  |
| 4342 | male | 52 | negative | 78.98 | 6.55 | 1.67 | 12.06 | negative | 12 |  |  |
| 4343 | male | 51 | negative | 90.50 | 7.15 | 5.62 | 12.66 | negative | 12 |  |  |
| 4344 | male | 51 | positive | 83.83 | 6.70 | 8.21 | 12.51 | negative | 15 |  |  |
| 4345 | male | 51 | positive | 99.88 | 10.99 | 8.03 | 9.09 | negative | 15 |  |  |
| 4346 | male | 51 | positive | 161.57 | 11.81 | 8.59 | 13.68 | negative | 15 |  |  |
| 4347 | male | 52 | negative | 106.83 | 4.78 | 1.70 | 22.35 | negative | 12 |  |  |
| 4348 | male | 51 | negative | 125.42 | 9.51 | 5.10 | 13.19 | negative | 12 |  |  |
| 4349 | male | 52 | negative | 116.62 | 12.72 | 1.71 | 9.17 | negative | 12 |  |  |
| 4350 | male | 51 | negative | 120.77 | 20.13 | 5.49 | 6.00 | negative | 12 |  |  |
| 4351 | male | 51 | negative | 123.29 | 13.46 | 5.90 | 9.16 | negative | 14 |  |  |
| 4352 | male | 51 | negative | 203.98 | 13.86 | 8.44 | 14.72 | negative | 14 |  |  |
| 4353 | male | 51 | positive | 115.60 | 16.10 | 6.60 | 7.18 | negative | 15 |  |  |
| 4354 | male | 51 | positive | 163.80 | 15.66 | 7.88 | 10.46 | negative | 15 |  |  |
| 4355 | male | 51 | negative | 215.78 | 23.75 | 8.62 | 9.09 | negative | 14 |  |  |
| 4356 | male | 51 | positive | 82.20 | 6.65 | 6.77 | 12.36 | negative | 15 |  |  |
| 4357 | male | 51 | negative | 145.12 | 11.38 | 6.28 | 12.75 | negative | 14 |  |  |
| 4358 | female | 51 | positive | 109.71 | 30.89 | 5.36 | 3.55 | negative | 12 |  |  |
| 4359 | male | 51 | positive | 81.09 | 10.39 | 5.69 | 7.80 | negative | 13 |  |  |
| 4360 | male | 51 | negative | 75.70 | 15.71 | 56.94 | 4.82 | negative | 14 |  |  |
| 4361 | male | 51 | negative | 130.79 | 9.28 | 5.46 | 14.09 | negative | 12 |  |  |
| 4362 | male | 52 | negative | 181.93 | 13.28 | 1.69 | 13.70 | negative | 12 |  |  |
| 4363 | male | 52 | negative | 153.42 | 24.36 | 1.72 | 6.30 | negative | 12 |  |  |
| 4364 | male | 51 | positive | 114.45 | 10.04 | 5.59 | 11.40 | negative | 13 |  |  |
| 4365 | male | 51 | positive | 131.94 | 14.26 | 5.69 | 9.25 | negative | 13 |  |  |
| 4366 | male | 51 | negative | 112.54 | 8.84 | 4.80 | 12.73 | negative | 12 |  |  |
| 4367 | male | 51 | negative | 143.98 | 17.80 | 5.44 | 8.09 | negative | 12 |  |  |
| 4368 | male | 52 | negative | 74.31 | 3.29 | 1.73 | 22.59 | negative | 12 |  |  |
| 4369 | male | 51 | negative | 48.58 | 11.39 | 48.79 | 4.27 | negative | 14 |  |  |
| 4370 | male | 51 | negative | 139.19 | 13.42 | 4.60 | 10.37 | negative | 12 |  |  |
| 4371 | male | 52 | negative | 143.41 | 9.90 | 1.74 | 14.49 | negative | 12 |  |  |
| 4372 | male | 51 | negative | 126.07 | 11.14 | 7.13 | 11.32 | negative | 14 |  |  |
| 4373 | male | 51 | positive | 100.70 | 16.90 | 5.00 | 5.96 | negative | 13 |  |  |
| 4374 | male | 52 | negative | 103.54 | 13.88 | 1.73 | 7.46 | negative | 12 |  |  |
| 4375 | male | 51 | negative | 97.08 | 8.58 | 4.52 | 11.31 | negative | 12 |  |  |
| 4376 | male | 51 | negative | 62.54 | 5.92 | 5.18 | 10.56 | negative | 12 |  |  |
| 4377 | male | 51 | positive | 150.47 | 14.62 | 5.28 | 10.29 | negative | 13 |  |  |
| 4378 | male | 51 | negative | 117.13 | 9.08 | 5.08 | 12.90 | negative | 12 |  |  |
| 4379 | male | 51 | negative | 132.70 | 8.60 | 7.03 | 15.43 | negative | 14 |  |  |
| 4380 | male | 51 | negative | 115.67 | 5.31 | 7.16 | 21.78 | negative | 14 |  |  |
| 4381 | male | 51 | positive | 161.59 | 9.09 | 6.84 | 17.78 | negative | 15 |  |  |
| 4382 | male | 51 | positive | 70.05 | 17.13 | 4.87 | 4.09 | negative | 13 |  |  |
| 4383 | male | 51 | negative | 102.40 | 7.70 | 6.09 | 13.30 | negative | 14 |  |  |
| 4384 | male | 51 | positive | 186.89 | 15.91 | 7.58 | 11.75 | negative | 15 |  |  |
| 4385 | male | 51 | positive | 72.50 | 12.90 | 5.30 | 5.62 | negative | 13 |  |  |
| 4386 | male | 51 | positive | 79.30 | 14.00 | 7.90 | 5.66 | negative | 15 |  |  |
| 4387 | male | 51 | negative | 63.86 | 11.06 | 4.68 | 5.77 | negative | 12 |  |  |
| 4388 | male | 51 | negative | 102.96 | 7.71 | 5.36 | 13.35 | negative | 12 |  |  |
| 4389 | male | 51 | negative | 107.22 | 6.75 | 8.37 | 15.88 | negative | 14 |  |  |
| 4390 | female | 52 | negative | 19.88 | 10.29 | 60.00 | 1.93 | negative | 13 |  |  |
| 4391 | male | 51 | negative | 37.58 | 5.46 | 55.42 | 6.88 | negative | 14 |  |  |
| 4392 | male | 51 | negative | 151.89 | 11.55 | 6.64 | 13.15 | negative | 14 |  |  |
| 4393 | male | 51 | negative | 33.55 | 8.54 | 55.36 | 3.93 | negative | 14 |  |  |
| 4394 | male | 51 | positive | 210.48 | 20.47 | 5.00 | 10.28 | negative | 13 |  |  |
| 4395 | male | 51 | negative | 67.28 | 8.99 | 5.81 | 7.48 | negative | 14 |  |  |
| 4396 | male | 51 | positive | 120.22 | 15.42 | 7.92 | 7.80 | negative | 15 |  |  |
| 4397 | male | 51 | negative | 46.21 | 7.50 | 5.12 | 6.16 | negative | 12 |  |  |
| 4398 | male | 51 | negative | 121.35 | 6.30 | 9.04 | 19.26 | negative | 14 |  |  |
| 4399 | male | 51 | negative | 174.04 | 8.04 | 8.81 | 21.65 | negative | 14 |  |  |
| 4400 | male | 51 | negative | 49.14 | 5.49 | 7.25 | 8.95 | negative | 14 |  |  |
| 4401 | male | 51 | positive | 91.13 | 12.92 | 6.89 | 7.05 | negative | 15 |  |  |
| 4402 | male | 51 | negative | 275.35 | 26.37 | 6.92 | 10.44 | negative | 14 |  |  |
| 4403 | male | 52 | negative | 277.14 | 10.78 | 1.70 | 25.71 | negative | 12 |  |  |
| 4404 | male | 51 | negative | 131.73 | 10.58 | 6.58 | 12.45 | negative | 14 |  |  |
| 4405 | male | 51 | negative | 105.75 | 5.59 | 7.05 | 18.92 | negative | 14 |  |  |
| 4406 | male | 52 | negative | 92.89 | 8.23 | 1.54 | 11.29 | negative | 12 |  |  |
| 4407 | male | 51 | positive | 91.67 | 7.79 | 4.53 | 11.77 | negative | 13 |  |  |
| 4408 | male | 51 | positive | 300.00 | 20.80 | 5.14 | 14.42 | negative | 13 |  |  |
| 4409 | male | 51 | positive | 113.41 | 11.02 | 6.12 | 10.29 | negative | 15 |  |  |
| 4410 | male | 51 | negative | 130.02 | 15.75 | 5.53 | 8.26 | negative | 12 |  |  |
| 4411 | male | 51 | negative | 112.97 | 10.31 | 5.89 | 10.96 | negative | 14 |  |  |
| 4412 | male | 51 | positive | 102.10 | 13.60 | 9.20 | 7.51 | negative | 15 |  |  |
| 4413 | male | 51 | positive | 45.69 | 14.31 | 5.15 | 3.19 | negative | 16 |  |  |
| 4414 | male | 51 | negative | 212.07 | 14.24 | 8.39 | 14.89 | negative | 14 |  |  |
| 4415 | male | 52 | negative | 84.22 | 4.14 | 1.66 | 20.34 | negative | 12 |  |  |
| 4416 | male | 51 | positive | 182.62 | 22.22 | 6.54 | 8.22 | negative | 15 |  |  |
| 4417 | male | 51 | negative | 78.61 | 8.12 | 7.97 | 9.68 | negative | 14 |  |  |
| 4418 | male | 51 | negative | 120.55 | 9.78 | 4.73 | 12.33 | negative | 12 |  |  |
| 4419 | male | 51 | negative | 75.94 | 7.28 | 5.78 | 10.43 | negative | 14 |  |  |
| 4420 | male | 51 | positive | 112.90 | 11.58 | 5.17 | 9.75 | negative | 13 |  |  |
| 4421 | female | 51 | positive | 62.58 | 16.40 | 5.28 | 3.82 | negative | 12 |  |  |
| 4422 | male | 51 | negative | 77.38 | 7.05 | 4.68 | 10.98 | negative | 12 |  |  |
| 4423 | male | 51 | negative | 41.20 | 8.90 | 42.40 | 4.63 | negative | 14 |  |  |
| 4424 | male | 51 | positive | 80.60 | 12.10 | 7.80 | 6.66 | negative | 15 |  |  |
| 4425 | male | 51 | negative | 154.92 | 21.15 | 5.98 | 7.32 | negative | 14 |  |  |
| 4426 | male | 51 | negative | 112.11 | 10.88 | 5.40 | 10.30 | negative | 12 |  |  |
| 4427 | male | 51 | negative | 179.23 | 15.42 | 9.42 | 11.62 | negative | 14 |  |  |
| 4428 | male | 52 | negative | 87.21 | 7.20 | 1.59 | 12.11 | negative | 12 |  |  |
| 4429 | male | 51 | positive | 96.58 | 12.61 | 7.92 | 7.66 | negative | 15 |  |  |
| 4430 | female | 51 | positive | 73.40 | 23.30 | 43.54 | 3.15 | negative | 14 |  |  |
| 4431 | male | 52 | negative | 100.17 | 9.57 | 1.64 | 10.47 | negative | 12 |  |  |
| 4432 | male | 51 | negative | 14.38 | 9.57 | 60.00 | 1.50 | negative | 17 |  |  |
| 4433 | male | 52 | positive | 117.99 | 8.72 | 1.50 | 13.53 | negative | 13 |  |  |
| 4434 | male | 51 | negative | 91.68 | 5.57 | 5.07 | 16.46 | negative | 12 |  |  |
| 4435 | male | 51 | positive | 155.37 | 15.48 | 6.45 | 10.04 | negative | 15 |  |  |
| 4436 | male | 51 | positive | 95.89 | 11.13 | 5.92 | 8.62 | negative | 15 |  |  |
| 4437 | male | 51 | negative | 52.21 | 12.21 | 5.09 | 4.28 | negative | 12 |  |  |
| 4438 | male | 51 | negative | 61.64 | 5.38 | 5.09 | 11.46 | negative | 12 |  |  |
| 4439 | male | 51 | negative | 95.81 | 7.60 | 4.83 | 12.61 | negative | 12 |  |  |
| 4440 | male | 51 | positive | 206.25 | 17.00 | 5.94 | 12.13 | negative | 15 |  |  |
| 4441 | male | 51 | positive | 118.81 | 14.98 | 4.97 | 7.93 | negative | 13 |  |  |
| 4442 | male | 51 | negative | 123.34 | 13.05 | 5.58 | 9.45 | negative | 12 |  |  |
| 4443 | male | 51 | negative | 159.44 | 14.38 | 4.92 | 11.09 | negative | 12 |  |  |
| 4444 | male | 51 | positive | 135.94 | 11.91 | 9.76 | 11.41 | negative | 15 |  |  |
| 4445 | male | 51 | positive | 147.28 | 24.72 | 7.63 | 5.96 | negative | 15 |  |  |
| 4446 | male | 51 | positive | 174.87 | 12.09 | 8.47 | 14.46 | negative | 15 |  |  |
| 4447 | male | 51 | positive | 127.30 | 11.33 | 6.54 | 11.24 | negative | 15 |  |  |
| 4448 | male | 51 | positive | 93.53 | 10.98 | 8.35 | 8.52 | negative | 15 |  |  |
| 4449 | male | 51 | negative | 167.54 | 22.40 | 6.27 | 7.48 | negative | 14 |  |  |
| 4450 | male | 51 | negative | 84.26 | 8.95 | 4.70 | 9.41 | negative | 12 |  |  |
| 4451 | male | 52 | negative | 190.10 | 9.60 | 1.65 | 19.80 | negative | 12 |  |  |
| 4452 | male | 51 | positive | 162.09 | 10.01 | 9.46 | 16.19 | negative | 15 |  |  |
| 4453 | male | 51 | positive | 179.36 | 19.43 | 5.78 | 9.23 | negative | 15 |  |  |
| 4454 | male | 51 | positive | 137.92 | 12.91 | 7.11 | 10.68 | negative | 15 |  |  |
| 4455 | male | 51 | negative | 101.60 | 5.04 | 3.64 | 20.16 | negative | 12 |  |  |
| 4456 | male | 51 | negative | 89.29 | 6.92 | 2.40 | 12.90 | negative | 12 |  |  |
| 4457 | male | 51 | positive | 131.48 | 17.14 | 28.06 | 7.67 | negative | 15 |  |  |
| 4458 | male | 51 | negative | 160.84 | 8.48 | 2.32 | 18.97 | negative | 12 |  |  |
| 4459 | male | 51 | positive | 287.62 | 18.61 | 4.20 | 15.46 | negative | 13 |  |  |
| 4460 | male | 51 | negative | 122.30 | 7.56 | 4.17 | 16.18 | negative | 12 |  |  |
| 4461 | male | 51 | negative | 135.71 | 15.62 | 2.27 | 8.69 | negative | 12 |  |  |
| 4462 | male | 51 | negative | 88.08 | 4.38 | 2.11 | 20.11 | negative | 12 |  |  |
| 4463 | male | 51 | negative | 136.10 | 10.00 | 31.30 | 13.61 | negative | 14 |  |  |
| 4464 | male | 51 | negative | 106.26 | 5.83 | 2.74 | 18.23 | negative | 12 |  |  |
| 4465 | male | 51 | negative | 119.06 | 8.87 | 3.79 | 13.42 | negative | 12 |  |  |
| 4466 | male | 51 | positive | 226.40 | 23.60 | 22.10 | 9.59 | negative | 15 |  |  |
| 4467 | male | 51 | negative | 80.88 | 4.82 | 4.49 | 16.78 | negative | 12 |  |  |
| 4468 | male | 51 | negative | 74.34 | 8.44 | 3.67 | 8.81 | negative | 12 |  |  |
| 4469 | male | 51 | negative | 78.87 | 4.21 | 2.13 | 18.73 | negative | 12 |  |  |
| 4470 | male | 51 | positive | 268.11 | 14.27 | 3.54 | 18.79 | negative | 13 |  |  |
| 4471 | male | 51 | negative | 77.76 | 3.27 | 2.12 | 23.78 | negative | 12 |  |  |
| 4472 | male | 51 | negative | 128.45 | 10.78 | 2.84 | 11.92 | negative | 12 |  |  |
| 4473 | male | 51 | negative | 134.33 | 5.69 | 2.68 | 23.61 | negative | 12 |  |  |
| 4474 | male | 51 | negative | 182.27 | 9.25 | 3.81 | 19.70 | negative | 12 |  |  |
| 4475 | male | 51 | negative | 122.99 | 6.28 | 4.07 | 19.58 | negative | 12 |  |  |
| 4476 | male | 51 | negative | 98.28 | 5.62 | 3.17 | 17.49 | negative | 12 |  |  |
| 4477 | male | 51 | negative | 87.69 | 5.72 | 3.16 | 15.33 | negative | 12 |  |  |
| 4478 | male | 51 | negative | 74.32 | 6.09 | 2.51 | 12.20 | negative | 12 |  |  |
| 4479 | male | 51 | negative | 88.70 | 9.50 | 2.90 | 9.34 | negative | 12 |  |  |
| 4480 | male | 51 | negative | 99.70 | 11.90 | 2.40 | 8.38 | negative | 12 |  |  |
| 4481 | male | 51 | negative | 55.55 | 5.68 | 2.10 | 9.78 | negative | 12 |  |  |
| 4482 | male | 51 | negative | 72.54 | 9.20 | 4.44 | 7.88 | negative | 12 |  |  |
| 4483 | male | 51 | negative | 70.28 | 3.21 | 2.58 | 21.89 | negative | 12 |  |  |
| 4484 | male | 51 | negative | 128.38 | 7.39 | 2.89 | 17.37 | negative | 12 |  |  |
| 4485 | male | 51 | negative | 98.78 | 6.65 | 2.82 | 14.85 | negative | 12 |  |  |
| 4486 | male | 51 | negative | 285.55 | 26.95 | 3.62 | 10.60 | negative | 12 |  |  |
| 4487 | male | 51 | negative | 85.42 | 7.99 | 2.28 | 10.69 | negative | 12 |  |  |
| 4488 | male | 51 | negative | 113.22 | 4.70 | 3.80 | 24.09 | negative | 12 |  |  |
| 4489 | male | 51 | positive | 178.36 | 13.46 | 2.98 | 13.25 | negative | 13 |  |  |
| 4490 | male | 51 | negative | 122.89 | 7.16 | 4.07 | 17.16 | negative | 12 |  |  |
| 4491 | male | 51 | positive | 205.06 | 16.59 | 2.72 | 12.36 | negative | 13 |  |  |
| 4492 | male | 51 | positive | 85.66 | 6.20 | 2.17 | 13.82 | negative | 13 |  |  |
| 4493 | male | 51 | negative | 153.47 | 12.76 | 2.67 | 12.03 | negative | 12 |  |  |
| 4494 | male | 51 | negative | 112.68 | 7.33 | 3.48 | 15.37 | negative | 12 |  |  |
| 4495 | male | 51 | negative | 68.47 | 5.06 | 3.61 | 13.53 | negative | 12 |  |  |
| 4496 | male | 51 | negative | 235.53 | 13.37 | 26.34 | 17.62 | negative | 14 |  |  |
| 4497 | male | 51 | positive | 154.20 | 33.11 | 25.76 | 4.66 | negative | 15 |  |  |
| 4498 | male | 51 | negative | 71.80 | 10.30 | 2.90 | 6.97 | negative | 12 |  |  |
| 4499 | male | 51 | negative | 76.80 | 6.50 | 4.50 | 11.82 | negative | 12 |  |  |
| 4500 | male | 51 | positive | 79.52 | 15.34 | 2.97 | 5.18 | negative | 13 |  |  |
| 4501 | male | 51 | negative | 71.65 | 5.93 | 3.53 | 12.08 | negative | 12 |  |  |
| 4502 | male | 51 | negative | 82.41 | 3.24 | 3.35 | 25.44 | negative | 12 |  |  |
| 4503 | male | 51 | negative | 139.26 | 11.57 | 2.70 | 12.04 | negative | 12 |  |  |
| 4504 | male | 51 | positive | 98.70 | 14.14 | 4.48 | 6.98 | negative | 13 |  |  |
| 4505 | male | 51 | negative | 193.94 | 12.04 | 2.42 | 16.11 | negative | 12 |  |  |
| 4506 | male | 51 | negative | 133.80 | 11.66 | 3.23 | 11.48 | negative | 12 |  |  |
| 4507 | male | 51 | negative | 140.50 | 11.60 | 3.50 | 12.11 | negative | 12 |  |  |
| 4508 | male | 51 | negative | 110.06 | 8.91 | 4.05 | 12.35 | negative | 12 |  |  |
| 4509 | male | 51 | negative | 138.85 | 10.47 | 3.78 | 13.26 | negative | 12 |  |  |
| 4510 | male | 51 | negative | 114.40 | 9.46 | 3.22 | 12.09 | negative | 12 |  |  |
| 4511 | male | 51 | positive | 110.59 | 10.74 | 2.20 | 10.30 | negative | 13 |  |  |
| 4512 | male | 51 | negative | 159.01 | 9.87 | 3.52 | 16.11 | negative | 12 |  |  |
| 4513 | male | 51 | positive | 96.39 | 14.35 | 3.25 | 6.72 | negative | 13 |  |  |
| 4514 | male | 51 | negative | 118.61 | 4.67 | 3.19 | 25.40 | negative | 12 |  |  |
| 4515 | male | 51 | negative | 73.26 | 5.30 | 2.28 | 13.82 | negative | 12 |  |  |
| 4516 | male | 51 | negative | 97.14 | 9.13 | 2.13 | 10.64 | negative | 12 |  |  |
| 4517 | male | 51 | positive | 67.36 | 14.32 | 20.75 | 4.70 | negative | 15 |  |  |
| 4518 | male | 51 | negative | 75.90 | 5.18 | 2.24 | 14.65 | negative | 12 |  |  |
| 4519 | male | 51 | positive | 110.35 | 11.05 | 3.85 | 9.99 | negative | 13 |  |  |
| 4520 | male | 51 | positive | 155.91 | 13.20 | 3.60 | 11.81 | negative | 13 |  |  |
| 4521 | male | 51 | positive | 103.59 | 12.08 | 26.93 | 8.58 | negative | 15 |  |  |
| 4522 | male | 51 | negative | 138.82 | 10.35 | 3.21 | 13.41 | negative | 12 |  |  |
| 4523 | male | 51 | negative | 68.15 | 5.17 | 2.56 | 13.18 | negative | 12 |  |  |
| 4524 | male | 51 | negative | 220.27 | 9.46 | 2.38 | 23.28 | negative | 12 |  |  |
| 4525 | male | 51 | positive | 195.21 | 10.37 | 3.58 | 18.82 | negative | 13 |  |  |
| 4526 | male | 51 | negative | 105.32 | 5.92 | 2.64 | 17.79 | negative | 12 |  |  |
| 4527 | male | 51 | negative | 94.97 | 10.31 | 3.10 | 9.21 | negative | 12 |  |  |
| 4528 | male | 51 | positive | 123.79 | 9.36 | 4.13 | 13.23 | negative | 13 |  |  |
| 4529 | male | 51 | negative | 153.01 | 8.72 | 2.41 | 17.55 | negative | 12 |  |  |
| 4530 | male | 51 | negative | 107.04 | 8.38 | 2.60 | 12.77 | negative | 12 |  |  |
| 4531 | male | 51 | negative | 56.25 | 15.68 | 3.26 | 3.59 | negative | 15 |  |  |
| 4532 | male | 51 | negative | 71.49 | 14.99 | 2.77 | 4.77 | negative | 12 |  |  |
| 4533 | male | 51 | negative | 43.87 | 3.49 | 2.37 | 12.57 | negative | 12 |  |  |
| 4534 | male | 51 | negative | 90.37 | 6.19 | 2.79 | 14.60 | negative | 12 |  |  |
| 4535 | male | 51 | negative | 113.56 | 11.63 | 2.99 | 9.76 | negative | 12 |  |  |
| 4536 | male | 51 | negative | 94.77 | 11.65 | 4.12 | 8.13 | negative | 12 |  |  |
| 4537 | male | 51 | positive | 140.15 | 11.47 | 4.48 | 12.22 | negative | 13 |  |  |
| 4538 | male | 51 | positive | 85.75 | 7.83 | 2.54 | 10.95 | negative | 13 |  |  |
| 4539 | male | 51 | negative | 146.46 | 9.99 | 2.16 | 14.66 | negative | 12 |  |  |
| 4540 | male | 51 | positive | 100.40 | 29.90 | 25.90 | 3.36 | negative | 18 |  |  |
| 4541 | male | 51 | negative | 129.85 | 6.71 | 3.00 | 19.35 | negative | 12 |  |  |
| 4542 | male | 51 | negative | 52.29 | 4.83 | 3.22 | 10.83 | negative | 12 |  |  |
| 4543 | male | 51 | negative | 125.53 | 8.61 | 4.27 | 14.58 | negative | 12 |  |  |
| 4544 | male | 51 | negative | 84.60 | 8.10 | 3.90 | 10.44 | negative | 12 |  |  |
| 4545 | male | 51 | positive | 126.44 | 7.68 | 2.61 | 16.46 | negative | 13 |  |  |
| 4546 | male | 51 | negative | 131.76 | 6.70 | 2.40 | 19.67 | negative | 12 |  |  |
| 4547 | male | 51 | positive | 121.75 | 11.12 | 2.80 | 10.95 | negative | 13 |  |  |
| 4548 | male | 51 | negative | 146.49 | 9.04 | 3.12 | 16.20 | negative | 12 |  |  |
| 4549 | male | 51 | negative | 139.84 | 7.43 | 2.15 | 18.82 | negative | 12 |  |  |
| 4550 | male | 51 | negative | 109.60 | 6.92 | 4.30 | 15.84 | negative | 12 |  |  |
| 4551 | male | 51 | negative | 113.99 | 7.66 | 2.78 | 14.88 | negative | 12 |  |  |
| 4552 | female | 51 | positive | 59.48 | 18.10 | 4.36 | 3.29 | negative | 12 |  |  |
| 4553 | male | 51 | negative | 76.00 | 10.70 | 2.20 | 7.10 | negative | 12 |  |  |
| 4554 | male | 51 | positive | 257.37 | 13.42 | 3.73 | 19.18 | negative | 13 |  |  |
| 4555 | male | 51 | negative | 85.97 | 5.57 | 2.46 | 15.43 | negative | 12 |  |  |
| 4556 | male | 51 | negative | 51.64 | 5.73 | 2.29 | 9.01 | negative | 12 |  |  |
| 4557 | male | 51 | negative | 80.08 | 3.51 | 2.84 | 22.81 | negative | 12 |  |  |
| 4558 | male | 51 | positive | 137.76 | 12.02 | 4.11 | 11.46 | negative | 13 |  |  |
| 4559 | male | 51 | negative | 88.30 | 8.04 | 3.25 | 10.98 | negative | 12 |  |  |
| 4560 | male | 51 | negative | 141.62 | 6.60 | 3.08 | 21.46 | negative | 12 |  |  |
| 4561 | male | 51 | negative | 181.58 | 9.22 | 2.38 | 19.69 | negative | 12 |  |  |
| 4562 | male | 51 | negative | 125.21 | 9.41 | 2.37 | 13.31 | negative | 12 |  |  |
| 4563 | male | 51 | negative | 86.24 | 3.46 | 2.72 | 24.92 | negative | 12 |  |  |
| 4564 | male | 51 | negative | 75.42 | 4.52 | 3.20 | 16.69 | negative | 12 |  |  |
| 4565 | male | 51 | positive | 132.59 | 23.22 | 3.23 | 5.71 | negative | 13 |  |  |
| 4566 | male | 51 | negative | 129.06 | 10.29 | 2.67 | 12.54 | negative | 12 |  |  |
| 4567 | male | 51 | negative | 175.21 | 23.15 | 3.80 | 7.57 | negative | 12 |  |  |
| 4568 | male | 51 | negative | 120.47 | 6.81 | 2.51 | 17.69 | negative | 12 |  |  |
| 4569 | male | 51 | positive | 150.12 | 19.89 | 21.31 | 7.55 | negative | 15 |  |  |
| 4570 | male | 51 | positive | 119.97 | 11.27 | 3.42 | 10.65 | negative | 13 |  |  |
| 4571 | male | 51 | negative | 83.85 | 5.70 | 2.65 | 14.71 | negative | 12 |  |  |
| 4572 | male | 51 | negative | 70.16 | 13.21 | 24.59 | 5.31 | negative | 14 |  |  |
| 4573 | male | 51 | negative | 101.28 | 13.22 | 4.31 | 7.66 | negative | 12 |  |  |
| 4574 | male | 51 | positive | 116.39 | 14.55 | 4.36 | 8.00 | negative | 13 |  |  |
| 4575 | male | 51 | negative | 78.84 | 5.78 | 2.41 | 13.64 | negative | 12 |  |  |
| 4576 | male | 51 | negative | 92.09 | 7.68 | 2.91 | 11.99 | negative | 12 |  |  |
| 4577 | male | 51 | positive | 100.99 | 9.52 | 2.72 | 10.61 | negative | 13 |  |  |
| 4578 | male | 51 | negative | 155.71 | 14.92 | 2.36 | 10.44 | negative | 12 |  |  |
| 4579 | male | 51 | negative | 214.05 | 17.81 | 3.95 | 12.02 | negative | 12 |  |  |
| 4580 | male | 51 | negative | 145.23 | 9.11 | 2.49 | 15.94 | negative | 12 |  |  |
| 4581 | male | 51 | negative | 97.72 | 7.51 | 2.47 | 13.01 | negative | 12 |  |  |
| 4582 | male | 51 | positive | 114.30 | 11.66 | 20.69 | 9.80 | negative | 15 |  |  |
| 4583 | male | 51 | negative | 181.97 | 15.32 | 2.42 | 11.88 | negative | 12 |  |  |
| 4584 | male | 51 | negative | 108.10 | 11.90 | 4.00 | 9.08 | negative | 12 |  |  |
| 4585 | male | 51 | negative | 81.01 | 4.22 | 2.16 | 19.20 | negative | 12 |  |  |
| 4586 | male | 51 | negative | 89.78 | 8.36 | 4.46 | 10.74 | negative | 12 |  |  |
| 4587 | male | 51 | positive | 76.73 | 9.93 | 3.12 | 7.73 | negative | 13 |  |  |
| 4588 | male | 51 | negative | 147.61 | 8.19 | 2.17 | 18.02 | negative | 12 |  |  |
| 4589 | male | 51 | positive | 183.03 | 22.83 | 4.18 | 8.02 | negative | 13 |  |  |
| 4590 | male | 51 | negative | 79.16 | 9.08 | 2.68 | 8.72 | negative | 12 |  |  |
| 4591 | male | 51 | negative | 145.48 | 8.99 | 2.31 | 16.18 | negative | 12 |  |  |
| 4592 | male | 51 | positive | 152.88 | 13.00 | 2.57 | 11.76 | negative | 13 |  |  |
| 4593 | male | 51 | negative | 232.76 | 9.72 | 3.07 | 23.95 | negative | 12 |  |  |
| 4594 | male | 51 | negative | 79.77 | 4.22 | 2.85 | 18.90 | negative | 12 |  |  |
| 4595 | male | 51 | negative | 129.80 | 9.67 | 3.94 | 13.42 | negative | 12 |  |  |
| 4596 | male | 51 | positive | 200.19 | 14.34 | 3.26 | 13.96 | negative | 13 |  |  |
| 4597 | male | 51 | negative | 98.85 | 6.45 | 2.70 | 15.33 | negative | 12 |  |  |
| 4598 | male | 51 | negative | 79.03 | 10.59 | 2.42 | 7.46 | negative | 12 |  |  |
| 4599 | male | 51 | negative | 65.60 | 8.10 | 4.00 | 8.10 | negative | 12 |  |  |
| 4600 | male | 51 | negative | 172.58 | 9.00 | 2.46 | 19.18 | negative | 12 |  |  |
| 4601 | male | 51 | negative | 40.54 | 16.66 | 39.55 | 2.43 | negative | 17 |  |  |
| 4602 | male | 51 | negative | 76.89 | 4.67 | 4.16 | 16.46 | negative | 12 |  |  |
| 4603 | male | 51 | negative | 86.10 | 13.90 | 2.60 | 6.19 | negative | 12 |  |  |
| 4604 | male | 51 | negative | 105.13 | 8.89 | 2.65 | 11.83 | negative | 12 |  |  |
| 4605 | male | 51 | positive | 159.82 | 9.24 | 3.40 | 17.30 | negative | 13 |  |  |
| 4606 | male | 51 | negative | 111.57 | 6.37 | 4.31 | 17.51 | negative | 12 |  |  |
| 4607 | male | 51 | negative | 223.64 | 7.81 | 2.13 | 28.64 | negative | 12 |  |  |
| 4608 | male | 51 | negative | 101.26 | 7.50 | 2.83 | 13.50 | negative | 12 |  |  |
| 4609 | male | 51 | positive | 109.24 | 14.37 | 3.84 | 7.60 | negative | 13 |  |  |
| 4610 | male | 51 | negative | 291.77 | 11.18 | 2.60 | 26.10 | negative | 12 |  |  |
| 4611 | male | 51 | negative | 163.90 | 9.23 | 2.76 | 17.76 | negative | 12 |  |  |
| 4612 | male | 51 | negative | 99.93 | 5.89 | 2.25 | 16.97 | negative | 12 |  |  |
| 4613 | male | 51 | positive | 123.82 | 11.83 | 2.38 | 10.47 | negative | 13 |  |  |
| 4614 | male | 51 | negative | 129.64 | 8.65 | 4.47 | 14.99 | negative | 12 |  |  |
| 4615 | male | 51 | negative | 120.28 | 8.75 | 3.02 | 13.75 | negative | 12 |  |  |
| 4616 | male | 51 | negative | 107.15 | 5.26 | 4.34 | 20.37 | negative | 12 |  |  |
| 4617 | male | 51 | negative | 184.03 | 10.10 | 1.98 | 18.22 | negative | 12 |  |  |
| 4618 | male | 50 | negative | 112.10 | 9.92 | 9.25 | 11.30 | negative | 14 |  |  |
| 4619 | male | 51 | positive | 234.15 | 24.21 | 12.89 | 9.67 | negative | 15 |  |  |
| 4620 | male | 50 | positive | 163.11 | 17.73 | 9.64 | 9.20 | negative | 15 |  |  |
| 4621 | male | 51 | positive | 143.62 | 14.10 | 11.19 | 10.19 | negative | 15 |  |  |
| 4622 | male | 51 | negative | 94.49 | 9.40 | 1.94 | 10.05 | negative | 12 |  |  |
| 4623 | male | 51 | positive | 207.71 | 27.80 | 11.73 | 7.47 | negative | 15 |  |  |
| 4624 | male | 51 | negative | 92.17 | 6.87 | 1.70 | 13.42 | negative | 12 |  |  |
| 4625 | male | 51 | negative | 148.85 | 10.38 | 13.64 | 14.34 | negative | 14 |  |  |
| 4626 | male | 51 | negative | 189.84 | 24.22 | 17.12 | 7.84 | negative | 14 |  |  |
| 4627 | male | 50 | positive | 134.11 | 10.50 | 9.21 | 12.77 | negative | 15 |  |  |
| 4628 | male | 51 | negative | 95.40 | 5.02 | 1.58 | 19.00 | negative | 12 |  |  |
| 4629 | male | 51 | positive | 118.50 | 20.60 | 11.70 | 5.75 | negative | 15 |  |  |
| 4630 | male | 51 | negative | 75.60 | 14.90 | 18.70 | 5.07 | negative | 14 |  |  |
| 4631 | male | 51 | positive | 112.85 | 15.34 | 13.60 | 7.36 | negative | 15 |  |  |
| 4632 | male | 51 | negative | 62.72 | 7.13 | 1.94 | 8.80 | negative | 12 |  |  |
| 4633 | male | 51 | positive | 191.44 | 15.81 | 14.86 | 12.11 | negative | 15 |  |  |
| 4634 | male | 51 | positive | 229.63 | 24.70 | 13.57 | 9.30 | negative | 15 |  |  |
| 4635 | male | 51 | positive | 285.36 | 26.47 | 17.26 | 10.78 | negative | 15 |  |  |
| 4636 | male | 50 | positive | 138.85 | 11.60 | 9.69 | 11.97 | negative | 15 |  |  |
| 4637 | male | 51 | negative | 93.00 | 10.20 | 10.10 | 9.12 | negative | 14 |  |  |
| 4638 | male | 50 | negative | 127.43 | 10.24 | 9.01 | 12.44 | negative | 14 |  |  |
| 4639 | male | 51 | negative | 68.16 | 4.43 | 1.81 | 15.39 | negative | 12 |  |  |
| 4640 | male | 51 | negative | 122.20 | 5.77 | 1.55 | 21.18 | negative | 12 |  |  |
| 4641 | male | 51 | negative | 107.91 | 9.86 | 1.89 | 10.94 | negative | 12 |  |  |
| 4642 | male | 51 | positive | 114.71 | 9.09 | 16.17 | 12.62 | negative | 15 |  |  |
| 4643 | male | 51 | negative | 103.08 | 7.08 | 1.57 | 14.56 | negative | 12 |  |  |
| 4644 | male | 51 | positive | 92.07 | 7.77 | 1.65 | 11.85 | negative | 13 |  |  |
| 4645 | male | 51 | positive | 107.55 | 9.64 | 12.24 | 11.16 | negative | 15 |  |  |
| 4646 | male | 51 | negative | 154.46 | 14.70 | 13.25 | 10.51 | negative | 14 |  |  |
| 4647 | male | 51 | negative | 67.65 | 15.98 | 12.14 | 4.23 | negative | 14 |  |  |
| 4648 | male | 51 | negative | 130.78 | 5.74 | 1.97 | 22.78 | negative | 12 |  |  |
| 4649 | male | 51 | negative | 155.39 | 10.93 | 11.47 | 14.22 | negative | 14 |  |  |
| 4650 | male | 50 | positive | 112.62 | 10.69 | 8.42 | 10.54 | negative | 15 |  |  |
| 4651 | male | 51 | negative | 66.32 | 7.61 | 1.91 | 8.71 | negative | 12 |  |  |
| 4652 | male | 51 | negative | 89.53 | 5.50 | 1.88 | 16.28 | negative | 12 |  |  |
| 4653 | male | 51 | negative | 95.51 | 6.18 | 1.85 | 15.45 | negative | 12 |  |  |
| 4654 | male | 51 | negative | 100.34 | 4.22 | 1.98 | 23.78 | negative | 12 |  |  |
| 4655 | male | 51 | negative | 67.58 | 9.58 | 1.82 | 7.05 | negative | 12 |  |  |
| 4656 | male | 51 | negative | 111.49 | 11.13 | 1.74 | 10.02 | negative | 12 |  |  |
| 4657 | male | 50 | positive | 92.56 | 13.12 | 9.37 | 7.05 | negative | 15 |  |  |
| 4658 | male | 51 | negative | 65.49 | 5.75 | 1.77 | 11.39 | negative | 12 |  |  |
| 4659 | male | 51 | positive | 98.27 | 12.40 | 16.75 | 7.93 | negative | 15 |  |  |
| 4660 | male | 51 | negative | 149.58 | 17.37 | 17.45 | 8.61 | negative | 14 |  |  |
| 4661 | male | 51 | negative | 119.99 | 7.52 | 2.06 | 15.96 | negative | 12 |  |  |
| 4662 | male | 51 | negative | 80.66 | 5.45 | 1.88 | 14.80 | negative | 12 |  |  |
| 4663 | male | 51 | negative | 94.72 | 6.62 | 2.07 | 14.31 | negative | 12 |  |  |
| 4664 | male | 51 | negative | 140.10 | 14.98 | 11.76 | 9.35 | negative | 14 |  |  |
| 4665 | male | 51 | negative | 84.00 | 8.20 | 1.80 | 10.24 | negative | 12 |  |  |
| 4666 | male | 51 | negative | 148.58 | 19.53 | 10.83 | 7.61 | negative | 14 |  |  |
| 4667 | male | 50 | positive | 88.90 | 14.90 | 8.30 | 5.97 | negative | 15 |  |  |
| 4668 | male | 51 | negative | 90.39 | 11.42 | 1.91 | 7.92 | negative | 12 |  |  |
| 4669 | male | 51 | positive | 165.12 | 17.73 | 13.12 | 9.31 | negative | 15 |  |  |
| 4670 | male | 51 | negative | 159.89 | 15.81 | 1.98 | 10.11 | negative | 12 |  |  |
| 4671 | male | 51 | negative | 134.65 | 10.10 | 1.79 | 13.33 | negative | 12 |  |  |
| 4672 | male | 51 | positive | 143.96 | 15.50 | 17.65 | 9.29 | negative | 15 |  |  |
| 4673 | male | 51 | negative | 151.49 | 11.42 | 1.91 | 13.27 | negative | 12 |  |  |
| 4674 | male | 51 | negative | 284.59 | 19.62 | 1.72 | 14.51 | negative | 12 |  |  |
| 4675 | male | 51 | negative | 144.37 | 7.39 | 2.08 | 19.54 | negative | 12 |  |  |
| 4676 | male | 51 | negative | 103.50 | 7.99 | 1.83 | 12.95 | negative | 12 |  |  |
| 4677 | male | 51 | negative | 78.71 | 3.51 | 1.66 | 22.42 | negative | 12 |  |  |
| 4678 | male | 51 | negative | 79.81 | 6.47 | 2.05 | 12.34 | negative | 12 |  |  |
| 4679 | male | 51 | positive | 177.30 | 21.02 | 11.46 | 8.43 | negative | 15 |  |  |
| 4680 | male | 51 | negative | 103.98 | 8.58 | 1.96 | 12.12 | negative | 12 |  |  |
| 4681 | male | 51 | negative | 122.70 | 4.24 | 1.98 | 28.94 | negative | 12 |  |  |
| 4682 | male | 51 | positive | 158.27 | 13.92 | 10.89 | 11.37 | negative | 15 |  |  |
| 4683 | male | 50 | positive | 140.49 | 15.42 | 8.52 | 9.11 | negative | 15 |  |  |
| 4684 | male | 51 | negative | 145.30 | 11.92 | 1.59 | 12.19 | negative | 12 |  |  |
| 4685 | male | 51 | positive | 112.46 | 21.19 | 19.93 | 5.31 | negative | 15 |  |  |
| 4686 | male | 50 | positive | 132.50 | 14.05 | 8.25 | 9.43 | negative | 15 |  |  |
| 4687 | male | 50 | positive | 219.40 | 20.34 | 9.34 | 10.79 | negative | 15 |  |  |
| 4688 | male | 51 | positive | 85.78 | 13.35 | 14.48 | 6.43 | negative | 15 |  |  |
| 4689 | male | 51 | negative | 133.51 | 8.06 | 1.84 | 16.56 | negative | 12 |  |  |
| 4690 | male | 51 | negative | 187.87 | 10.13 | 1.97 | 18.55 | negative | 12 |  |  |
| 4691 | male | 50 | negative | 115.07 | 8.04 | 9.65 | 14.31 | negative | 14 |  |  |
| 4692 | male | 51 | negative | 80.67 | 4.66 | 1.52 | 17.31 | negative | 12 |  |  |
| 4693 | male | 51 | negative | 91.15 | 5.81 | 1.77 | 15.69 | negative | 12 |  |  |
| 4694 | male | 51 | negative | 215.94 | 10.35 | 1.83 | 20.86 | negative | 12 |  |  |
| 4695 | male | 51 | negative | 91.60 | 6.07 | 1.75 | 15.09 | negative | 12 |  |  |
| 4696 | male | 51 | positive | 80.50 | 14.80 | 10.70 | 5.44 | negative | 15 |  |  |
| 4697 | male | 51 | negative | 116.73 | 11.56 | 2.08 | 10.10 | negative | 12 |  |  |
| 4698 | male | 51 | negative | 86.55 | 5.83 | 1.58 | 14.85 | negative | 12 |  |  |
| 4699 | male | 51 | negative | 100.86 | 3.41 | 2.02 | 29.58 | negative | 12 |  |  |
| 4700 | male | 51 | negative | 107.54 | 7.56 | 1.92 | 14.22 | negative | 12 |  |  |
| 4701 | male | 51 | negative | 106.13 | 13.18 | 1.95 | 8.05 | negative | 12 |  |  |
| 4702 | male | 51 | negative | 51.71 | 9.65 | 1.89 | 5.36 | negative | 12 |  |  |
| 4703 | male | 51 | negative | 89.50 | 8.00 | 1.70 | 11.19 | negative | 12 |  |  |
| 4704 | male | 50 | positive | 241.70 | 38.50 | 8.50 | 6.28 | negative | 15 |  |  |
| 4705 | male | 50 | negative | 141.85 | 13.88 | 9.18 | 10.22 | negative | 14 |  |  |
[truncated: 306,546 more chars]
